# C2-Linked Arabinose-Functionalized Polystyrene Microbeads Selectively Target *Staphylococcus aureus*

**DOI:** 10.1021/jacsau.4c00931

**Published:** 2024-11-08

**Authors:** Gulab Walke, Cristina Santi, Calum Haydon, Pooja Joshi, Yuiko Takebayashi, Sylvain Rama, Josephine Dorh, Srinivas Hotha, James Spencer, M. Carmen Galan

**Affiliations:** a School of Chemistry, University of Bristol, Cantock’s Close, Bristol BS8 1TS, United Kingdom; b School of Cellular and Molecular Medicine, University of Bristol Biomedical Sciences Building, Bristol BS8 1TD, United Kingdom; c Department of Chemistry, Indian Institute of Science Education and Research Pune, Pune 411 008, India; d FluoretiQ Ltd., Futurespace, Filton Road, Stoke Gifford, Bristol BS34 8RB, United Kingdom

**Keywords:** antimicrobial probes, oligosaccharide synthesis, nonmamalian glycans, bacteria targeting, agglutination

## Abstract

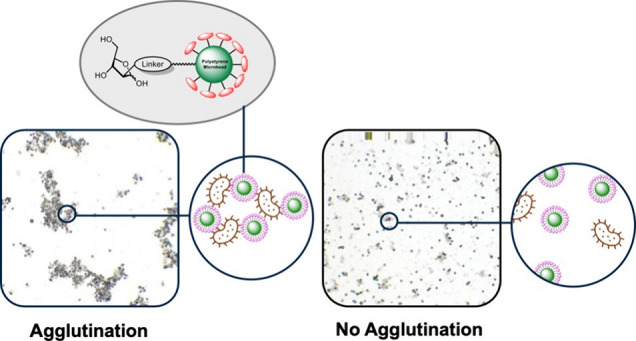

Carbohydrates play pivotal roles in the first stages of microbial infections and can be exploited as decoys to hijack the interactions between bacteria and the host cell. Multivalent glycan probes mimicking the natural presentation of glycans in living cells have been successfully employed to study fundamental carbohydrate/protein interactions in microbial systems; however, most pathogenic glycan receptors exhibit a shared specificity for commonly found sugars present in both healthy and pathogenic cells, posing a challenge for target selectivity. In this study, we report the synthesis of a small library of d-arabinose multivalent probes, a sugar absent in human physiology, and their evaluation in a bacteria agglutination assay using cluster analysis. Our findings reveal preferential binding to *Staphylococcus aureus* of C2-linked arabinose moieties over C1- or C5-linked probes, underscoring the importance of glycan presentation in targeting specificity. Furthermore, we demonstrate the selectivity of the C2-linked probe toward *S. aureus* across a panel of common bacterial pathogens. Additionally, these probes are able to disrupt biofilm formation in *S. aureus* SH1000, thereby further proving the cell surface interactions with *S. aureus*

Bacterial adhesion to host cells is a crucial step in the infection process. Bacteria exploit interactions with specific carbohydrates via lectins (glycan-binding proteins), which recognize specific cell surface glycans on host cells and tissue surfaces, as part of the processes of colonization and infection.^1−4^ In the same manner that bacteria may exploit glycan mimics to hijack host biology to establish infection,^5^ it is possible to develop glycan-based tools as antagonists of such interactions for use as antiadhesive agents^6−9^ and diagnostic tools.^10,11^

To design probes to exploit bacterial interactions with carbohydrates for detection, structurally defined oligosaccharides as well as probes able to replicate physiological multivalent carbohydrate presentation and so overcome the often low binding affinities of monovalent glycosides are needed. Another important consideration is that glycan receptors are found in both human cells and pathogens and in some instances share similar specificities for the same carbohydrate moieties.^12^ For instance, many strains of uropathogenic *Escherichia coli* (UPEC) type 1 encode the virulence factor FimH, which is a mannose-specific lectin,^13^ while the DC-SIGN lectin also binds specifically to mannose (and fucose) residues.^14^ Therefore, the identification of glycan(s) and scaffold type, size, and degree of multivalency that allow for specific interactions with bacteria but do not interfere with the host or other species is the key to successful downstream applications.

*Staphylococcus aureus* (*S. aureus*) is a Gram-positive bacterium commonly found in the natural environment and as a human commensal and one of the so-called “ESKAPE” human pathogens,^15,16^ which has been linked to life-threatening diseases such as pneumonia, meningitis, septicaemia, and endocarditis.^17,18^ Moreover, under favorable conditions, *S. aureus* can produce enterotoxins, one of the most common causes of food-borne diseases.^19^ Due to the prevalence of *S. aureus* infections in healthcare settings and the rise of antimicrobial resistance, there is a pressing need to develop fast and accurate point-of-care diagnostic tools.^17,20−25^ However, most methods for *S. aureus* detection in clinical settings are time-consuming and costly, being based on bacterial culture, nucleic acid amplification, or antibody-based immune- or immuno-PCR assays.^17^

Rapid diagnostic strategies to detect and identify bacteria are vital to maintain effective antibiotic stewardship and avoid the over prescription of broad-spectrum agents. To that end, our team recently disclosed a rapid diagnostic agglutination strategy based on mannose-functionalized polymeric microbeads in combination with computer-aided cluster analysis for the detection and identification of laboratory, clinical, and UPEC strains.^26^ We thus proposed that such a strategy could be adapted for the fast screening of other bacterial species, employing glycans not found in mammalian systems with the aim of identifying a unique pathogen-specific glycan fingerprint.

d-Arabinofuranose (Ara*f*) is a rare sugar not found in humans but an essential component of the bacterial cell walls of the Mycobacteria^27,28^ and also found in Pseudomonads.^29^ The 2- and 5-azido-(*Z*,*Z*)-farnesyl phosphoryl-β-d-arabinofuranose analogues were shown to be useful probes for the preferential metabolic labeling of *Mycobacterium smegmatis* and *Corynebacterium glutamicum*, respectively.^28^ Moreover, l- and l-2-deoxy-2-[^18^F]fluoro-arabinofuranose analogues developed for positron emission tomography (PET) analysis of bacteria showed greater accumulation in *E. coli* than the corresponding 5-deoxy-5-[^18^F]fluoro-arabinofuranose.^30^

We thus speculated that Ara*f* represents a promising target for investigation with our agglutination strategy and that suitable modifications of key polar groups at different positions around the arabinose moiety might lead to glycan presentations that could enhance selectivity toward specific bacterial species, potentially aiding the discovery of novel sugar motifs that could eventually be applied in the development of bacterial diagnostics.

## Results and Discussion

**Figure 1:**
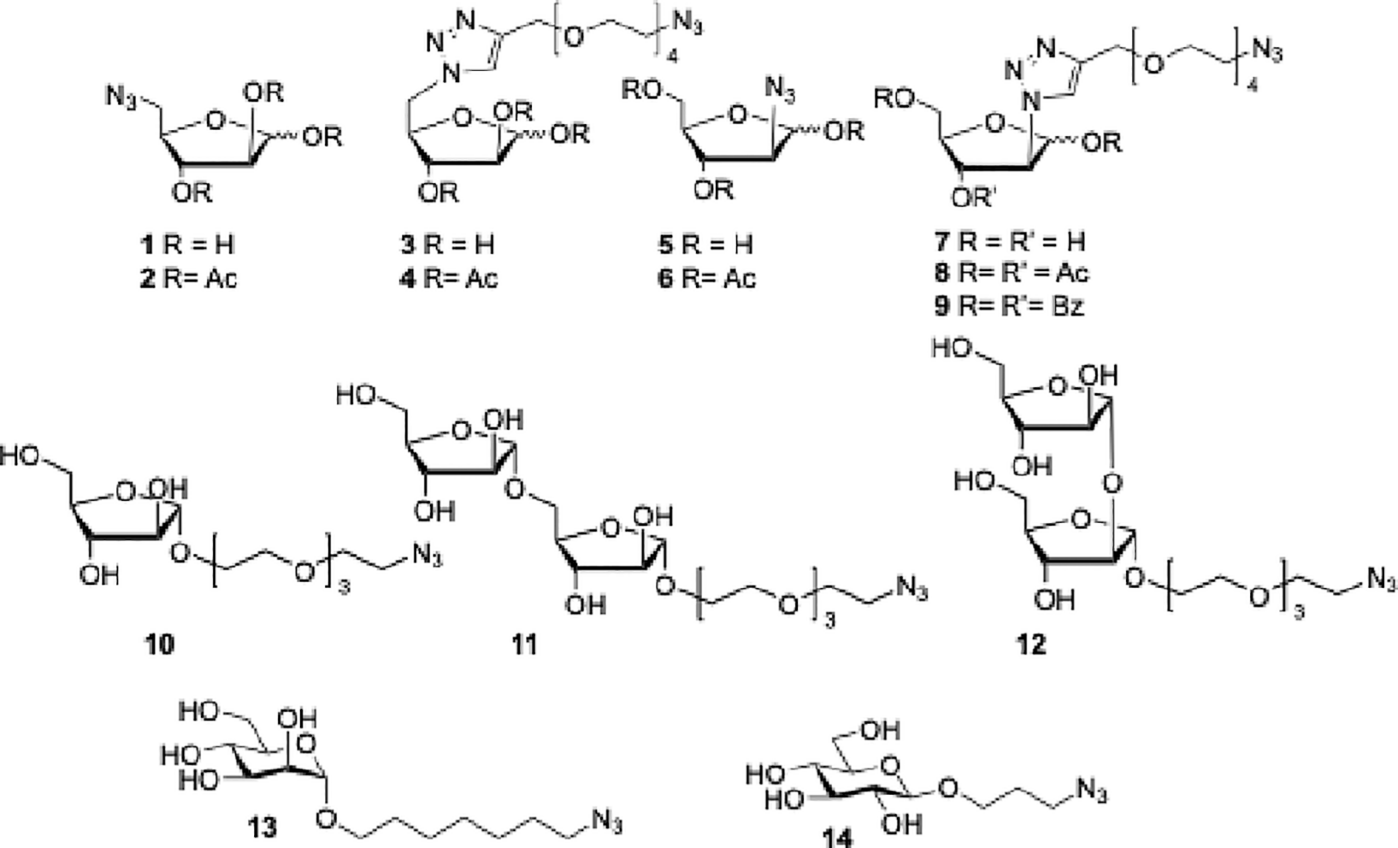
Library of Ara*f* analogues **1**–**12**, mannoside **13**, and glucoside **14**.

To that end, a library of Ara*f* derivatives (**1**–**12**) was prepared (Figure 1). Compounds **1**–**9**, including ester-protected examples, as in **2**, **4** and **6**, **8**, and **9**, were functionalized with an azido group at either the C2 or C5 positions for direct attachment to microbeads or a tetraethylene glycol (PEG)_4_-N_3_ linker to evaluate the importance of regio-substitution for the labeling of bacteria and also the importance of the spacer length between the probe and the sugar unit. In addition, the C1-modified Ara*f***10** and the corresponding α-1,5 and α-1,2-disaccharides (**11** and **12**) analogues featuring a PEG_3_-N_3_ linker were also prepared. Additionally, α-linked mannoside **13**(26) and β-linked glucoside **14**(31) were also prepared as controls.

**Scheme 1:**
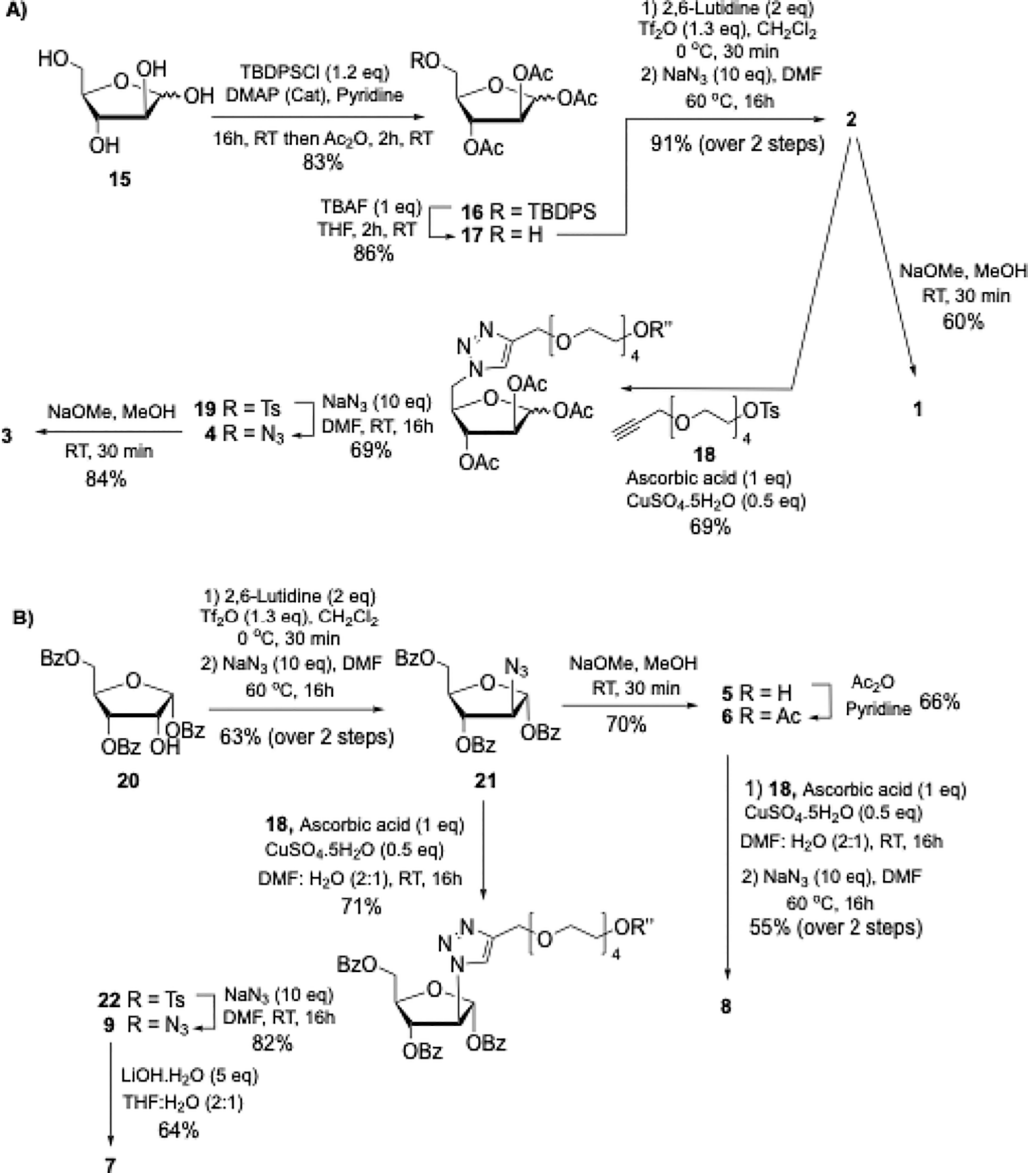
(A, B) Synthesis of 5- and 2-Azido-Linked Arabinose Glycosides **1**–**9**

In brief, C5-functionalized **3** and **4** can be accessed from d-arabinose **15** (Scheme 1A). The ^*t*-^butyldiphenylsilyl protection of 5-OH in **15** using TBDPSCl and DMAP followed by *in situ* acetylation in the presence of acetic anhydride and pyridine afforded fully protected arabinose intermediate **16** in 83% yield. Subsequent cleavage of silyl ether using TBAF gave the 5-OH derivative **17** in 86% yield, which following triflation and S_N_2 displacement with sodium azide afforded the 5-azido compound **2** in 91% yield over the two steps. The Cu(I)-catalyzed azide–alkyne cycloaddition (CuAAC) of **2** with monotosylated tetraethylene glycol (TsO-(PEG)_4_-alkyne) linker **18** followed by NaN_3_ displacement afforded the desired compound **4** with a (PEG)_4_-N_3_ spacer (48% overall yield). Finally, Zemplén’s deacetylation of compounds **2** and **4** afforded the unprotected derivatives **1** and **3** in 60 and 85% yield, respectively.

On the other hand, C2-substituted analogues **5**–**9** were prepared from commercially available 1,3,5-tri-*O*-benzoyl-α-d-ribofuranose **20** (Scheme 1B). Inversion of configuration at 2-OH via triflation with triflic anhydride followed by the S_N_2 displacement with sodium azide afforded the 2-azido arabinose intermediate **21** in 63% yield, which could be converted to compound **5** upon ester deprotection using NaOMe in MeOH in 70% yield, or to the acetylated analogue **6** after reaction with acetic anhydride and pyridine in 66% yield. Conjugation of the alkynyl-linker **18** with the azido compounds **6** or **21** under CuAAC conditions followed by azide displacement and saponification gave (PEG)_4_-N_3_-functionalized derivatives **8** and **9** in 55 and 58% yields, respectively. The latter was then converted to the unprotected analogue **7** upon ester deprotection with LiOH·H_2_O in 64% yield.

**Scheme 2:**
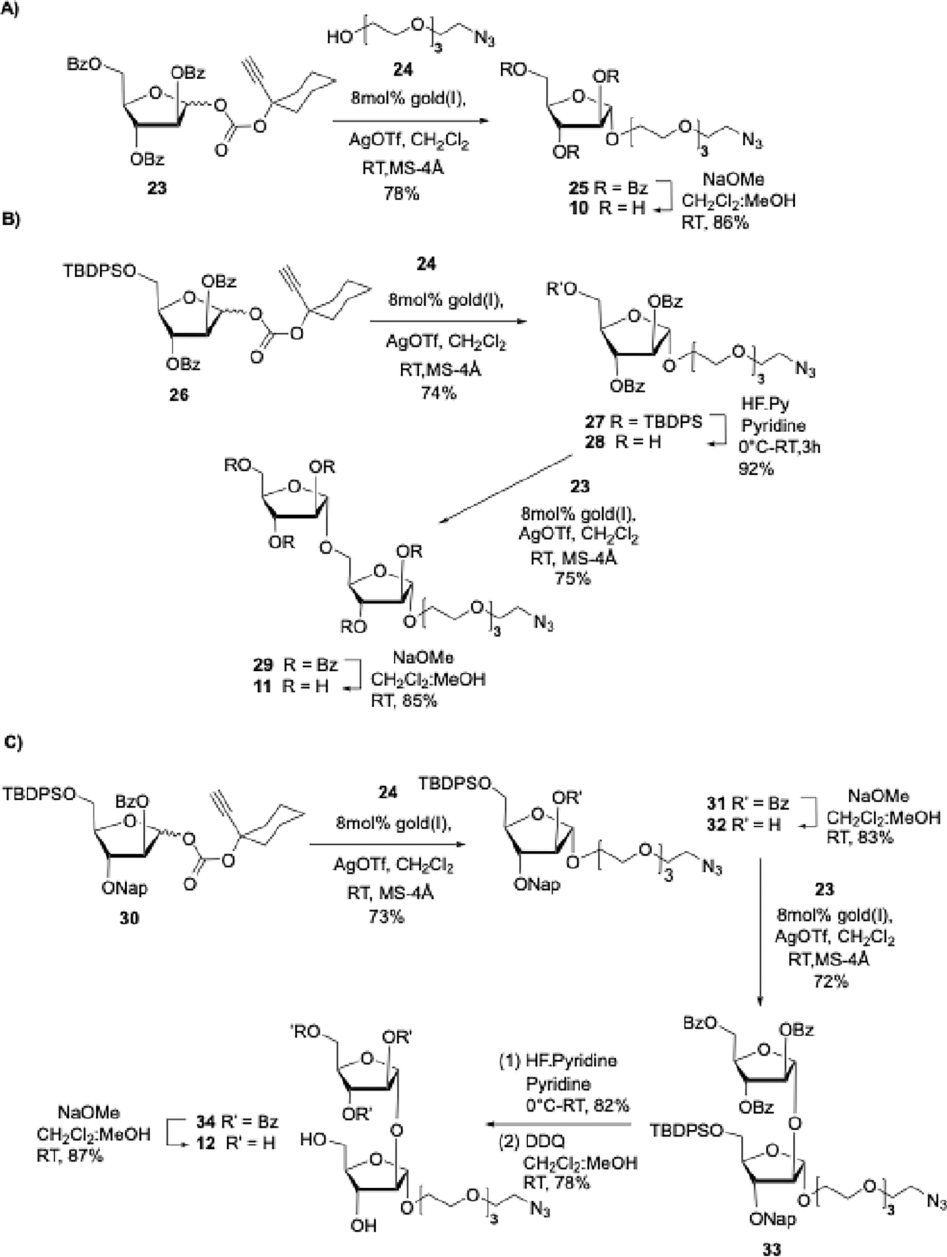
(A–C) Synthesis of 1-Azido-Linked Arabinose Glycosides **10**–**12**

An alternative strategy was required to access C1-modified analogues **10**–**12**. To achieve the desired 1,2-*trans* or α-linkages, a slightly modified glycosylation protocol^32^ utilizing alkynyl carbonate glycosyl donors in combination with neighboring group participation under gold–silver catalyzed glycosidation conditions was envisioned.^32^ The reaction of perbenzoylated alkynyl carbonate glycosyl donor **23**(33) with 1-azido tetraethylenene glycol **24** in the presence of 8 mol % each of AgOTf and gold-phosphite catalyst in CH_2_Cl_2_ afforded glycoside **25** in 78% yield, which upon saponification with NaOMe in a mixture of MeOH/CH_2_Cl_2_ afforded **10** in 86% yield (Scheme 2A). The synthesis of α-1,5-disaccharide **11** started with glycosylation of orthogonally protected donor **26**(33) with aglycone **24** under the aforementioned conditions to afford glycoside **31** in 73% yield. Further, selective removal of the silyl ether moiety with HF·Py afforded arabinofuranoside **28** in 68% overall yield that is ready for the next glycosylation with alkynyl carbonate **23** to give disaccharide **29** in 75% yield as a single diastereomer. Subsequent global deprotection of all esters under basic conditions afforded desired disaccharide **11** in 85% yield (Scheme 2B). Similarly, α-1,2 disaccharide **12** was obtained from glycosylation of differentially protected arabinofuranoside **30**,^34^ featuring a base-labile −OBz group at C-2, with aglycon **24** under the Au(I)/Ag catalytic conditions to give the arabinofuranoside **31** in 73% yield. Selective C-2 deprotection of esters under Zemplen conditions (NaOMe/MeOH) afforded glycosyl acceptor **32** in 61% overall yield, which upon glycosylation with donor **23** gave disaccharide **33** in 72% yield. Last, global deprotection entailed cleavage of the silyl ether using HF·Py as before; naphthyl ether cleavage using DDQ and benzoyl ester deprotection using NaOMe in a mixture of CH_2_Cl_2_/MeOH gave the desired disaccharide **12** in 56% yield over three steps (Scheme 2C).

The azido-terminated glycans **1**–**14** were then conjugated to alkyne-functionalized 10 μm polystyrene microbeads **MB-Alkyne**, which were prepared as previously described from commercially available carboxylic acid-coated microbeads (**MB-CO**_**2**_**H**)^26^ via CuAAC^35^ to generate probes **MB-1** to **MB-14**. As an additional control, azido-tetraethylene glycol moiety **24** was also conjugated to the microbeads (**MB-24**) to investigate the importance of the multivalent presentation of glycans to bacterial interaction as opposed to just the presence of −OH groups. The conjugations were performed using a large excess of reagents (400 equiv of azido-derivatives, 11K equiv of sodium ascorbate and 2.1K equiv of CuSO_4_·5H_2_O) to ensure complete surface functionalization, which was monitored by a coumarin-based fluorescence test^36^ (see the SI for full experimental details (Figure S1)).

**Scheme 3:**
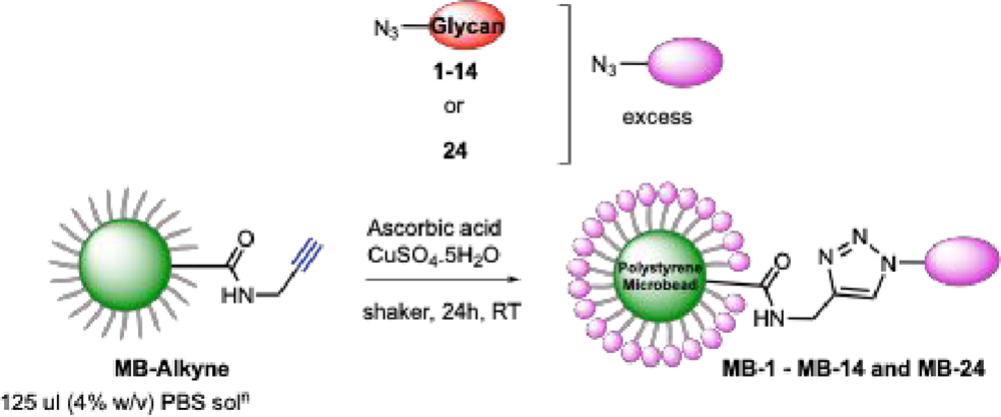
General Microbead Glycan Conjugation Strategy

Next, the ability of the resulting functionalized microspheres to agglutinate *S. aureus* Newman strain was investigated using bright field microscopy and computer-aided cluster analysis.^26^ Naked **MB-CO**_**2**_**H** and functionalized microbeads **MB-1** to **MB-14**, **MB-24**, and **MB-Alkyne** (10 μL of microbeads at 0.6% w/v) were incubated for 1 h at 21 °C with 20 μL of bacterial suspension that had been grown overnight in Luria–Bertani (LB) broth (37 °C, 200 rpm shaking) and standardized to a concentration of 10^9^ CFU/mL in PBS. The results were compared to **MB-Alkyne** in PBS-only, as a control. It was previously established that the microbead sample concentration was critical, with an average of 350–400 beads per image found to be optimal for accurate cluster measurements.^26^ The bacteria/microbead suspension was deposited onto the glass slides and imaged at 10× magnification in a ZEISS Primo Star iLED microscope.^37^ To ensure reproducibility, each agglutination assay was repeated in triplicate (three biological replicates each with three technical replicates), and five images were taken for each sample. To ensure that no residual copper from the conjugation reaction (Scheme 3) could affect the bacterial agglutination assay,^38^**MB-Alkyne** was resubjected to the same coupling conditions (CuSO_4_, Na ascorbate, overnight shaking) used to conjugate the azido-Ara*f* derivatives albeit in the absence of the azido partner and then washed using the usual procedure. These microbeads were then incubated with *S. aureus* as above and showed negligible agglutination compared to the untreated **MB-Alkyne** only control (Figure S4), confirming that any observed agglutination can be attributed to the glycan-functionalized probes.

**Figure 2:**
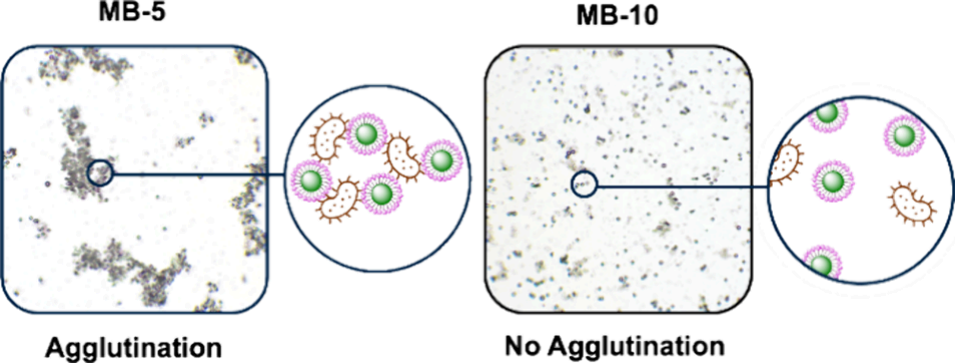
Representative images (10× magnification) of positive and negative agglutination events with *S. aureus* Newman (10^9^ CFU/mL) upon incubation with **MB-5** and **MB-10**.

To identify clusters and distinguish them from single beads, the Python-based software Abacus^26^ was used to determine the number of agglutinated beads contained in all the visible clusters. In this manner, the cluster to beads ratio (CBR) was calculated to parametrize the agglutination events observed and the extent of agglutination.^39^Figure 2 and Figures S3 and S4 (SI) show the typical images of a positive and negative agglutination event.

**Figure 3:**
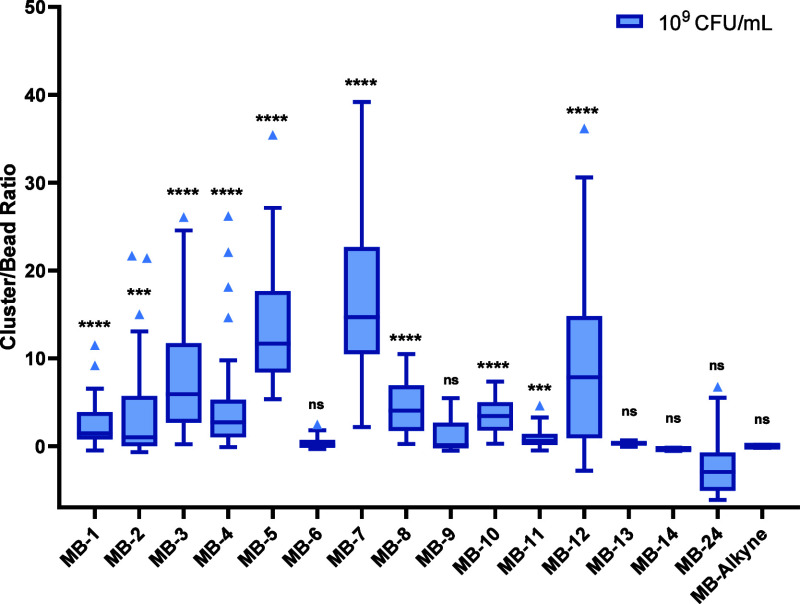
CBR for **MB-1** to **MB-14** and **MB-24** upon a 1 h incubation with *S. aureus* (10^9^ CFU/mL in PBS) at 21 °C. The statistical significance was assessed with the Mann–Whitney U test, comparing the sample population data against the control population. (*p* < 0.05 = *, *p* < 0.01 = **, *p* < 0.001 = ***, *p* < 0.0001 = ****).

**Figure 4:**
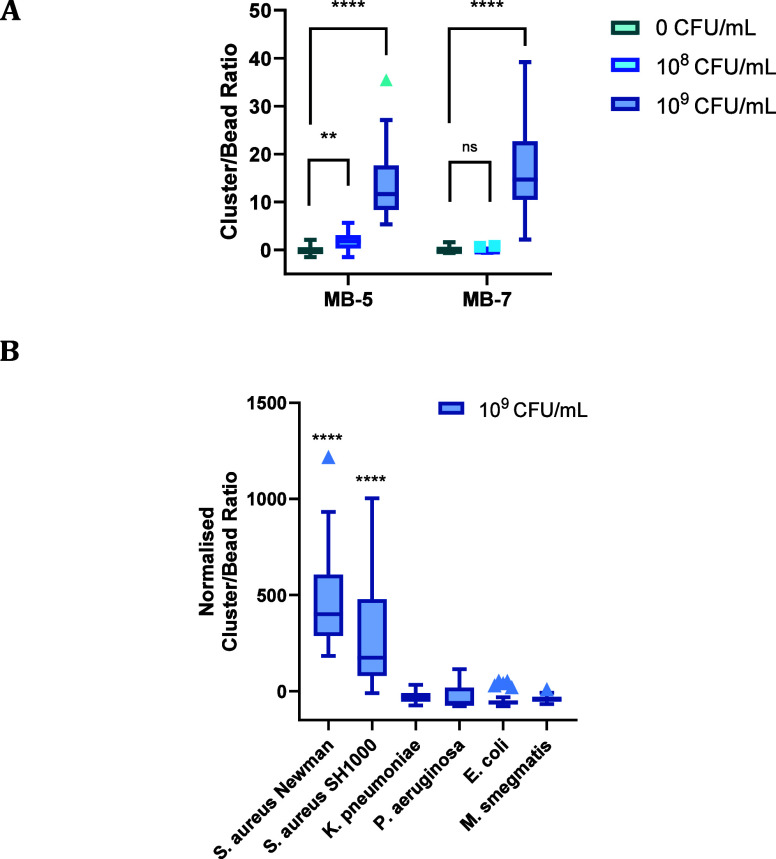
(A) CBR for **MB-5** and **MB-7** with *S. aureus* Newman at 10^8^ and 10^9^ CFU/mL. (B) CBR ratio for **MB-5** after a 1 h incubation at 21 °C with *S. aureus* Newman and SH1000, Gram-positive *M. smegmatis* mc(2)155, and Gram-negative *E. coli* BW25113, *P. aeruginosa* PA01, and *K. pneumoniae* NCTC 5055 at 10^9^ CFU/mL in PBS and 21 °C. Statistical significance assessed with the Mann–Whitney U test, comparing sample data populations against the agglutination of the microbead probe in PBS as a control (*p* < 0.0001 = ****).

Interestingly, the highest levels of agglutination were observed for the C2-linked arabinose polystyrene beads **MB-5** and **MB-7** (Figures 3 and 4A) at concentrations down to 10^8^ CFU/mL (for **MB-5**), whereas lower levels of clustering were detected for any of the other functionalized probes when compared to controls (functionalized microbeads incubated in PBS in the absence of bacteria, acid-coated microbeads **MB-CO**_**2**_**H** (data not shown), or unconjugated alkyne-microbeads **MB-Alkyne** incubated in the presence of bacteria). These results suggest that both the specific glycan and its presentation (e.g., C1- vs C2- vs C5- conjugation) are crucial for the interaction. Moreover, the presence of the hemiacetal, which exists in equilibrium with the corresponding open aldehyde form^40^ is also important, since C2- or C5-linked disaccharide probes conjugated to the microbead via the C-1 position at the reducing end (e.g., **MB-11** and **MB-12**) elicited very low agglutination compared to **MB-5** or **MB-7** (Figure 3 and Figure S5). Moreover, it is notable that while C2- and C5-linked arabinoside derivatives (**MB-1**, **MB-3**, **MB-5**, and **MB-7**) all feature a hemiacetal motif, the C2-substituted probes (**MB-5** and **MB-7**) are in equilibria between the linear and both the furanose and pyranose forms and the C5-counterparts (**MB-1** and **MB-3**) cannot access the pyranose conformations,^40^ which might potentially contribute to the observed agglutination differences. Additionally, mannose, glucose, or tetraethylene glycol-functionalized beads (**MB-13**, **MB-14**, or **MB-24**) showed no changes in agglutination when incubated with *S. aureus* when compared to controls, further suggesting that the 2-arabinofuranose motif is crucial to the observed interaction.

The selectivity of the C2-linked arabinose probes **MB-5** toward other bacterial species, namely, the Gram-positive organisms *M. smegmatis* mc (2)155 and Gram-negative species *E. coli* BW25113, *P. aeruginosa* PA01, and *K. pneumoniae* NCTC 5055, was then investigated. To that end, the functionalized probes were incubated with 20 μL of bacteria (10^9^ CFU/mL in PBS) and images were collated and processed as before (Figure 4B and Figure S6). No substantial agglutination, compared to controls, was observed for any of these species, suggesting the probes to be selective for *S. aureus*.

To evaluate whether the agglutination observed with our C2-linked probes involved metabolic pathways for Ara*f* bacterial uptake,^41^ C2-linked microbeads **MB-5** were incubated in the presence of *Araf* at a range of concentrations up to 10K-fold excess with respect to conjugated Ara*f* on the probe. No statistically significant difference in agglutination was observed (Figure S7), suggesting a different interaction mechanism with the bacterial surface. Moreover, we also showed that multivalency and degree of substitution on the microbead were important parameters since probes that had a lower degree of substitution (20 equiv vs 400 equiv) showed diminished binding (Figure S8).

Bacterial biofilms, formed on surfaces, including living tissues, by the accumulation of microorganisms within a supporting matrix, are a leading cause of persistent and chronic infections.^42^ The complex biofilm matrix is composed of extracellular polymeric substances and acts as a mechanical barrier against both antibacterial drugs and components of the host immune response. Moreover, enzymes present in the extracellular biofilm matrix can modulate drug absorption. Generally, bacterial biofilms are hard to eradicate and enable emergence of drug resistance considerably faster than in the corresponding planktonic bacteria.^43^ Previous studies have found that multivalent carbohydrate-based ligands can intercept and interfere with glycan recognition pathways in bacteria, leading to biofilm inhibition and dispersal.^44^

**Figure 5:**
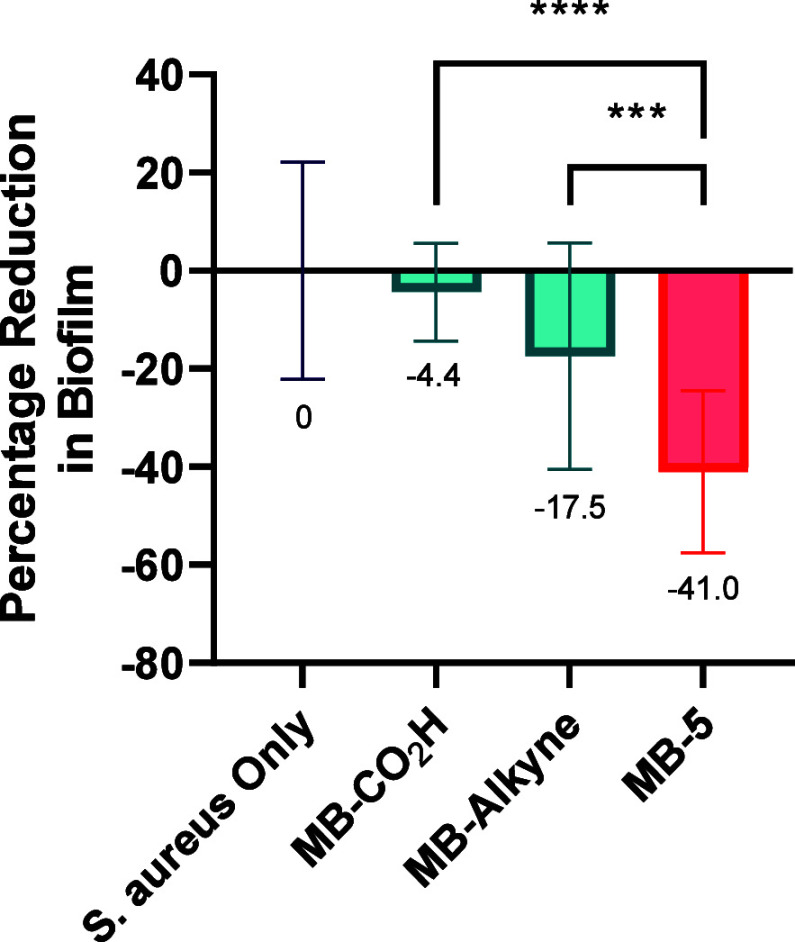
*S. aureus* SH1000 biofilm disruption assay. Established biofilms treated with 6% w/v of **MB-5**, **MB-CO**_**2**_**H**, or **MB-Alkyne** and compared to the untreated biofilm as a control. Statistical significance assessed with the Mann–Whitney U test. (*p* < 0.05 = *, *p* < 0.01 = **, *p* < 0.001 = ***, *p* < 0.0001 = ****).

Encouraged by these reports and the ability of our C2-linked Ara*f* probes to interact with *S. aureus*, we then decided to evaluate the ability of these probes to disrupt *S. aureus* SH1000 biofilms. This strain was chosen for its ability to generate consistent biofilms.^45^ Ara*f* microbeads **MB-5** (6% w/v) were added to a 96-well plate containing preformed bacterial biofilms, the plate was incubated for a further 24 h, and biofilm accumulation was compared to that of biofilms incubated with the same concentration of propargylated microbeads **MB-Alkyne** or PBS buffer controls, using crystal violet staining (see full experimental details in the SI). While incubation with **MB-Alkyne** led to a 17.5% reduction in biofilm accumulation, likely due to the abrasive effect of the polystyrene particles,^30^ a 41% decrease compared to untreated controls was observed upon exposure to **MB-5**. (Figure 5) Moreover, biofilm incubation with free d-arabinose, l-arabinose, or 2-deoxy-2-azido-d-arabinose in LB-broth (20 μM to 2 mM) did not show any biofilm disruption (Figure S10), suggesting that preferential interaction of *S. aureus* SH1000 with the conjugated glycan beads encourages detachment of bacterial cells from the biofilm.

## Conclusions

In summary, we have synthesized a library of nonmammalian d-arabinofuranose (Ara*f*) probes that were conjugated, at the C-1, C-2, or C-5 positions, to polystyrene microbeads to explore and exploit sugar/receptor interactions at the bacterial cell surface. We established a practical screening platform, based on bacterial agglutination in conjunction with computer-aided cluster analysis,^13^ to evaluate the ability of the probes to interact with *S. aureus*. We found preferential and selective binding of C2-linked Ara*f* probes (e.g., **MB-5**) toward *S. aureus* (Newman and SH1000) in contrast to comparator Gram-positive (*M. smegmatis* mc(2)155) and Gram-negative (*E. coli* BW25113, *P. aeruginosa* PA01, and *K. pneumoniae* NCTC 5055) species, which showed no significant binding. Notably, we further demonstrate the ability of **MB-5** to interact with the bacterial cell surface by showing disruption of established *S. aureus* SH1000 biofilms by up to 23%, compared to alkyne-functionalized **MB-Alkyne**. This study identifies multivalent C2-linked Ara*f* derivatives as promising candidates for detection and/or antiadhesion strategies for *S. aureus* and reveals some key structural features (e.g., glycan type and spatial presentation) needed for recognition of the cell surface while also highlighting the utility of glycans, beyond those found in the host, in strategies exploiting interactions with bacteria.

## Supporting Information Available

Synthetic protocols and characterization data for all compounds, including all bacteria screening, biofilm studies, and NMR spectra (PDF)
