# Supplementary material for: C2-Linked Arabinose-Functionalized Polystyrene Microbeads Selectively Target Staphylococcus aureus
Source: JACS Au. 2024 Nov 8;4(11):4537–43. doi: 10.1021/jacsau.4c00931 (PMC11600162; doi:10.1021/jacsau.4c00931)

Supporting Information

C2-linked Arabinose Functionalized Polystyrene Microbeads Selectively Target *Staphylococcus Aureus*

Gulab Walke [a‡], Cristina Santi [a‡], Calum Haydon [a], Pooja Joshi [c], Yuiko Takebayashi [b], Sylvain Rama[d], Josephine Dorh [d], Srinivas Hotha [c*], James Spencer [b*], M. Carmen Galan [a*]

[a]School of Chemistry, University of Bristol, Cantock's Close, Bristol, BS8 1TS, United Kingdom. [b]School of Cellular and Molecular Medicine, University of Bristol Biomedical Sciences Building, Bristol, BS8 1TD, United Kingdom. [c]Department of Chemistry, Indian Institute of Science Education and Research Pune, Pune 411 008, India. [d]FluoretiQ Ltd, Futurespace, Filton Road, Stoke Gifford, Bristol, BS34 8RB, United Kingdom.

‡equal contribution

*Email: s.hotha@iiserpune.ac.in; jim.spencer@bristol.ac.uk and m.c.galan@bristol.ac.uk

KEYWORDS. Antimicrobial probes • Oligosaccharide synthesis • non-mamalian glycans • bacteria targeting • agglutination

Index:

1) **Materials General Experimental Details**

2) **Synthetic Schemes**

3) **Experimental Procedure and Analytical Data**

4) **Arabinose Sugar-Microbead Conjugation and Analysis**

5) **Microbead-Based Bacterial Assays**

   A] Microbead-Based Agglutination Assay

   B] Biofilm Disruption Assay

6) **References**

7) **NMR Spectra**

General Experimental Details:

D-(-)-Arabinose 99%, Tert-butyl(chloro)diphenyl silane, Tetrabutylammonium fluoride solution (1 M THF), Trifluorosulfonic anhydride, 2,6-Lutidine, Sodium methoxide (25% in methanol), Lithium hydroxide monohydrate, Sodium azide and Amberlite® IR-120 H^+ form, were purchased from Sigma Aldrich. Tetraethylene glycol was purchased from Acros Organics. 1,3,5-Tri-*O*-benzoyl-α-D-ribofuranose was purchased from US Biological Life Sciences. Carboxylate-modified latex (CML) beads 4% w/v in H_2O (10 µm), 1-ethyl-3-(3-dimethylaminopropyl) carbodiimide hydrochloride (EDC), propargylamine, copper sulphate, and L-ascorbic acid (and its sodium salt) were purchased from Fisher Scientific.

E. coli BW25113 was purchased from the *E. coli* Genetic Stock Center (Yale CSC, New Haven, United States). *K. pneumoniae* NCTC 5055, *S. aureus* Newman and S. *aureus* SH1000 were gifts from Matthew B Avison and Angela Nobbs respectively (University of Bristol). *P. aeruginosa* PA01 was obtained from the *Pseudomonas* Genetic Stock Centre (East Carolina University, United States). *M. smegmatis* mc^2155 was purchased from atcc.org. Phosphate buffered saline (PBS), sterile inoculation loops were purchased from Fisher Scientific Ltd, and microscope slides were purchased from AmScope.

Chemical reactions were monitored by TLC on Kieselgel 60 F254 (Merck) and examined under UV light (254nm) or by staining with 5% sulfuric acid in ethanol (sugar dip) or $KMnO_4$. Flash column chromatography was performed using silica gel - Merck, 230−400 mesh (40−63 µm). NMR spectra were recorded in the appropriate deuterated solvent (purchased from Sigma Aldrich) at 400 MHz, 500 MHz on a Brucker spectroscope. The chemical shifts were referenced to the residual solvent peak and reported in ppm. Mass spectrometry was carried out by the University of Bristol Mass

Spectrometry Service on a micrOTOF II (ESI) spectrometer, with the HRMS mode incorporating a lock-in mass injected midway through the run (sodium formate).

All bacteria suspensions were centrifuged on a Hermle Z 207 M centrifuge and their OD600 was measured with Ultrospec 2100 pro (Amersham Biosciences) UV/Visible Spectrophotometer. Bacteria/microbeads suspensions were shaken on a ThermoMixer C (Eppendorf) shaker. Agglutination images were captured with a ZEISS Primo Star iLED microscope. Crystal violet fluorescence was measured on a CLARIOstar (BMG Labtech) plate reader, using Costar 96-well, flat bottom plates.

Synthetic Schemes:

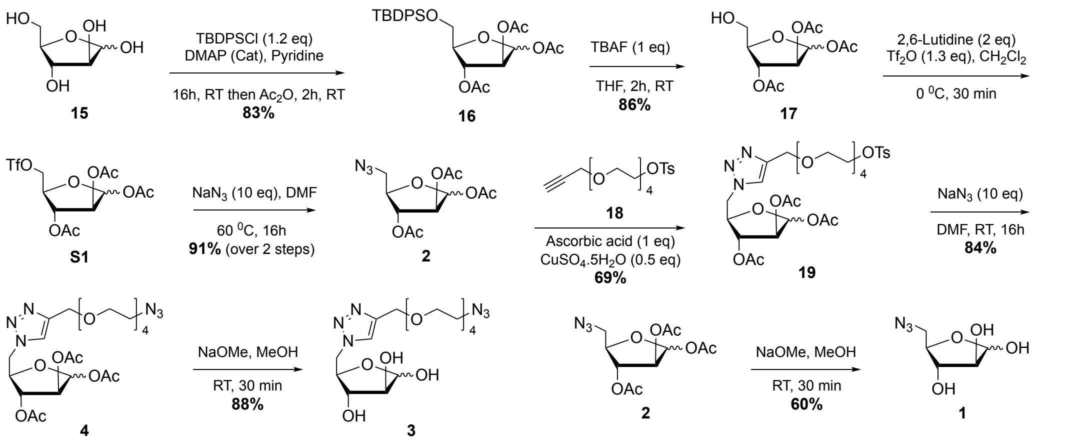

Scheme S1: Synthesis of 5-Azido-Arabinose Derivatives

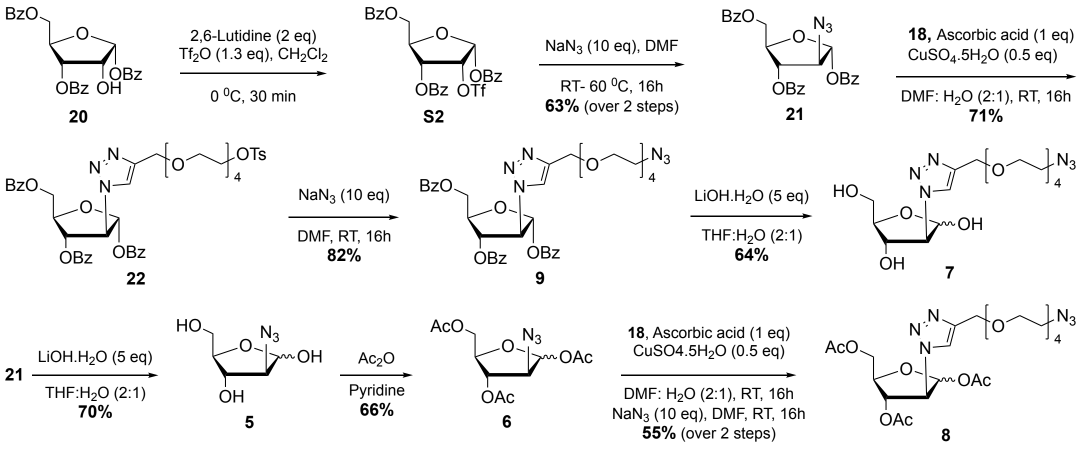

Scheme S2: Synthesis of 2-Azido-Arabinose Derivatives

A)

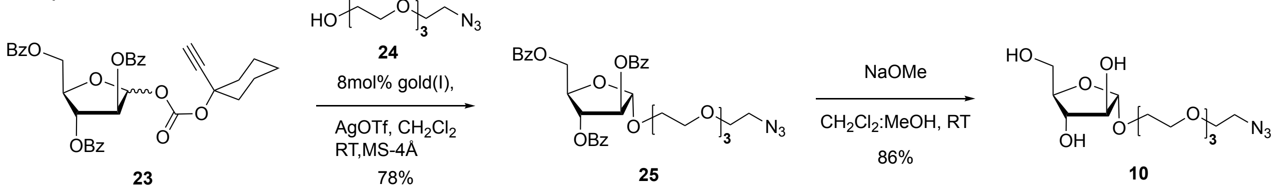

BzO
OBz
OBz
23
HO
O
3
N3
24
8mol% gold(I),
AgOTf, CH2Cl2
RT,MS-4Å
78%
25
NaOMe
CH2Cl2:MeOH, RT
86%
HO
OH
10

B)

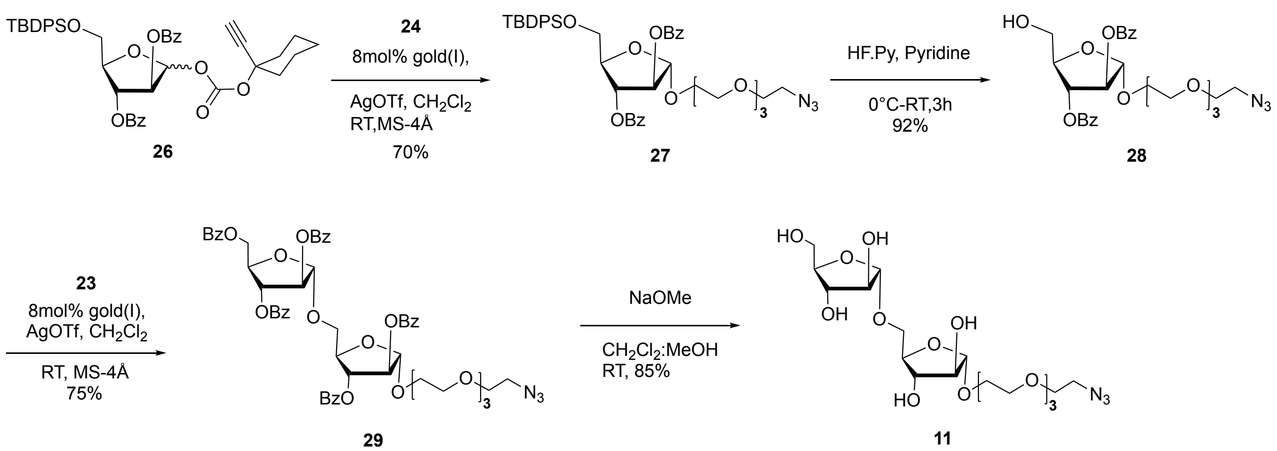

TBDPSO
OBz
26
24
8mol% gold(I),
AgOTf, CH2Cl2
RT,MS-4Å
70%
27
HF.Py, Pyridine
0°C-RT,3h
92%
28
23
8mol% gold(I),
AgOTf, CH2Cl2
RT, MS-4Å
75%
29
NaOMe
CH2Cl2:MeOH
RT, 85%
11

C)

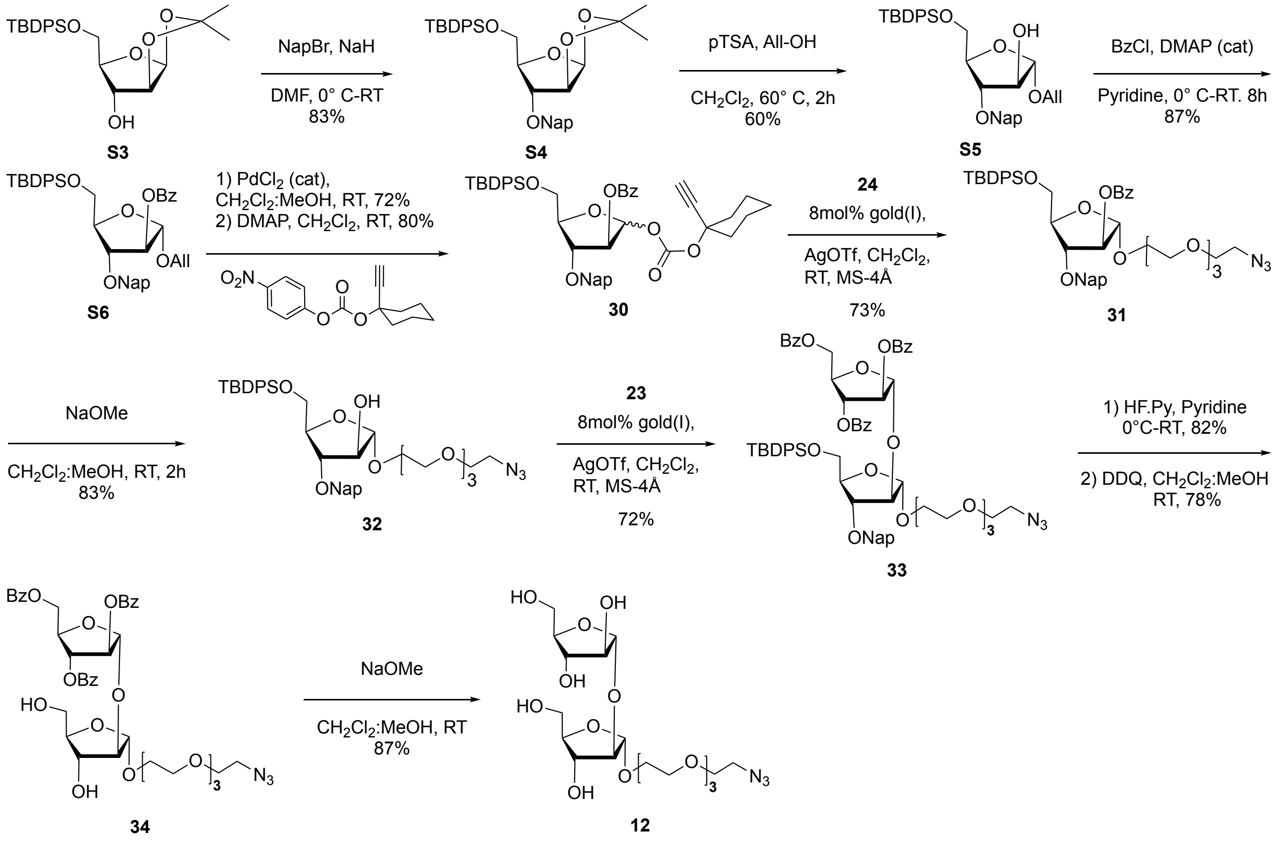

TBDPSO
OH
S3
NapBr, NaH
DMF, 0° C-RT
83%
ONap
S4
pTSA, All-OH
CH2Cl2, 60° C, 2h
60%
OAll
S5
BzCl, DMAP (cat)
Pyridine, 0° C-RT. 8h
87%
S6
1) PdCl2 (cat),
CH2Cl2:MeOH, RT, 72%
2) DMAP, CH2Cl2, RT, 80%
O2N
30
24
8mol% gold(I),
AgOTf, CH2Cl2,
RT, MS-4Å
73%
31
NaOMe
CH2Cl2:MeOH, RT, 2h
83%
32
23
8mol% gold(I),
AgOTf, CH2Cl2,
RT, MS-4Å
72%
33
1) HF.Py, Pyridine
0°C-RT, 82%
2) DDQ, CH2Cl2:MeOH
RT, 78%
34
NaOMe
CH2Cl2:MeOH, RT
87%
12

Scheme S3: Synthesis of 1-Azido-TEG Arabinofuranose Derivatives

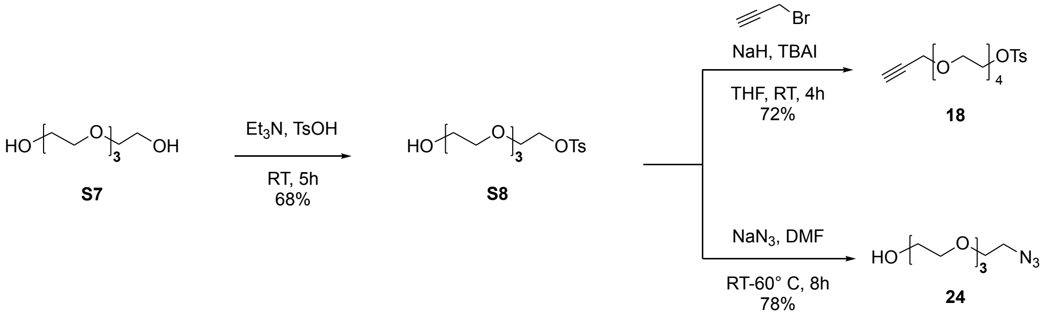

Scheme 4: Decoration of Tetra ethylene Glycol Linker

Experimental Procedure and Analytical Data:

Compound 16: To a stirred solution of D-arabinose **15** (5 g, 33.30 mmol) in pyridine (30 mL) at 0°C, TBDPSCl (8.64 mL, 33.30 mmol) was added, followed by catalytic DMAP under inert conditions. The reaction was allowed to stir at room temperature (RT) for 16h. Then, acetic anhydride (12.59 mL, 133.22 mmol) was added at 0°C and stirred for additional 2h at RT. After completion, the reaction mixture was diluted with ethyl acetate, washed successively with 2M aqueous HCl, saturated $NaHCO_3$ and brine. The organic layer was dried over $MgSO_4$, filtered, and concentrated under reduced pressure. The crude was purified by silica gel column chromatography, yielding compound **16** (14.2 g, 83% yield) as a fluffy, white solid. IR (cm^{-1}, $CHCl_3$): 2953, 2927, 2856, 1747, 1428, 1370, 1210, 1111, 1063, 103; ^1H NMR (400.34 MHz, $CDCl_3$) δ 7.71 – 7.66 (m, 8H), 7.46 – 7.35 (m, 12H), 6.36 (d, J = 4.7 Hz, 1H), 6.20 (s, 1H), 5.63 (dd, J = 7.2, 6.0 Hz, 1H), 5.38 (ddd, J = 4.9, 1.7, 0.7 Hz, 1H), 5.35 (dd, J = 7.2, 4.7 Hz, 1H), 5.21 (dd, J = 1.7, 0.6 Hz, 1H), 4.28 – 4.23 (m, 1H), 4.15 – 4.10 (m, 1H), 3.92 – 3.84 (m, 3H), 3.83 – 3.77 (m, 1H), 2.11 (s, 3H), 2.11 (s, 3H), 2.07 (s, 3H), 2.06 (s, 3H), 2.04 (s, 3H), 1.89 (s, 3H), 1.07 (s, 18H); ^{13}C NMR (100.68 MHz, $CDCl_3$) δ 170.1, 170.0, 169.8, 169.6, 169.5, 135.8, 135.8, 135.7, 133.4, 133.2, 133.2, 133.1, 129.9, 129.9, 127.9, 127.8, 99.7, 93.8, 85.0, 81.8, 81.5, 76.9, 75.8, 74.3, 64.5, 62.9, 26.9, 26.8, 21.2, 21.1, 21.0, 20.9, 20.8, 20.6, 19.5, 19.4; HRMS (ESI-MS): m/z calcd for $C_{27}H_{34}O_8Si$ $[M+Na]^+$: 537.1921, Found: 537.1924.

Compound 17: Tetrabutylammonium fluoride (1.0 M in THF, 15.54 mL) was added dropwise to a solution of compound **16** (8.0 g, 15.54 mmol) in THF (60 mL) at 0˚C under nitrogen atmosphere. The reaction mixture was stirred at room temperature for 2 hours. After completion, the reaction was diluted with ethyl acetate (200 mL), washed with water and brine (2 x 100 mL), dried over $MgSO_4$,

and concentrated in *vacuo*. The crude product was purified by silica gel flash column chromatography using hexane:ethyl acetate (3:7) mixture as an eluent to afford 3.7 g (86% yield) of pure compound **17** as a white solid. IR (cm^{-1}, $CHCl_3$): 3477, 2921, 2851, 1739, 1371, 1216, 1060, 764; ^1H NMR (400.34 MHz, $CDCl_3$) δ 6.17 (d, *J* = 0.4 Hz, 1H), 5.24 (dd, *J* = 1.9, 0.6 Hz, 1H), 5.11 (ddd, *J* = 5.2, 1.9, 0.7 Hz, 1H), 4.22 (dd, *J* = 5.1, 3.7 Hz, 1H), 3.89 (ddd, *J* = 12.3, 5.2, 3.4 Hz, 1H), 3.80 (ddd, *J* = 12.3, 8.2, 4.2 Hz, 1H), 2.13 (s, 3H), 2.12 (s, 3H), 2.11 (s, 3H); ^{13}C NMR (100.68 MHz, $CDCl_3$) δ 170.5, 169.7, 169.6, 99.4, 85.1, 81.1, 76.8, 61.9, 21.2, 20.9, 20.8; HRMS (ESI-MS): m/z calcd for $C_{11}H_{16}O_8$ $[M+Na]^+$: 299.0743, Found: 299.0744.

Compound 2: To a solution of 1,2,3-tri-*O*-acetyl arabinofuranose **17** (3.5 g, 12.67 mmol) in CH_2Cl_2 (50 mL), 2,6-lutidine (2.94 mL, 25.34 mmol) and Tf_2O (3.32 mL, 16.47 mmol) were added at 0°C under nitrogen atmosphere. The reaction was stirred for 30 min at 0°C. Upon completion, it was diluted with CH_2Cl_2 (100 mL) and washed with a mixture of cold water-brine solution (2 x 100 mL). The organic layer was dried over $MgSO_4$, filtered and the solvent was removed under reduced pressure. The crude product **S1** was dissolved in DMF (60 mL), NaN_3 (8.23 g, 126.62 mmol) was added, and the mixture was stirred for 16h at 60 °C. After completion, the reaction mixture was diluted with ethyl acetate (200 mL) and washed with brine (2 x 200 mL). The organic layer was dried over $MgSO_4$, filtered and the solvent was evaporated under vacuum. Following purification through silica gel column chromatography, 3.46 g of 5-azido compound **2** was obtained, constituting a yield of 91% as a sticky solid. IR (cm^{-1}, $CHCl_3$): 2922, 2851, 2101, 1742, 1371, 1212, 1009, 981, 764; ^1H NMR (400.34 MHz, $CDCl_3$) δ 6.21 (s, 1H), 5.21 (dd, *J* = 1.5, 0.5 Hz, 1H), 5.04 (ddd, *J* = 4.6, 1.5, 0.8 Hz, 1H), 4.29 (td, *J* = 4.7, 3.2 Hz, 1H), 3.68 (dd, *J* = 13.3, 3.3 Hz, 1H), 3.45 (dd, *J* = 13.4, 4.8 Hz, 1H), 2.13 (s, 3H), 2.12 (s, 3H), 2.11 (s, 3H); ^{13}C NMR (100.68 MHz, $CDCl_3$) δ 170.5, 170.3, 169.8,

169.7, 169.3, 169.3, 99.3, 93.6, 84.2, 80.9, 80.7, 77.5, 75.2, 74.9, 53.1, 51.4, 21.1, 21.1, 20.8, 20.8, 20.5; HRMS (ESI-MS): m/z calcd for $C_{11}H_{15}N_3O_7$ $[M+Na]^+$: 324.0808, Found: 324.0804.

Compound 19: A solution of 5-azido arabinose **2** (650 mg, 2.16 mmol) in a mixture of DMF and H_2O (2:1, 18 mL) was prepared. To this solution TEG-alkyne **18** (834 mg, 2.16 mmol) and sodium ascorbate (427 mg, 2.16 mmol) were added at room temperature, followed by $CuSO_4{\cdot}5H_2O$ (269 mg, 1.08 mmol). The reaction mixture was stirred overnight. After completion, 100 mL of water was added, and the mixture was extracted with ethyl acetate (3 x 50 mL). The organic layer was washed with brine (2 x 100 mL), dried over $MgSO_4$, and the solvent was evaporated by *vacuo*. The crude product was purified by silica gel column chromatography using an eluent gradient of ethyl acetate: methanol (99: 1 to 96:4). This resulted in the isolation of 1.02 g (69% yield) of compound **19** as a sticky solid. IR (cm^{-1}, $CHCl_3$): 3011, 2922, 2851, 1746, 1275, 1260, 1011, 764; ^1H NMR (400.34 MHz, $CDCl_3$) δ 7.79 – 7.77 (m, 2H), 7.73 (s, 1H), 7.35 – 7.32 (m, 2H), 6.13 (s, 1H), 5.14 (d, *J* = 1.2 Hz, 1H), 4.99 (dt, *J* = 4.5, 1.1 Hz, 1H), 4.67 (s, 2H), 4.48 (td, *J* = 5.1, 3.5 Hz, 1H), 4.15 – 4.13 (m, 2H), 3.72 – 3.64 (m, 8H), 3.62 (s, 4H), 3.57 (s, 4H), 2.44 (s, 3H), 2.14 (s, 3H), 2.10 (s, 3H), 1.99 (s, 3H); ^{13}C NMR (100.68 MHz, $CDCl_3$) δ 170.3, 169.5, 169.2, 144.9, 133.2, 130.0, 128.1, 99.2, 83.4, 80.1, 77.8, 70.9, 70.7, 70.7, 70.7, 70.6, 70.1, 69.4, 68.8, 21.8, 21.1, 20.7, 20.7; HRMS (ESI-MS): m/z calcd for $C_{29}H_{41}N_3O_{14}S$ $[M+Na]^+$: 710.2207, Found: 710.2212.

Compound 4: Compound **19** (1.02 g, 1.49 mmol) was dissolved in DMF (10 mL), and NaN_3 (676 mg, 7 eq) was added at room temperature. The reaction mixture was stirred overnight. After completion water (100 mL) was added, and the mixture was extracted with ethyl acetate (3 x 50 mL). The organic layer was washed with a brine (2 x 100 mL), dried over $MgSO_4$, filtered, and the solvent

was evaporated in *vacuo*. Flash chromatography purification using an ethyl acetate:methanol eluent gradient (99: 1 to 96:4) yielded 860 mg of compound **4** as a sticky solid, in 84% yield. IR (cm^{-1}, $CHCl_3$): 3005, 2989, 2922, 2852, 2099, 1744, 1462, 1372, 1275, 764; ^{1}H NMR (400.34 MHz, $CDCl_3$) δ 7.68 (s, 1H), 6.13 (s, 1H), 5.14 (d, *J* = 1.3 Hz, 1H), 5.00 (dt, *J* = 4.6, 1.1 Hz, 1H), 4.83 – 4.70 (m, 2H), 4.68 – 4.67 (m, 2H), 4.50 – 4.46 (m, 1H), 3.70 – 3.65 (m, 14H), 3.38 (t, *J* = 5.1 Hz, 2H), 2.14 (s, 3H), 2.10 (s, 3H), 1.99 (s, 3H); ^{13}C NMR (100.68 MHz, $CDCl_3$) δ 170.3, 169.5, 169.2, 145.2, 124.7, 99.2, 83.5, 80.1, 77.8, 77.5, 77.4, 77.2, 76.8, 70.8, 70.8, 70.8, 70.8, 70.7, 70.2, 70.0, 64.8, 51.2, 50.8, 21.1, 20.7, 20.6; HRMS (ESI-MS): m/z calcd for $C_{22}H_{34}N_6O_{11}$ $[M+Na]^+$: 581.2183, Found: 581.2179.

Compound 3: In a 10 mL single neck round-bottom flask, compound **4** (100 mg, 0.145 mmol) was dissolved in 2 mL of methanol. Then, 30 µL of 25% sodium methoxide solution in methanol (0.131 mmol) was added. The reaction mixture was stirred at room temperature for 30 min. Subsequently, the reaction was neutralized by Amberlite IR-120 resin, H^+ form and filtered. The solvent was removed under vacuum, resulting in the formation of compound **3** as a sticky solid (72 mg, 88% yield). IR (cm^{-1}, $CHCl_3$): 3390, 3005, 2989, 2922, 2854, 2100, 1461, 1275, 1260, 1091, 764; ^{1}H NMR (400.34 MHz, CD_3OD) ^{1}H NMR (400 MHz, Methanol-*d*$_4$) δ 8.01 (d, *J* = 9.5 Hz, 2H), 5.23 (d, *J* = 4.4 Hz, 1H), 5.12 (d, *J* = 2.3 Hz, 1H), 4.72 (dd, *J* = 14.3, 3.4 Hz, 2H), 4.66 (d, *J* = 2.8 Hz, 4H), 4.62 – 4.55 (m, 2H), 4.32 (td, *J* = 6.8, 3.5 Hz, 1H), 4.06 – 3.99 (m, 2H), 3.95 (dd, *J* = 4.3, 2.3 Hz, 2H), 3.75 (dd, *J* = 6.4, 4.2 Hz, 1H), 3.69 – 3.64 (m, 28H), 3.38 (t, *J* = 4.9 Hz, 4H); ^{13}C NMR (100.68 MHz, CD_3OD) δ 145.92, 145.73, 126.27, 126.14, 103.80, 97.98, 83.67, 82.59, 81.77, 79.23, 78.41, 77.67, 71.62, 71.60, 71.56, 71.53, 71.52, 71.11, 70.74, 70.71, 65.02, 65.00, 64.98, 54.73, 53.19, 51.77; HRMS (ESI-MS): m/z calcd for $C_{16}H_{28}N_6O_8$ $[M+Na]^+$: 455.1866, Found: 455.1860.

Compound 1: In a 10 mL single neck round-bottom flask, compound **2** (100 mg, 0.332 mmol) was dissolved in 2 mL of methanol. Then, 46 µL of a 25% sodium methoxide solution in methanol (0.199 mmol) was added. The reaction mixture was stirred at room temperature for 30 min. Subsequently, the reaction was neutralized by the addition of Amberlite IR-120 resin, H^+ form. After filtration, the solvent was evaporated under vacuum. The crude product was then purified by silica gel flash column chromatography using ethyl acetate as an eluent, leading to the isolation of compound **1** as a pure sticky solid (60 mg, 60% yield). IR (cm^{-1}, $CHCl_3$): 3365, 2989, 2851, 2104, 1275, 1261, 780, 764; ^1H NMR (400.34 MHz, CD_3OD) δ 5.17 (dd, *J* = 24.6, 3.5 Hz, 2H), 4.12 – 3.96 (m, 2H), 3.94 – 3.88 (m, 2H), 3.85 – 3.77 (m, 2H), 3.49 (dd, *J* = 13.2, 3.4 Hz, 1H), 3.42 – 3.32 (m, 3H); ^{13}C NMR (100.68 MHz, CD_3OD) δ 103.52, 97.55, 83.89, 83.16, 82.24, 78.92, 78.46, 77.54, 55.04, 53.35; HRMS (ESI-MS): m/z calcd for $C_{16}H_{28}N_6O_8$ $[M+Na]^+$: 455.1866, Found: 455.1860.

Compound 21: In a 25 mL round-bottom flask, 1,3,5-tri-*O*-benzoyl-α-D-ribofuranose **20** (1.0 g, 2.16 mmol) was dissolved in CH_2Cl_2 (15 mL). To this solution, 2,6-lutidine (0.5 mL, 4.32 mmol) and Tf_2O (0.47 mL, 2.81 mmol) were added at 0°C under an inert atmosphere. The reaction mixture was stirred for 30 minutes. Upon completion, it was diluted with CH_2Cl_2 (100 mL) and washed with the cold water-brine solution (3 x 50 mL). The organic layer was then dried over $MgSO_4$. The solvent was removed under vacuum, yielding crude compound **S2.** This crude product was re-dissolved in DMF (12 mL), NaN_3 (987 mg, 7 eq) was added. The mixture was stirred overnight at 60 °C. After completion, water (100 mL) was added, and the mixture was extracted with ethyl acetate (2 x 50 mL). The organic layer was dried over $MgSO_4$, and the solvent was removed in vacuo. The crude product was then purified by flash column chromatography resulting in the isolation of compound **19**

(660 mg, 63% yield) as a sticky solid. IR (cm^{-1}, $CHCl_3$): 2989, 2922, 2850, 2109, 1724, 1453, 1275, 1261, 780, 708; ^{1}H NMR (400.34 MHz, $CDCl_3$) δ 8.15 – 7.99 (m, 6H), 7.66 – 7.52 (m, 3H), 7.48 – 7.35 (m, 6H), 6.55 (s, 1H), 5.46 (dt, *J* = 3.6, 0.9 Hz, 1H), 4.84 (dt, *J* = 5.3, 4.0 Hz, 1H), 4.76 – 4.62 (m, 2H), 4.52 (d, *J* = 1.1 Hz, 1H); ^{13}C NMR (100.68 MHz, $CDCl_3$) δ 166.3, 165.8, 164.9, 134.0, 133.9, 133.4, 130.0, 130.0, 129.9, 129.7, 129.3, 128.9, 128.7, 128.6, 128.5, 100.7, 83.6, 78.0, 69.9, 63.6; HRMS (ESI-MS): m/z calcd for $C_{26}H_{21}N_3O_7$ $[M+Na]^+$: 510.1277, Found: 510.1305.

Compound 22: A solution of compound **21** (400 mg, 0.821 mmol) in a mixture of 12 mL DMF: H2O (2:1) was prepared. To this solution, TEG-alkyne **18** (317 mg, 0.821 mmol) and sodium ascorbate (163 mg, 0.821 mmol) were added at room temperature, followed by the addition of $CuSO_4{\cdot}5H_2O$ (102 mg, 0.410 mmol). The reaction mixture was stirred for 18h. Upon completion, water (50 mL) was added, and the mixture was extracted with ethyl acetate (2 x 30 mL). The combined organic layers were washed with brine solution (2 x 30 mL), dried over $MgSO_4$, filtered, and concentrated under reduced pressure. Flash chromatography purification using ethyl acetate:methanol eluent gradient (99:01 to 96:4) resulted in the isolation of compound **22** as a sticky solid with a yield of 71% (510 mg). IR (cm^{-1}, $CHCl_3$): 3005, 2989, 2922, 2852, 2707, 1725, 1453, 1275, 1261, 1024, 764, 710; ^{1}H NMR (400.34 MHz, $CDCl_3$) δ 8.11 – 8.00 (m, 4H), 7.98 – 7.94 (m, 2H), 7.93 (s, 1H), 7.79 – 7.71 (m, 2H), 7.64 – 7.57 (m, 2H), 7.56 – 7.50 (m, 1H), 7.47 – 7.35 (m, 6H), 7.33 – 7.27 (m, 2H), 6.83 (s, 1H), 5.93 (ddd, *J* = 5.2, 2.1, 0.7 Hz, 1H), 5.49 (dd, *J* = 2.2, 0.8 Hz, 1H), 4.99 (td, *J* = 5.3, 3.8 Hz, 1H), 4.87 (dd, *J* = 12.1, 3.9 Hz, 1H), 4.77 (dd, *J* = 12.1, 5.5 Hz, 1H), 4.68 – 4.57 (m, 2H), 4.15 – 4.06 (m, 2H), 3.70 – 3.62 (m, 6H), 3.62 – 3.58 (m, 4H), 3.58 – 3.51 (m, 4H), 2.41 (s, 3H); ^{13}C NMR (100.68 MHz, $CDCl_3$) δ 166.2, 165.7, 164.8, 146.2, 144.8, 134.1, 134.0, 133.3, 133.1, 130.0, 130.0, 129.9, 129.9, 129.5, 128.9, 128.7, 128.7, 128.5, 128.0, 122.9, 100.7, 83.2, 78.4, 70.8, 70.7, 70.6, 70.6,

69.9, 69.3, 68.7, 64.5, 63.3, 21.7; HRMS (ESI-MS): m/z calcd for $C_{44}H_{47}N_3O_{14}S$ $[M+Na]^+$: 896.2676, Found: 896.2665.

Compound 9: In a 10 mL round-bottom flask, a solution of compound **22** (450 mg, 0.515 mmol) in DMF (5 mL) was prepared. NaN_3 (335 mg, 5.15 mmol) was added to the solution, and the reaction mixture stirred overnight at room temperature. After completion, water (50 mL) was added, and the mixture was extracted with ethyl acetate (2 x 30 mL). The organic layer was washed with brine (2 x 30 mL), dried over $MgSO_4$, and the solvent was evaporated under vacuum. The crude product was then purified by flash column chromatography using an eluent gradient ethyl acetate:methanol (99:1 to 96:4), resulting in the isolation of 315 mg of compound **9** as a sticky solid with an 82% yield. IR (cm^{-1}, $CHCl_3$): 3005, 2989, 2922, 2852, 2707, 2100, 1725, 1452, 1276, 1261, 1096, 897, 780, 764, 710; ^1H NMR (400.34 MHz, $CDCl_3$) δ 8.12 – 8.01 (m, 4H), 7.97 (dt, *J* = 8.5, 1.6 Hz, 2H), 7.91 (s, 1H), 7.62 (td, *J* = 7.4, 1.4 Hz, 2H), 7.57 – 7.51 (m, 1H), 7.48 – 7.36 (m, 6H), 6.83 (s, 1H), 5.93 (dd, *J* = 5.2, 2.1 Hz, 1H), 5.48 (dd, *J* = 2.1, 0.7 Hz, 1H), 4.99 (td, *J* = 5.3, 3.9 Hz, 1H), 4.88 (dd, *J* = 12.2, 3.8 Hz, 1H), 4.78 (dd, *J* = 12.2, 5.5 Hz, 1H), 4.70 – 4.59 (m, 2H), 3.72 – 3.60 (m, 14H), 3.39 – 3.32 (m, 2H); ^{13}C NMR (100.68 MHz, $CDCl_3$) δ 166.3, 165.7, 164.9, 146.3, 134.1, 134.1, 133.4, 130.1, 130.0, 129.9, 129.5, 129.0, 128.8, 128.8, 128.6, 122.9, 100.8, 83.3, 78.4, 70.8, 70.8, 70.8, 70.8, 70.7, 70.6, 70.1, 70.0, 64.6, 63.3, 50.8; HRMS (ESI-MS): m/z calcd for $C_{37}H_{40}N_6O_{11}$ $[M+Na]^+$: 767.2653, Found: 767.2642.

Compound 7: In a 5 mL round-bottom flask, compound **9** (100 mg, 0.134 mmol) was dissolved in a mixture of THF: H_2O (1.0 mL, 2:1). To this solution, $LiOH{\cdot}H_2O$ (28 mg, 0.671 mmol) was added and the reaction mixture was stirred for 30 minutes at room temperature. The reaction was quenched by the addition of Amberlite IR-120, H^+ form. The resin was filtered, and the solvent was evaporated

under vacuum. The crude product was purified by flash silica gel column chromatography, resulting in the isolation of 37 mg (64% yield) of the desired product **7** as a sticky solid. IR (cm^{-1}, $CHCl_3$): 3369, 3006, 2989, 2922, 2707, 2109, 1462, 1276, 1090, 764; HRMS (ESI-MS): m/z calcd for $C_{16}H_{28}N_6O_8$ $[M+Na]^+$: 455.1866, Found: 455.1858.

Compound 5: In a 10 mL round-bottom flask, compound **21** (300 mg, 0.615 mmol) was dissolved in a mixture of THF: H_2O (3 mL, 2:1). $LiOH{\cdot}H_2O$ (93 mg, 2.22 mmol) was then added, and the reaction mixture was stirred for 30 min at room temperature. The reaction was neutralized by the addition of Amberlite IR-120, H^+ form. The resin was filtered, and the solvent was removed by *vacuo.* Purification of the crude product *via* flash column chromatography yielded compound **5** in 70% yield (75mg) as a sticky solid. IR (cm^{-1}, $CHCl_3$): 3368, 2923, 2853, 2113, 1456, 1348, 1261, 1084, 1006, 750; ^1H NMR (400.34 MHz, $CDCl_3$) δ 5.18 (d, *J* = 3.3 Hz, 1H), 4.39 – 4.36 (m, 1H), 4.01 (ddd, *J* = 14.7, 11.2, 2.5 Hz, 2H), 3.89 – 3.84 (m, 2H), 3.75 (ddd, *J* = 3.3, 2.1, 1.2 Hz, 1H), 3.56 (ddd, *J* = 15.1, 12.5, 1.9 Hz, 2H), 3.48 – 3.44 (m, 1H), 3.44 – 3.40 (m, 2H); ^{13}C NMR (100.68 MHz, $CDCl_3$) δ 98.0, 93.9, 73.2, 70.5, 69.6, 68.6, 67.8, 67.2, 63.8, 62.4, 49.6, 49.4, 49.3, 49.2, 49.1, 49.0, 48.8, 48.6, 48.4.

Compound 6: To a solution of 2-azido arabinose **5** (50 mg, 0.286 mmol) in pyridine, acetic anhydride (108 µL, 1.14 mmol) was added dropwise, followed by the catalytic amount of DMAP under nitrogen atmosphere. The reaction mixture was stirred for 3h at room temperature. Upon completion, the reaction solvent was evaporated under reduced pressure, and the crude was purified by silica gel column chromatography to give compound **6** as a sticky solid (57 mg, 66% yield). IR (cm^{-1}, $CHCl_3$): 3006, 2989, 2923, 2851, 2707, 2111, 1747, 1456, 1372, 1275, 1261, 1216, 1091, 1011, 764; ^1H

NMR (400.34 MHz, $CDCl_3$) δ 6.29 (d, *J* = 3.5 Hz, 1H), 5.46 (d, *J* = 8.3 Hz, 1H), 5.36 (dt, *J* = 3.5, 1.7 Hz, 1H), 5.32 (dd, *J* = 10.8, 3.3 Hz, 1H), 5.28 – 5.24 (m, 1H), 4.89 (dd, *J* = 10.6, 3.4 Hz, 1H), 4.05 – 3.99 (m, 2H), 3.96 (dd, *J* = 10.8, 3.6 Hz, 1H), 3.89 – 3.85 (m, 1H), 3.81 (dd, *J* = 13.3, 2.1 Hz, 1H), 3.77 – 3.73 (m, 1H), 2.19 (s, 3H), 2.17 – 2.16 (m, 9H), 2.10 (s, 3H), 2.09 (s, 3H); ^{13}C NMR (100.68 MHz, $CDCl_3$) δ 170.2, 170.0, 169.8, 168.9, 93.5, 91.1, 71.1, 68.4, 68.1, 67.3, 65.3, 62.8, 60.1, 57.2, 21.0, 21.0, 21.0, 20.8, 20.8; HRMS (ESI-MS): m/z calcd for $C_{11}H_{15}N_3O_7$ $[M+Na]^+$: 324.0808, Found: 307.0798.

Compound 8: A solution of compound **6** (40 mg, 0.133 mmol) in a mixture of DMF: H_2O (1.5 mL, 2:1) was prepared. To this solution, PEG-alkyne **18** (51 mg, 0.133 mmol) and sodium ascorbate (26 mg, 0.331 mmol) were added at room temperature, followed by the addition of $CuSO_4{\cdot}5H_2O$ (17 mg, 0.066 mmol). The reaction mixture was stirred overnight at room temperature. After completion, water (10 mL) was added, and the mixture was extracted with ethyl acetate (2 x 10 mL). The combined organic layers were washed with a brine solution (2 x 10 mL), dried over $MgSO_4$, filtered, and concentrated under reduced pressure. The crude residue was used directly for the subsequent reaction.

The crude residue (91 mg, 0.132 mmol) was dissolved in 1 mL of DMF, followed by the addition of NaN_3 (86 mg, 1.32 mmol). The reaction mixture was stirred overnight at room temperature. After completion, the reaction was diluted with 10 mL of ethyl acetate and washed with a mixture of cold water and brine. The organic layer was then dried over $MgSO_4$, and the solvent was concentrated under reduced pressure. Subsequently, the crude product was purified by silica gel column chromatography using an ethyl acetate:methanol eluent gradient (99:1 to 96:4), resulting in the isolation of 41 mg of compound **8** as a sticky solid with a yield of 55%. IR (cm^{-1}, $CHCl_3$): 3006, 2939,

2922, 2851, 2707, 2109, 1747, 1462, 1372, 1276, 1217, 1045, 764; ^{1}H NMR (500.23 MHz, $CDCl_3$) δ 7.63 (s, 1H), 7.61 (s, 1H), 6.39 (d, *J* = 3.4 Hz, 1H), 6.05 (d, *J* = 8.6 Hz, 1H), 5.89 (dd, *J* = 11.8, 3.3 Hz, 1H), 5.69 (dd, *J* = 11.2, 3.4 Hz, 1H), 5.51 – 5.48 (m, 1H), 5.45 (ddd, *J* = 3.4, 1.7, 1.0 Hz, 1H), 5.39 (dd, *J* = 11.8, 3.5 Hz, 1H), 4.82 (dd, *J* = 11.2, 8.5 Hz, 1H), 4.69 (s, 2H), 4.67 (s, 2H), 4.16 (ddd, *J* = 13.7, 5.0, 1.7 Hz, 2H), 3.99 (dd, *J* = 13.6, 1.3 Hz, 1H), 3.94 (dd, *J* = 13.4, 2.0 Hz, 1H), 3.71 – 3.63 (m, 28H), 3.39 (t, *J* = 5.2 Hz, 4H), 2.20 (s, 6H), 2.09 (s, 3H), 2.00 (s, 3H), 1.88 (s, 6H); ^{13}C NMR (125.80 MHz, $CDCl_3$) δ 170.2, 170.1, 169.9, 169.3, 168.4, 168.3, 145.8, 145.5, 123.4, 121.7, 92.8, 91.3, 70.8, 70.8, 70.8, 70.7, 70.7, 70.7, 70.7, 70.2, 70.0, 69.9, 67.8, 67.2, 66.2, 65.6, 64.9, 64.7, 63.0, 59.8, 57.8, 50.8, 21.0, 21.0, 20.9, 20.7, 20.6, 20.5; HRMS (ESI-MS): m/z calcd for $C_{22}H_{34}N_6O_{11}$ $[M+H]^+$: 581.2183, Found: 581.2174.

Compound 25: To a solution of the glycosyl donor **23** (1g, 1.0 mmol) and acceptor **24** (220 mg, 1.0 mmol) in anhydrous CH_2Cl_2 (7mL) was added freshly activated 4Å MS powder at 25 °C. After stirring at 25 °C for 10 minutes chloro[tris(2,4-di-tert-butylphenyl)phosphite]gold(I) (8mol%) and AgOTf (8mol%) were added to the reaction mixture and stirred for 15-25 minutes if the reaction was carried out at 25°C. the reaction was neutralized by Et_3N and filtered through a bed of Celite®. The filtrate was concentrated in vacuo and the residue was purified by silica gel column chromatography using n-hexane/EtOAc as mobile phase to afford **25** (550 mg, 70-85%) yield. IR (cm^{-1}, $CHCl_3$): 3068, 2927, 2160, 1730, 1601, 1413, 1268, 1110, 1060, 1032, 982, 712; ^{1}H NMR (400 MHz, $CDCl_3$) δ 8.11 – 7.98 (m, 6H), 7.59 (q, *J* = 7.5 Hz, 2H), 7.53 – 7.38 (m, 5H), 7.31 – 7.26 (m, 2H), 5.56 (s, 1H), 5.35 (s, 1H), 4.83 (dd, *J* = 11.9, 3.3 Hz, 1H), 4.71 – 4.59 (m, 2H), 3.98 – 3.91 (m, 1H), 3.79 – 3.58 (m, 14H), 3.35 (t, *J* = 5.0 Hz, 2H); ^{13}C NMR (101 MHz, $CDCl_3$) δ 166.37, 165.93, 165.53, 133.65, 133.62, 133.18, 130.08, 129.99, 129.88, 129.85, 105.88, 82.19, 81.15, 78.08, 70.86, 70.82, 70.76, 70.73,

70.37, 70.11, 66.91, 63.88, 50.76. HRMS (ESI-MS): m/z calcd for $C_{34}H_{37}N_3O_{11}$ $[M+Na]^+$: 686.2326; Found: 686.2322.

Compound 10: NaOMe (45 mg, 0.3 mmol per benzoate) was added to a solution of the benzoate **25** (550mg,1 mmol) in CH_2Cl_2:MeOH (1:4) and stirred for 8 h at 25 °C. The reaction mixture was concentrated in vacuo to obtain a residue purified by column chromatography (ethyl acetate/hexanes) to obtain alcohol. Yield for **10,** 230 mg, 79%; IR (cm^{-1}, $CHCl_3$): 3245, 2166, 1690, 1632,1368, 1227, 1090; ^1H NMR (400 MHz, DMSO-d_6) δ 5.10 (d, *J* = 5.1 Hz, 1H), 4.89 (d, *J* = 5.4 Hz, 1H), 4.52 (s, 1H), 4.43 (d, *J* = 1.7 Hz, 1H), 3.51 – 3.43 (m, 2H), 3.27 – 3.19 (m, 14H), 3.08 – 3.05 (m, 2H); ^{13}C NMR (101 MHz, DMSO-d_6) δ 108.22, 83.99, 82.29, 77.36, 70.07, 70.04, 69.97, 69.94, 69.52, 66.54, 61.62, 50.30. HRMS (ESI-MS): m/z calcd for $C_{13}H_{25}N_3O_8$ $[M+Na]^+$: 374.1539; Found: 374.1530.

Compound 27: This compound was synthesized from compound **26** using the procedure outlined for the preparation of compound **25**. glycosyl donor **26** (1 g, 1.0 mmol) and acceptor **24** (293 mg, 1.0 mmol) in anhydrous CH_2Cl_2 (7mL). Yield for **27,** 800 mg, 75%. IR (cm^{-1}, $CHCl_3$): 2860, 2160, 1722, 1601, 1445, 1326, 1228, 1102, 950, 702; ^1H NMR (400 MHz, $CDCl_3$) δ 8.07 (d, *J* = 7.5 Hz, 2H), 7.99 (d, *J* = 7.4 Hz, 2H), 7.72 (dd, *J* = 5.8, 2.0 Hz, 4H), 7.61 – 7.54 (m, 2H), 7.46 (t, *J* = 7.7 Hz, 2H), 7.41 – 7.32 (m, 8H), 5.62 (d, *J* = 5.1 Hz, 1H), 5.50 (s, 1H), 5.28 (s, 1H), 4.41 (q, *J* = 4.6 Hz, 1H), 4.02 (d, *J* = 4.5 Hz, 2H), 3.96 – 3.88 (m, 1H), 3.77 – 3.72 (m, 3H), 3.70 (dd, *J* = 6.0, 3.5 Hz, 2H), 3.65 – 3.62 (m, 8H), 3.37 – 3.33 (m, 2H), 1.06 (s, 9H); ^{13}C NMR (101 MHz, $CDCl_3$) δ 165.71, 165.52, 135.78, 135.76, 133.43, 133.40, 130.02, 129.78, 129.64, 129.35, 128.49, 127.78, 106.00, 83.09, 82.46, 77.59, 70.83, 70.81, 70.75, 70.73, 70.40, 70.09, 66.80, 63.61, 50.75, 26.88, 19.42. HRMS (ESI-MS): m/z calcd for $C_{43}H_{51}N_3O_{10}Si$ $[M+Na]^+$: 820.3241; Found: 820.3240.

Compound 28: HF·py (450 µL, 5 mmol) was added dropwise under an inert atmosphere to a solution of silyl ether **27** (800mg,1 mmol) in pyridine (3 times to the volume of HF·py) at 0 °C. The reaction mixture was stirred for 5 h at 25°C, 6N HCl was added to quench the reaction at 0°C, diluted with EtOAc, and washed successively with ice-cold water, saturated solution of $NaHCO_3$ (aq), and brine solution. The organic layer was dried with anhydrous Na_2SO_4, concentrated in vacuo to obtain a residue that was purified by silica gel column chromatography (ethyl acetate/hexanes) to furnish the alcohol. Yield for **28,** 430 mg, 77%; IR (cm^{-1}, $CHCl_3$): 3050, 2911, 2737, 2158, 1721, 1602, 1176,1036, 925, 700; ^1H NMR (400 MHz, $CDCl_3$) δ 8.08 – 8.02 (m, 4H), 7.60 – 7.55 (m, 2H), 7.47 – 7.42 (m, 4H), 5.55 (s, 1H), 5.42 – 5.40 (m, 1H), 5.29 (s, 1H), 4.35 (q, *J* = 4.2 Hz, 1H), 3.99 (td, *J* = 11.3, 10.7, 3.7 Hz, 2H), 3.95 – 3.86 (m, 1H), 3.76 – 3.70 (m, 3H), 3.67 – 3.57 (m, 11H), 3.34 (t, *J* = 5.0 Hz, 2H); ^{13}C NMR (101 MHz, $CDCl_3$) δ 166.24, 165.39, 133.64, 133.63, 130.02, 129.93, 129.31, 129.20, 128.63, 128.58, 105.70, 83.65, 81.93, 77.95, 70.79, 70.72, 70.67, 70.40, 70.07, 66.82, 62.45, 50.73. HRMS (ESI-MS): m/z calcd for $C_{27}H_{33}N_3O_{10}$ $[M+Na]^+$: 582.2064; Found: 582.2066.

Compound 29: This compound was synthesized from compound **28** using the procedure outlined for the preparation of compound **25**. Glycosyl donor **23** (800 mg, 1.0 mmol) and acceptor **28** (731 mg, 1.0 mmol) in anhydrous CH_2Cl_2 (7mL). Yield for **29**, 950mg, 73%). IR (cm^{-1}, $CHCl_3$): 3067, 2929, 2168, 1718, 1600, 1480, 1433, 1247, 1156, 1104, 1056, 1027, 969, 710; ^1H NMR (400 MHz, $CDCl_3$) δ 8.08 – 7.91 (m, 10H), 7.62 – 7.35 (m, 12H), 7.34 – 7.21 (m, 5H), 5.63 (d, *J* = 6.0 Hz, 2H), 5.58 (d, *J* = 6.1 Hz, 2H), 5.46 (s, 1H), 5.29 (s, 1H), 4.84 (dd, *J* = 11.8, 3.2 Hz, 1H), 4.78 – 4.73 (m, 1H), 4.67 (dd, *J* = 11.9, 4.6 Hz, 1H), 4.52 – 4.46 (m, 1H), 4.24 (dd, *J* = 11.3, 4.5 Hz, 1H), 4.01 – 3.87 (m, 2H), 3.74 – 3.58 (m, 14H), 3.35 (t, *J* = 5.0 Hz, 2H); ^{13}C NMR (101 MHz, $CDCl_3$) δ 166.33, 165.85, 165.81, 165.45, 165.34, 133.57, 133.54, 133.43, 133.12, 129.99, 129.93, 129.87, 129.82, 129.38, 129.18,

129.13, 129.04, 128.62, 128.55, 128.43, 128.40, 105.96, 105.77, 82.03, 81.95, 81.89, 81.34, 77.93, 77.36, 70.84, 70.80, 70.74, 70.71, 70.35, 70.09, 66.75, 66.16, 63.75, 50.73. HRMS (ESI-MS): m/z calcd for $C_{53}H_{53}N_3O_{17}$ $[M+Na]^+$: 1026.3273; Found: 1026.3250.

Compound 11: This compound was synthesized from compound **29** using the procedure outlined for the preparation of compound **10**. NaOMe (77mg, 0.3 mmol per benzoate) was added to a solution of the benzoate **29** (950mg,1 mmol) in CH_2Cl_2:MeOH (1:4) and stirred for 7h at 25 °C. Yield for **11**, 370mg, 81%; IR (cm^{-1}, $CHCl_3$): 3312, 2160, 1692, 1602, 1405, 1328, 1217, 1090; ^1H NMR (400 MHz, Methanol-d_4) δ 4.95 (d, *J* = 1.3 Hz, 1H), 4.91 (d, *J* = 1.4 Hz, 1H), 4.07 – 4.03 (m, 1H), 3.99 (ddd, *J* = 6.2, 3.0, 1.3 Hz, 3H), 3.90 – 3.87 (m, 1H), 3.86 – 3.79 (m, 3H), 3.75 (dd, *J* = 11.8, 3.4 Hz, 1H), 3.69 – 3.62 (m, 15H), 3.40 – 3.36 (m, 2H); ^{13}C NMR (101 MHz, Methanol-d_4) δ 109.67, 109.60, 85.88, 83.84, 83.41, 83.08, 79.19, 78.73, 71.59, 71.54, 71.49, 71.41, 71.09, 68.10, 67.86, 63.05, 51.74. HRMS (ESI-MS): m/z calcd for $C_{18}H_{33}N_3O_{12}$ $[M+Na]^+$: 506.1962; Found: 506.1950.

Compound S4: To a solution of the alcohol **S3** (2gm, 1mmol) in anhydrous DMF was cooled to 0°C under nitrogen atmosphere and NaH (168mg, 60% oil dispersion, 1.3mmol per alcohol) was added and stirred for 10 minutes at 0°C, 2-bromomethyl naphthalene (1.24gm, 1.2 mmol per alcohol) was added and stirred for 2 h at 25°C. The reaction mixture was poured into cold water with vigorous shaking, extraction with ethyl acetate and combined EtOAc layers were washed with cold water, brine solution, and dried over Na_2SO_4. The EtOAc solution was decanted and evaporated in vacuo to obtain a reddish-brown coloured residue that was purified by column chromatography (ethyl acetate/hexanes) to obtain the naphthyl ether(s) as pale-yellow coloured syrup. Yield for **S4**, 2.19 g, 79%; IR (cm^{-1}, $CHCl_3$): 3068, 2830, 1704, 1600, 1430, 1326, 1122, 1012, 1077, 966, 710; ^1H NMR (400 MHz, $CDCl_3$) δ 7.85 – 7.78 (m, 4H), 7.67 – 7.64 (m, 4H), 7.50 – 7.39 (m, 5H), 7.35 (t, *J* = 7.1 Hz, 4H), 5.92 (d, *J* = 4.1 Hz, 1H), 4.78 (d, *J* = 2.3 Hz, 2H), 4.72 (d, *J* = 4.1 Hz, 1H), 4.28 (s, 2H), 3.83

(d, *J* = 7.0 Hz, 2H), 1.35 (s, 3H), 1.31 (s, 3H), 1.02 (s, 9H); ^{13}C NMR (101 MHz, $CDCl_3$) δ 135.74, 135.72, 135.06, 134.94, 133.41, 133.37, 133.23, 133.22, 129.89, 129.84, 128.47, 128.07, 127.88, 127.84, 126.69, 126.29, 126.12, 125.76, 112.62, 105.91, 85.43, 85.29, 82.99, 71.94, 63.58, 27.10, 26.92, 26.28, 19.31. HRMS (ESI-MS): m/z calcd for $C_{35}H_{40}N_5Si$ $[M+Na]^+$: 591.2543; Found: 591.2540.

Compound S5: Allyl alcohol (2.06 mL, 8 mmol) was added to a solution of acetonide **S4** (2.15 gm, 1 mmol) in CH_2Cl_2 (20 mL), and stirred at 60°C. After 5 min, P-TSA (196mg, 0.3 mmol) was added and continued stirring for an additional 2h, the reaction mixture was neutralized by the addition of Et_3N (excess), diluted with water and extracted with CH_2Cl_2. Combined CH_2Cl_2 layers were washed with a brine solution, and dried over anhydrous sodium sulphate. decanted and concentrated in vacuo to obtain a residue that was partially purified by silica gel column chromatography (ethyl acetate/hexanes) to obtain α/β-allyl furanosides which were separated by flash chromatography by obtaining diastereomerically pure as a pale-yellow syrup. Yield for **S5**, 900 mg, α isomer (42%), 800 mg β isomer (38%); IR (cm^{-1}, $CHCl_3$): 3068, 2900, 2820, 1690, 1456, 1268, 1210, 1050, 950, 932, 707; ^{1}H NMR (400 MHz, $CDCl_3$) δ 7.74 – 7.66 (m, 4H), 7.57 – 7.53 (m, 2H), 7.44 – 7.40 (m, 2H), 7.39 – 7.23 (m, 7H), 7.17 – 7.12 (m, 2H), 5.92 – 5.81 (m, 1H), 5.25 (dt, *J* = 17.2, 1.4 Hz, 1H), 5.13 – 5.08 (m, 1H), 5.03 (s, 1H), 4.77 (d, *J* = 12.4 Hz, 1H), 4.60 (d, *J* = 12.4 Hz, 1H), 4.22 – 4.13 (m, 3H), 3.98 (ddd, *J* = 14.7, 7.0, 1.8 Hz, 2H), 3.71 (dd, *J* = 11.2, 2.3 Hz, 1H), 3.47 (dd, *J* = 11.2, 2.0 Hz, 1H), 3.20 (dd, *J* = 10.2, 3.8 Hz, 1H), 0.89 (s, 9H); ^{13}C NMR (101 MHz, $CDCl_3$) δ 135.71, 135.60, 135.40, 134.49, 133.33, 133.15, 132.44, 132.42, 130.02, 130.00, 128.36, 128.03, 127.90, 127.79, 126.84, 126.24, 126.05, 125.87, 117.26, 108.00, 84.65, 84.39, 78.10, 78.08, 77.48, 77.16, 76.84, 72.19, 68.24, 64.20, 26.79, 19.17. HRMS (ESI-MS): m/z calcd for $C_{35}H_{40}O_5Si$ $[M+Na]^+$: 591.2543; Found: 591.2533.

Compound S6: A solution of the alcohol **S5** (900 mg, 1 mmol) in anhydrous pyridine and DMAP (37mg, 0.1 mmol) was cooled to 0 °C under a nitrogen atmosphere and benzoyl chloride (276 µL, 1.2 mmol per alcohol) was added to dropwise and stirred for 5 h at 25°C. The reaction mixture was poured into cold water with vigorous shaking, neutralized by the addition of 6N HCl solution, extracted with ethyl acetate and combined EtOAc layers were washed with cold water, sat. $NaHCO_3$, brine solution, and dried over Na_2SO_4. The EtOAc solution was decanted and evaporated in vacuo to obtain a reddish-brown coloured residue that was purified by column chromatography (ethyl acetate/hexanes) to obtain the required benzoate. Yield for **S6**, 850 mg, 80%; IR (cm^{-1}, $CHCl_3$): 3068, 2820, 2758, 1277, 1579, 1248, 1102, 1046, 951, 922, 702; ^1H NMR (400 MHz, $CDCl_3$) δ 7.95 (dd, *J* = 8.3, 1.2 Hz, 2H), 7.79 – 7.72 (m, 3H), 7.69 – 7.63 (m, 3H), 7.61 – 7.54 (m, 3H), 7.44 – 7.36 (m, 7H), 7.32 – 7.28 (m, 4H), 5.96 (dddd, *J* = 16.9, 11.0, 6.2, 5.0 Hz, 1H), 5.48 (d, *J* = 1.3 Hz, 1H), 5.36 (dq, *J* = 17.2, 1.5 Hz, 1H), 5.23 – 5.20 (m, 2H), 4.94 (d, *J* = 12.3 Hz, 1H), 4.76 (d, *J* = 12.3 Hz, 1H), 4.33 – 4.25 (m, 2H), 4.19 (d, *J* = 5.5 Hz, 1H), 4.10 (ddt, *J* = 13.1, 6.2, 1.2 Hz, 1H), 3.84 (dd, *J* = 4.1, 2.7 Hz, 2H), 0.95 (s, 9H); ^{13}C NMR (101 MHz, $CDCl_3$) δ 165.65, 135.78, 135.73, 135.20, 134.20, 133.48, 133.43, 133.32, 133.18, 129.94, 129.80, 129.77, 129.58, 128.54, 128.36, 128.04, 127.79, 127.76, 127.08, 126.12, 125.98, 117.65, 105.30, 83.60, 83.00, 82.50, 72.50, 68.00, 63.39, 26.83, 19.39; HRMS (ESI-MS): m/z calcd for $C_{42}H_{44}O_6Si$ $[M+Na]^+$: 695.2805; Found: 695.2802.

Compound 30: To the solution of the allyl glycoside **S6** (850 mg, 1 mmol) in CH_2Cl_2 (7mL), $PdCl_2$ (45mg, 0.2mmol) in MeOH(19mL) was added at 25°C and stirred for 4-5 h. After completion of the reaction, an excess amount of Et_3N was added and the solid residue was filtered off through a pad of Celite®. The solvent was evaporated in vacuo and the crude compound was purified by conventional silica gel column chromatography (ethyl acetate/hexanes) to obtain the desired hemiacetal. Yield for **30,** 550 mg, 69%. **Synthesis of ethynyl cyclohexyl glycosyl carbonate**

donor: To a rapidly stirring CH_2Cl_2 solution of arabinofuranosyl hemiacetal (550 mg, 1.0 mmol) and DMAP (114 mg, 1.1mmol) at 25ºC, ethynyl cyclohexyl (4-nitrophenyl) carbonate (268 mg, 1.1 mmol) was added and continued stirring at 25ºC for 2 h. After complete consumption of the hemiacetal, the reaction mixture was concentrated in vacuo and purified by silica gel column chromatography (n-hexane/EtOAc) to afford ethynyl cyclohexyl glycosyl carbonate donors containing trace quantity of the 4-nitro phenol. Eluents containing the compound are concentrated and redissolved in CH_2Cl_2 and washed with sat. aq. $NaHCO_3$ solution to get 530 mg, 80% yield. IR (cm^{-1}, $CHCl_3$): 3067, 2929, 2757, 1662, 1700, 1590, 1455, 1140, 1102, 1061, 1012, 940, 910, 849, 707; ^1H NMR (400 MHz, $CDCl_3$) δ 7.95 – 7.91 (m, 2H), 7.80 – 7.70 (m, 4H), 7.60 – 7.54 (m, 5H), 7.47 – 7.34 (m, 7H), 7.28 (d, *J* = 7.4 Hz, 4H), 6.27 (s, 1H), 5.63 (s, 1H), 4.99 (d, *J* = 12.2 Hz, 1H), 4.81 (d, *J* = 12.2 Hz, 1H), 4.48 (q, *J* = 4.4 Hz, 1H), 4.31 (d, *J* = 4.8 Hz, 1H), 3.82 (d, *J* = 4.4 Hz, 2H), 2.64 (s, 1H), 2.21 (td, *J* = 14.4, 14.0, 6.9 Hz, 2H), 1.95 – 1.85 (m, 2H), 1.69 – 1.57 (m, 4H), 1.37 – 1.25 (m, 2H), 0.91 (s, 9H); ^{13}C NMR (101 MHz, $CDCl_3$) δ 165.37, 151.46, 135.68, 135.03, 133.61, 133.34, 133.22, 133.19, 130.00, 129.85, 129.80, 129.17, 128.60, 128.40, 128.07, 127.81, 127.79, 127.05, 126.16, 126.04, 125.89, 103.15, 85.86, 82.84, 82.53, 81.62, 78.38, 75.34, 72.55, 62.86, 37.00, 36.84, 26.78, 25.10, 22.73, 19.34. HRMS (ESI-MS): m/z calcd for $C_{37}H_{41}O_7Si$ $[M+Na]^+$: 648.2519; Found: 648.2511.

Compound 31: This compound was synthesized from compound **30** using the procedure outlined for the preparation of compound **25**. glycosyl donor **30** (1.5 g, 1.0 mmol) and acceptor **24** (420 mg, 1.0 mmol) in anhydrous CH_2Cl_2 (10 mL). Yield for **30,** 1.2 g, 76%. IR (cm^{-1}, $CHCl_3$): 2760, 2168, 1720 1600, 1430, 1346, 1246, 1122, 1012, 1057, 962, 709; ^1H NMR (400 MHz, $CDCl_3$) δ 7.96 (dd, *J* = 8.3, 1.1 Hz, 2H), 7.79 – 7.68 (m, 4H), 7.65 – 7.55 (m, 5H), 7.44 – 7.36 (m, 7H), 7.32 – 7.28 (m, 4H), 5.49 (d, *J* = 1.2 Hz, 1H), 5.23 (s, 1H), 4.95 (d, *J* = 12.1 Hz, 1H), 4.75 (d, *J* = 12.2 Hz, 1H), 4.30 (q, *J* = 4.2 Hz, 1H), 4.20 (d, *J* = 5.5 Hz, 1H), 3.90 (dd, *J* = 9.7, 5.3 Hz, 1H), 3.84 (q, *J* = 4.3 Hz, 2H),

3.74 – 3.67 (m, 5H), 3.64 – 3.60 (m, 8H), 3.34 (t, *J* = 5.1 Hz, 2H), 0.95 (s, 9H); ^{13}C NMR (101 MHz, $CDCl_3$) δ 165.57, 135.75, 135.70, 135.22, 133.43, 133.30, 133.16, 129.91, 129.79, 129.76, 129.58, 128.54, 128.33, 128.02, 127.78, 127.75, 127.07, 126.13, 126.00, 106.46, 83.56, 83.07, 82.37, 72.51, 70.84, 70.80, 70.76, 70.72, 70.40, 70.08, 66.76, 63.27, 50.78, 26.81, 19.37. HRMS (ESI-MS): m/z calcd for $C_{36}H_{46}N_3O_8Si$ $[M+Na]^+$: 699.2952; Found: 699.2952.

Compound 32: This compound was synthesized from compound **31** using the procedure outlined for the preparation of compound **10**. NaOMe (24 mg, 0.3 mmol per benzoate) was added to a solution of the benzoate **31** (1.2 g,1 mmol) in CH_2Cl_2:MeOH (1:4) and stirred for 8 h at 25 °C. Yield for **32,** 870g, 83%; IR (cm^{-1}, $CHCl_3$): 3458, 2760, 2168, 1430, 1346, 1256, 1100, 1010, 1057, 898, 700; ^1H NMR (400 MHz, $CDCl_3$) δ 7.84 – 7.76 (m, 4H), 7.65 (dd, *J* = 7.8, 1.3 Hz, 2H), 7.55 (d, *J* = 6.9 Hz, 2H), 7.48 – 7.33 (m, 8H), 7.28 – 7.24 (m, 2H), 5.12 (s, 1H), 4.86 (d, *J* = 12.2 Hz, 1H), 4.70 (d, *J* = 12.3 Hz, 1H), 4.30 (s, 1H), 4.22 (q, *J* = 2.7 Hz, 1H), 4.06 (dd, *J* = 3.8, 1.6 Hz, 1H), 3.89 (dt, *J* = 8.4, 4.4 Hz, 1H), 3.81 (dd, *J* = 11.2, 2.6 Hz, 1H), 3.74 – 3.65 (m, 5H), 3.61 (q, *J* = 3.4 Hz, 9H), 3.35 – 3.31 (m, 2H), 0.99 (s, 9H).

^{13}C NMR (101 MHz, $CDCl_3$) δ 135.70, 135.61, 135.50, 133.31, 133.11, 132.65, 132.63, 129.95, 129.92, 128.27, 128.00, 127.85, 127.77, 126.73, 126.20, 126.01, 125.89, 109.28, 84.45, 83.91, 78.64, 72.16, 70.73, 70.69, 70.66, 70.63, 70.04, 67.16, 64.11, 50.70, 26.79, 19.19. HRMS (ESI-MS): m/z calcd for $C_{29}H_{42}N_3O_7Si$ $[M+Na]^+$: 595.2690; Found: 595.2650.

Compound 33: This compound was synthesized from compound **32** using the procedure outlined for the preparation of compound **25**. glycosyl donor **23** (1 g, 1.0 mmol) and acceptor **32** (835 mg, 0.7 mmol) in anhydrous CH_2Cl_2 (10 mL). Yield for **33**, 1.3 g, 68%; IR (cm^{-1}, $CHCl_3$): 3068, 2860,

2160, 1724, 1600, 1450, 1356, 1266, 1122, 1022, 1067, 967, 702; ^{1}H NMR (400 MHz, $CDCl_3$) δ 8.04 (dd, *J* = 8.3, 1.2 Hz, 2H), 7.99 (ddd, *J* = 8.3, 4.7, 1.2 Hz, 4H), 7.77 – 7.58 (m, 9H), 7.52 (dtd, *J* = 10.2, 7.5, 1.3 Hz, 2H), 7.44 – 7.34 (m, 9H), 7.34 – 7.26 (m, 6H), 5.58 – 5.55 (m, 1H), 5.45 (d, *J* = 1.3 Hz, 1H), 5.34 (s, 1H), 5.16 (s, 1H), 4.84 – 4.66 (m, 4H), 4.60 (q, *J* = 4.9 Hz, 1H), 4.40 – 4.37 (m, 1H), 4.21 (q, *J* = 4.4 Hz, 1H), 4.13 (dd, *J* = 6.0, 2.4 Hz, 1H), 3.86 – 3.81 (m, 3H), 3.67 – 3.56 (m, 14H), 3.34 – 3.30 (m, 2H), 0.99 (s, 9H); ^{13}C NMR (101 MHz, $CDCl_3$) δ 166.27, 165.83, 165.42, 135.80, 135.73, 135.37, 133.67, 133.63, 133.50, 133.46, 133.30, 133.21, 133.08, 130.04, 129.99, 129.87, 129.80, 129.78, 129.09, 128.66, 128.60, 128.47, 128.28, 127.99, 127.78, 127.75, 127.72, 126.75, 126.14, 125.93, 125.82, 106.96, 105.48, 86.61, 82.99, 82.61, 82.59, 81.46, 77.76, 72.34, 70.73, 70.68, 70.37, 70.06, 66.89, 63.87, 63.82, 50.74, 26.85, 19.35. HRMS (ESI-MS): m/z calcd for $C_{55}H_{62}N_3O_4Si$ $[M+Na]^+$: 1039.3899; Found: 1039.3872.

Compound 34: HF·py (540μL, 5 mmol) was added dropwise under an inert atmosphere to a solution of silyl ether **33** (1.3gm, 1 mmol) in pyridine (3 times to the volume of HF·py) at 0 °C. The reaction mixture was stirred for 5 h at 25°C, 6N HCl was added to quench the reaction at 0°C, diluted with EtOAc, and washed successively with ice-cold water, saturated solution of $NaHCO_3$ (aq), and brine solution. The organic layer was dried with anhydrous Na_2SO_4, concentrated in vacuo (Crude yield for **34,** 830 mg) and directly proceeded for **Naphthyl deprotection:** 2,3-Dichloro-5,6-dicyano-1,4-benzoquinone (2 mmol) was added to a rapidly stirred solution of alcohol (1 mmol) in CH_2Cl_2:MeOH (1:4) at 25°C. After 4 h, the reaction mixture was quenched by the addition of Et_3N (the reaction mixture turns black from brown) the solvent was evaporated under reduced pressure and the residue was purified by silica gel column chromatography (ethyl acetate/hexanes) to furnish the alcohol as a pale yellow coloured syrup. IR (cm^{-1}, $CHCl_3$): $\tilde{\nu}$ = 3067, 2931, 2857, 2158, 1721, 1602, 1470, 1259, 1176,1036, 945, 709; ^{1}H NMR (400 MHz, $CDCl_3$) δ 8.05 (t, *J* = 8.1 Hz, 4H), 7.99 (dd, *J* = 8.1,

1.5 Hz, 2H), 7.61 – 7.56 (m, 2H), 7.49 (dt, *J* = 19.3, 7.5 Hz, 4H), 7.42 – 7.37 (m, 2H), 7.33 – 7.27 (m, 2H), 5.60 (d, *J* = 4.9 Hz, 1H), 5.48 (s, 1H), 5.48 (d, *J* = 1.5 Hz, 1H), 5.22 (s, 1H), 4.82 (dd, *J* = 11.9, 3.6 Hz, 1H), 4.69 (dd, *J* = 11.9, 4.9 Hz, 1H), 4.63 – 4.59 (m, 1H), 4.26 (d, *J* = 1.6 Hz, 1H), 4.18 (p, *J* = 2.2 Hz, 1H), 4.12 (dd, *J* = 3.8, 1.6 Hz, 1H), 3.94 – 3.86 (m, 2H), 3.81 (td, *J* = 11.8, 11.0, 4.7 Hz, 2H), 3.66 – 3.60 (m, 13H), 3.40 – 3.36 (m, 2H); **^{13}C NMR (101 MHz, $CDCl_3$)** δ 166.25, 165.80, 165.68, 133.74, 133.21, 130.00, 129.98, 129.82, 129.70, 128.97, 128.84, 128.63, 128.43, 106.00, 105.44, 86.32, 86.29, 86.21, 86.13, 82.53, 81.48, 77.59, 75.95, 70.68, 70.62, 70.56, 70.46, 70.13, 70.02, 65.71, 65.68, 63.66, 62.83, 50.68. HRMS (ESI-MS): m/z calcd for $C_{39}H_{45}N_3O_{15}$ $[M+Na]^+$: 818.2748; Found: 818.2744.

Compound 12: This compound was synthesized from compound **34** using the procedure outlined for the preparation of compound **10**. NaOMe (61 mg, 0.3 mmol per benzoate) was added to a solution of the benzoate **34** (800 mg,1 mmol) in CH_2Cl_2:MeOH (1:4) and stirred for 8 h at 25 °C. Yield for **12,** 380mg, 85%; IR (cm^{-1}, $CHCl_3$): 3312, 2160, 1692, 1602, 1405, 1328, 1217, 1090; ^{1}H NMR (400 MHz, Methanol-*d*$_4$) δ 5.09 (d, *J* = 1.1 Hz, 1H), 5.08 (d, *J* = 1.6 Hz, 1H), 4.06 (dd, *J* = 3.5, 1.4 Hz, 1H), 4.01 (dd, *J* = 3.8, 1.7 Hz, 1H), 3.96 (qd, *J* = 7.5, 3.0 Hz, 3H), 3.88 – 3.83 (m, 2H), 3.81 – 3.78 (m, 1H), 3.76 (t, *J* = 2.6 Hz, 1H), 3.71 – 3.62 (m, 16H), 3.42 – 3.39 (m, 2H); ^{13}C NMR (101 MHz, Methanol-*d*$_4$) δ 109.38, 108.10, 89.80, 85.59, 84.48, 83.75, 78.65, 77.39, 71.59, 71.56, 71.49, 71.39, 71.10, 67.81, 62.96, 62.72, 51.75.

Compound S8: Tetra ethylene glycol (1.22 g, 6.29 mmol) was dissolved in CH_2Cl_2 (10 mL). To the solution triethylamine (2.19 mL, 15.74 mmol) and tosyl chloride (1.0 g, 5.25 mmol) were added, and the reaction mixture was stirred overnight at room temperature. After completion, the reaction mixture was washed sequentially with 1M HCl (50 mL x 3), sat. $NaHCO_3$ (50 mL x 3), and brine (50 mL) solution. The crude was

purified by flash chromatography using a DCM:MeOH (98:2) eluent, yielding compound **S8** in 76% yield (1.39 g).

Compound 18: To the solution of compound S8 (0.8 g, 2.3 mmol) and propargyl bromide (522 μL, 6.89 mmol) in a dry DMF (10 mL), NaH (60% oil dispersion, 138 mg, 3.44 mmol) was added portion-wise at 0°C under an inert atmosphere. The reaction mixture was allowed to stir at room temperature for 3h. After completion, the mixture was quenched by the addition of cold water and extracted with ethyl acetate. The organic layer was dried over $MgSO_4$, filtered, and concentrated. The crude was purified by flash chromatography using a DCM:MeOH (98:2) eluent, yielding compound **18** in 81% yield (720 mg). ^{1}H NMR (399.79 MHz, $CHCl_3$) δ 7.79 (d, *J* = 8.3 Hz, 2H), 7.33 (d, *J* = 8.2 Hz, 2H), 4.19 (d, *J* = 2.3 Hz, 2H), 4.15 (dd, *J* = 5.4, 4.2 Hz, 2H), 3.70 – 3.65 (m, 6H), 3.64 – 3.60 (m, 4H), 3.58 (s, 4H), 2.44 (s, 3H), 2.42 (t, *J* = 2.4 Hz, 1H); ^{13}C NMR (100.53 MHz, $CHCl_3$) δ 144.9, 133.1, 129.9, 128.1, 79.8, 74.6, 70.8, 70.7, 70.6, 70.5, 69.4, 69.2, 68.8, 58.5, 21.8.[1]

Compound 24: In an oven-dried RBF, compound **S8** (1 g, 1 mmol), and NaN_3 (374 mg, 2 mmol) were taken and dissolved in DMF under stirring. The resultant mixture was allowed to react for 12 hours. Upon completion, the reaction was quenched by the dropwise addition of water and extracted with EtOAc thrice. The combined organic layer was dried over Na_2SO_4 and concentrated under reduced pressure to get a crude product that was purified using silica gel column chromatography using Methanol/ ethyl acetate as eluent. The product was obtained as a pale-yellow liquid. Yield for **24,** 540mg, 85%; IR (cm^{-1}, $CHCl_3$): 3455, 3200, 2160, 1368, 1237, 860, 770; ^{1}H NMR (400 MHz, $CDCl_3$) δ 3.72 (s, 2H), 3.66 (d, *J* = 4.8 Hz, 10H), 3.62 – 3.59 (m, 2H), 3.39 (t, *J* = 5.0 Hz, 2H); ^{13}C NMR (101 MHz, $CDCl_3$) δ 72.61, 70.83, 70.79, 70.72, 70.47, 70.17, 61.88, 50.80. HRMS (ESI-MS): m/z calcd for $C_8H_{17}N_3O_4$ $[M+Na]^+$: 242.1117; Found: 242.1100.

Glycan Microbead Conjugation and Analysis:

Procedure for the Preparation of Propargylated Microbeads (MB-Alkyne): CML beads (500 µL, 4% w/v in H_2O, 0.033 µmol carboxylic functionalities) were centrifuged (8000 rpm, 8 min), and the supernatant was removed and replaced with PBS (500 µL). EDC (119 mg, 627 µmol) and propargylamine (29 µL, 462 µmol) were added to the suspension and the reaction mixture was agitated at 900 rpm for 24h. Then the suspension was centrifuged (8000 rpm, 8 min), the supernatant was removed, and the beads were washed 3 times with water (dist) and once with methanol (500 µL). Finally, the supernatant was replaced with PBS (500 µL) to provide 4% w/v solution of the MB-Alkyne.

General Protocol for the Functionalization of MB-Alkyne: To a suspension of MB-Alkyne in PBS (125 µL, 4% w/v, 8.3 nmol propargyl functionality), azido arabinose compound (**1-14** or linker **24**) (3.5 µmol), ascorbic acid sodium salt (21.3 mg, 90 µmol) and $CuSO_4 \cdot 5H_2O$ (5.1mg, 17.3 µmol) were added. The reaction mixture was shaken at 900 rpm for 24 h. Subsequently the reaction mixture was transferred to a 100kDa centrifuge filter and centrifuged (8000 rpm, 10 min). The residue was washed with distilled water (125 µL, twice), MeOH (125 µL) and distilled water (125 µL) again and then re-suspended in sterile PBS (125 µL) to provide a 4% w/v suspension of conjugated beads.

Coumarin Fluorescence Test to Confirm Microbead Glycan Functionalization: A fluorescence-based test to confirm the conjugation of the sugars' azido derivatives to MB-Alkyne was performed according to our previous work with some modifications [2]. A solution of 3-azido-7-hydroxycoumarin (2.5 µL, 1.0 mM) was added to MB-Alkyne or arabinose functionalised beads (15 µL, 4% w/v in PBS). After the addition of stock solutions of sodium ascorbate (6.3 µL, 10 mM) and $CuSO_4 \cdot 5H_2O$

(1.3 μL, 10 mM), the reaction mixture was shaken at 900rpm overnight. The beads were centrifuged at 8000 rpm for 15 mins and washed (1 × H_2O, 2 × MeOH). After the final wash, the reaction mixture was diluted into 300 μL MeOH and 3 × 100 μL portions were transferred into a 96-well plate and the fluorescence emission intensities were recorded (excitation = 355 nm; emission = 460 nm). As a control, a separate 15 μL portion of the 4% w/v in PBS solution of beads was subjected to the same overnight shaking, washing and dilution procedure in the absence of 3-azido-7-hydroxycoumarin, sodium ascorbate and $CuSO_4$, and fluorescence of this control sample was measured in the same way.

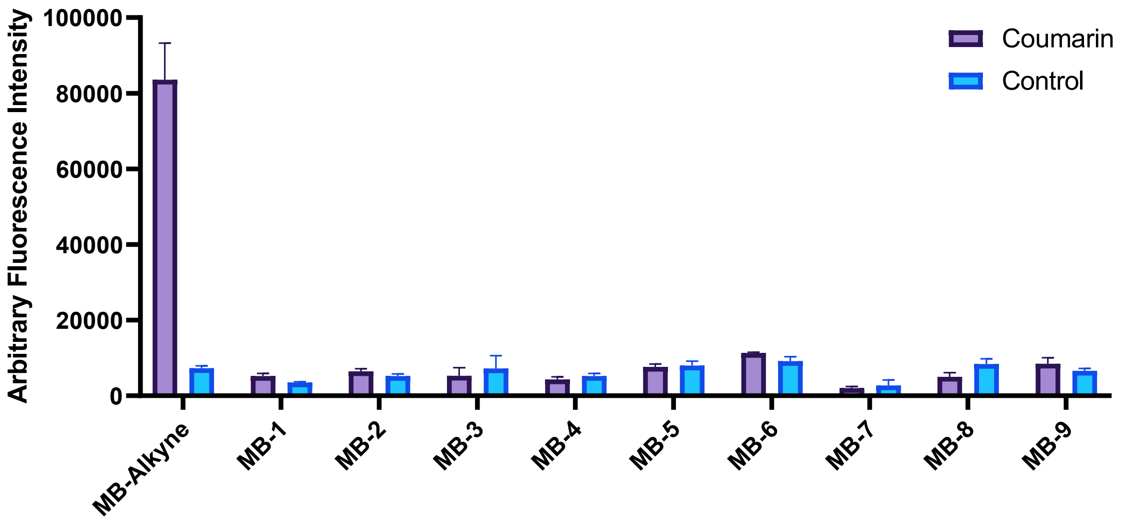

Figure S1. Fluorescence intensity (460 nm emission) of functionalised microbeads **MB-1-9** and **MB-Alkyne** after subjection to CuAAC conjugation conditions in the presence (Coumarin) and absence (Control) of 3-azido-7-hydroxycoumarin.

As expected, the fluorescence of CML beads didn't change compared to the control after subjecting them to reaction with 3-azido-7-hydroxycoumarin and $CuSO_4$/Na ascorbate, since no propargyl group was present and, therefore, no click reaction could occur. A significative increase in

fluorescence was observed for MB-Alkyne-coumarin compared to the MB-Alkyne only control, due to successful conjugation of the fluorescent coumarin moiety on the bead surface. Concerning the sugar-functionalized beads, the lack of change in fluorescence proved that there was no significant amount of propargyl groups left on the particle surface, and, therefore, the coupling of the azido-sugars was successful.

Microbead-Based Bacterial Assays:

Preparation of bacterial cultures: *S. aureus* Newman, *E. coli* BW25113, *K. pneumoniae* NCTC 5055 and *P. aeruginosa* PA01 and *S. aureus* SH1000, were grown in 10 mL Luria-Bertani (LB) broth for 18-20 h, 180 rpm at 37°C. *M. smegmatis* mc^2155 was grown in Middlebrook 7H9 broth supplemented with 1 mL Middlebrook ADC supplement (Sigma-Aldrich).

A] Microbead-Based Agglutination Assay

Experimental Protocol: Microbead-based agglutination assays were performed as outlined previously[2] with some modifications. 1 mL of bacterial overnight suspension was centrifuged (3,000 x g, 5 min), the supernatant was removed, and the bacteria pellet was washed with sterile PBS. The bacterial suspension was diluted to a final OD600 of 1.0 and 0.1 to give about 10^9 and 10^8 CFU/mL suspensions respectively.

20 μL of 10^9 CFU/mL or 10^8 CFU/mL bacterial suspension (20 μL of PBS was used as a negative control) was added to 10μL of 0.6% beads suspension in sterile PBS (freshly prepared from the 4% stock solution) and shaken at RT (1600rmp) for 1 h. After this time, the suspensions were vortex and then spread on glass slides using a pipette tip to approximately 1 cm in diameter. The slides were covered to avoid the drops to dry and, after 30 minutes, 5 images were taken from different sections of each drop at 10x magnification with ZEISS Primo Star iLED. The collected images were analyzed through Abacus analysis.

Each assay has been repeated 3 times (3 biological replicates) and each sample has been tested 3 times per each biological replicate (3 technical replicates each).

Microscope Images, Data Processing and Box Plots for bacteria samples incubated with glycan-functionalised microbeads.:

Microscope Images

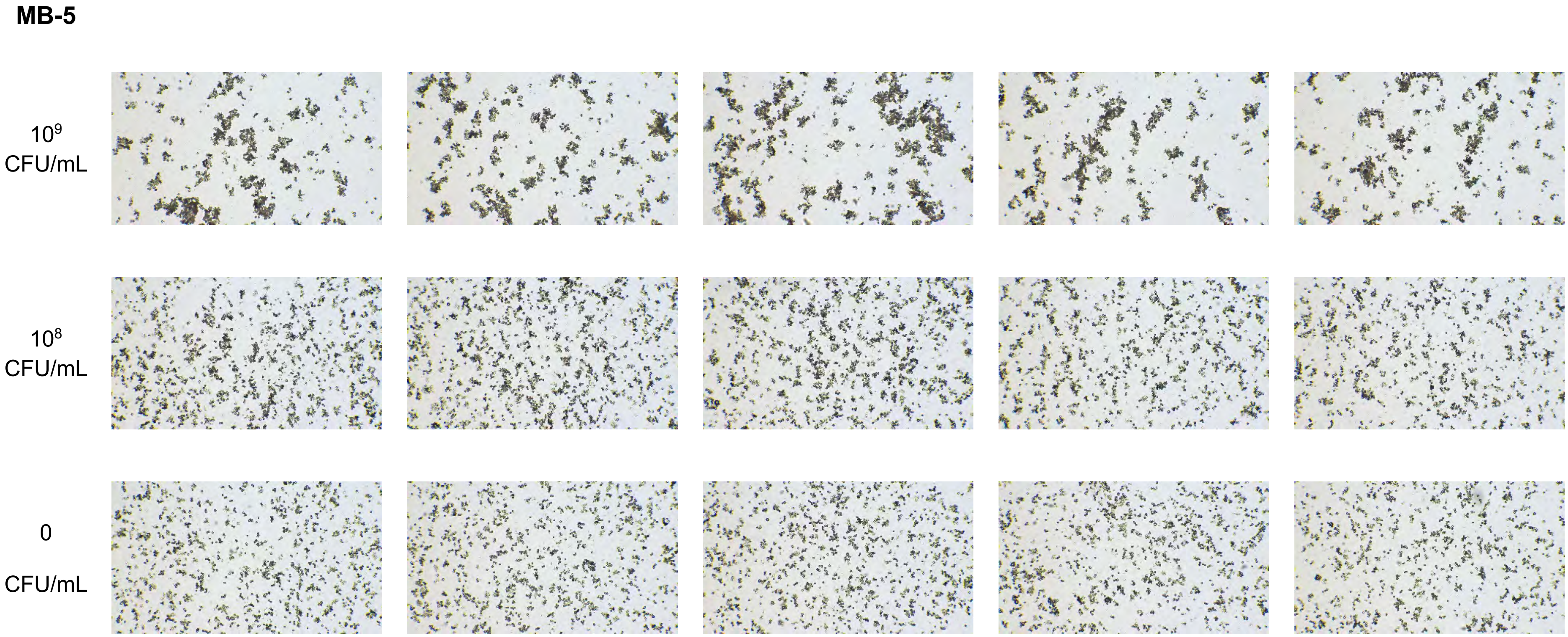

Figure S2. Representative brightfield microscope images (10× magnification) of **MB-5** incubated with *S. aureus* (10^9 & 10^8 CFU/mL) in PBS and in PBS only as a control (0 CFU/mL)

MB-11

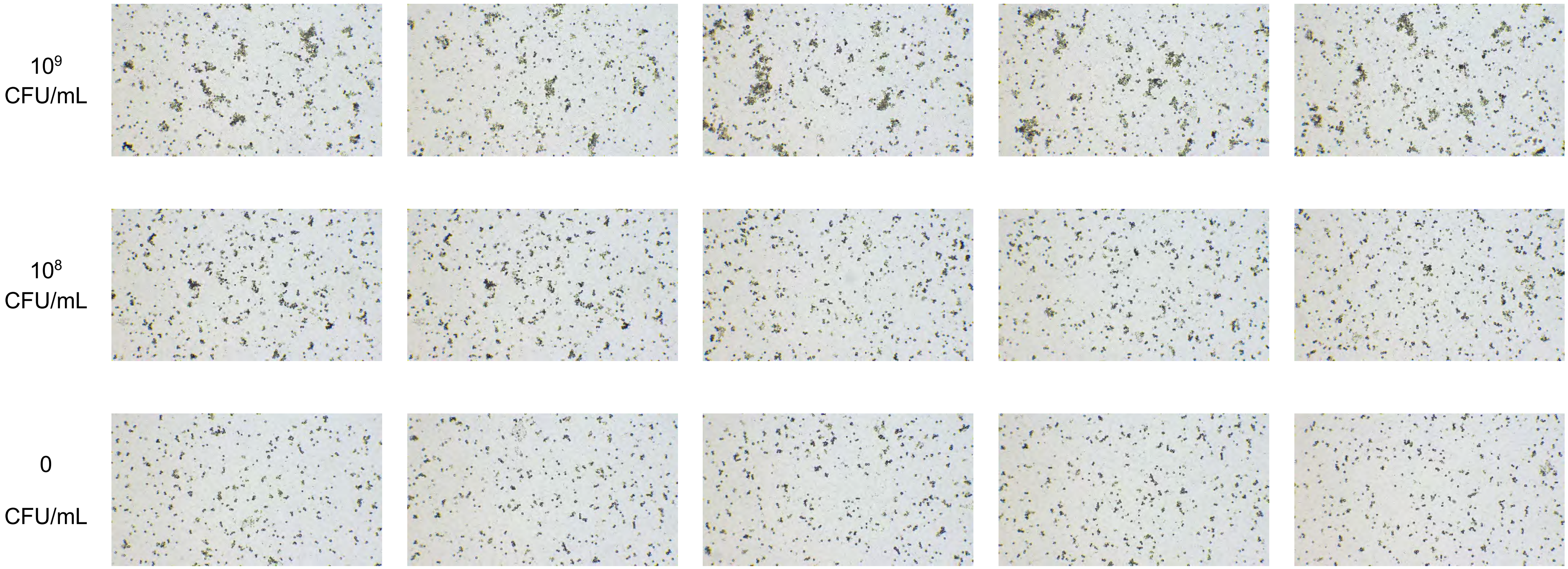

Figure S3. Representative brightfield microscope images (10× magnification) of **MB-11** incubated with *S. aureus* (10^9 & 10^8 CFU/mL) in PBS and in PBS only as a control (0 CFU/mL)

Image Processing and data analysis: All images collected were processed by Abacus, a Python-based image-processing software developed by FluoretiQ Ltd.[2] The software detects the number of beads and clusters per image and calculates the cluster-to-bead ratio (CBR) by dividing the total number of beads in clusters over the total number of isolated beads present in each image.

$$\boldsymbol{Cluster\ to\ Bead\ Ratio\ (CBR)} = \frac{\boldsymbol{Number\ of\ Beads\ in\ Clusters}}{\boldsymbol{Number\ of\ Isolated\ Beads}}$$

The CBR ratios for all images taken for each bacterial concentration and microbead conjugation were then visualized as box and whisker plots using GraphPad Prism v9.4.0.

For comparative analysis the CBR was routinely normalized through subtraction of the mean CBR of the control sample (4% microbeads incubated in sterile PBS only) from all CBR values for a given probe.

Normalized CBR signifies an analogous normalization procedure where all datapoints for a given probe were divided by the mean CBR of the control sample (defined above).

Cu(I) Agglutination Control Experiment

To ensure no residual Cu(I) from the conjugation reaction could affect the bacterial agglutination assay, MB-Alkyne was resubjected to the same coupling conditions ($CuSO_4$, Na ascorbate, overnight shaking) used to conjugate the azido-Araf derivatives albeit in the absence of the azido partner, and then washed using the usual procedure. These microbeads were then incubated with *S. aureus* and

showed no significant agglutination compared to the untreated MB-Alkyne only control (Figure S6), confirming that any observed agglutination could be attributed to the functionalized-probes.

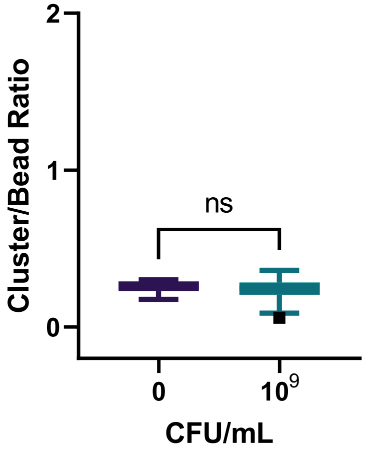

Figure S4. CBR for **MB-Alkyne** subjected to CuAAC coupling conditions in the absence of the azido coupling partner, then incubated for 1 h with *S. aureus* Newman (10^9 CFU/mL in PBS) at 21°C in PBS.

Agglutination Data for all Functionalized Microbeads incubated with *S. aureus* Newman.

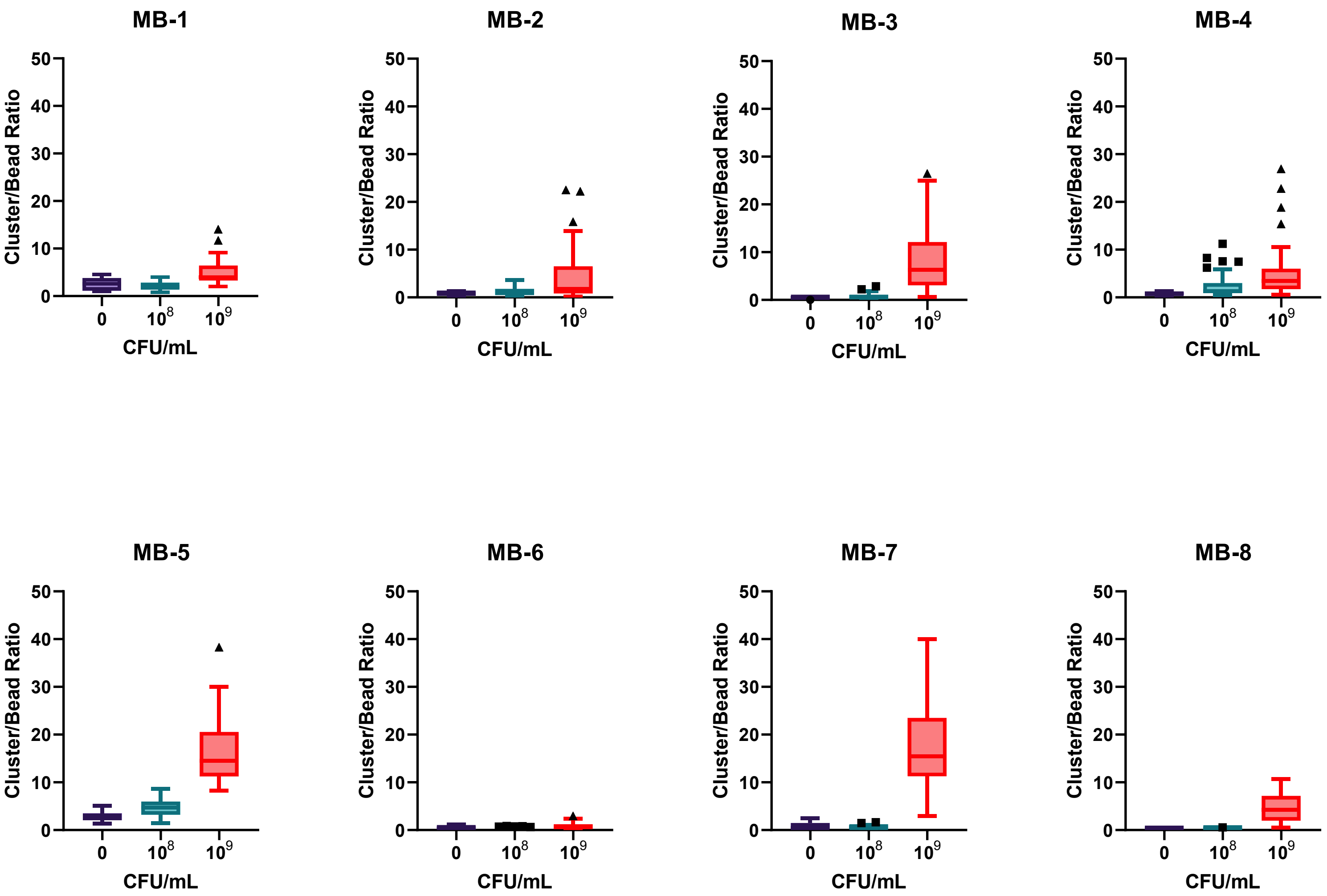

Figure S5A. CBR ratio for **MB-1 – MB-8** upon 1h incubation with *S. aureus* (10^9 and 10^8 CFU/mL in PBS) at 21°C.

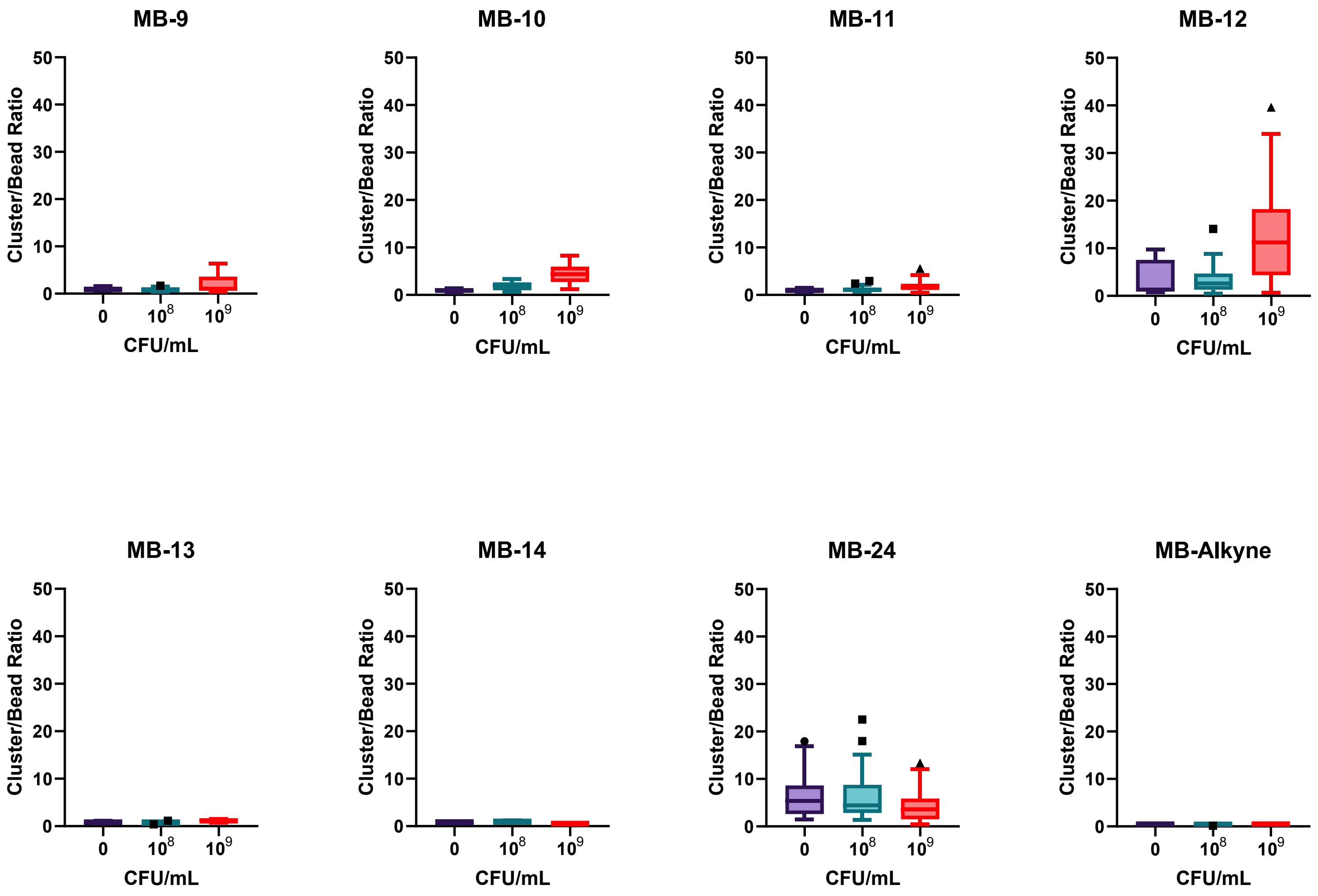

Figure S5B. CBR ratio for **MB-9 – MB14, MB-24, MB-Alkyne,** upon 1h incubation with *S. aureus* (10^9 and 10^8 CFU/mL in PBS) at 21°C.

Agglutination Data for MB-5 Bacteria Screen:

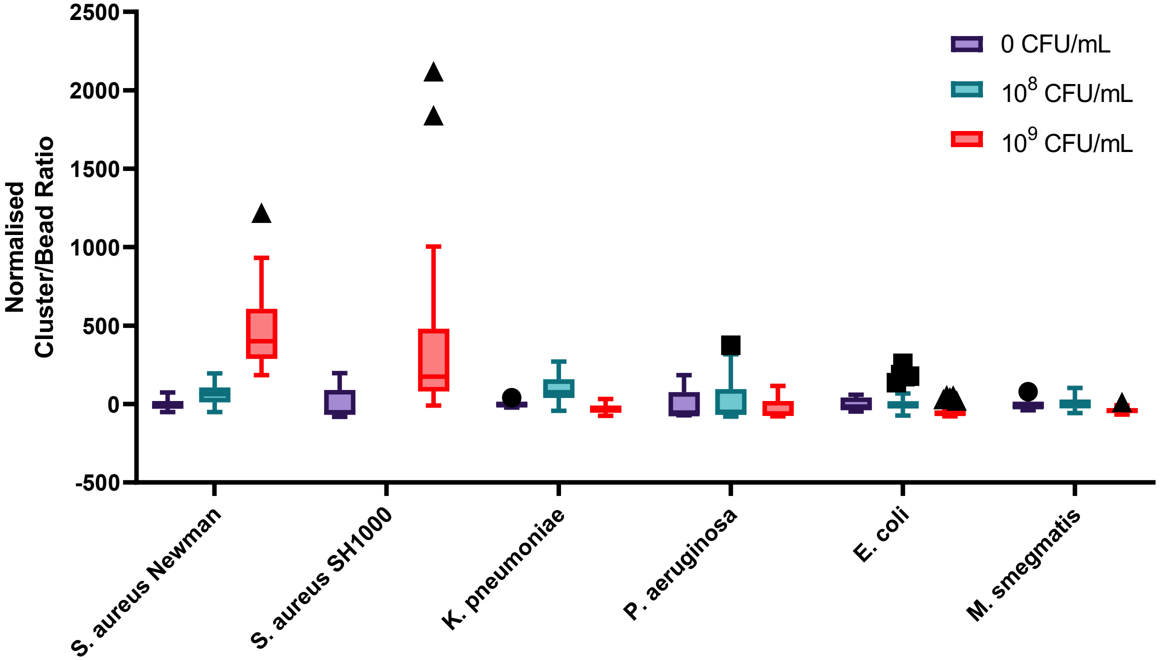

Figure S6. CBR ratio for **MB-5** after 1h incubation at 21°C with *S. aureus* Newman and SH1000, Gram-positive *M. smegmatis* mc(2)155 and Gram-negative *E. coli* BW25113, *P. aeruginosa* PA01 and *K. pneumoniae* NCTC 5055, respectively at 10^9 and 10^8 CFU/mL in PBS and 21°C, compared to controls of **MB-5** in sterile PBS (0 CFU/mL) at 21°C.

Agglutination Assay (MB-7 with *S. aureus*) With Added External D-Arabinose

MB-7 was incubated with 10^9 CFU/mL *S. aureus* in PBS as described above (1 h, 1600 rpm, 21°C), with the addition of 10 uL D-arabinose stock solution in PBS, or just PBS (0 eq.) as a control. Four concentrations of D-ara in PBS were prepared by serial dilution: 1 mM (10,000 eq.), 10 uM (1000 eq.), 100 nM (100 eq.) and 1 nM (10 eq.). The solutions were then deposited onto microscope slides, imaged and the CBR calculated as previously outlined.

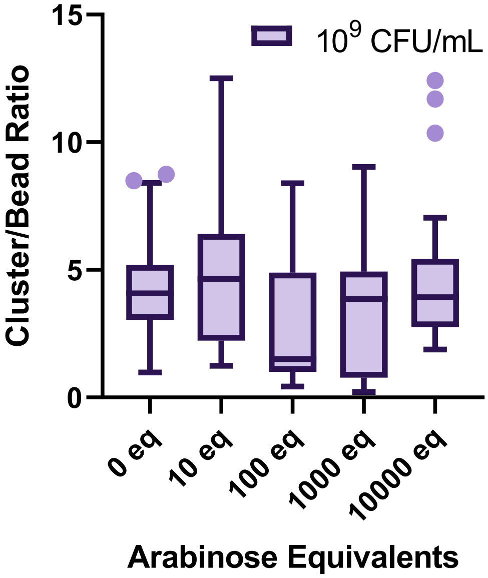

Figure S7. Agglutination of **MB-7** (incubated with *S. aureus* Newman, 10^9 CFU/mL in PBS) in the presence and absence of an excess of free arabinose (up to 10,000 equivalents with respect to the moles of immobilized arabinose derivative on the microbeads).

Lower Arabinose Functionalization on Microbead Surface Impacts Agglutination

A separate batch of microbeads functionalised with 2-deoxy-2-azido arabinose **5** was prepared, using lower equivalents (20 vs 400) of the azido-sugar and the CuAAC coupling reagents: To a suspension of **MB-Alkyne** in PBS (125 µL, 4% w/v, 8.3 nmol propargyl functionality), 2-deoxy-2-azido arabinose **5** (0.17 µmol), ascorbic acid sodium salt (1.06 mg, 4.5 µmol) and $CuSO_4.5H_2O$ (0.26 mg, 0.87 µmol) were added. The reaction mixture was shaken at 900 rpm for 24 h. Subsequently the reaction mixture was transferred to a 100kDa centrifuge filter and centrifuged (8000 rpm, 10 min). The residue was washed with distilled water (125 µL, twice), MeOH (125 µL) and distilled water (125 µL) again and then re-suspended in sterile PBS (125 µL) to provide a 4% w/v suspension of conjugated beads (**MB-5 (20 eq.)**).

MB-5 (20 eq.) was then tested for surface functionalisation using the azido-coumarin protocol outlined above. An increase in fluorescence was observed for **MB-5 (20 eq.)** after reaction with the azido coumarin, whereas no increase was observed with the original **MB-5 (400 eq.)**, suggesting that unreacted alkynes are present on the microbead surface in **MB-5 (20 eq.),** and therefore a lower amount of arabinose derivative is conjugated, potentially explaining the absence of agglutnation of **MB-5 (20 eq.)** when incubated with *S. aureus*.

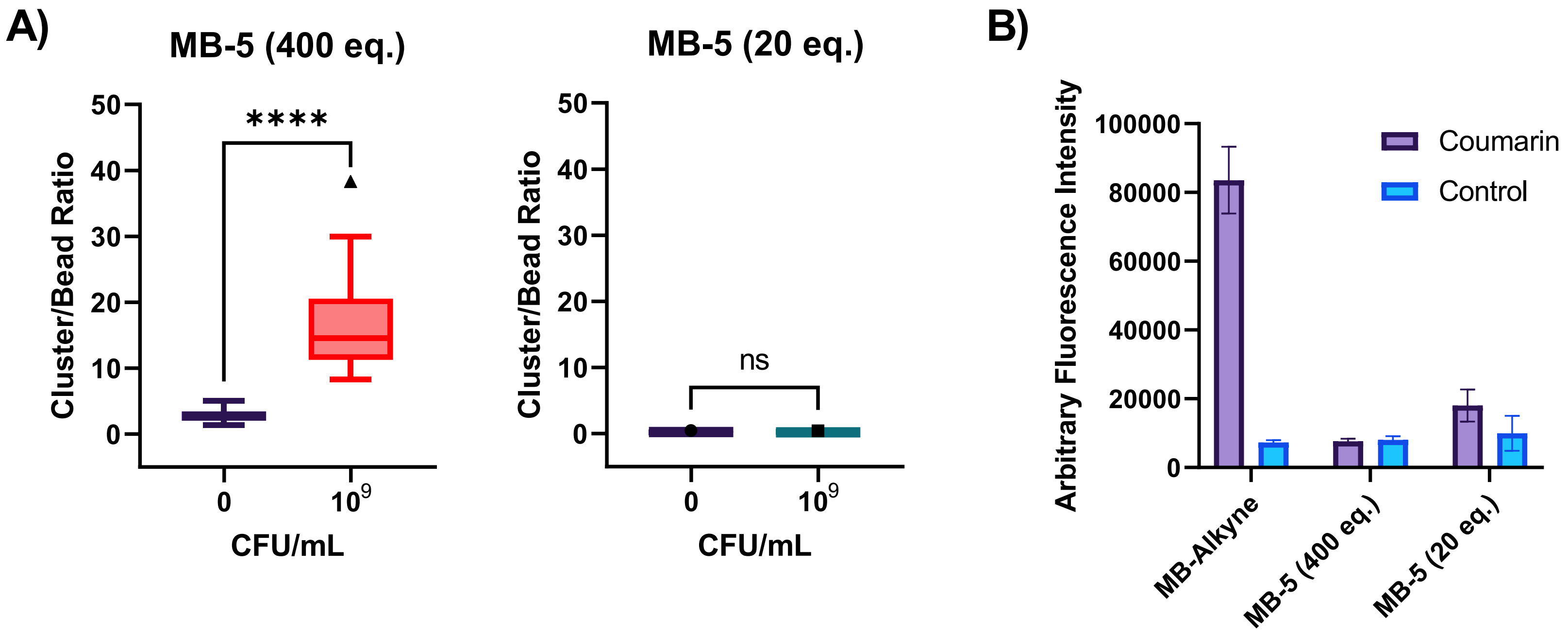

Figure S8. A) CBR for microbeads functionalized with 400 equivalents (**MB-5 400 eq.**) and 20 equivalents (**MB-5 20 eq.**) of 2-azido arabinose derivative **5**, then incubated with *S. aureus* (10^9 CFU/mL) in PBS or in PBS only (0 CFU/mL) at 21°C for 1h. **B)** Fluorescence intensity (460 nm emission) of functionalized microbeads **MB-5 400 eq.**, **MB-5 20 eq.** and **MB-Alkyne** after subjection to CuAAC conjugation conditions in the presence (Coumarin) and absence (Control) of 3-azido-7-hydroxycoumarin.

B] Biofilm Disruption Assay

S. aureus SH1000 overnight cultures were diluted in LB broth supplemented with 1% glucose to a final concentration of 1 x 10^6 CFU/mL. 100μl of bacterial suspension and the blank (LB+glucose only) were aliquoted in a 96-wells plate (16 wells each), ensuring 8 technical replicates per each condition. The plate was incubated at 37°C for 24 h with 5% CO_2 to promote biofilm formation. After this time, the broth was removed, and the wells were rinsed twice with 100 μL of sterile PBS to remove planktonic cells. Then, the wells were air-dried at 37°C for 1 h. After that, 8 wells containing biofilm and 8 blank wells (previously containing LB+1% glucose only) were filled with 100μl of 6% beads (suspension in PBS) and the remaining wells (8 containing biofilm and 8 blank) were filled with PBS as control. The plate was incubated at 37°C for 24 h. After this time, the wells were emptied, rinsed twice with 100 μL of sterile PBS and air-dried at 37°C for 1 h. 100 μL of 0.1% crystal violet (CV) solution was added to each well and, after 5 min, the CV solution was removed, and the wells rinsed twice with 100 μL PBS. The plate was air-dried for 1 h and the stained cells were solubilized in 150 μL of aqueous 30% acetic acid for 30 min. 20 μL of solution from each well was diluted in 180μl of 30% acetic acid into a new plate and absorbance at 595 nm was quantified using a plate reader. These data were routinely analyzed as a percentage change in absorbance (as a proxy for amount of biofilm) relative to an untreated *S. aureus* biofilm control (Figure S8). The experiment was repeated in biological triplicate.

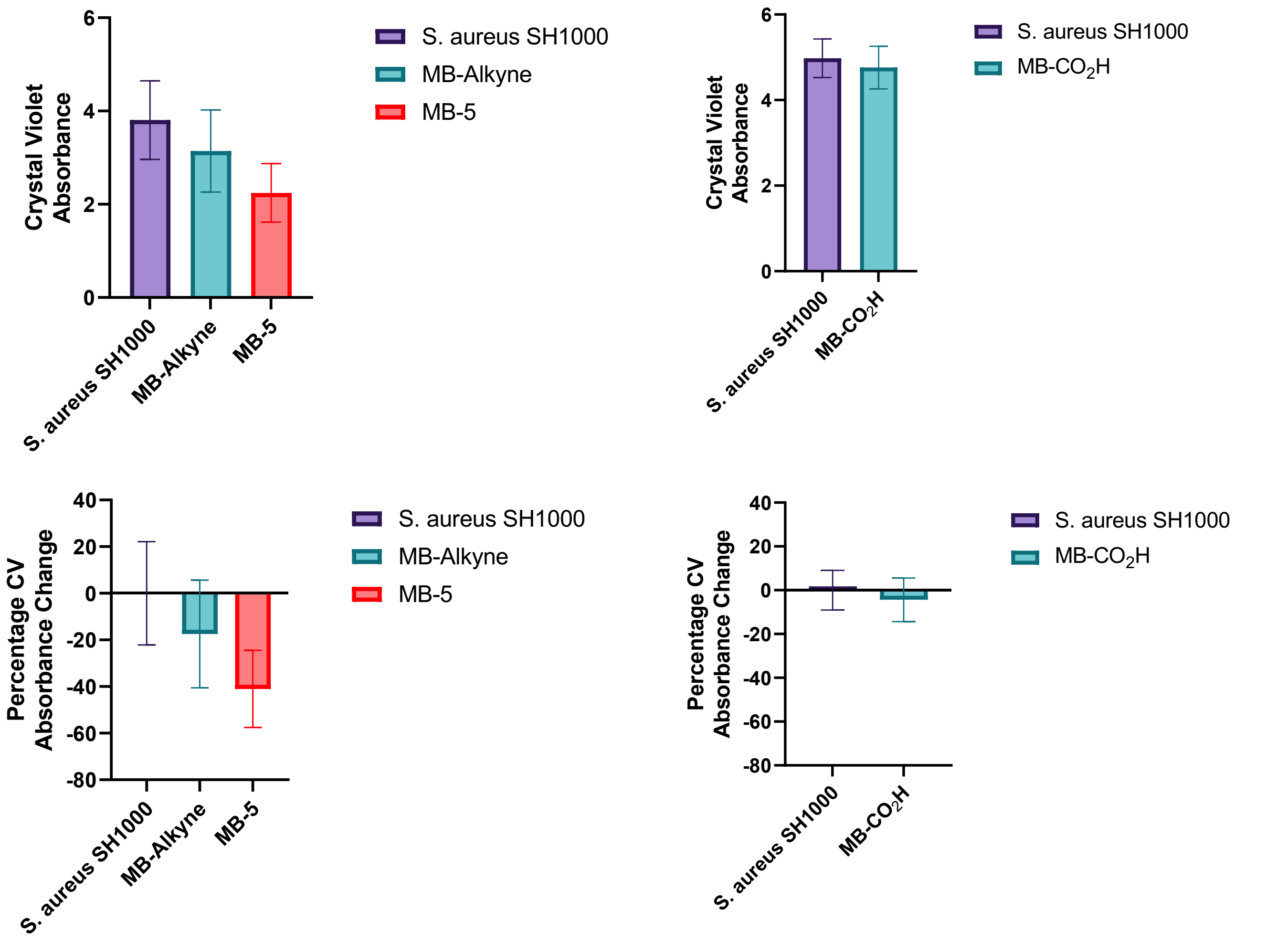

Figure S9. Absorbance (595 nm) of *S. aureus* SH1000 biofilms, untreated and after 24 h treatment with 6% w/v microbeads (**MB-5**, **MB-Alkyne** and **MB-CO_2H**), washing and staining with Crystal Violet. Data also reported as percentage change in absorbance relative to untreated biofilm.

Effect of D-arabinose, L-arabinose and 2-deoxy-2-azido-D-arabinose on *S. aureus* Biofilms.

The effect of free arabinoses and azido-arabinose derivatives on *S. aureus* SH1000 biofilms was assessed using the assay described above. After formation for 24 h, biofilms were rinsed, dried and solutions of D-arabinose, L-arabinose and 2-deoxy-2-azido-D-arabinose in LB-broth at concentrations of 2 mM, 200 μM and 20 μM were carefully introduced. The biofilms were treated for a further 24 h, then washed, stained and quantified as described (Figure S10).

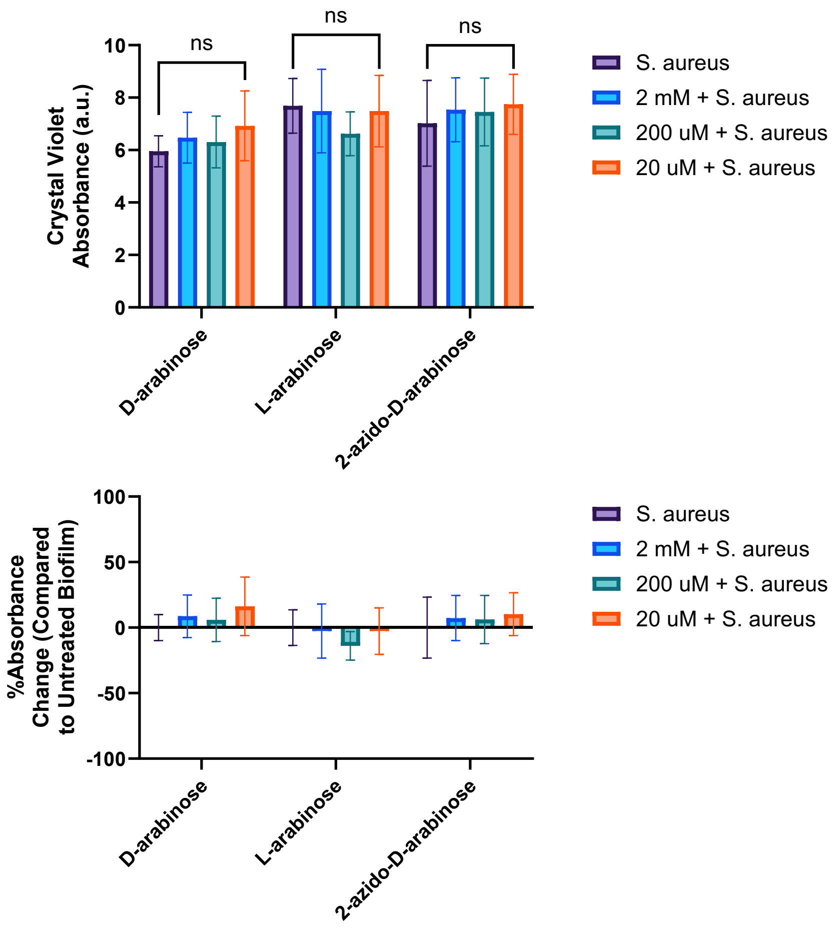

Figure S10. Absorbance (595 nm) of *S. aureus* SH1000 biofilms, untreated and after 24 h treatment with D-arabinose, L-arabinose and 2-deoxy-2-azido-D-arabinose at 2mM, 200μM and 20 μM followed by washing and staining with Crystal Violet. Data also reported as percentage change in absorbance relative to untreated biofilm.

References:

1) Shilei Zhu, Jingtuo Zhang, Giri Vegesna, Fen-Tair Luo, Sarah A. Green, Haiying Liu; *Org. Lett.* **2011**, 13, *3*, 438–441.
2) Jean-Baptiste Vendeville, Mathew John Kyriakides, Yuiko Takebayashi, Sylvain Rama, James Preece, Jenny Samphire, Javier Ramos-Soriano, Abraham Mauleon Amieva, Maisie Emma Holbrow-Wilshaw, Henry Ralph Gordon Newman, Sio Lou Kou, Sandra Medina-Villar, Neciah Dorh, Josephine Ndoa Dorh, James Spencer, M. Carmen Galan, *ACS Biomater. Sci. Eng*. **2022**, 8, 242−252.
3) Teodoro Garcia-Millan, Javier Ramos-Soriano, Mattia Ghirardello, Xia Liu, Cristina Manuela Santi, Jean-Charles Eloi, Natalie Pridmore, Robert L. Harniman, David J. Morgan, Stephen Hughes, Sean A. Davis, Thomas A. A. Oliver, Kathreena M. Kurian, M. Carmen Galan; *ACS Appl. Mater. Interfaces,* **2023**, 15, *38*, 44711–44721.

NMR Spectra:

^{1}H NMR Spectrum (400.34 MHz, $CDCl_3$) of **Compound 16**

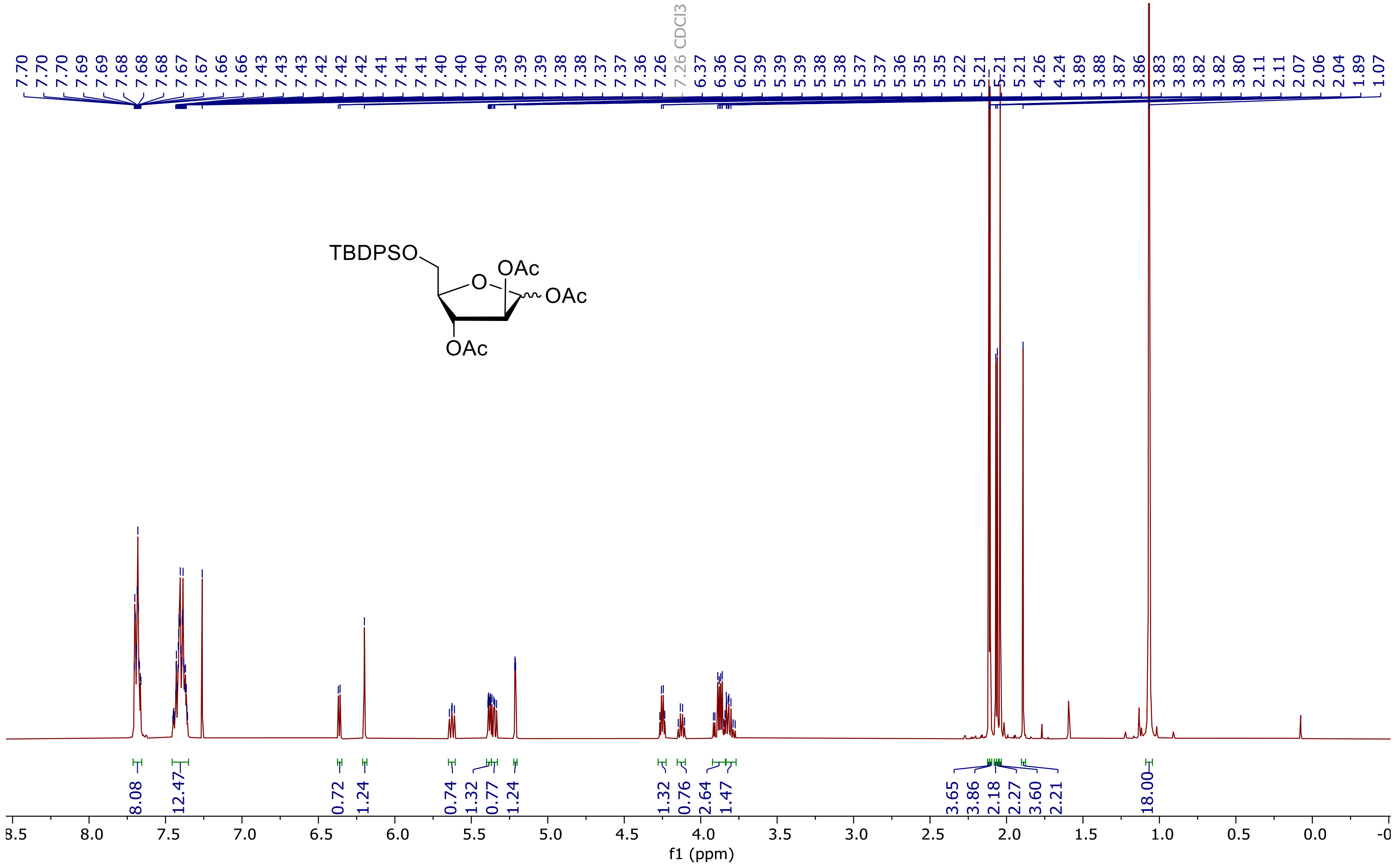

^{13}C NMR Spectrum (100.68 MHz, $CDCl_3$) of **Compound 16**

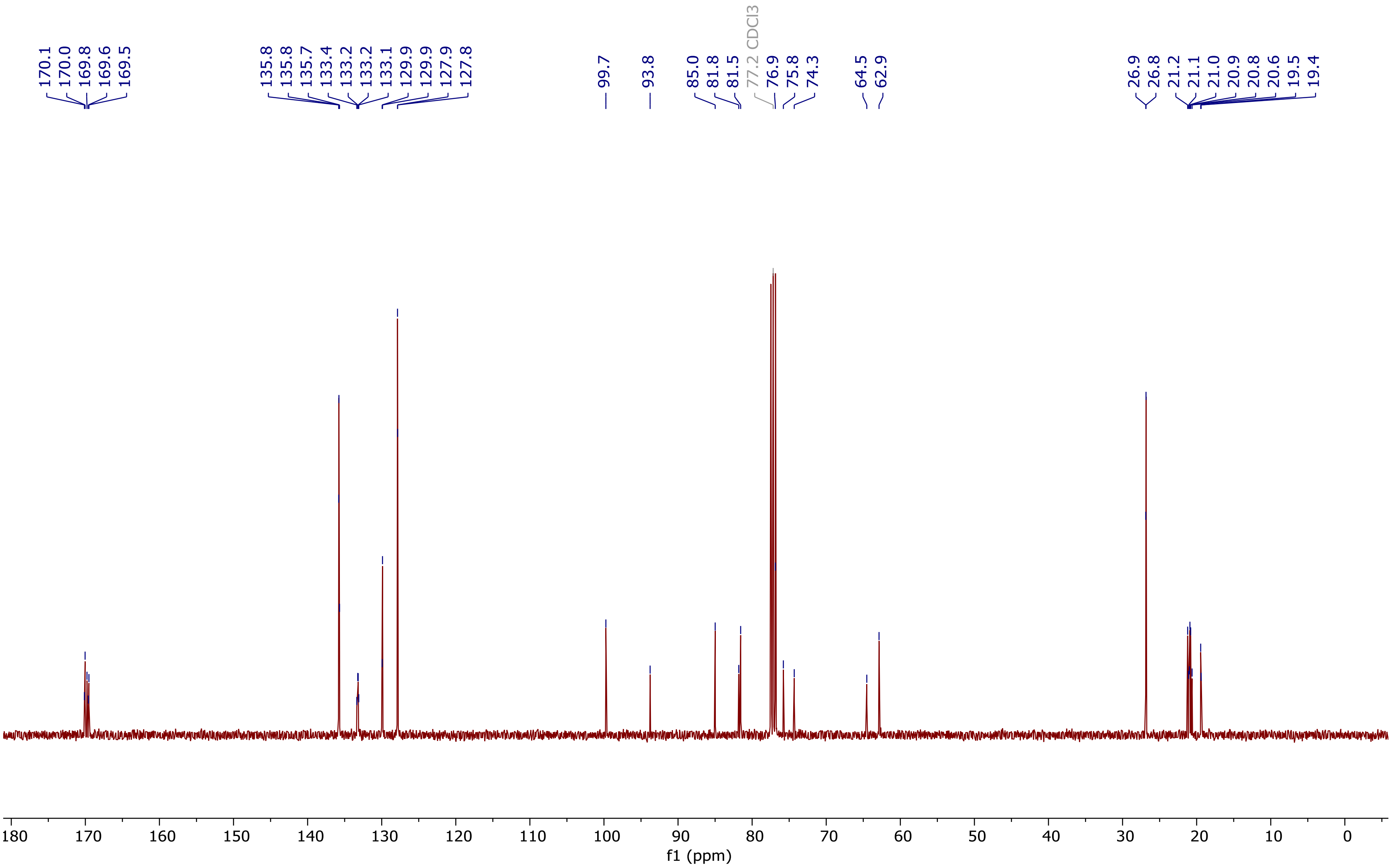

HSQC NMR Spectrum (400.34, 100.67 MHz, $CDCl_3$) of **Compound 16**

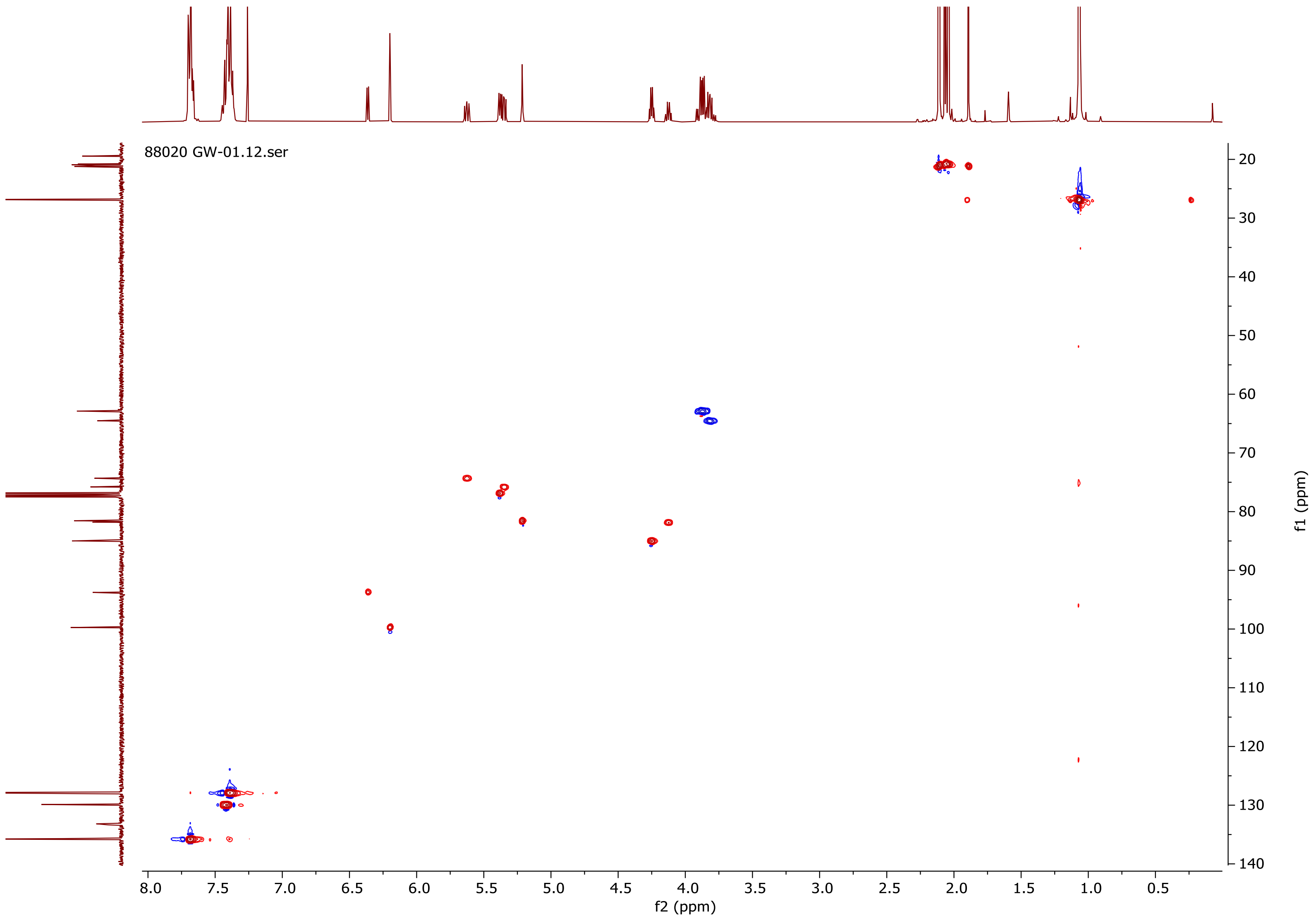

COSY NMR Spectrum (400.34 MHz, $CDCl_3$) of **Compound 16**

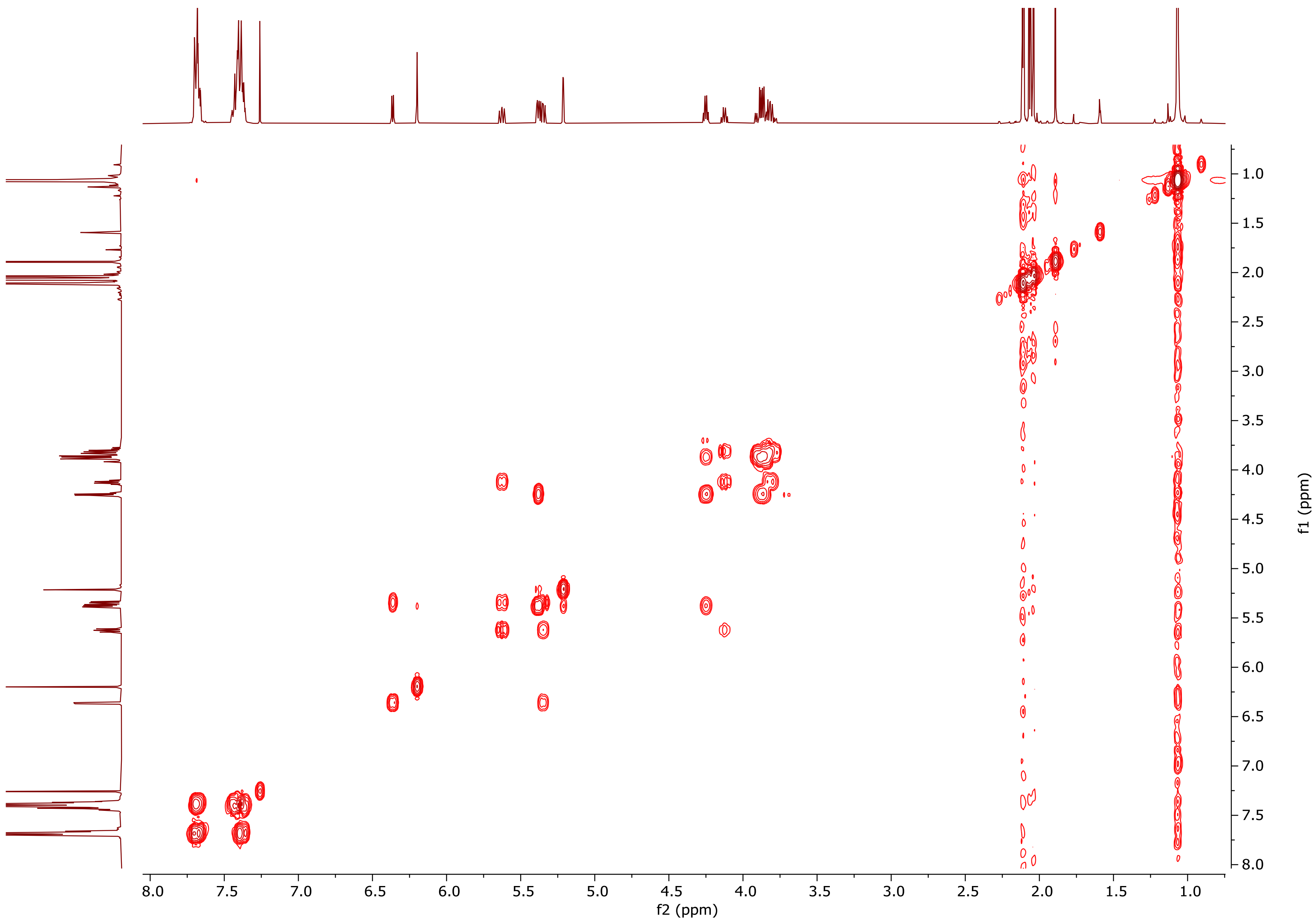

^{1}H NMR Spectrum (400.34 MHz, $CDCl_3$) of **Compound 17**

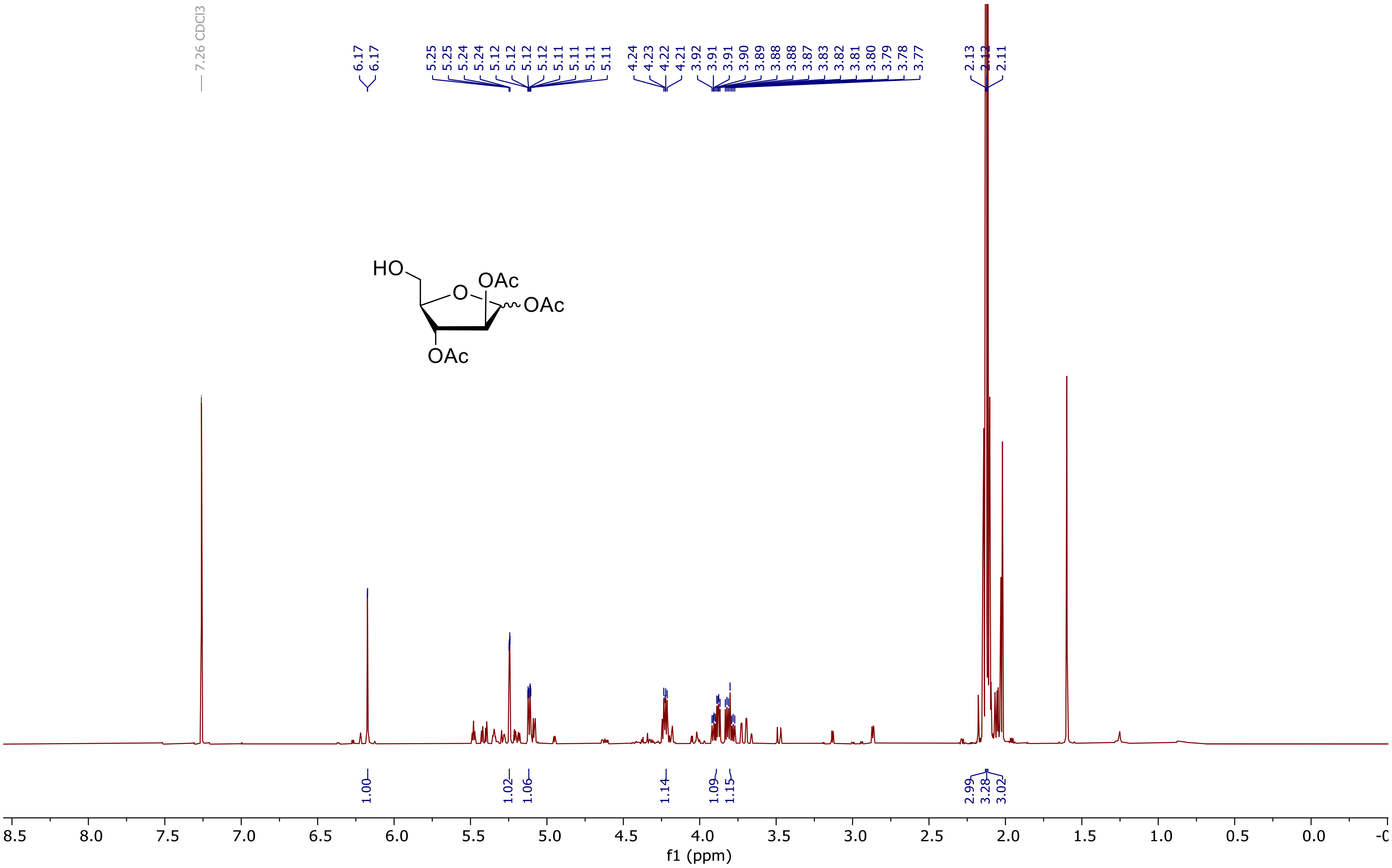

^{13}C NMR Spectrum (100.68 MHz, $CDCl_3$) of **Compound 17**

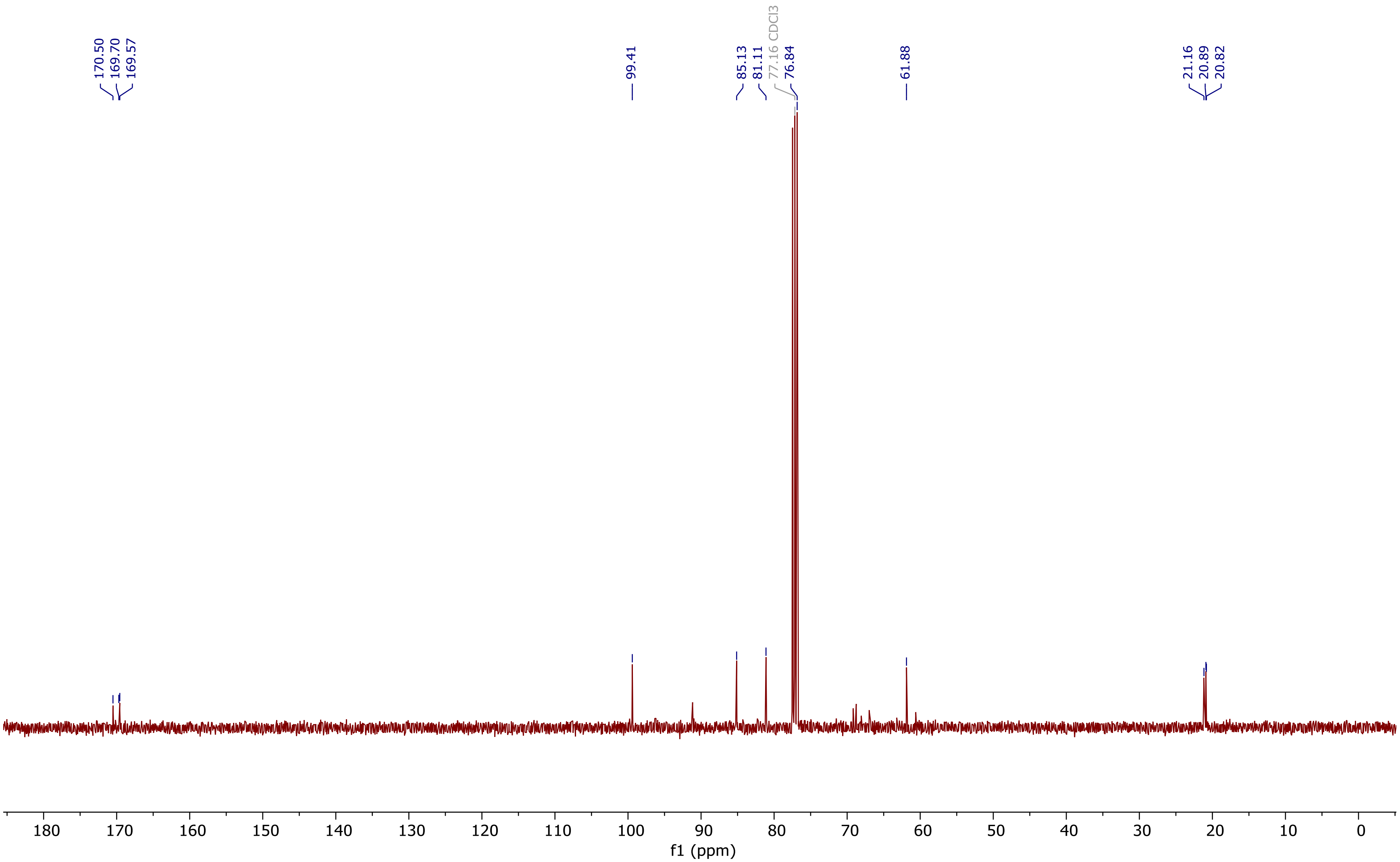

HSQC NMR Spectrum (400.34, 100.67 MHz, $CDCl_3$) of **Compound 17**

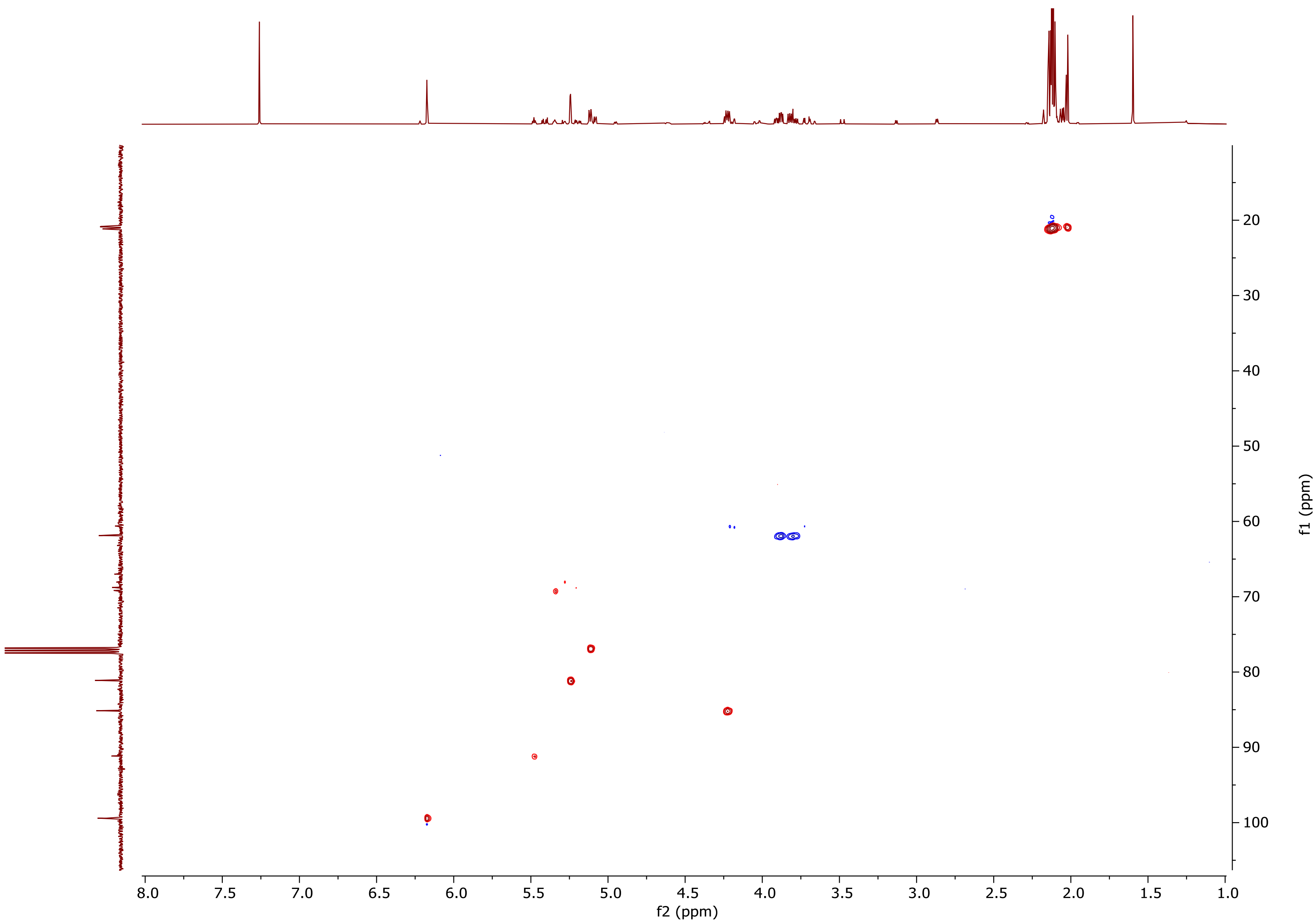

COSY NMR Spectrum (400.34 MHz, $CDCl_3$) of **Compound 17**

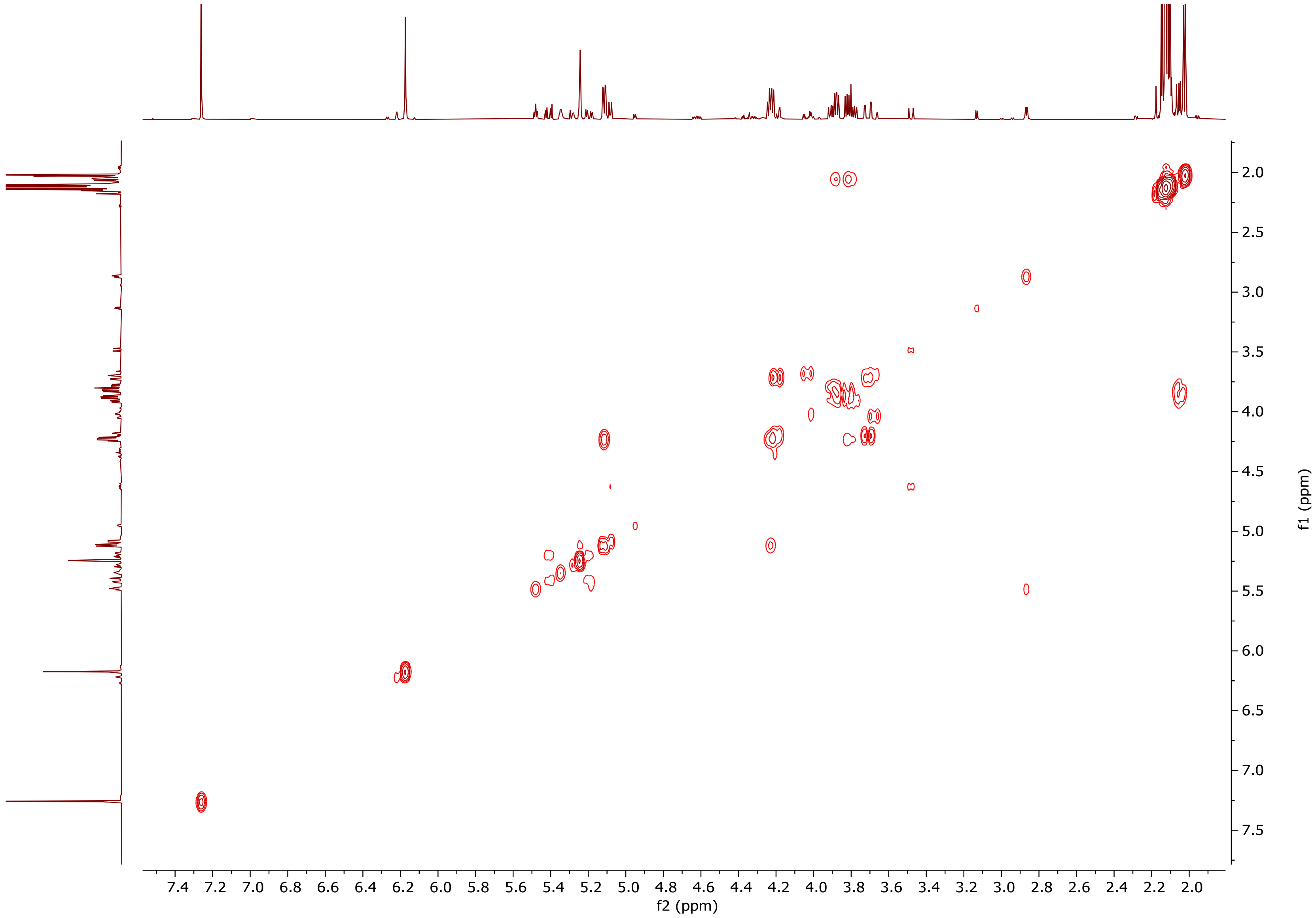

HMBC NMR Spectrum (400.34, 100.68 MHz, $CDCl_3$) of **Compound 17**

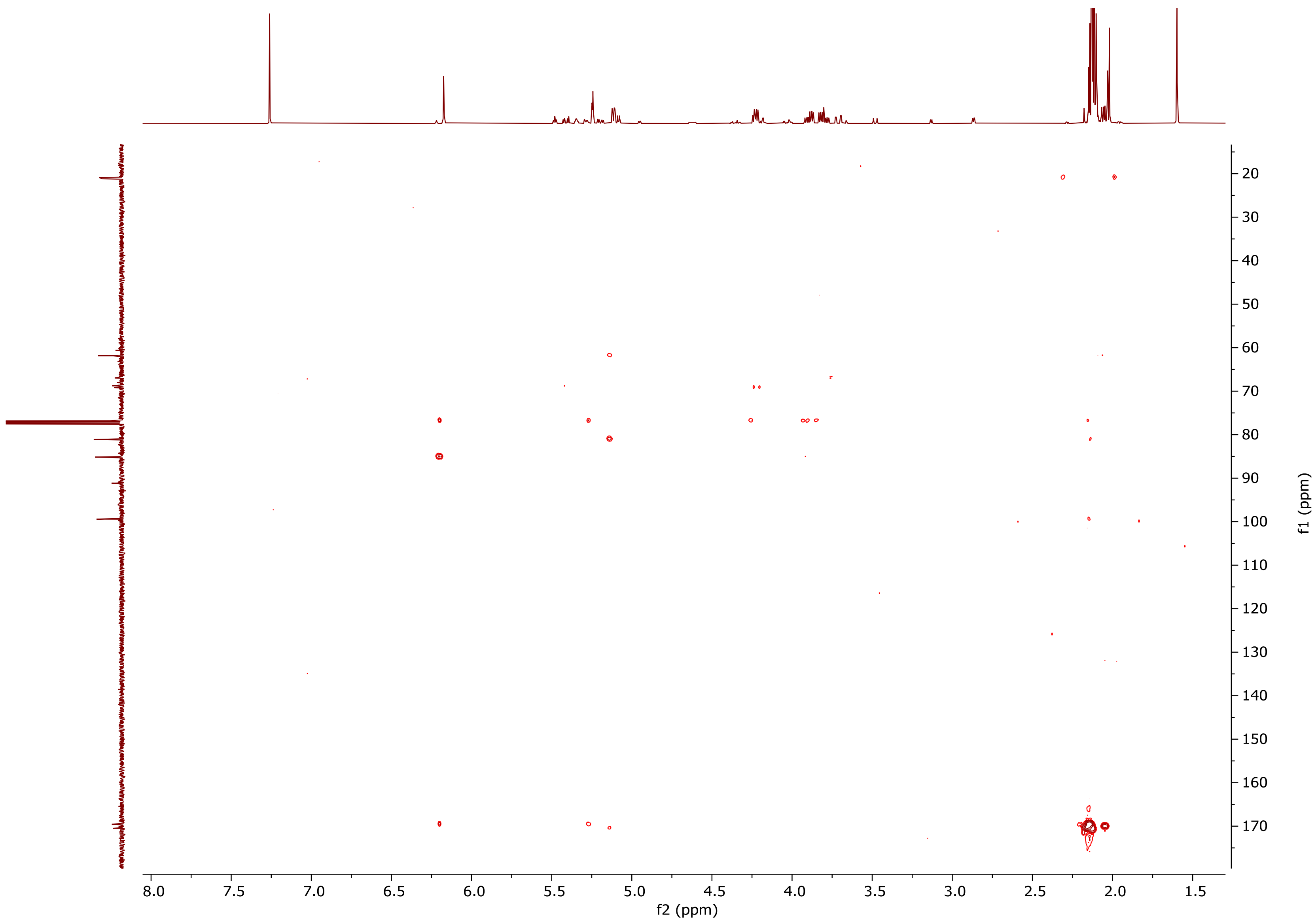

^{1}H NMR Spectrum (400.34 MHz, $CDCl_3$) of **Compound 2**

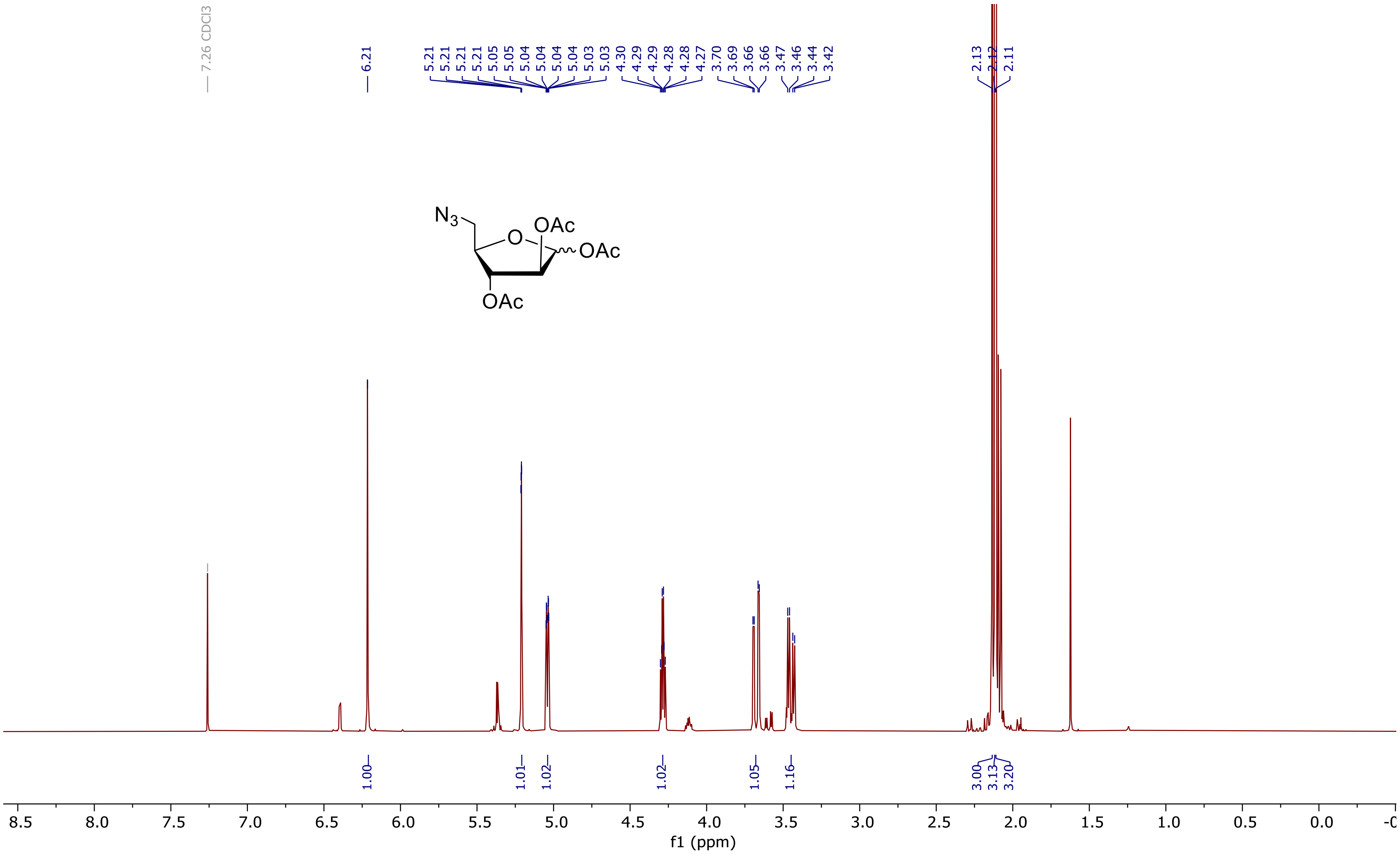

^{13}C NMR Spectrum (100.68 MHz, $CDCl_3$) of **Compound 2**

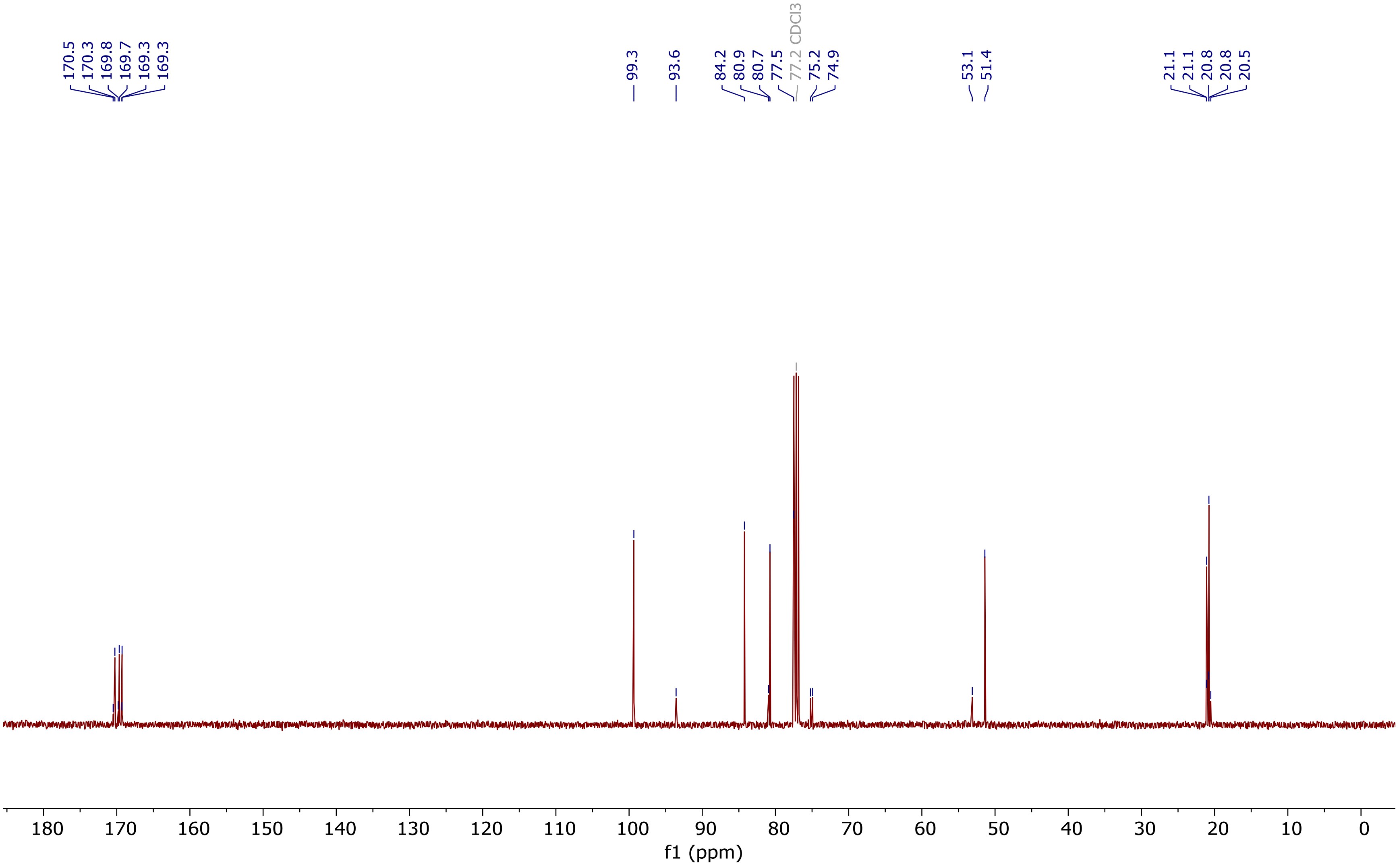

HSQC NMR Spectrum (400.34, 100.67 MHz, $CDCl_3$) of **Compound 2**

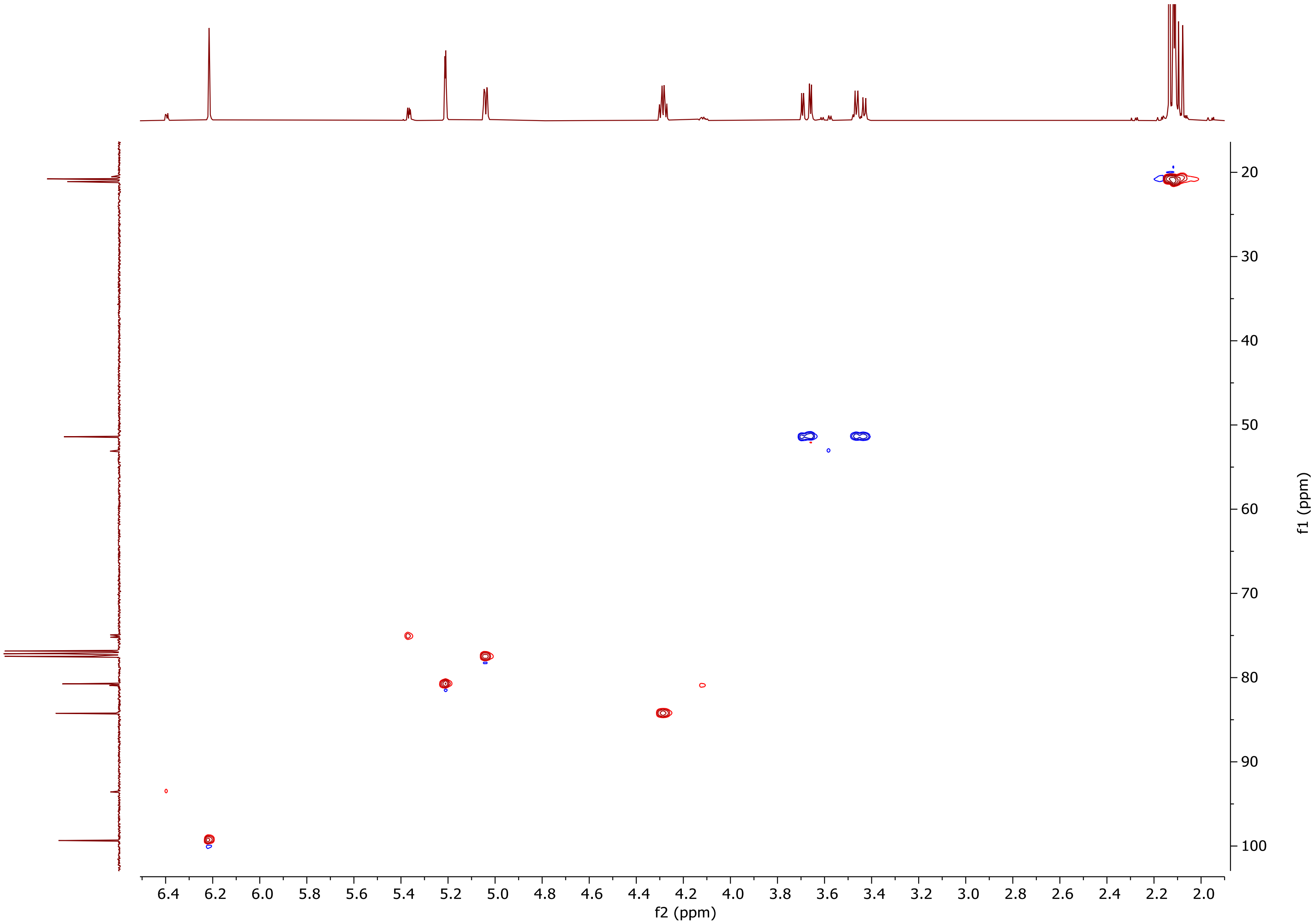

COSY NMR Spectrum (400.34 MHz, $CDCl_3$) of **Compound 2**

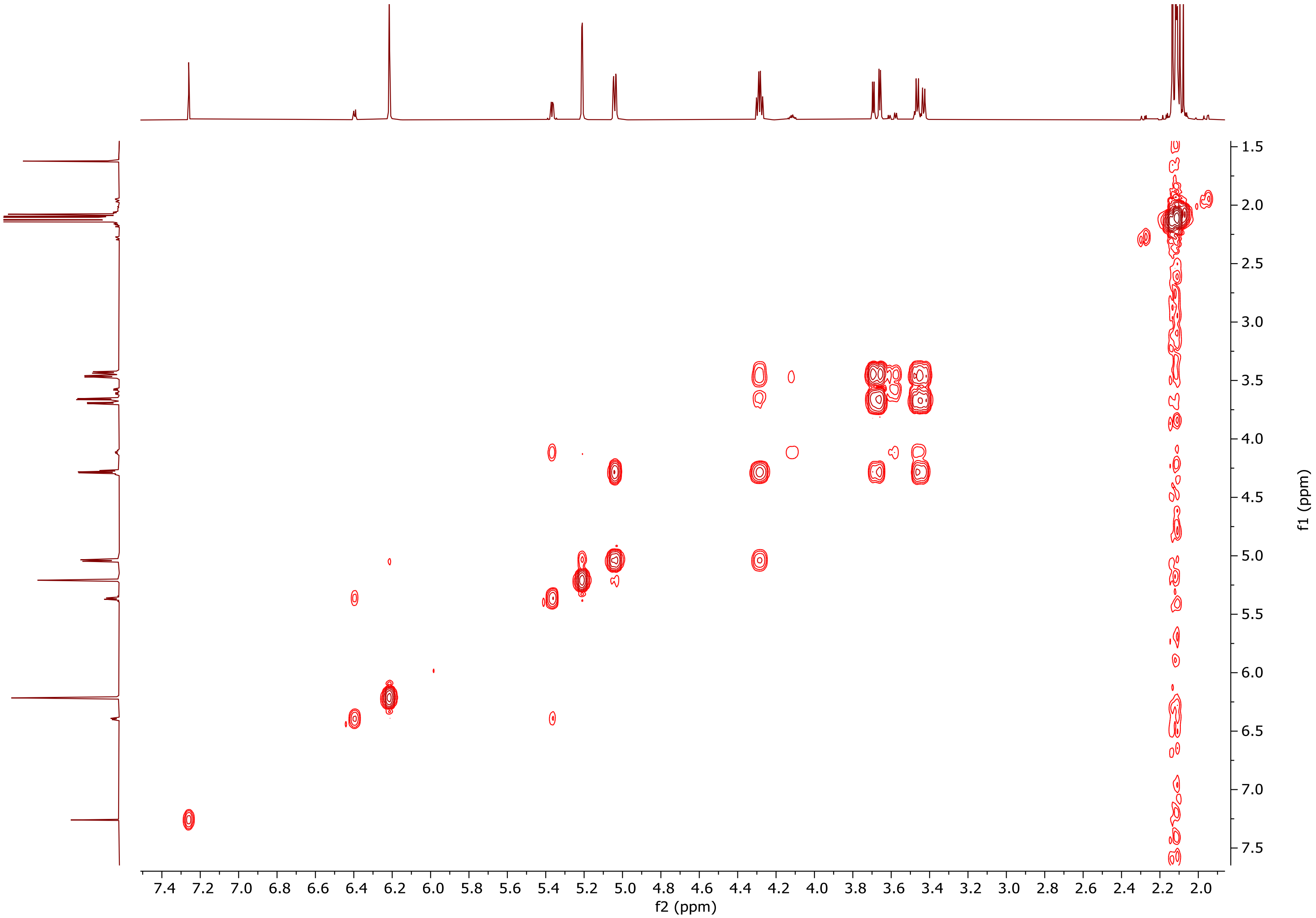

HMBC NMR Spectrum (400.34, 100.68 MHz, $CDCl_3$) of **Compound 2**

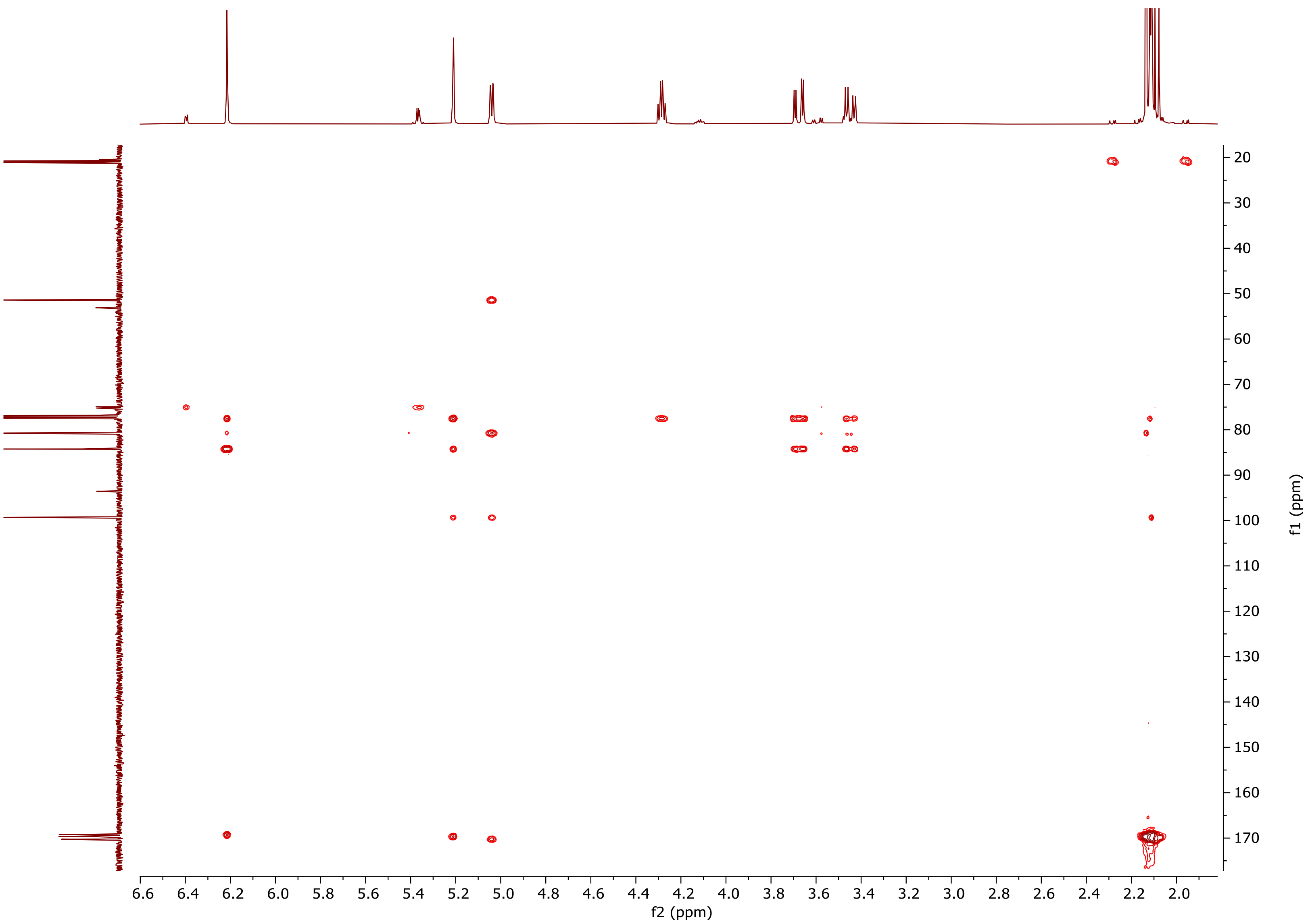

^{1}H NMR Spectrum (400.34 MHz, $CDCl_3$) of **Compound 19**

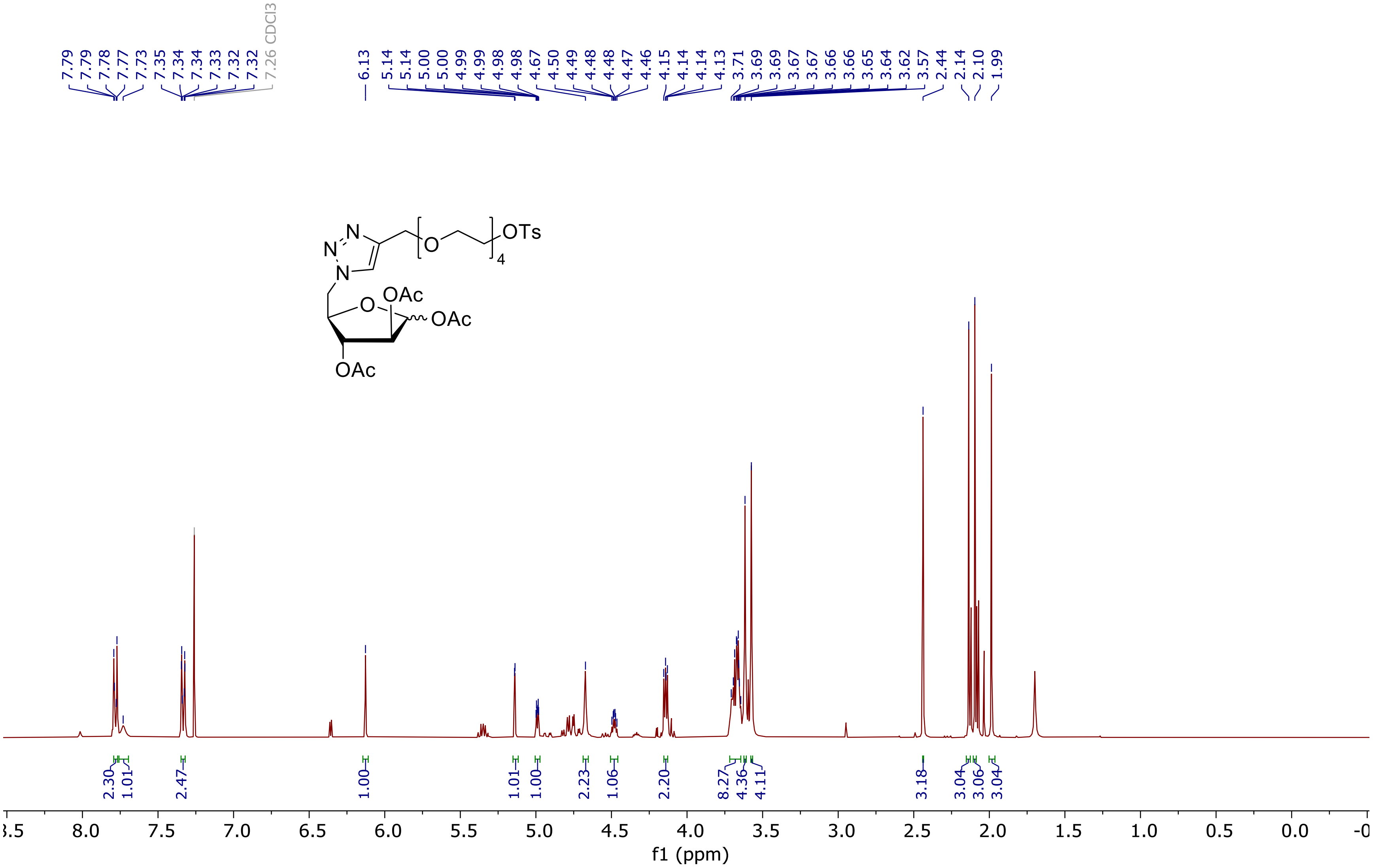

^{13}C NMR Spectrum (100.68 MHz, $CDCl_3$) of **Compound 19**

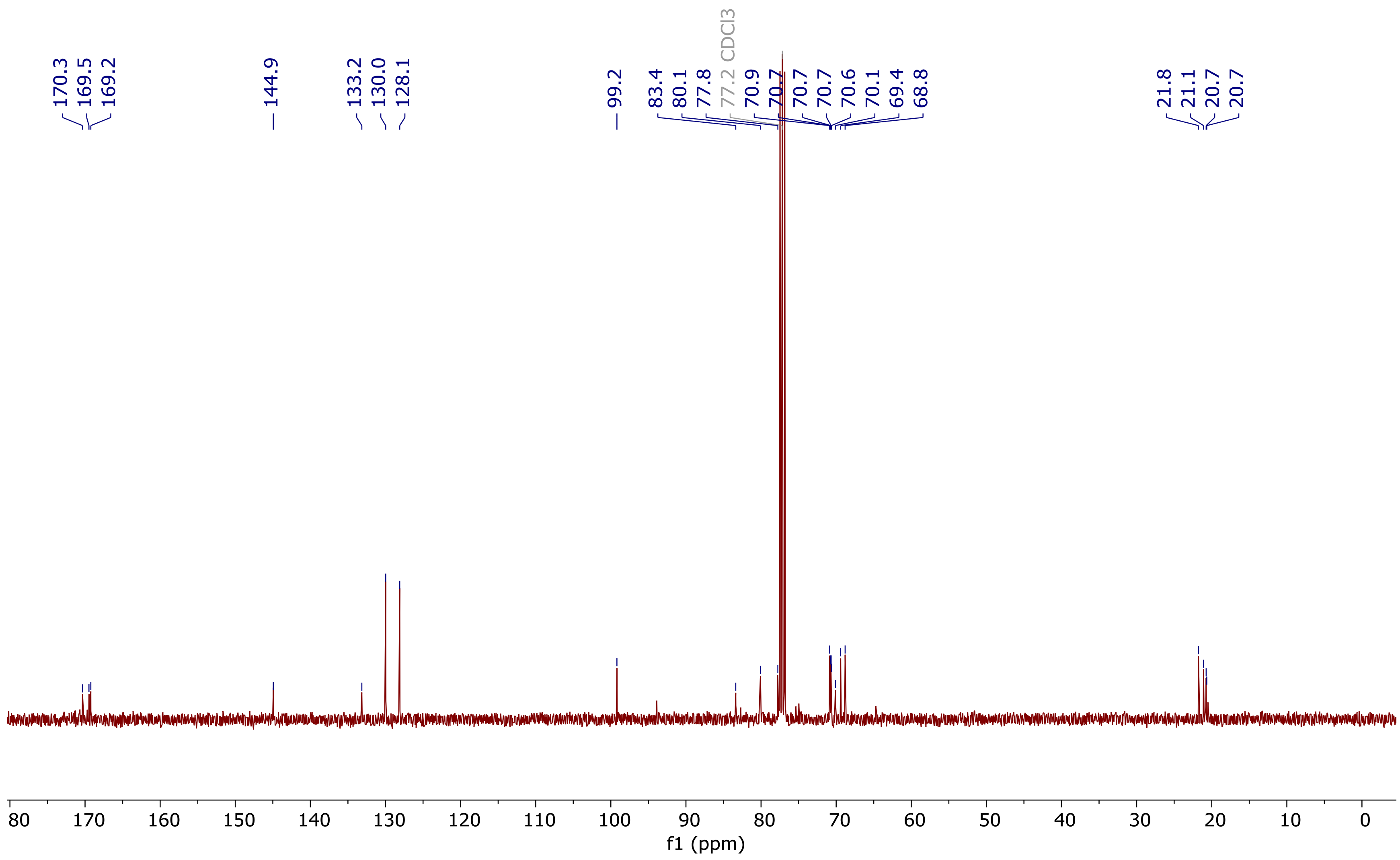

HSQC NMR Spectrum (400.34, 100.67 MHz, $CDCl_3$) of **Compound 19**

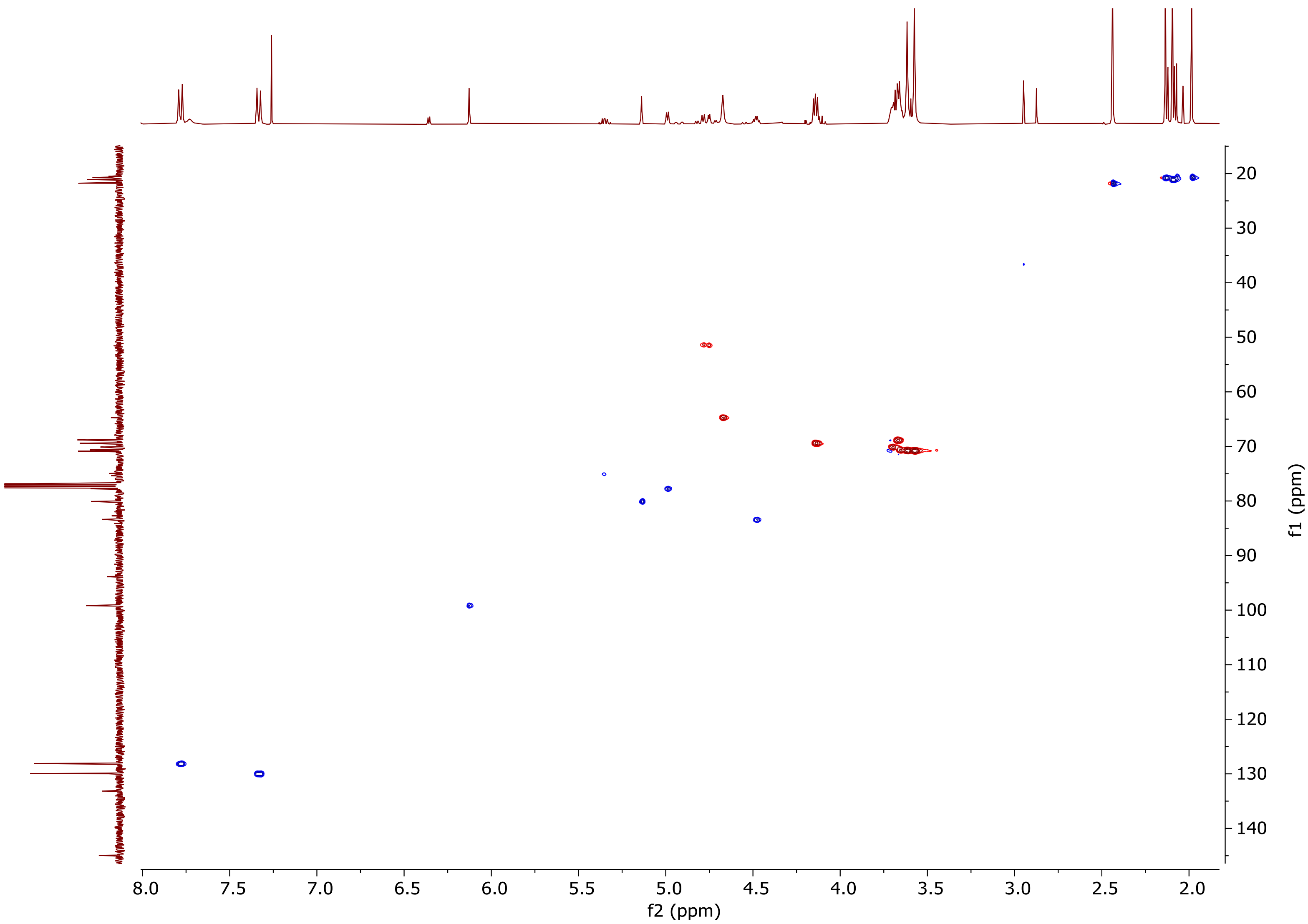

COSY NMR Spectrum (400.34 MHz, $CDCl_3$) of **Compound 19**

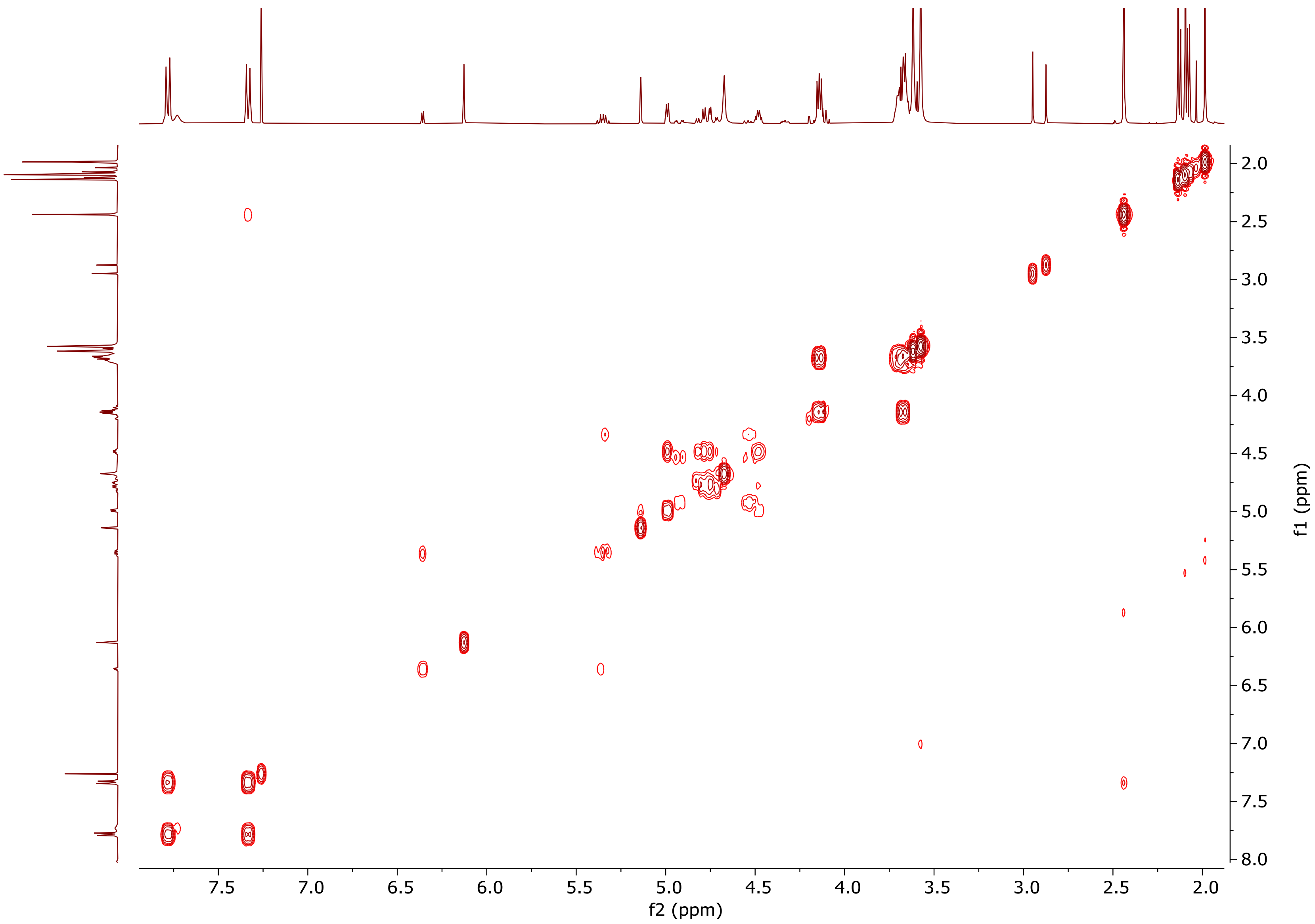

HMBC NMR Spectrum (400.34, 100.68 MHz, $CDCl_3$) of **Compound 19**

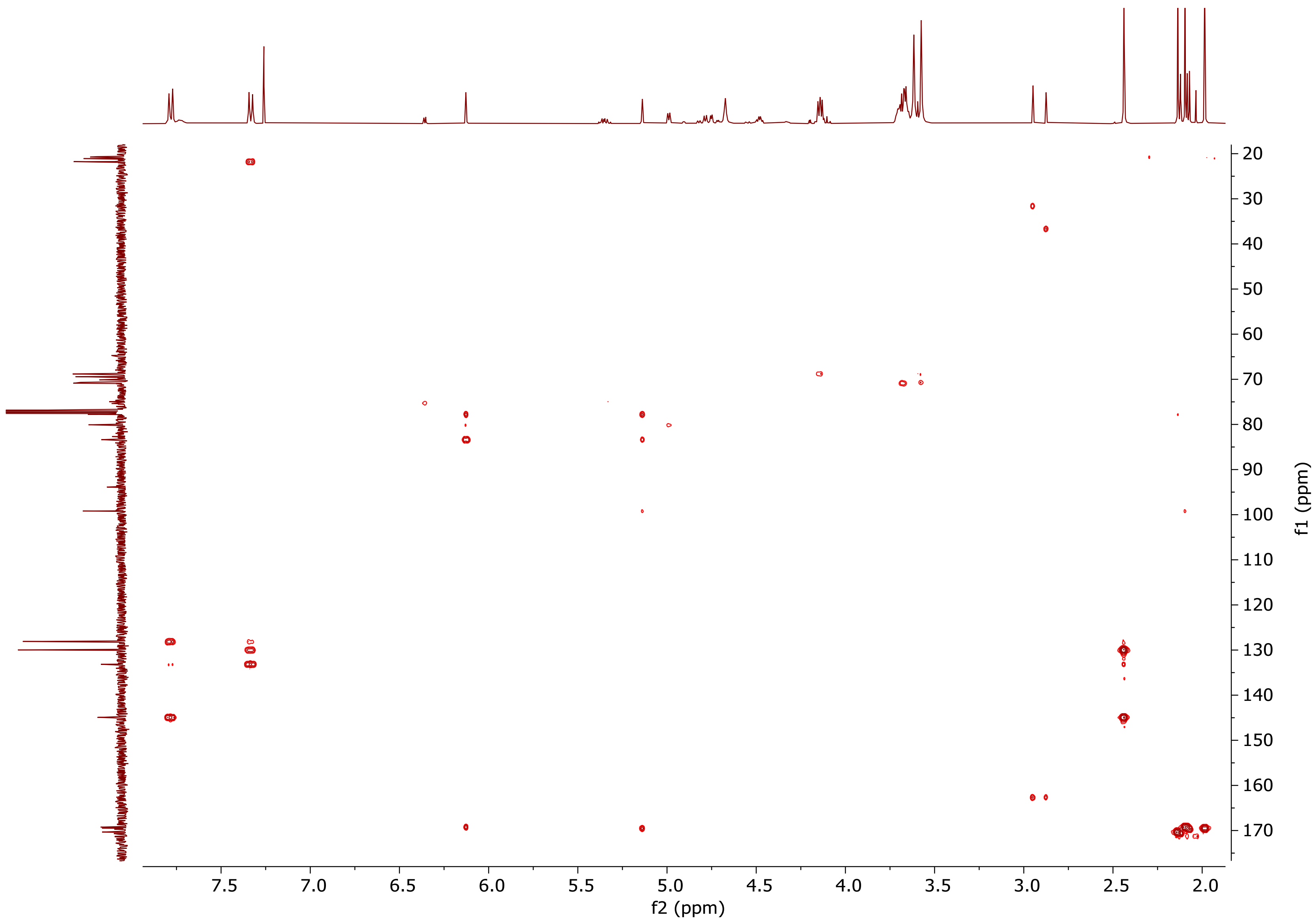

^{1}H NMR Spectrum (400.34 MHz, $CDCl_3$) of **Compound 4**

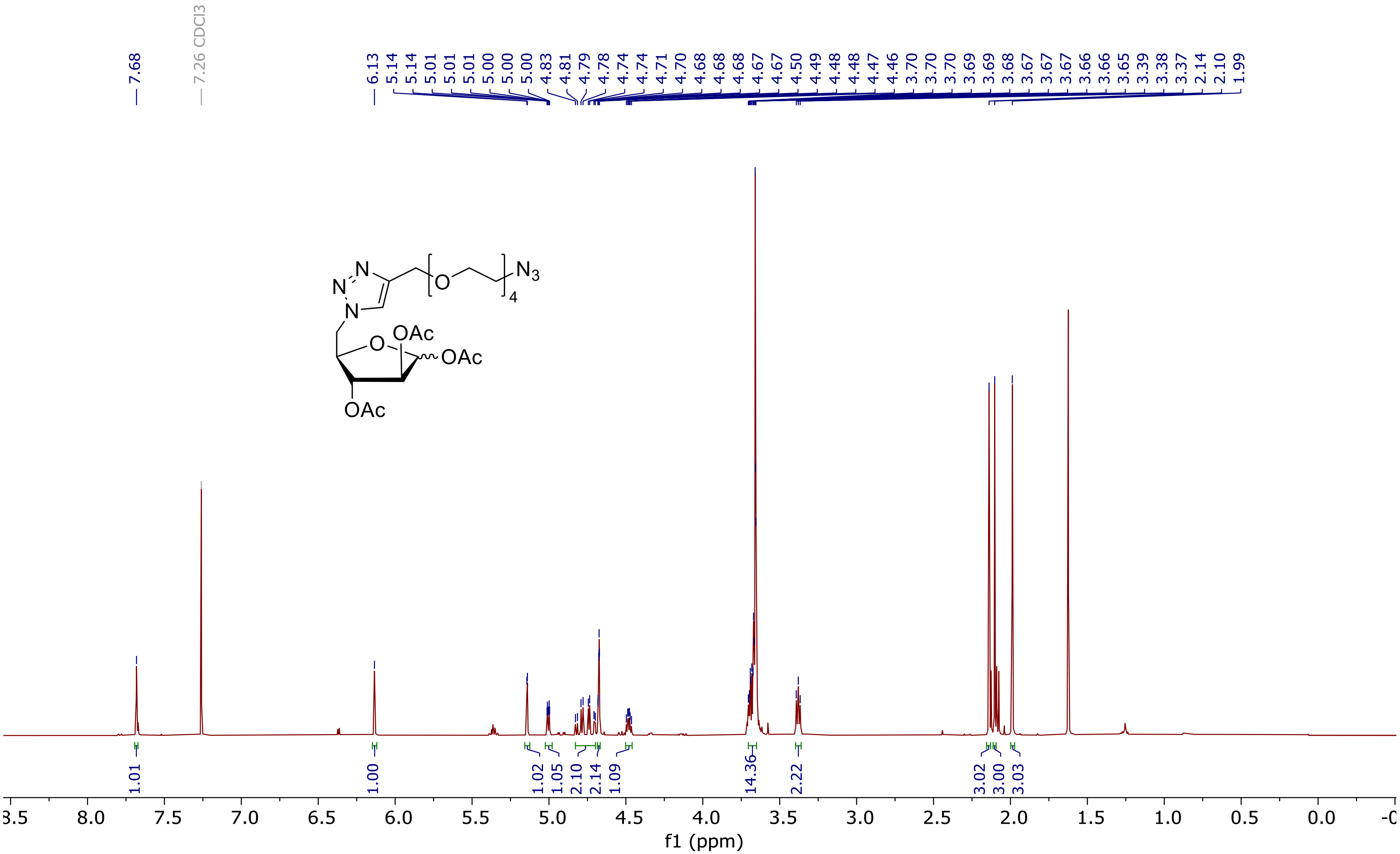

^{13}C NMR Spectrum (100.68 MHz, $CDCl_3$) of **Compound 4**

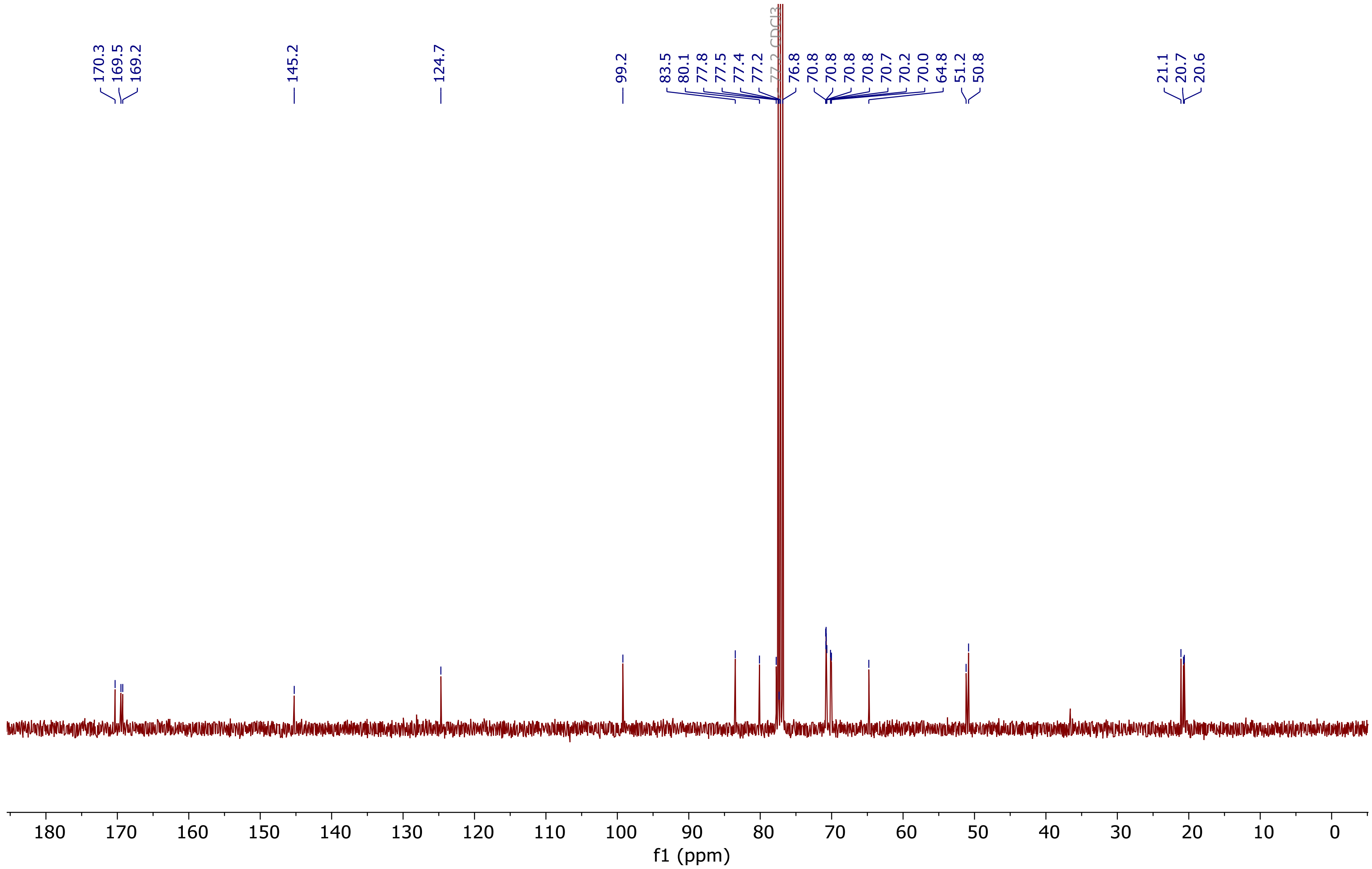

HSQC NMR Spectrum (400.34, 100.67 MHz, $CDCl_3$) of **Compound 4**

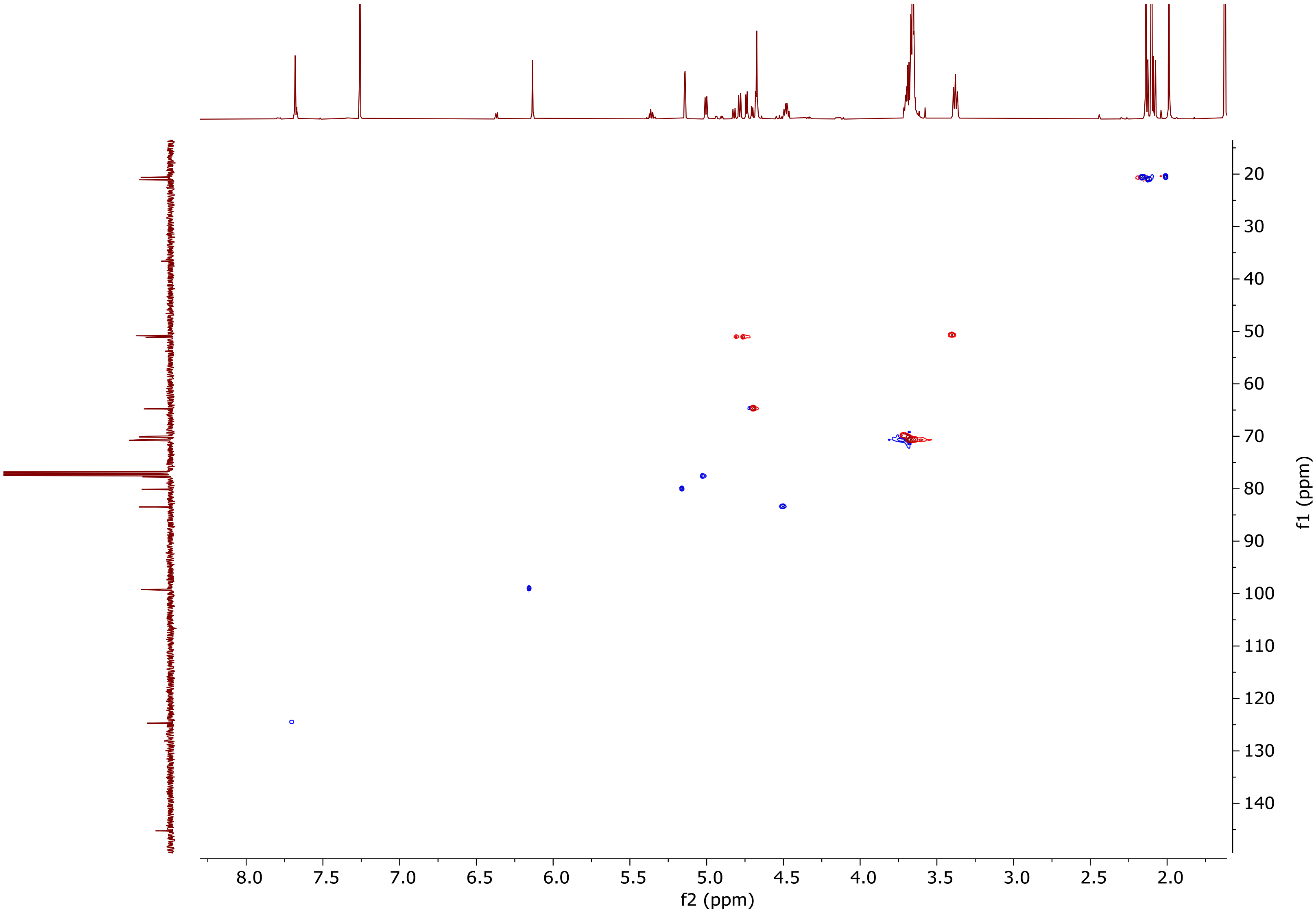

COSY NMR Spectrum (400.34 MHz, $CDCl_3$) of **Compound 4**

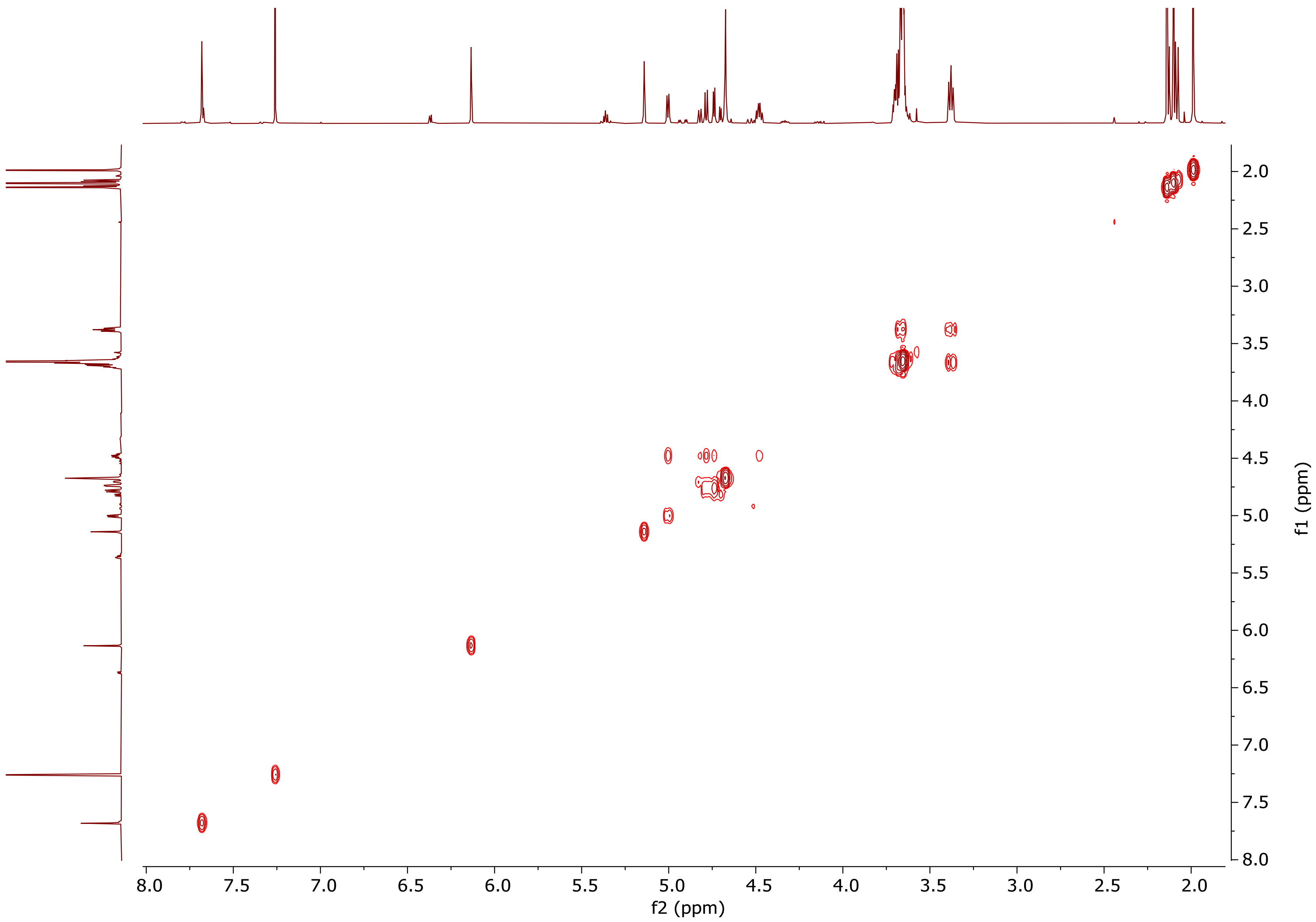

HMBC NMR Spectrum (400.34, 100.68 MHz, $CDCl_3$) of **Compound 4**

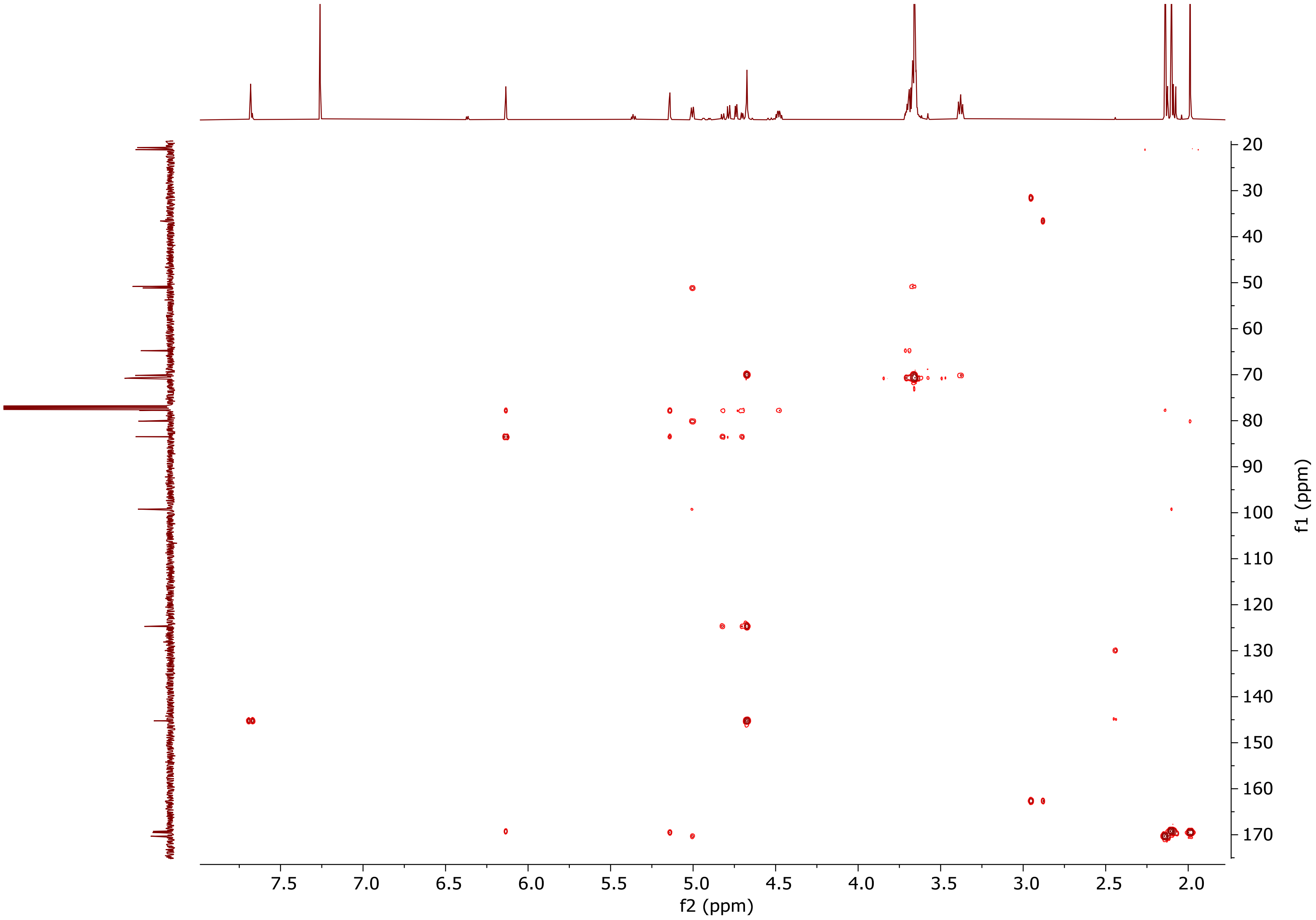

^{1}H NMR Spectrum (400.34 MHz, $CDCl_3$) of **Compound 3**

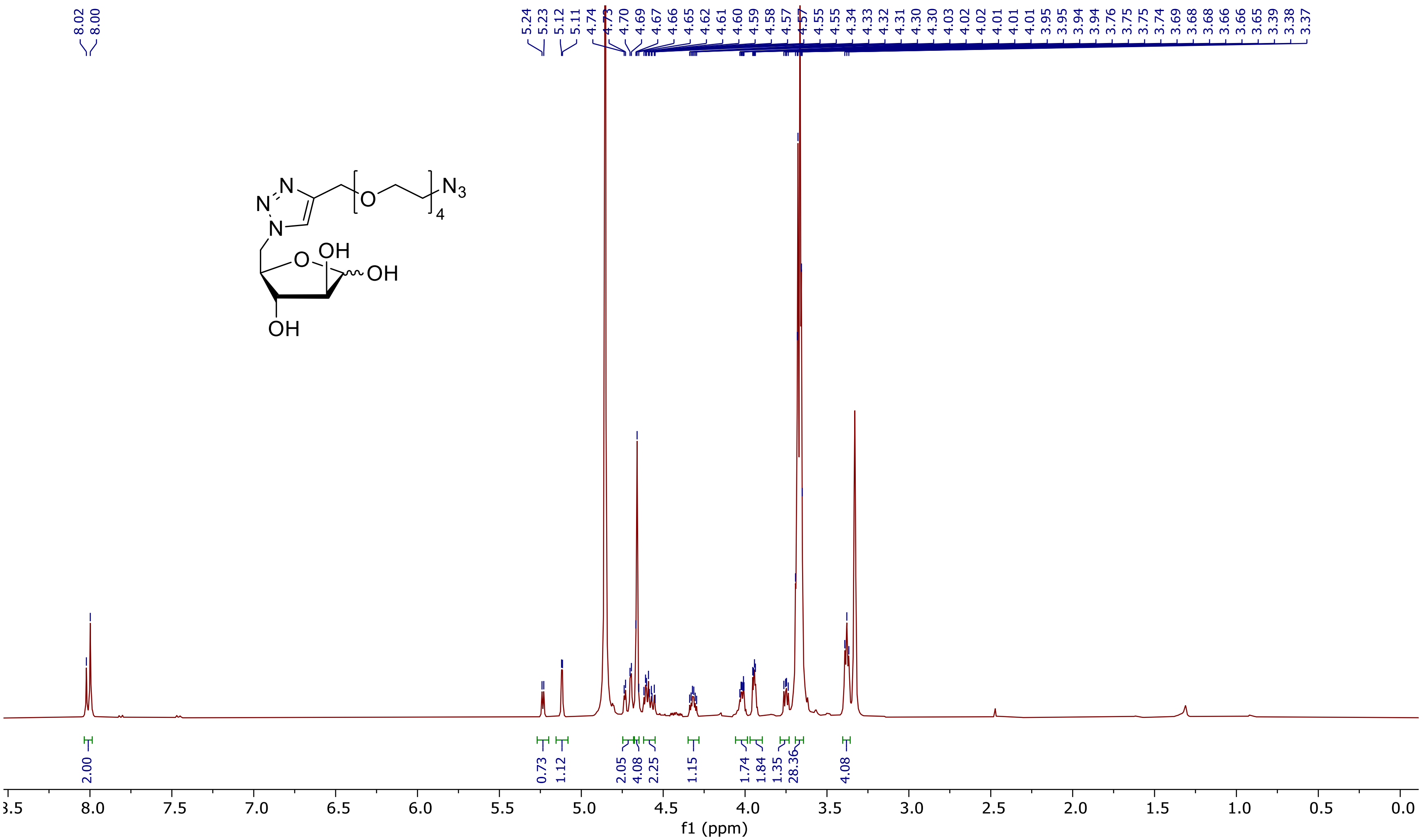

^{13}C NMR Spectrum (100.68 MHz, $CDCl_3$) of Compound 3

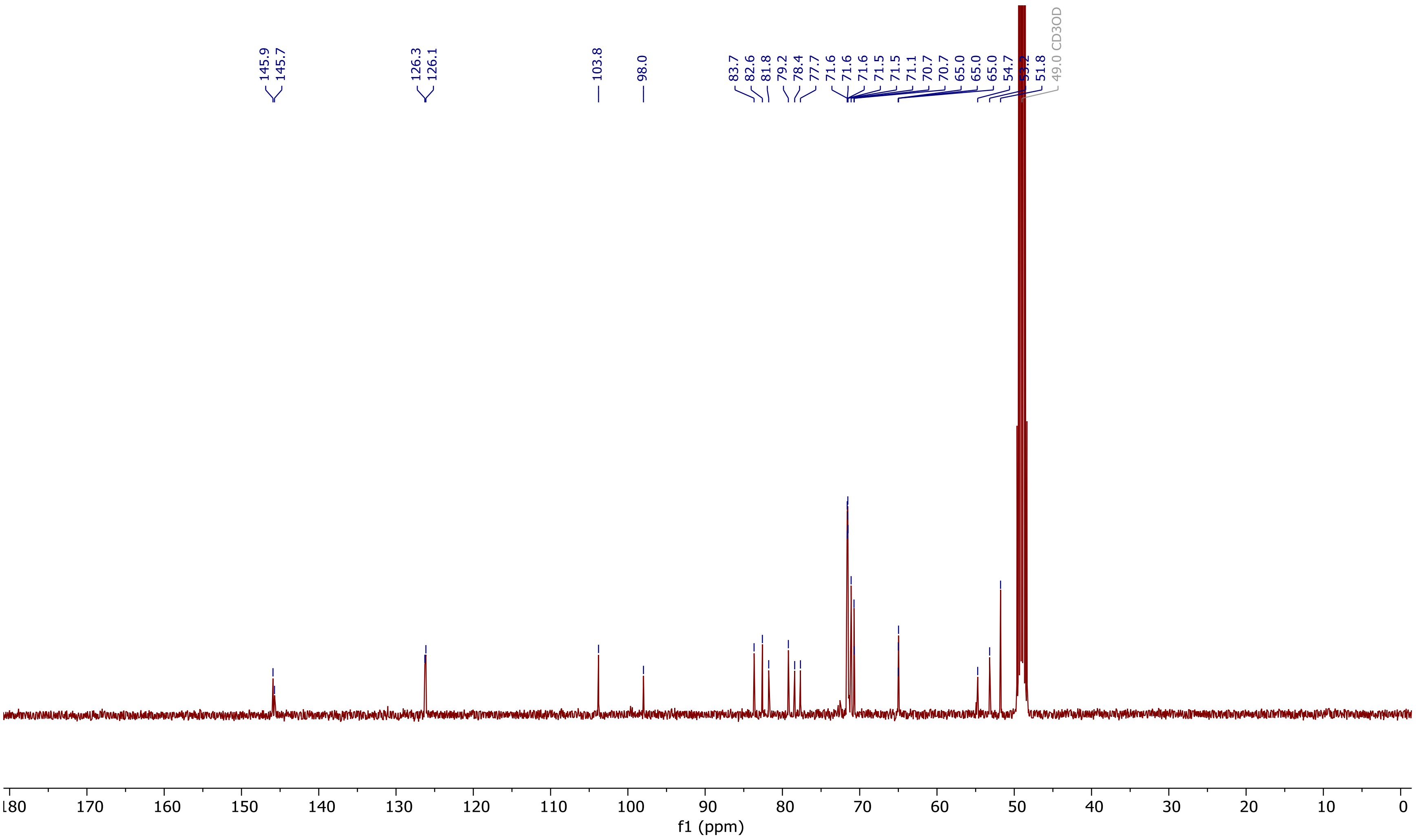

DEPT NMR Spectrum (100.67 MHz, $CDCl_3$) of Compound 3

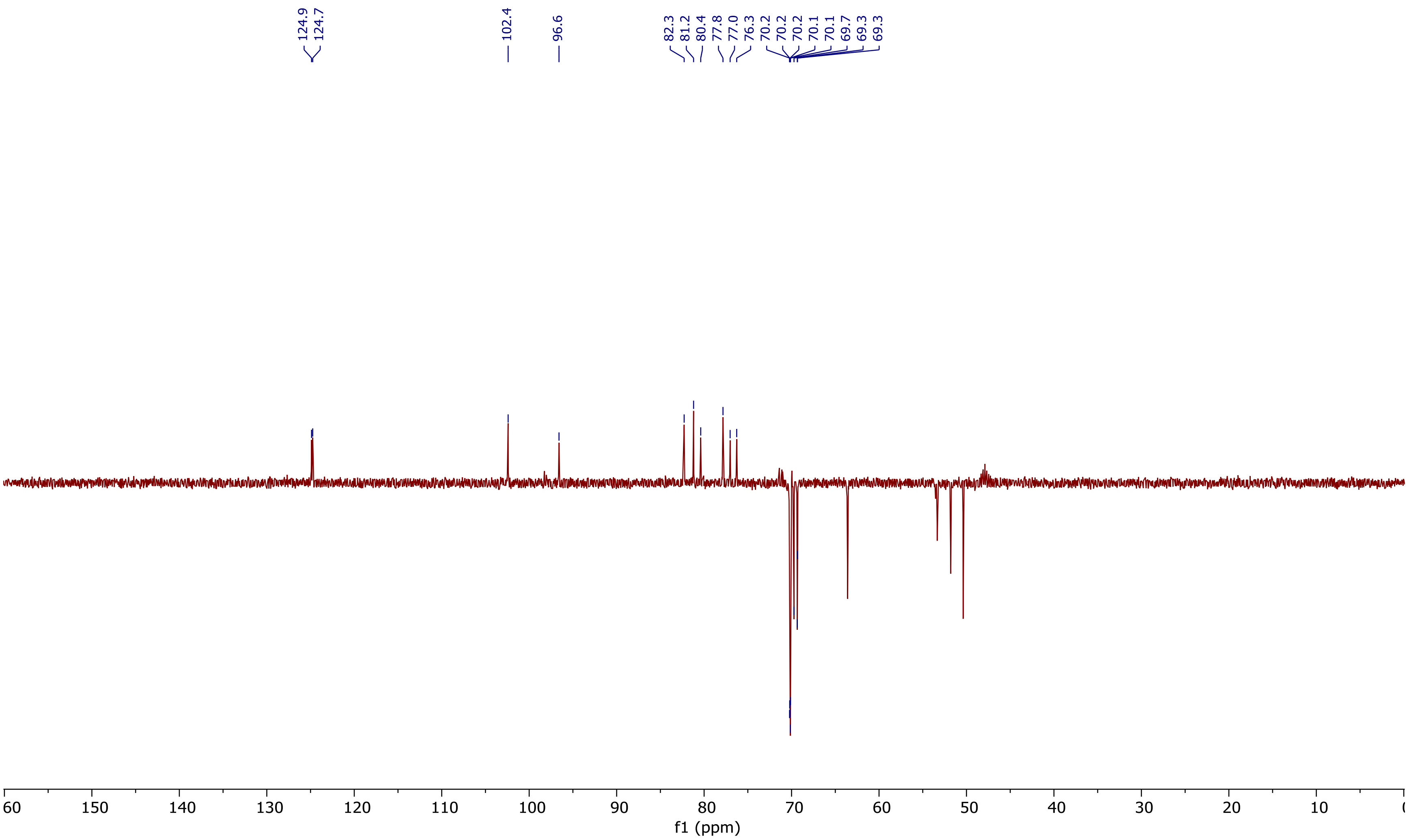

HSQC NMR Spectrum (400.34, 100.67 MHz, $CDCl_3$) of Compound 3

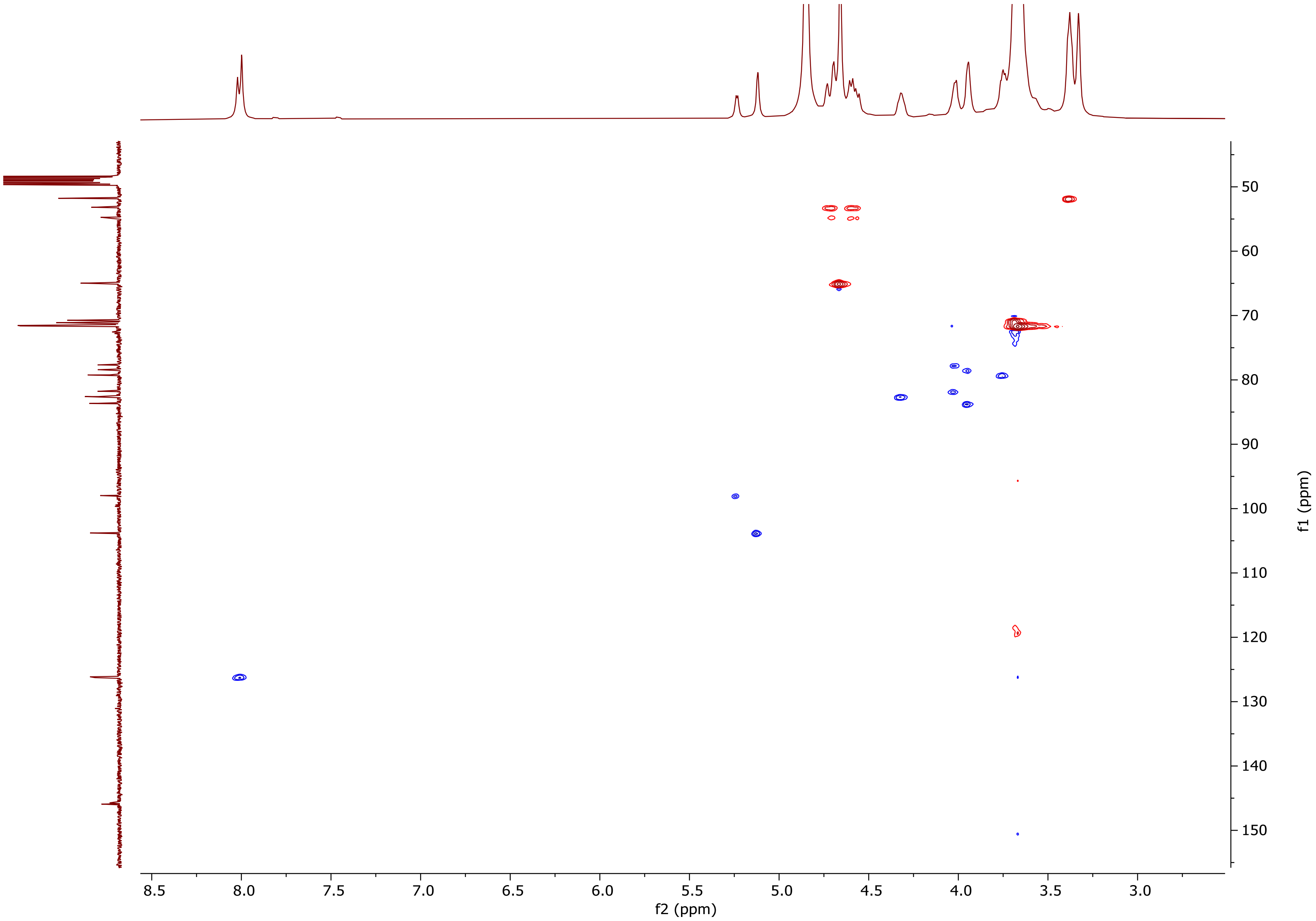

HMBC NMR Spectrum (400.34, 100.68 MHz, $CDCl_3$) of Compound 3

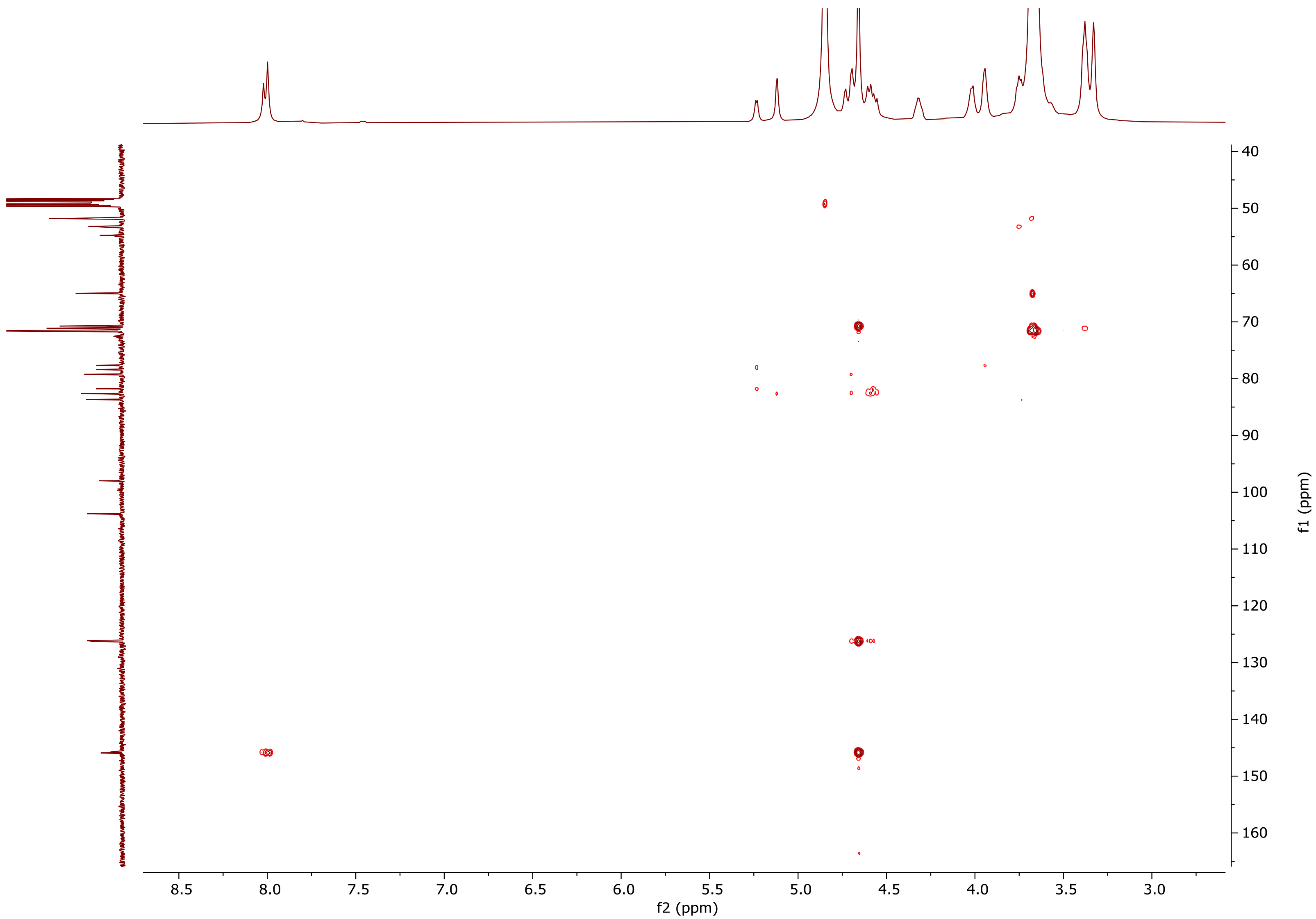

^{1}H NMR Spectrum (400.34 MHz, $CDCl_3$) of Compound 1

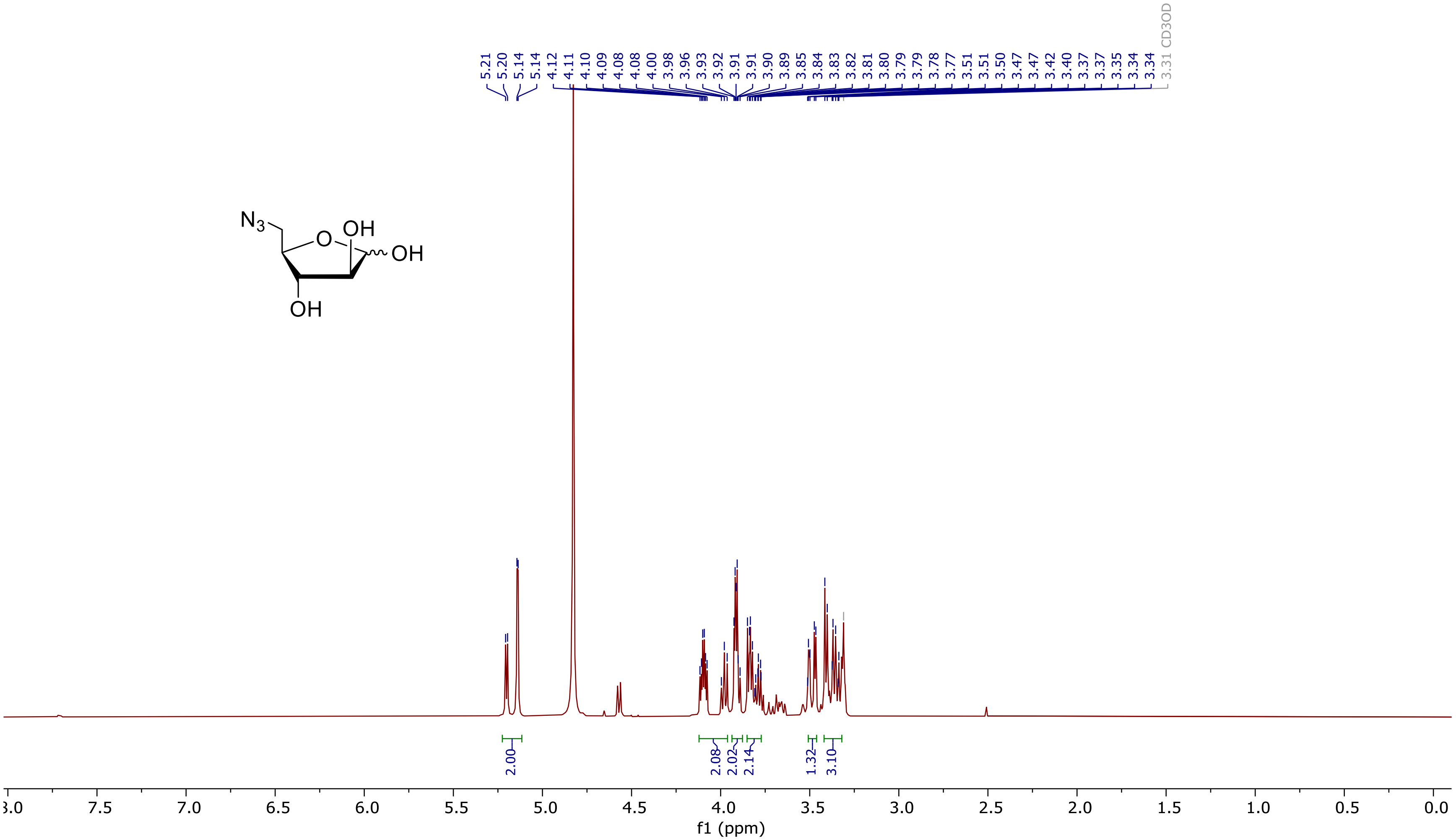

^{13}C NMR Spectrum (100.68 MHz, $CDCl_3$) of Compound 1

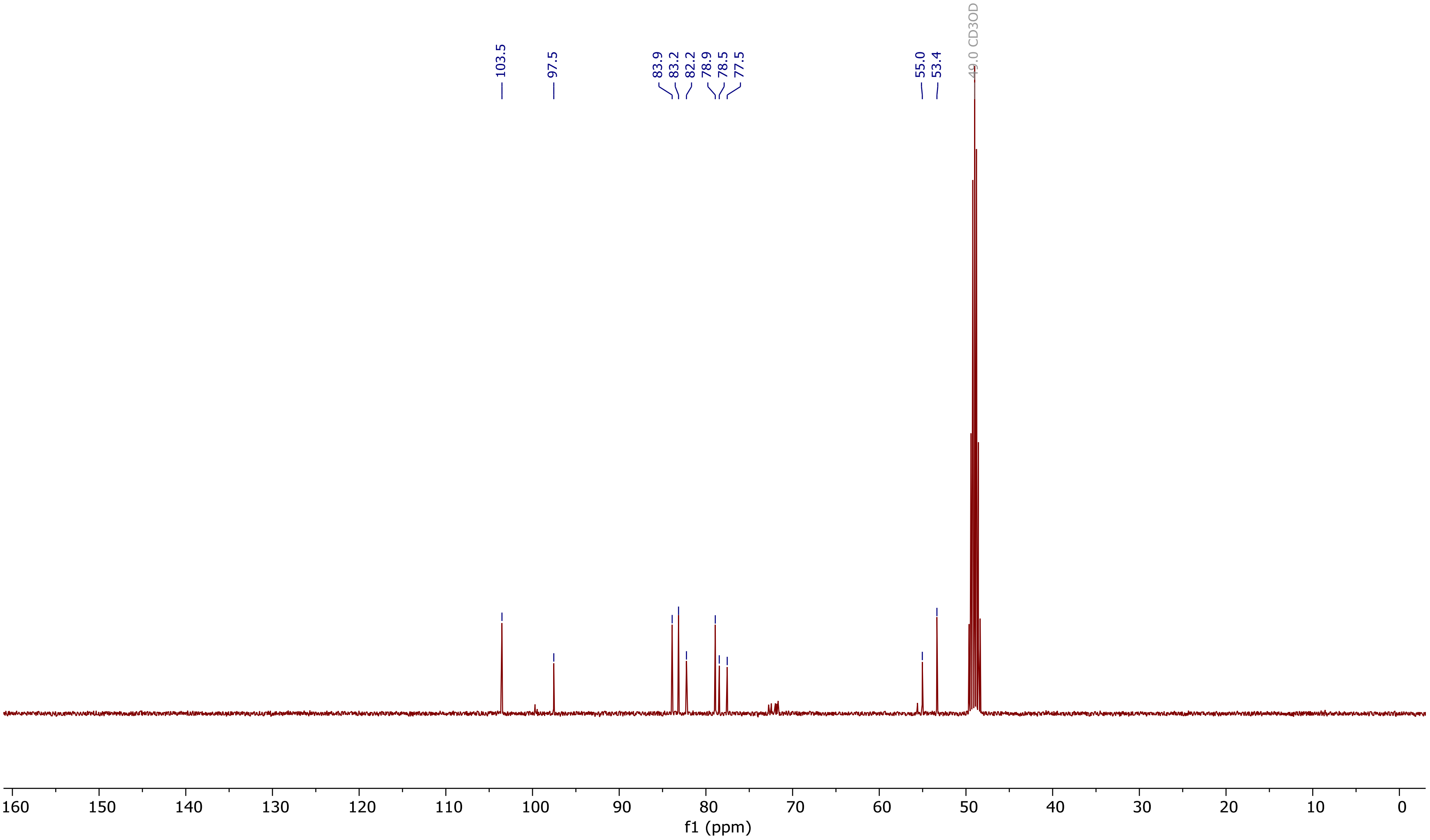

DEPT NMR Spectrum (100.67 MHz, $CDCl_3$) of Compound 1

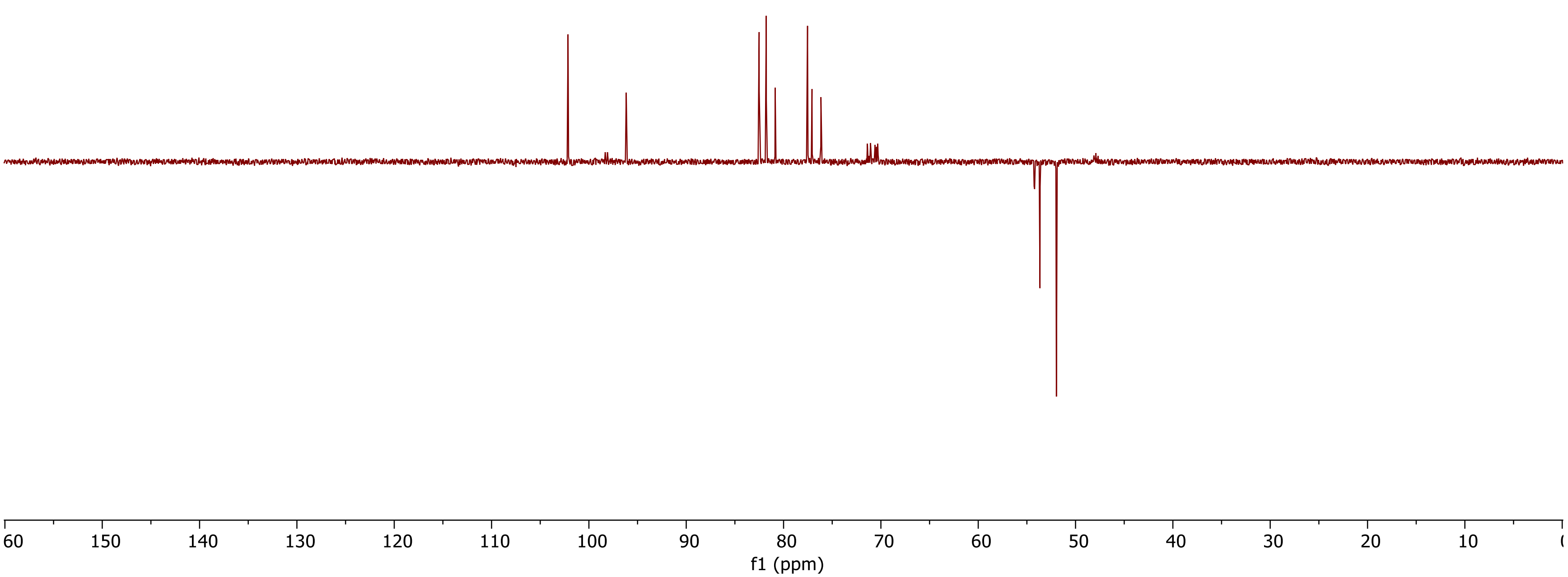

HSQC NMR Spectrum (400.34, 100.67 MHz, CDCl$_3$) of Compound 1

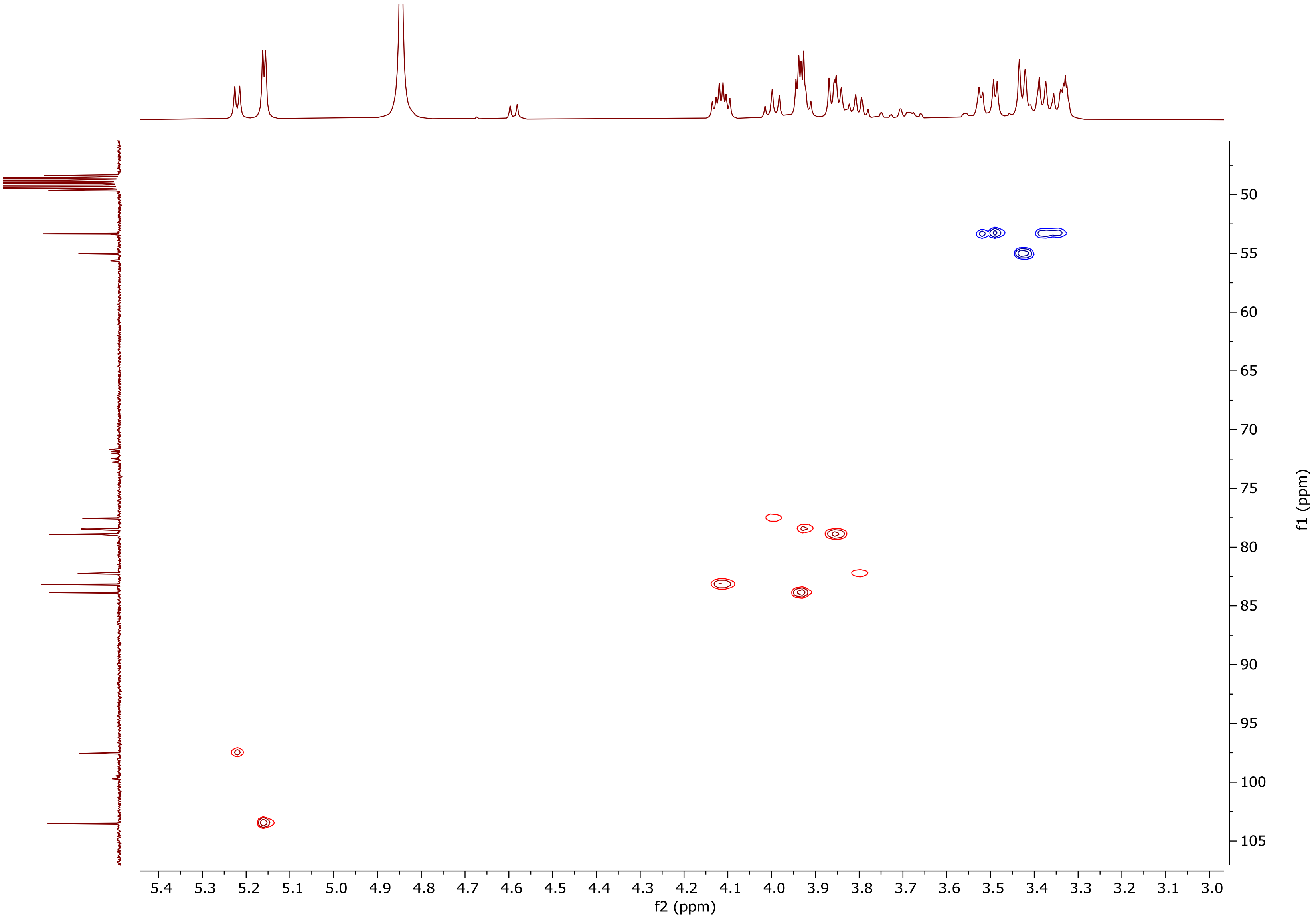

COSY NMR Spectrum (400.34 MHz, $CDCl_3$) of Compound 1

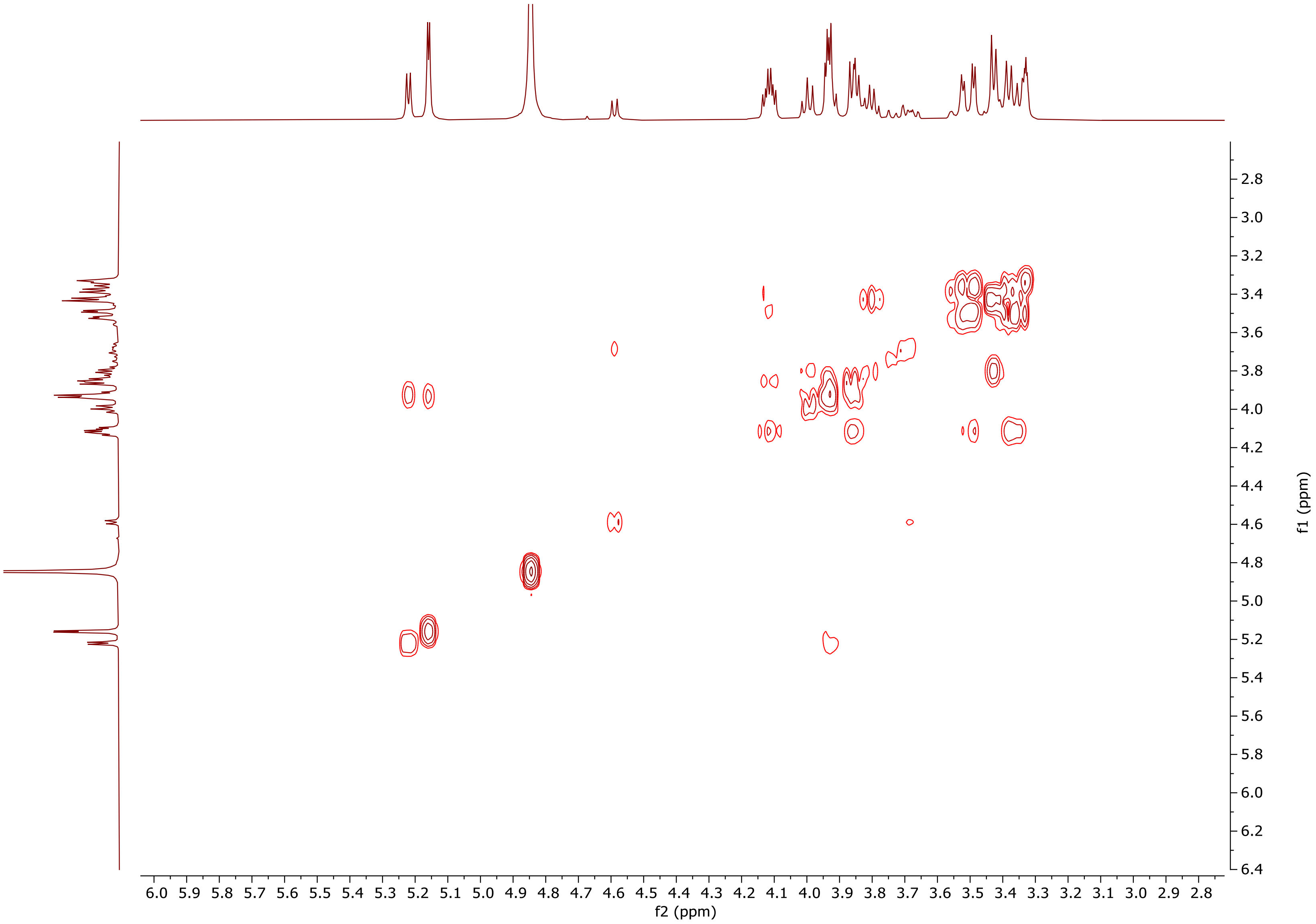

HMBC NMR Spectrum (400.34, 100.68 MHz, $CDCl_3$) of Compound 1

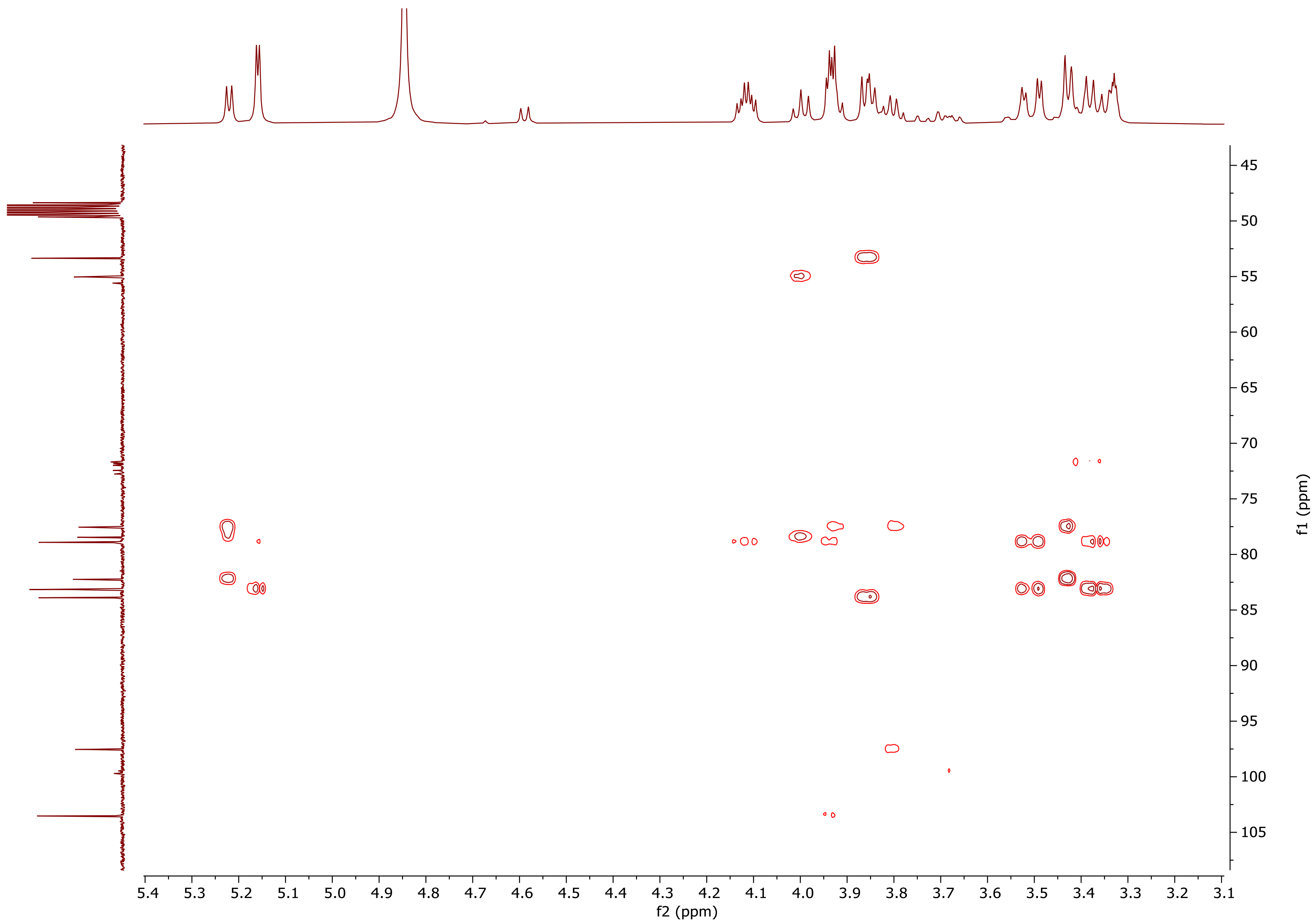

^{1}H NMR Spectrum (400.34 MHz, $CDCl_3$) of Compound 21

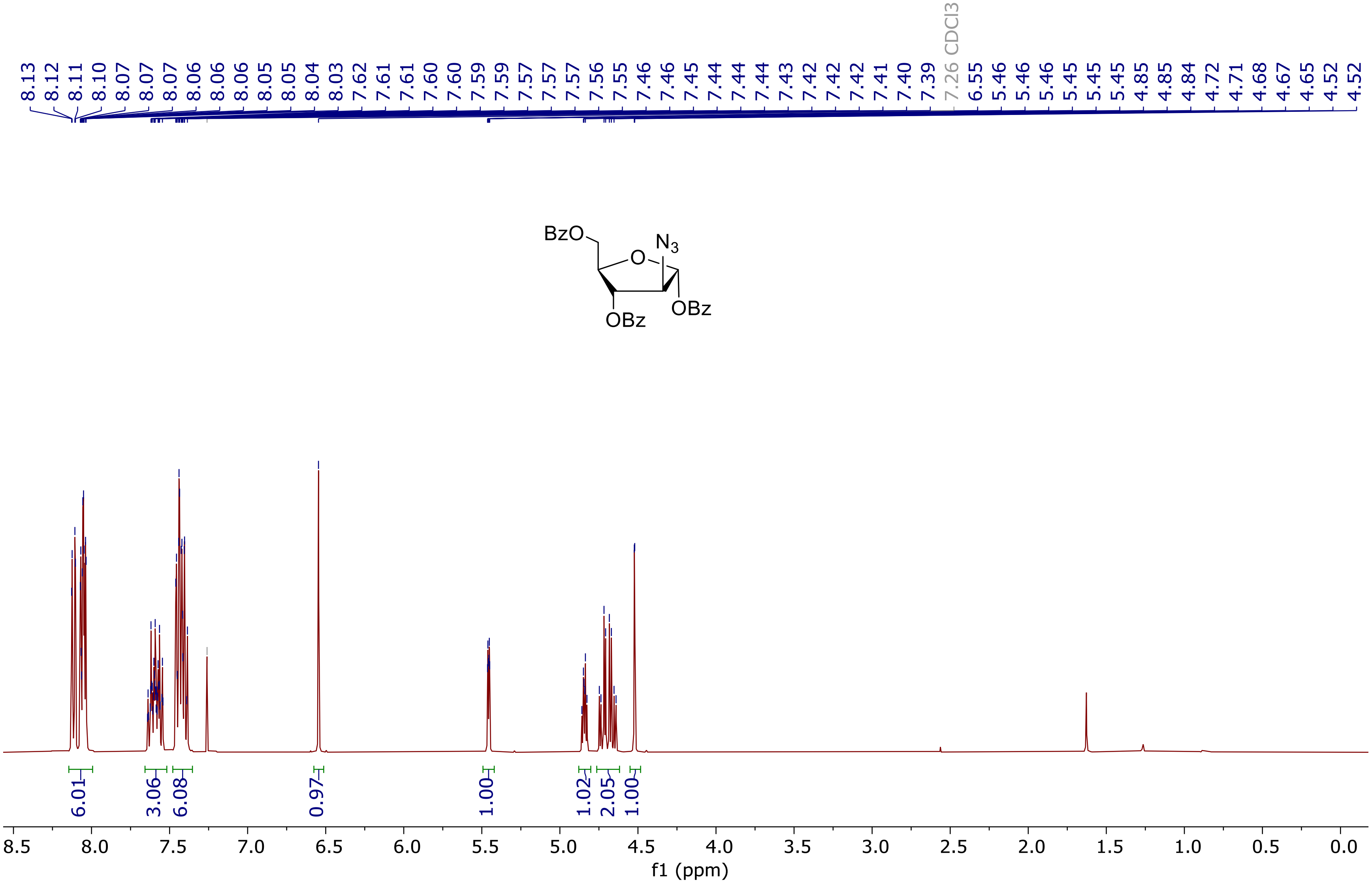

^{13}C NMR Spectrum (100.68 MHz, $CDCl_3$) of Compound 21

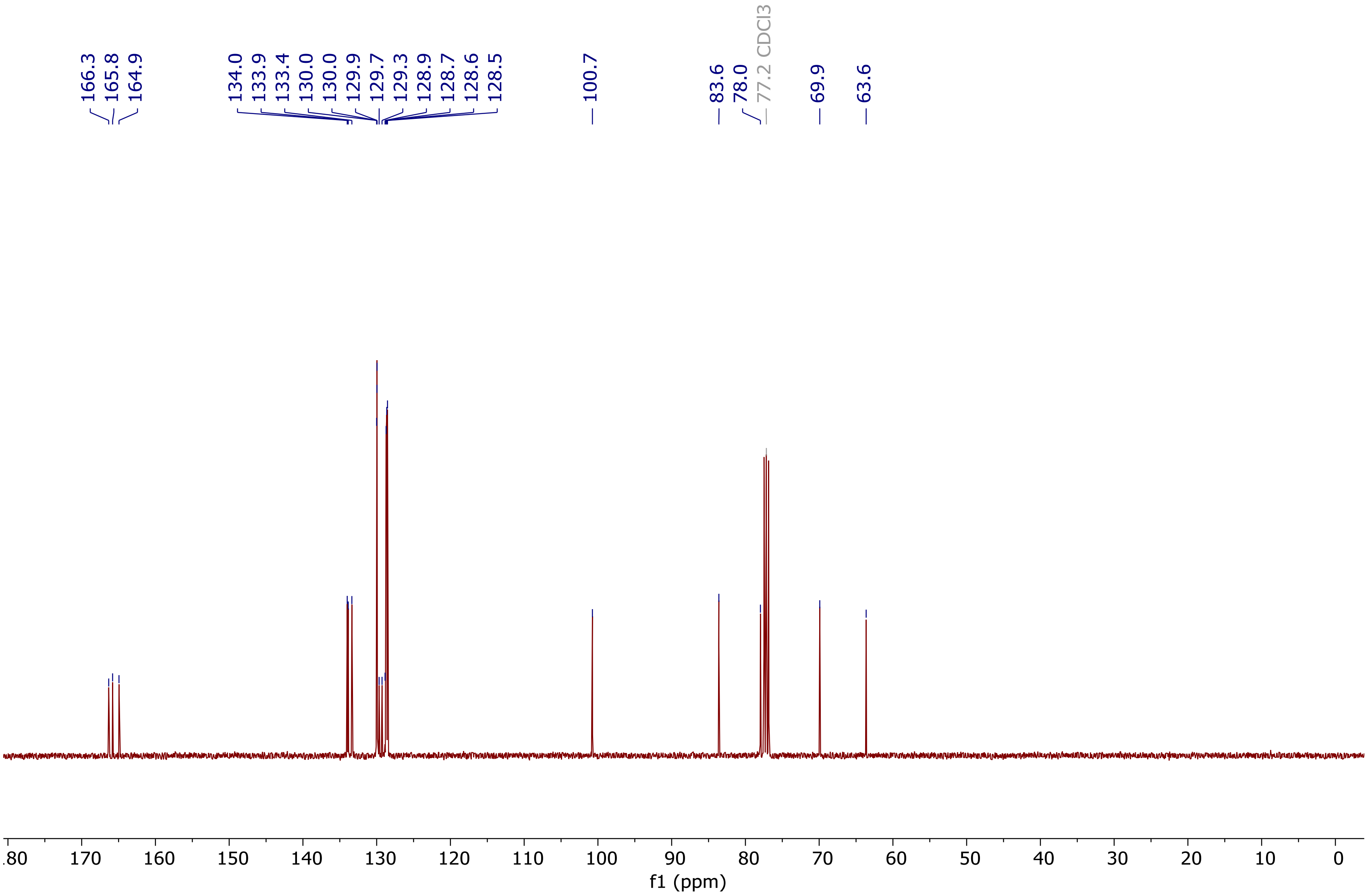

HSQC NMR Spectrum (400.34, 100.67 MHz, $CDCl_3$) of Compound 21

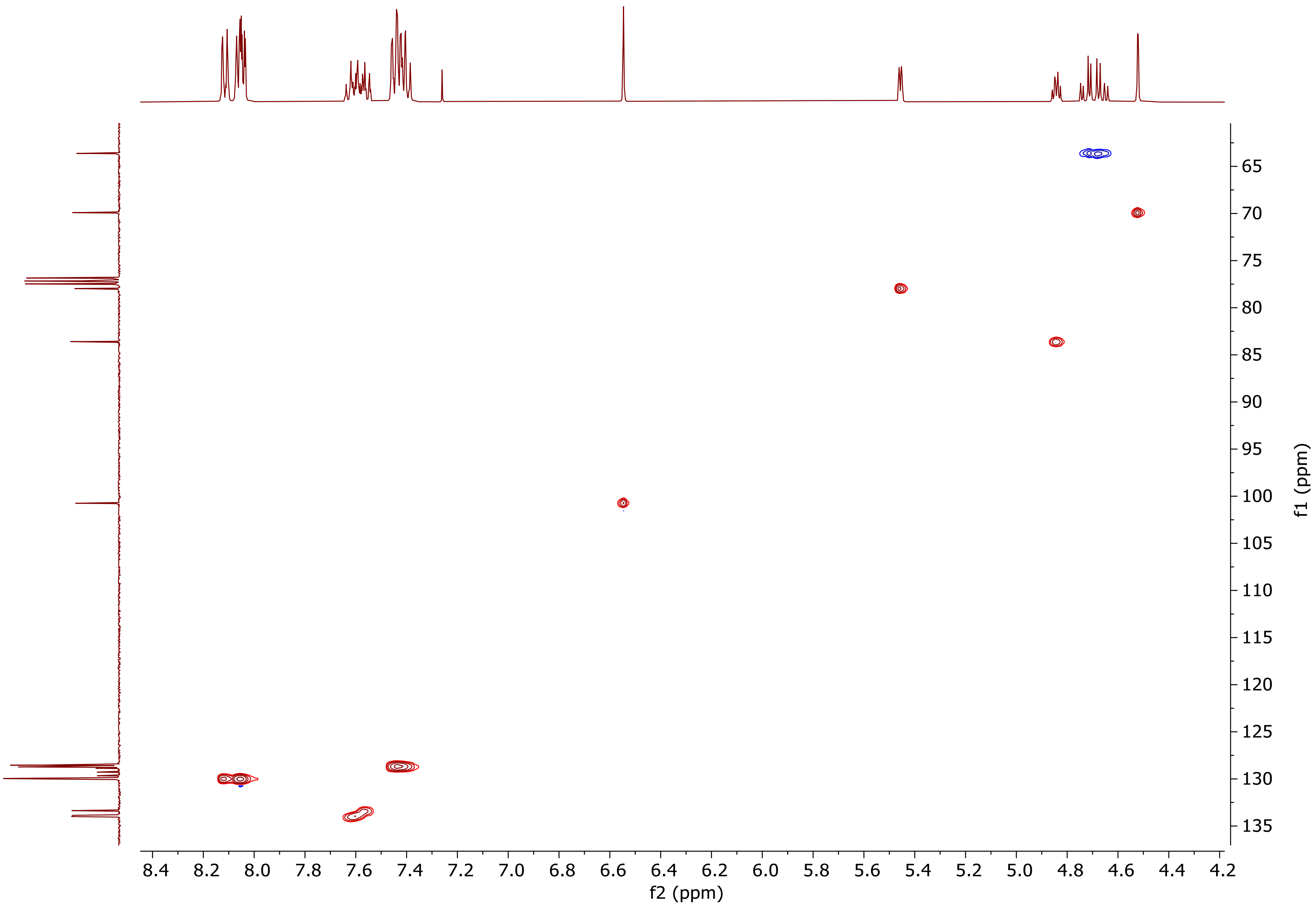

COSY NMR Spectrum (400.34 MHz, $CDCl_3$) of Compound 21

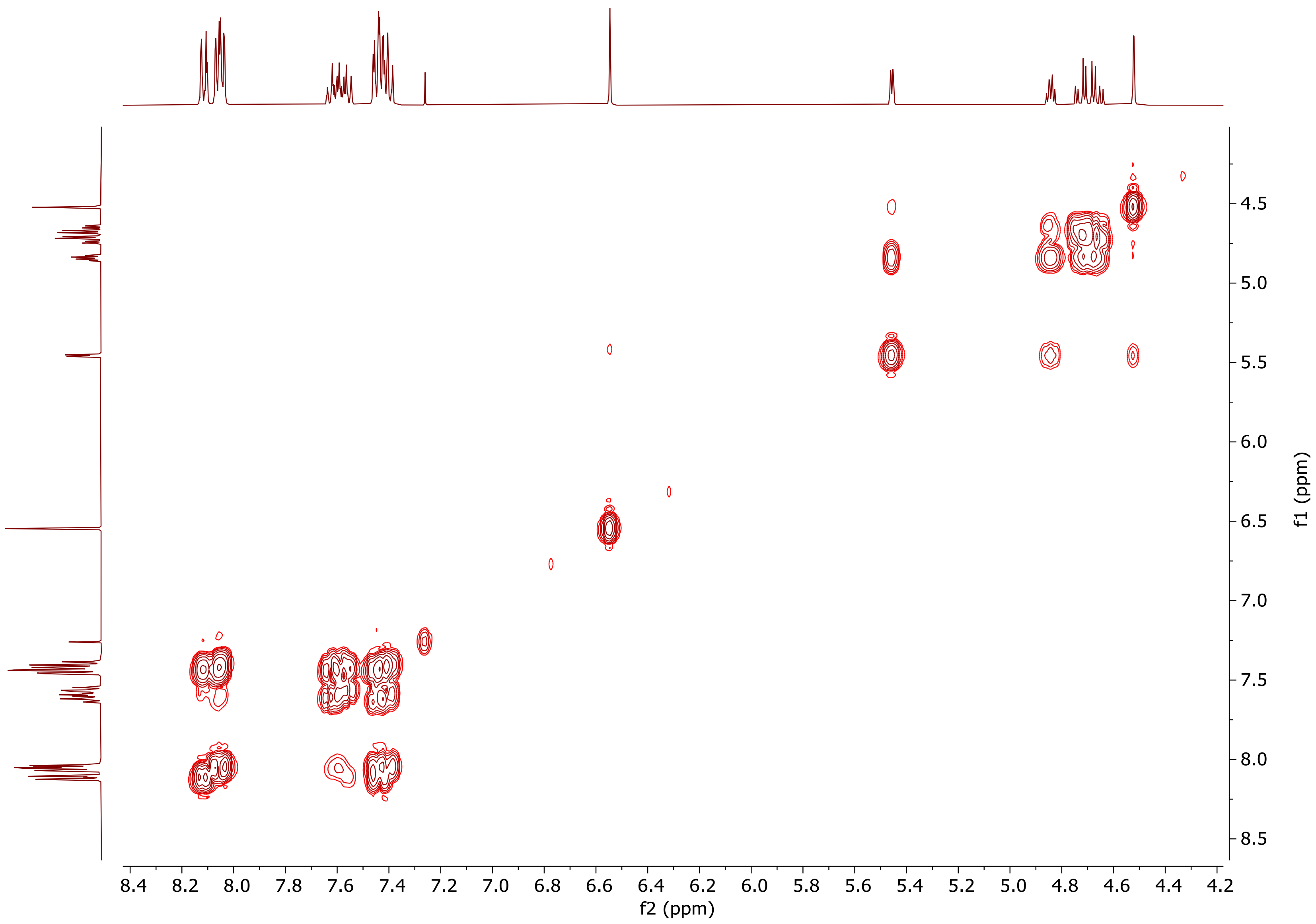

HMBC NMR Spectrum (400.34, 100.68 MHz, $CDCl_3$) of Compound 21

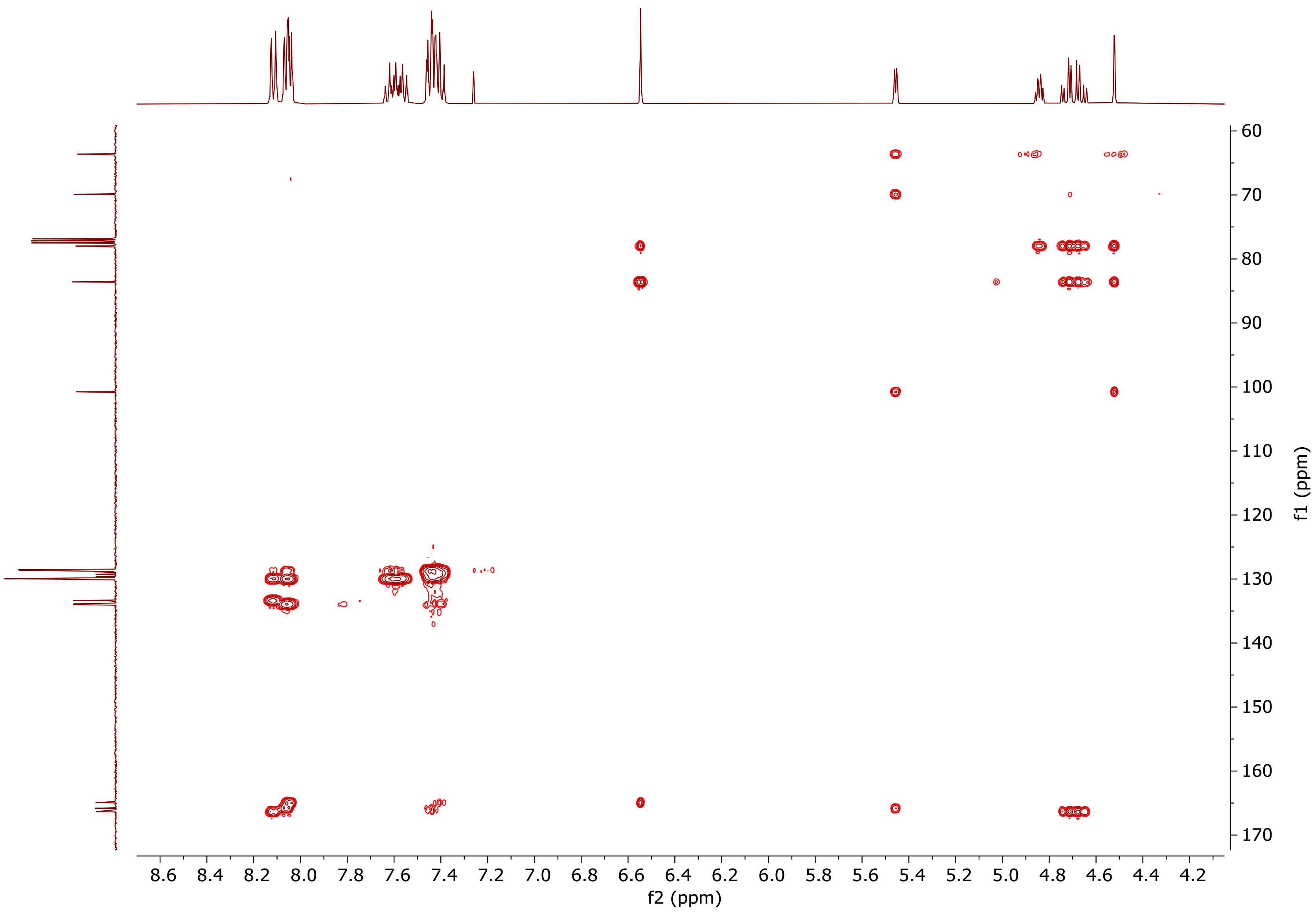

^{1}H NMR Spectrum (400.34 MHz, $CDCl_3$) of Compound 22

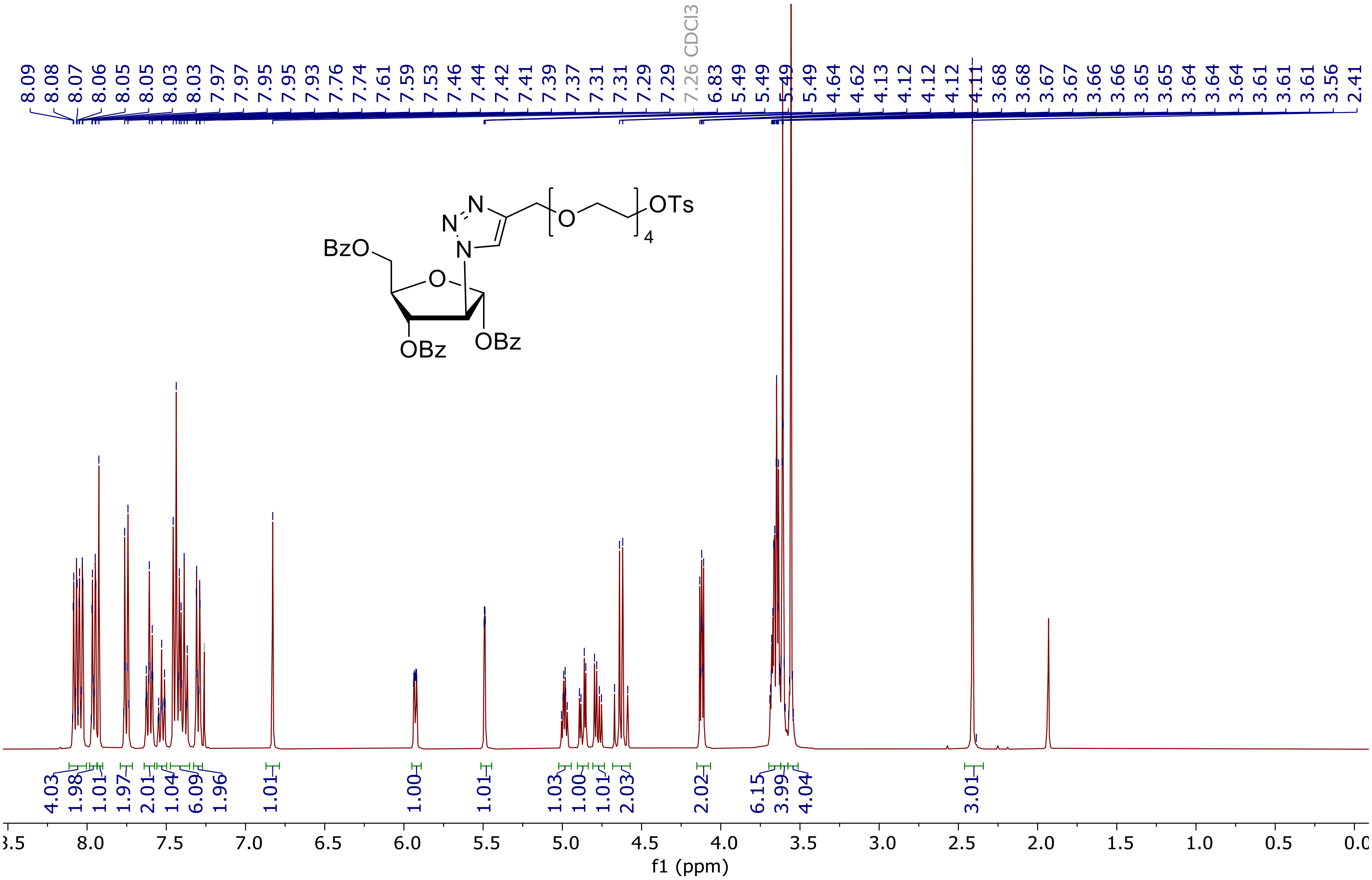

^{13}C NMR Spectrum (100.68 MHz, $CDCl_3$) of Compound 22

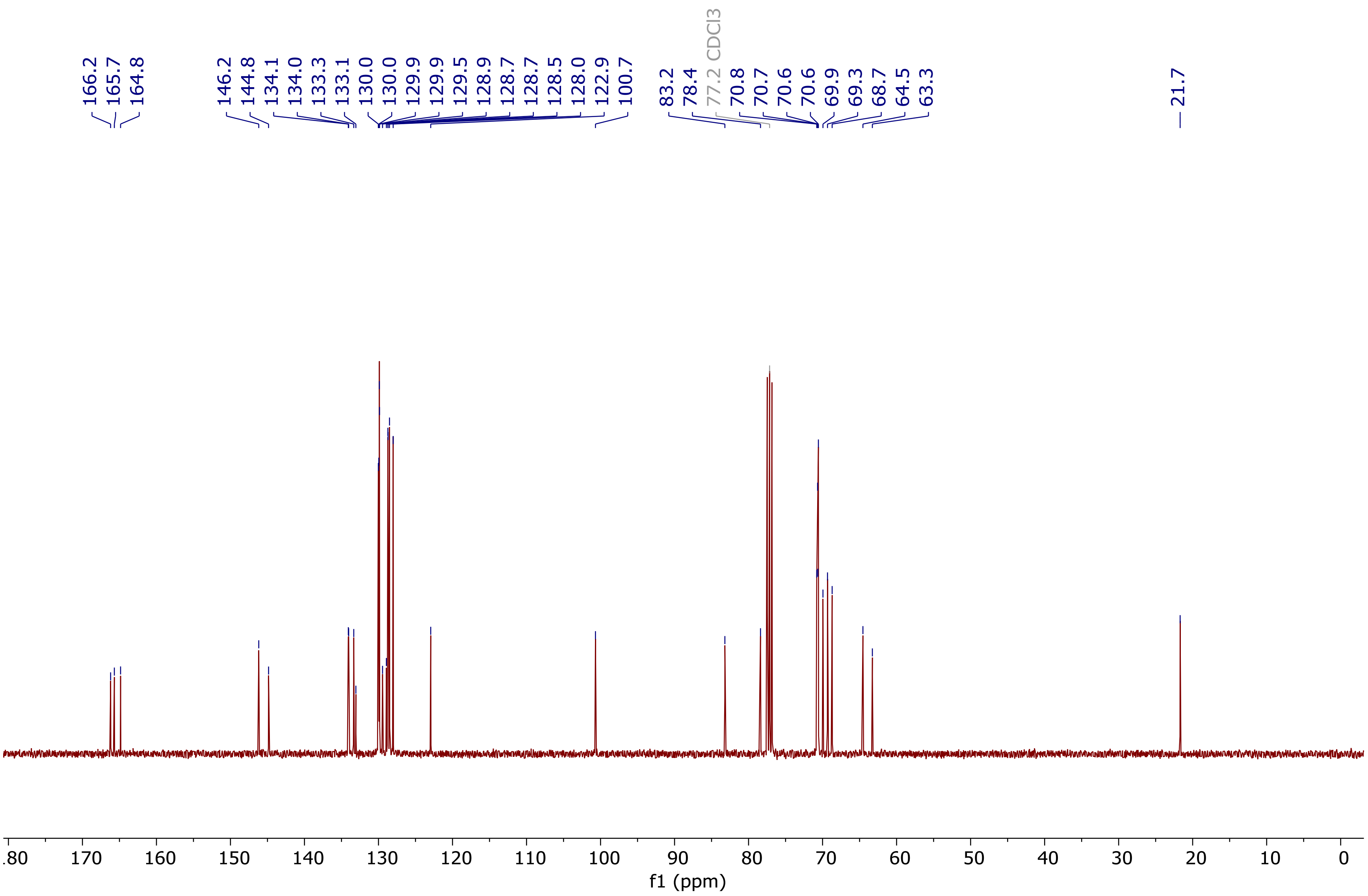

HSQC NMR Spectrum (400.34, 100.67 MHz, $CDCl_3$) of Compound 22

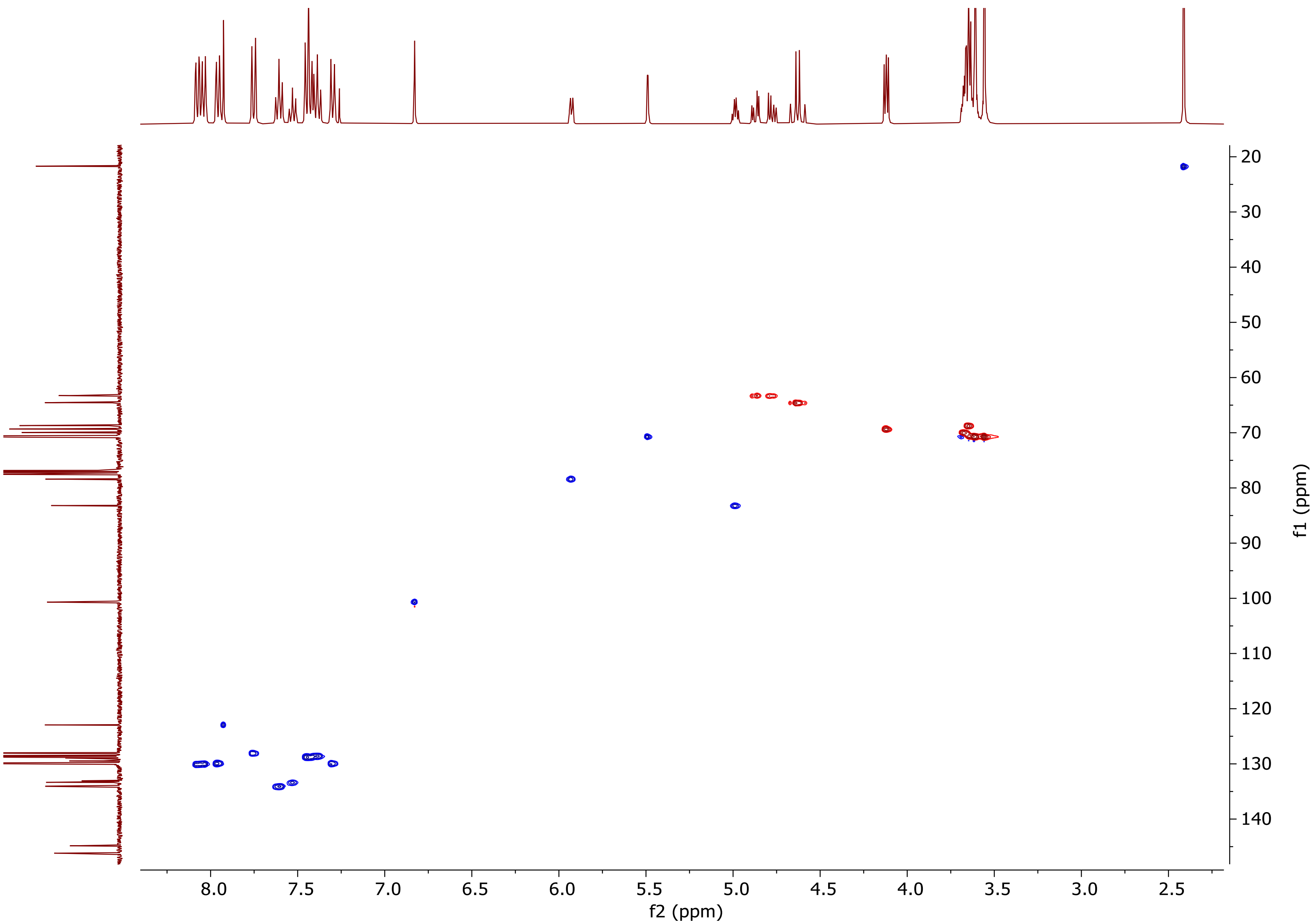

COSY NMR Spectrum (400.34 MHz, $CDCl_3$) of Compound 22

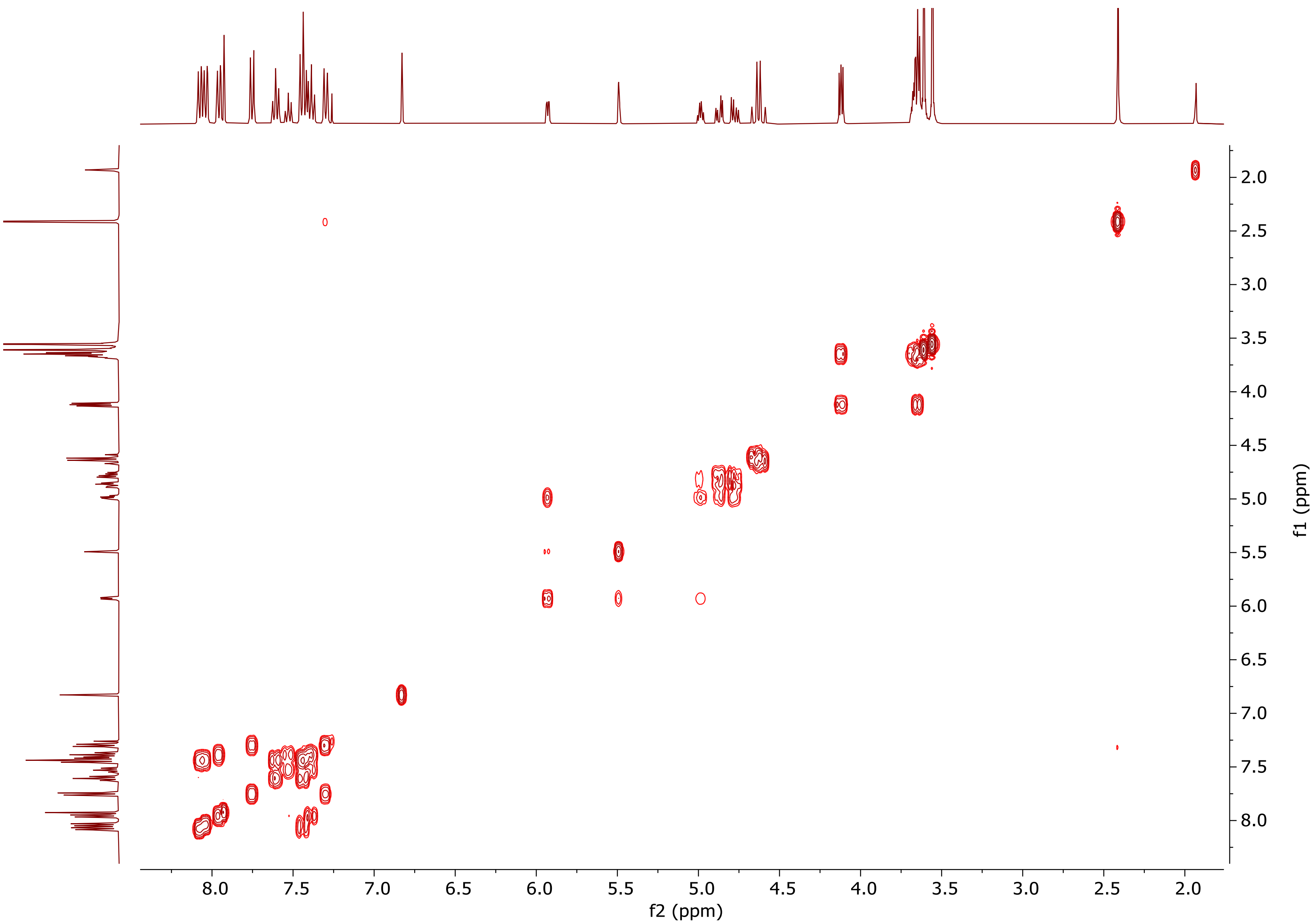

HMBC NMR Spectrum (400.34, 100.68 MHz, $CDCl_3$) of Compound 22

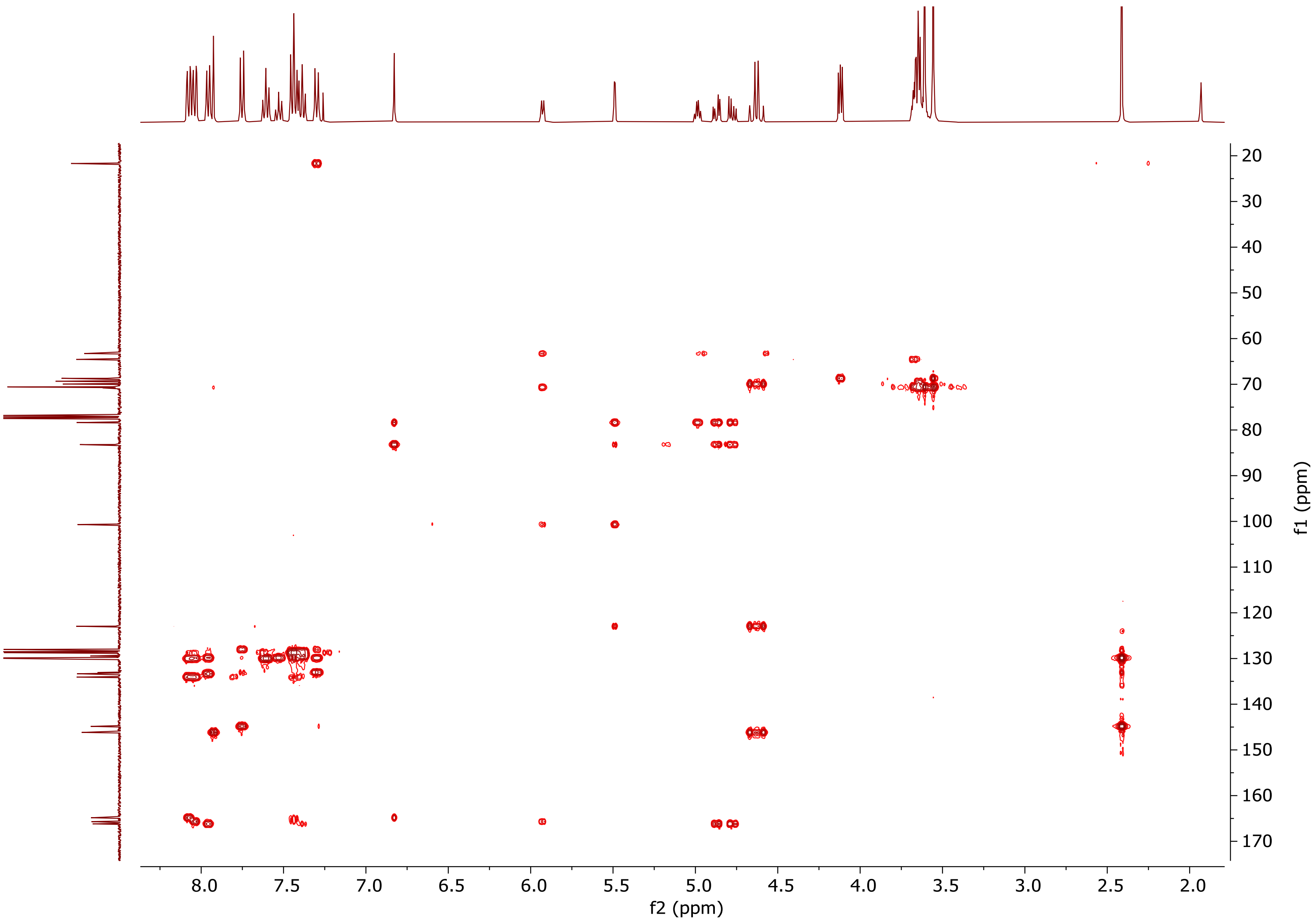

^{1}H NMR Spectrum (400.34 MHz, $CDCl_3$) of Compound 9

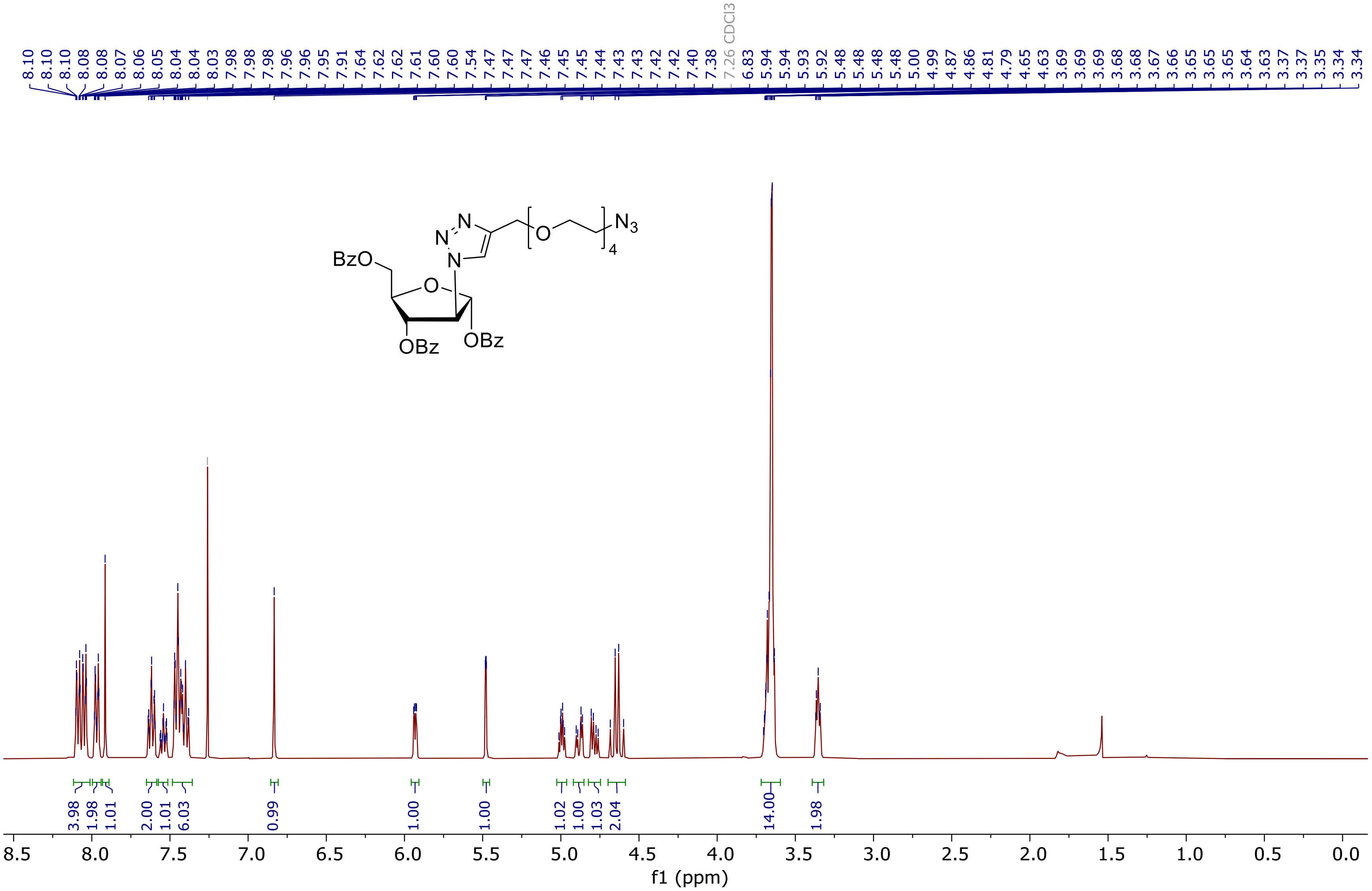

^{13}C NMR Spectrum (100.68 MHz, $CDCl_3$) of Compound 9

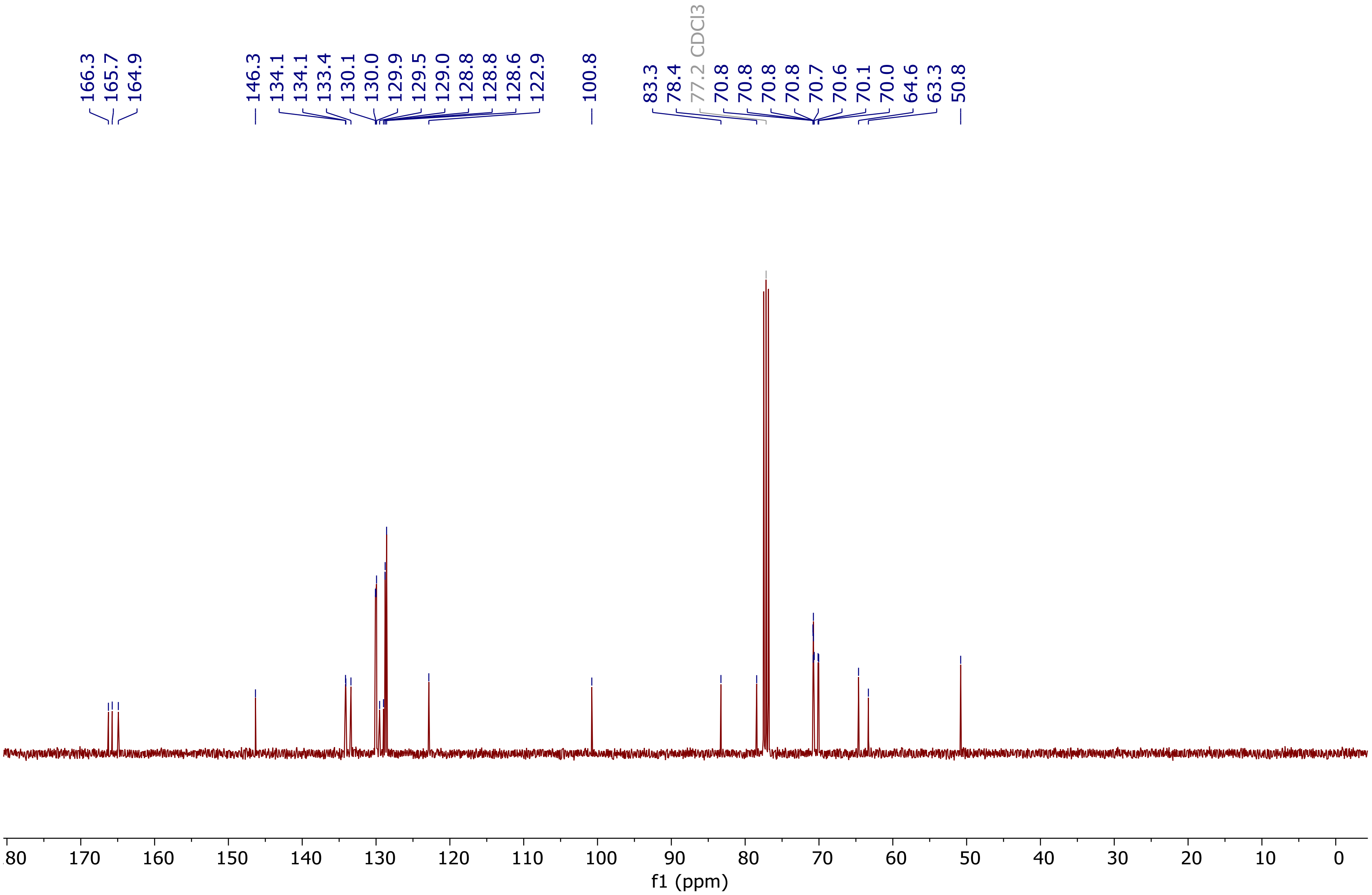

HSQC NMR Spectrum (400.34, 100.67 MHz, $CDCl_3$) of Compound 9

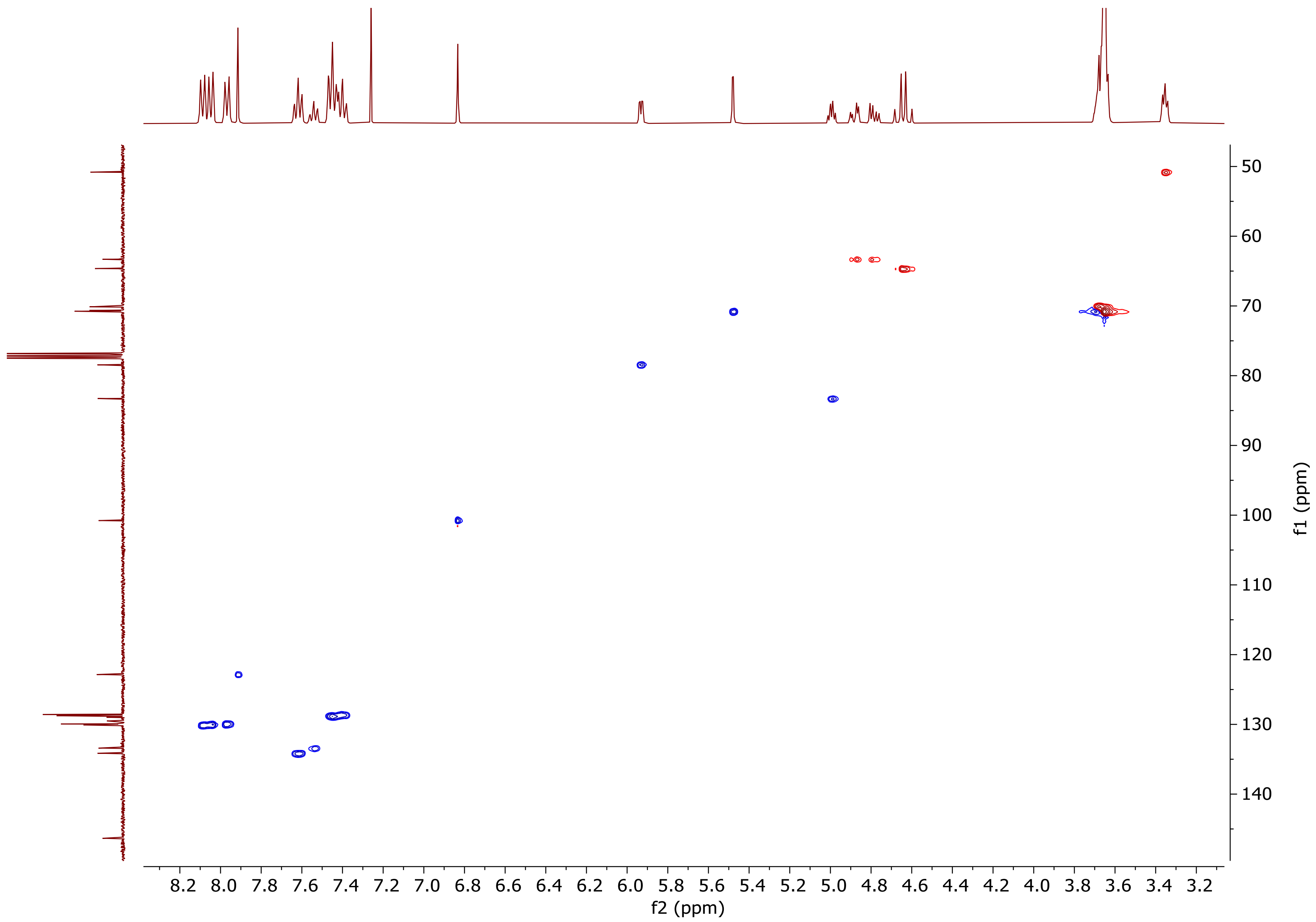

COSY NMR Spectrum (400.34 MHz, $CDCl_3$) of Compound 9

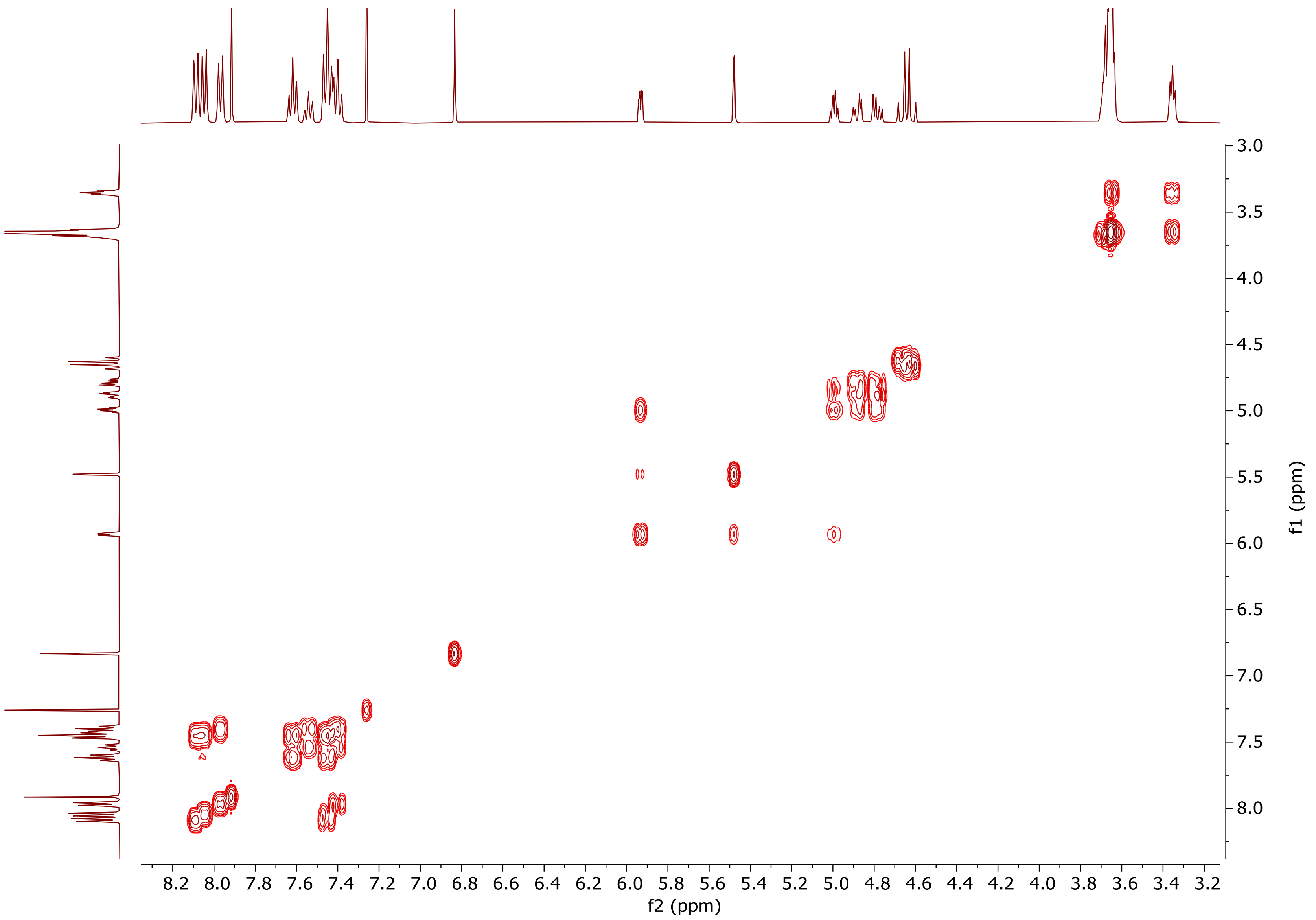

HMBC NMR Spectrum (400.34, 100.68 MHz, CDCl$_3$) of Compound 9

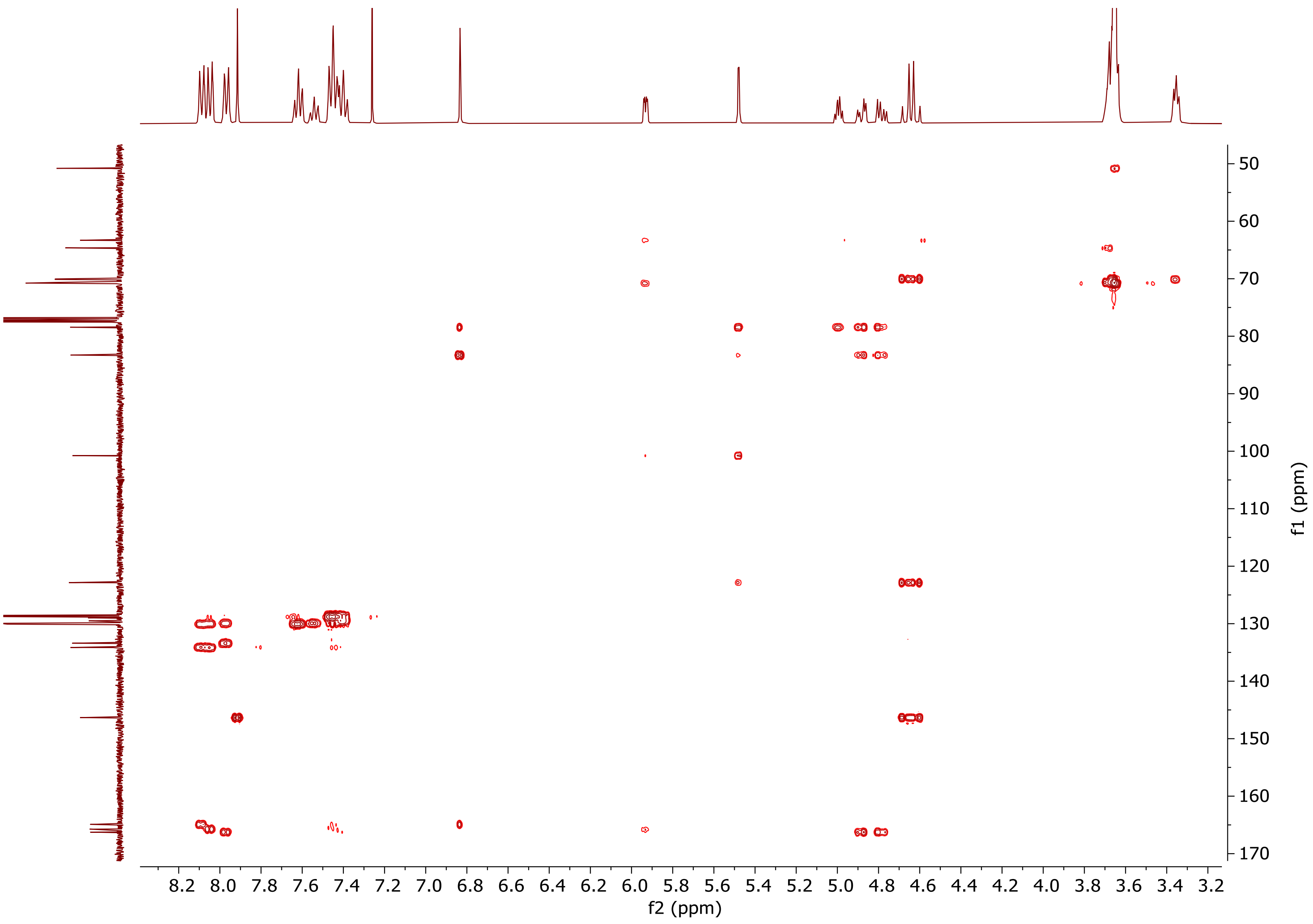

^{1}H NMR Spectrum (500.23 MHz, $CDCl_3$) of Compound 7

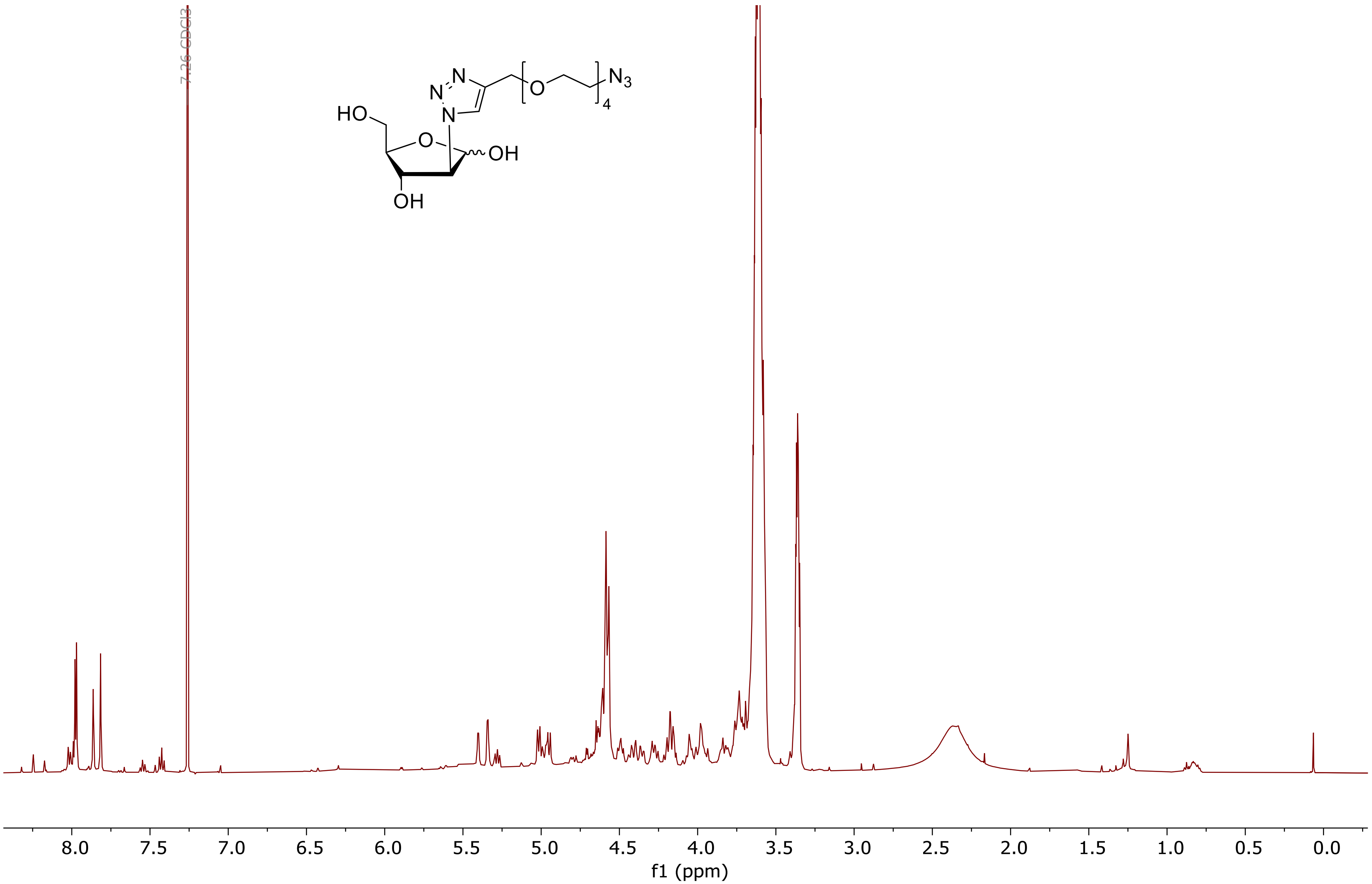

^{13}C NMR Spectrum (125.80 MHz, $CDCl_3$) of Compound 7

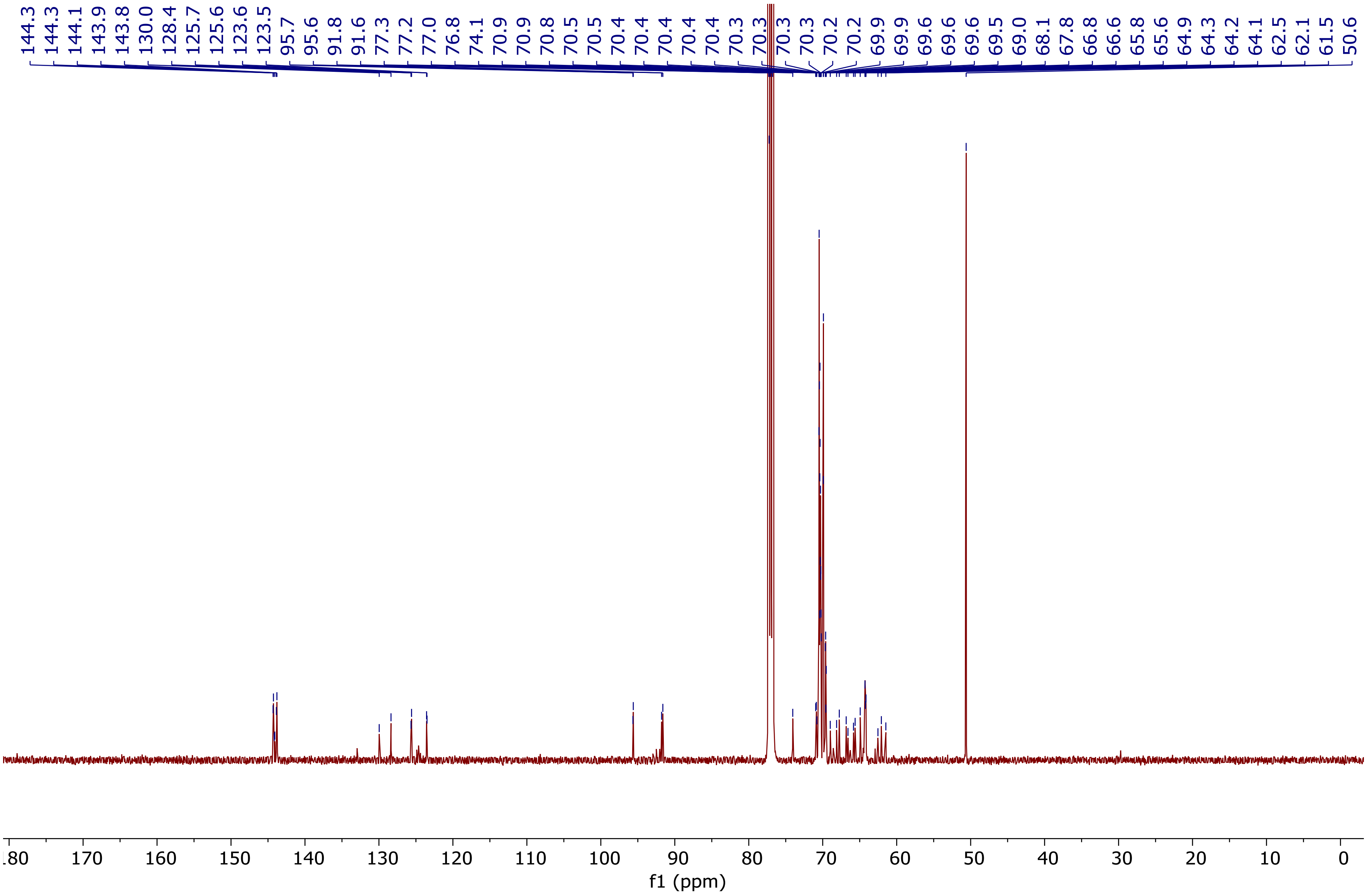

HSQC NMR Spectrum (500.23, 125.79 MHz, $CDCl_3$) of Compound 7

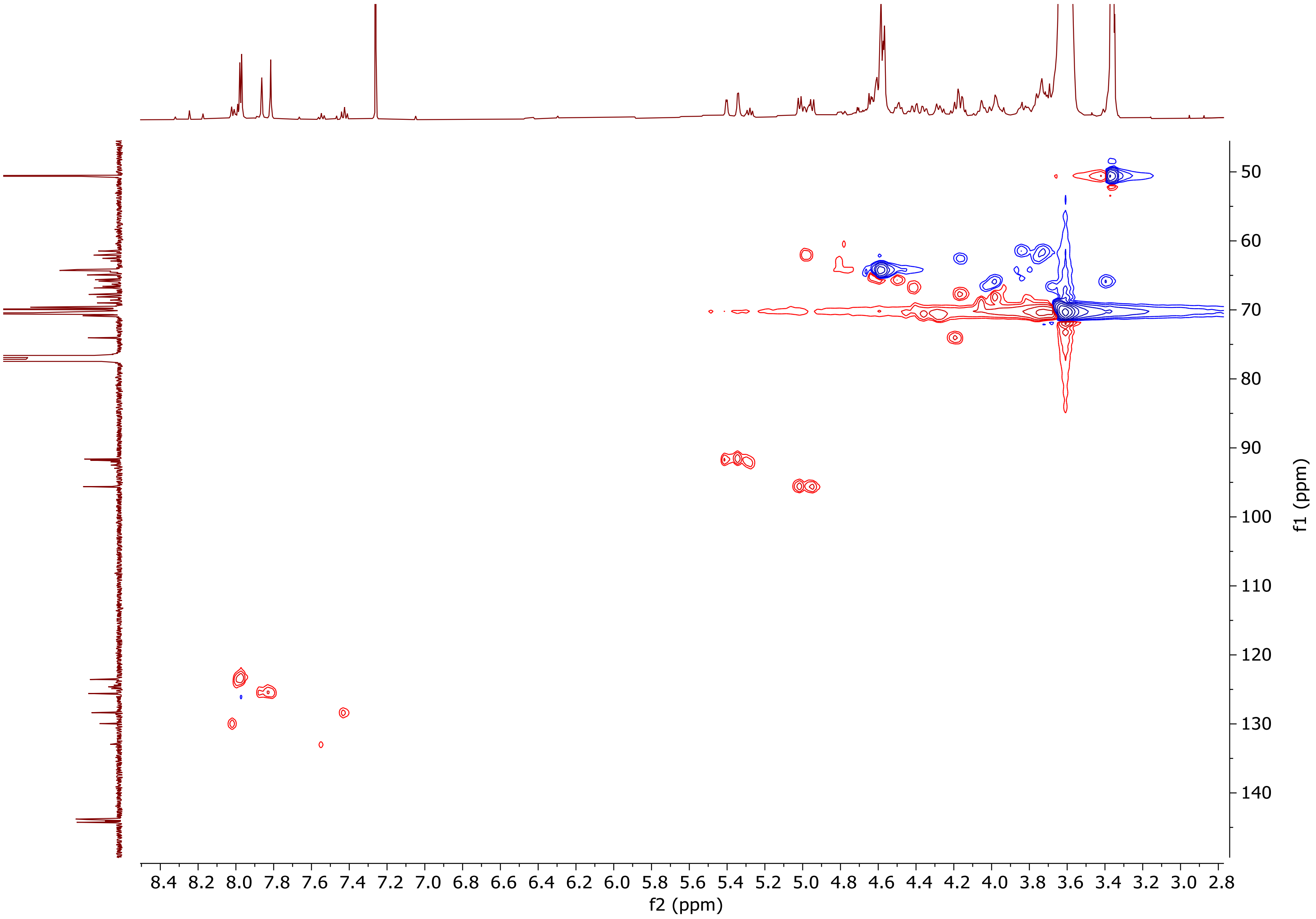

COSY NMR Spectrum (500.23 MHz, $CDCl_3$) of Compound 7

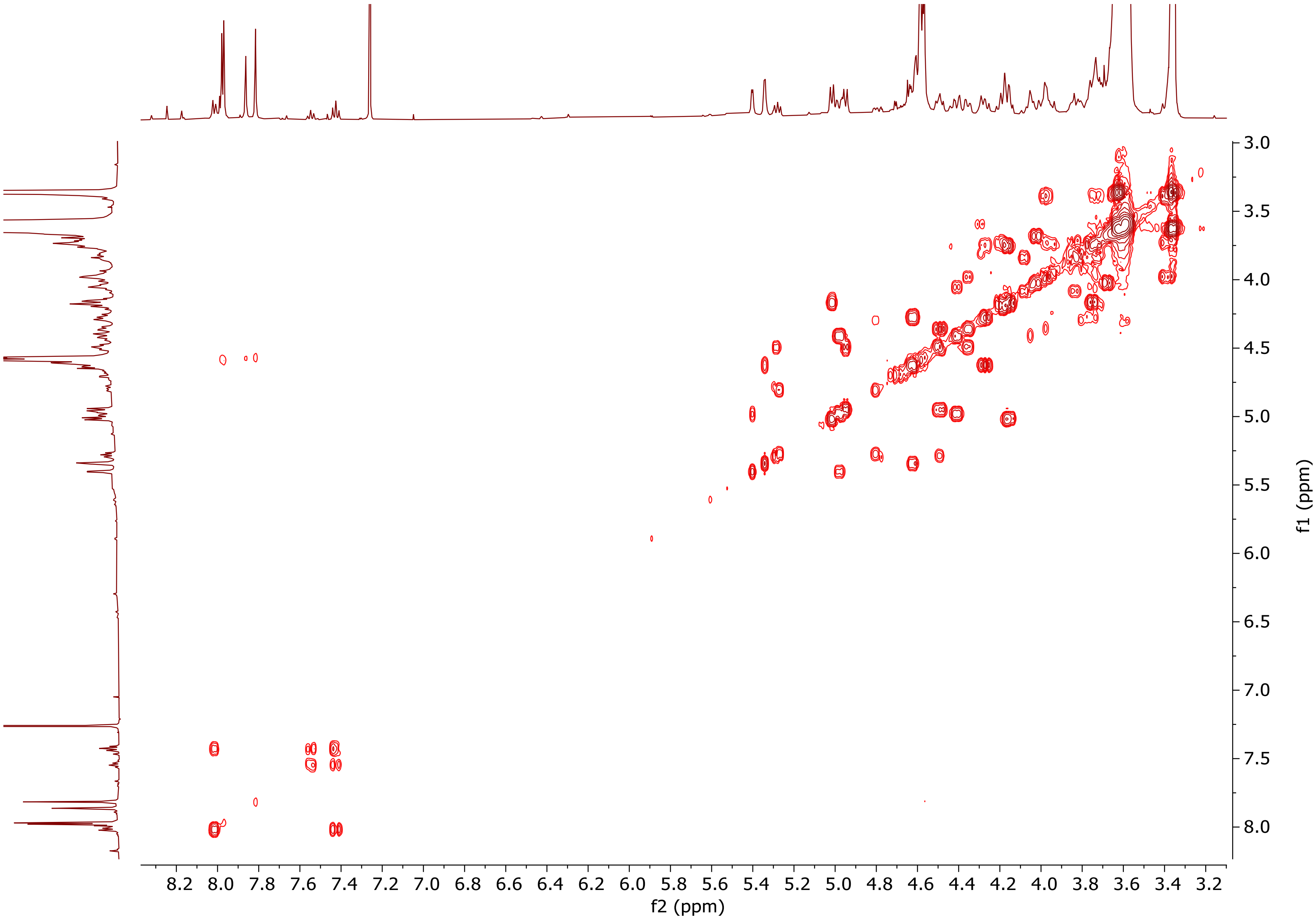

HMBC NMR Spectrum (500.23, 125.80 MHz, $CDCl_3$) of Compound 7

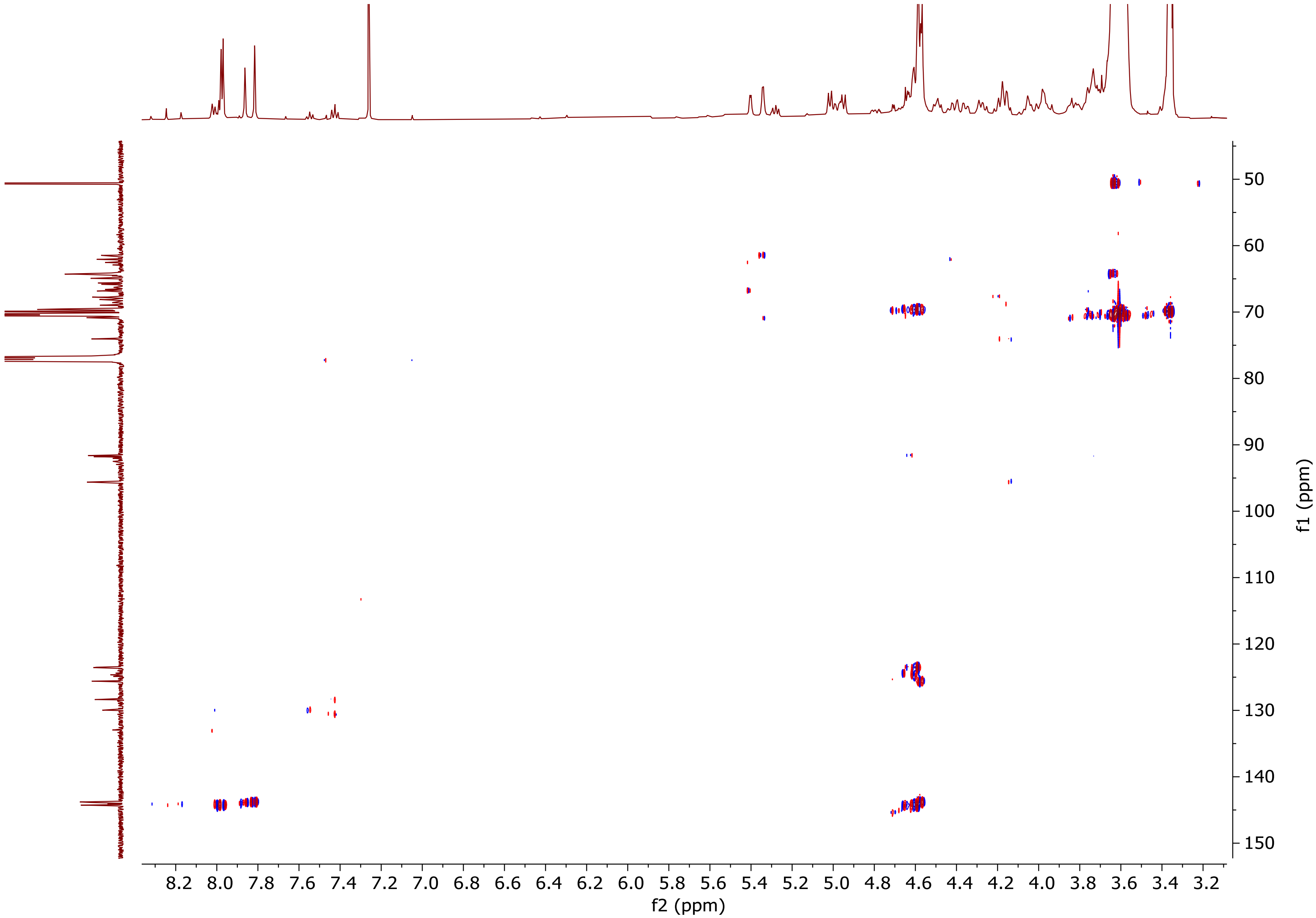

^{1}H NMR Spectrum (400.34 MHz, CDCl$_3$) of Compound 5

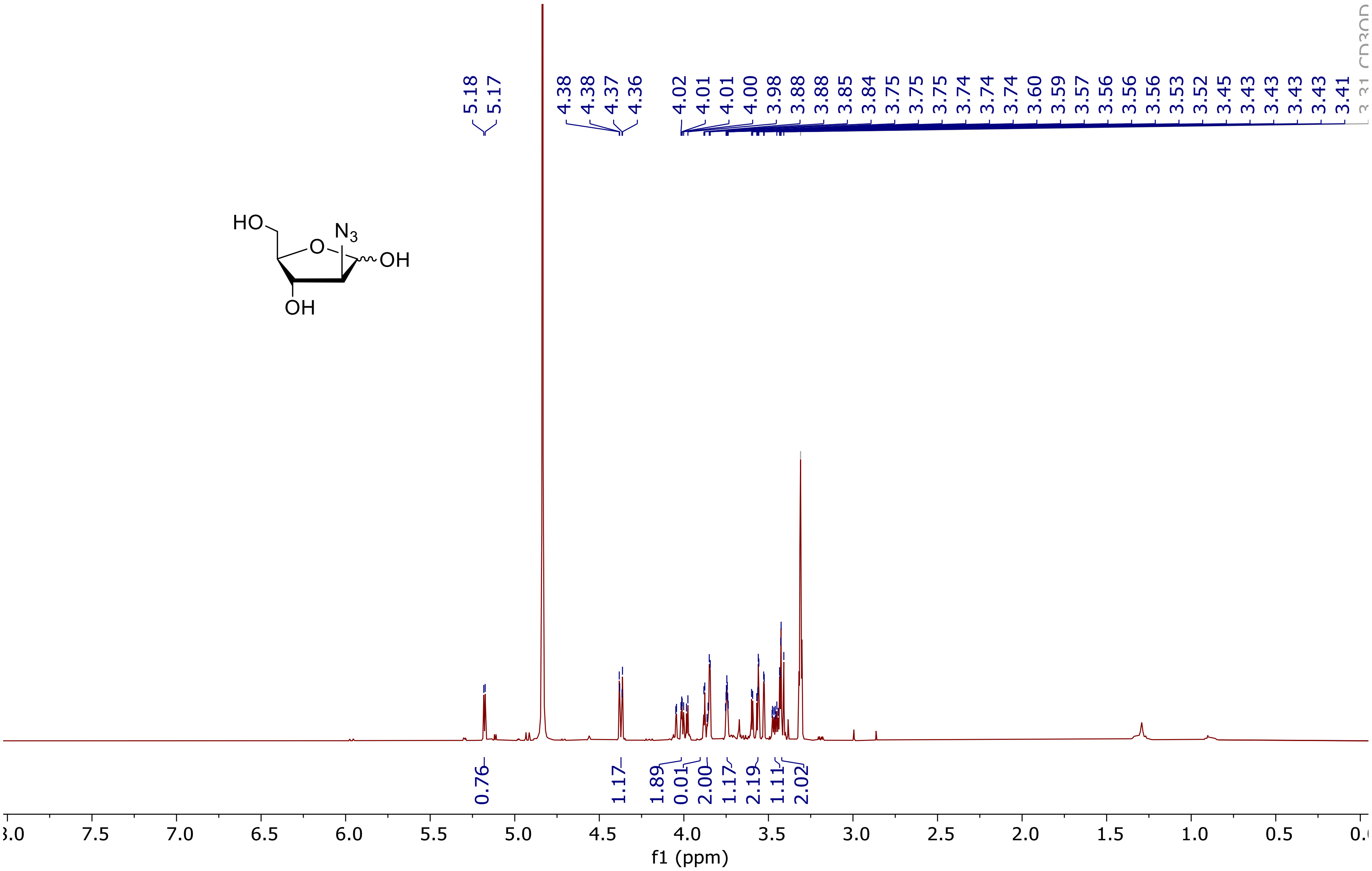

^{13}C NMR Spectrum (100.68 MHz, $CDCl_3$) of Compound 5

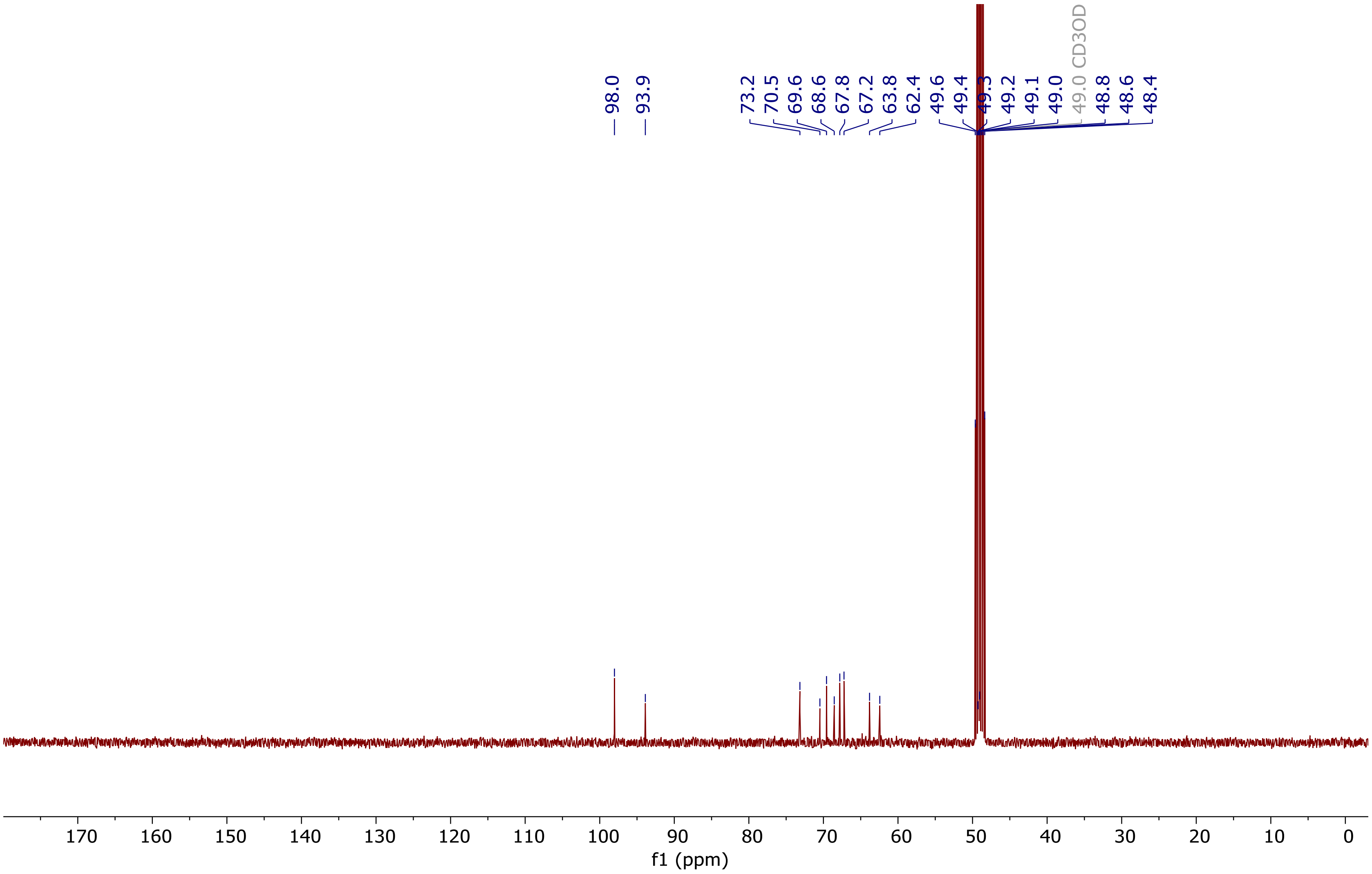

DEPT NMR Spectrum (100.67 MHz, $CDCl_3$) of Compound 5

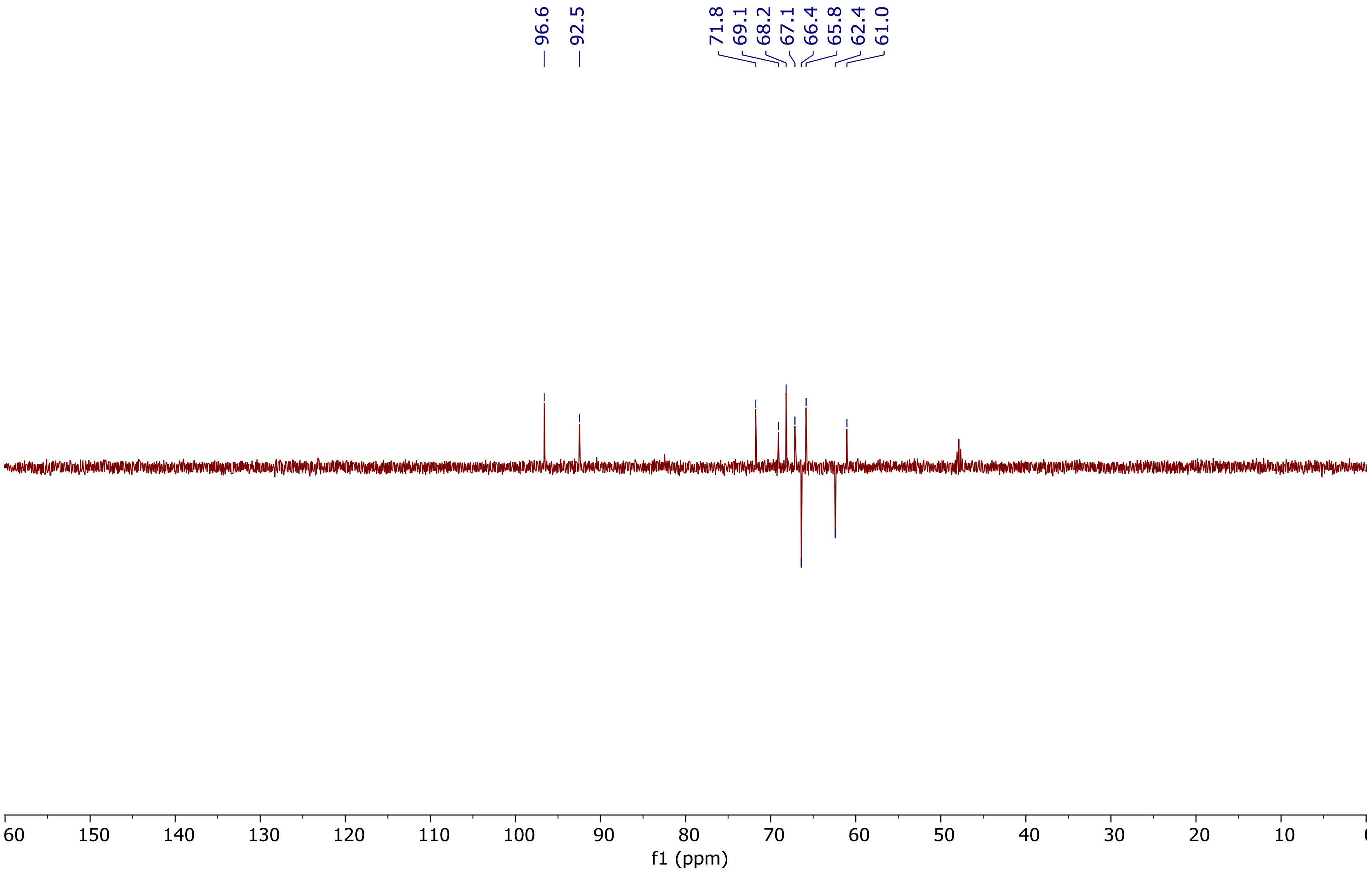

HSQC NMR Spectrum (400.34, 100.67 MHz, $CDCl_3$) of Compound 5

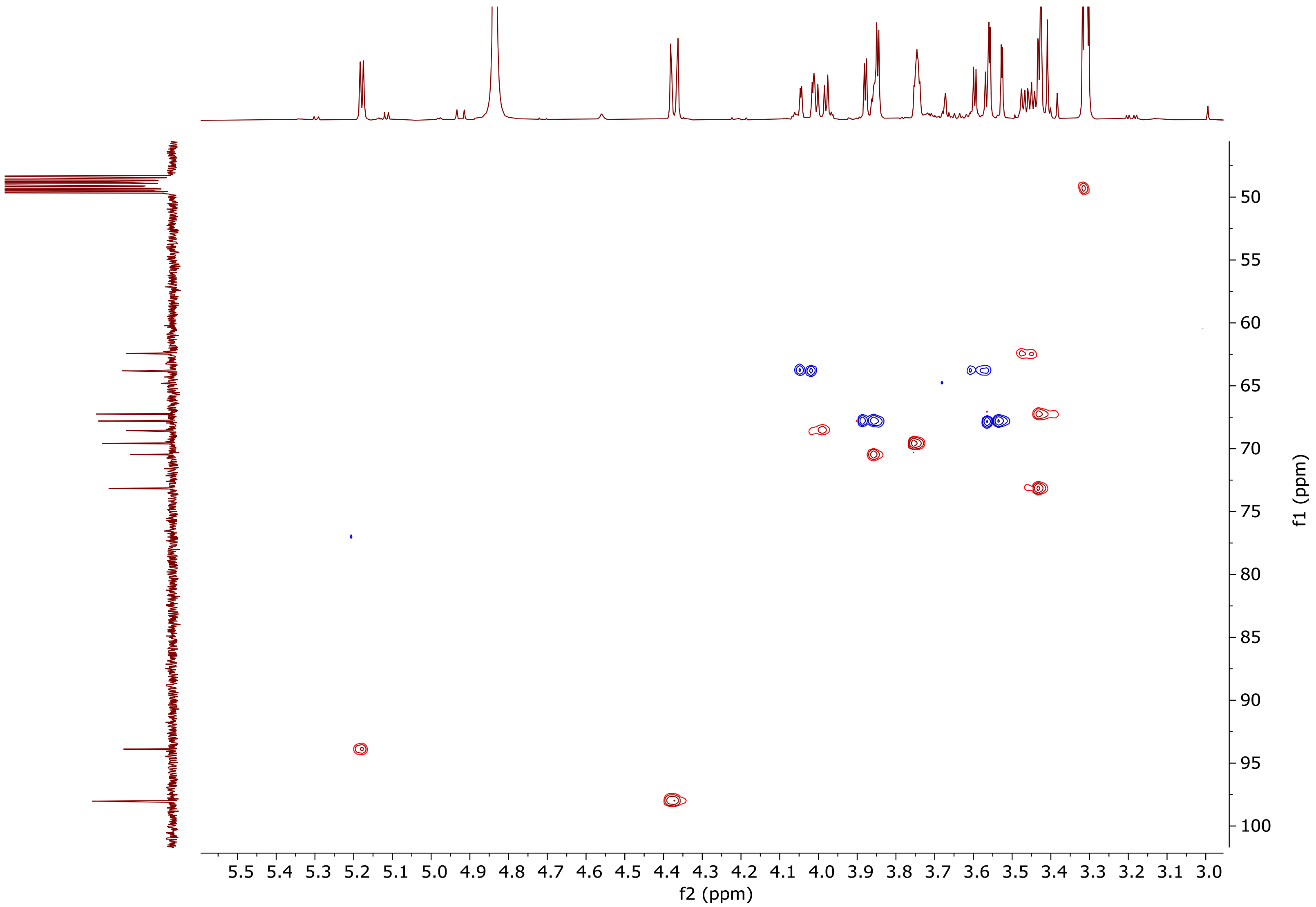

COSY NMR Spectrum (400.34 MHz, $CDCl_3$) of Compound 5

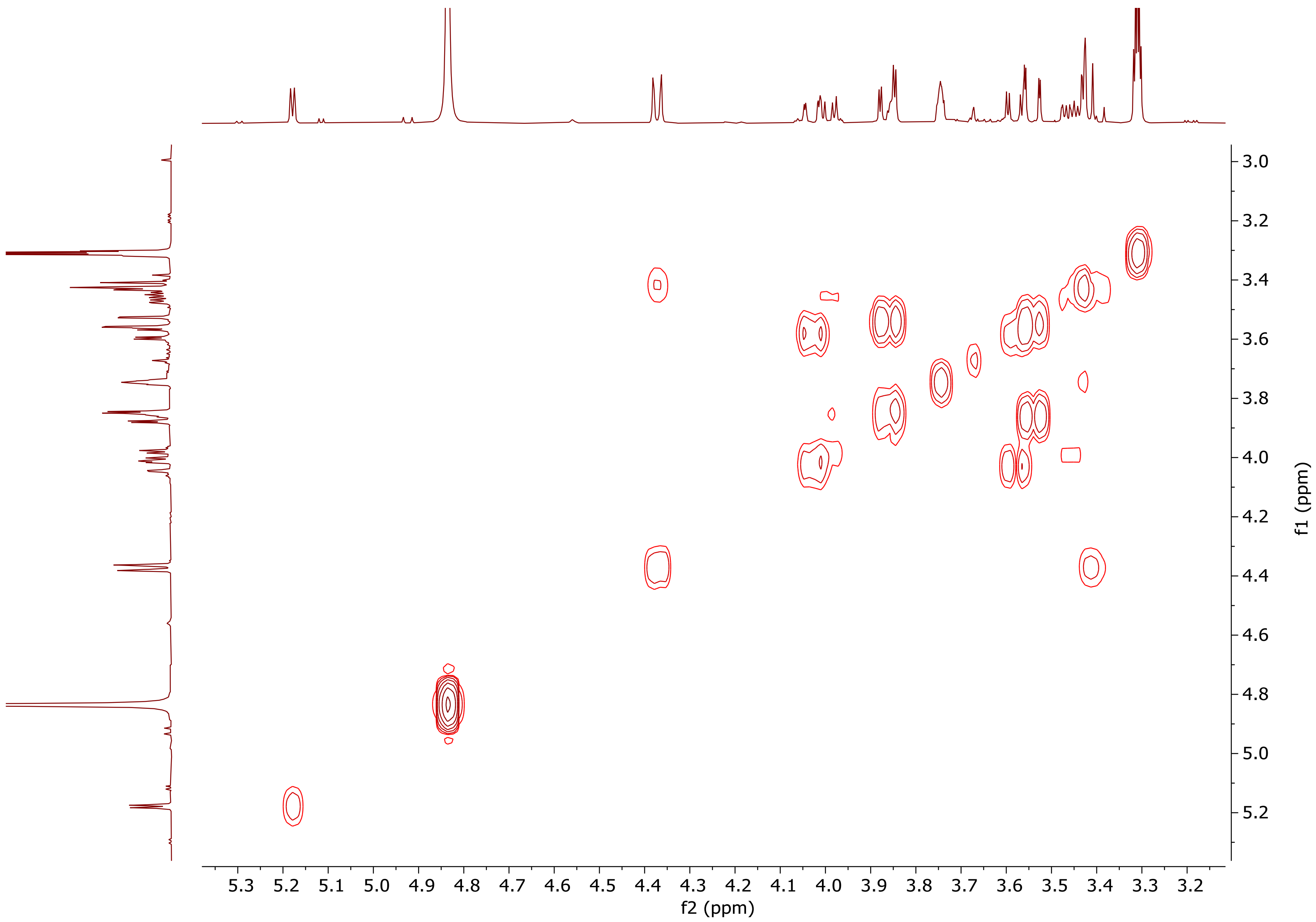

HMBC NMR Spectrum (400.34, 100.68 MHz, $CDCl_3$) of Compound 5

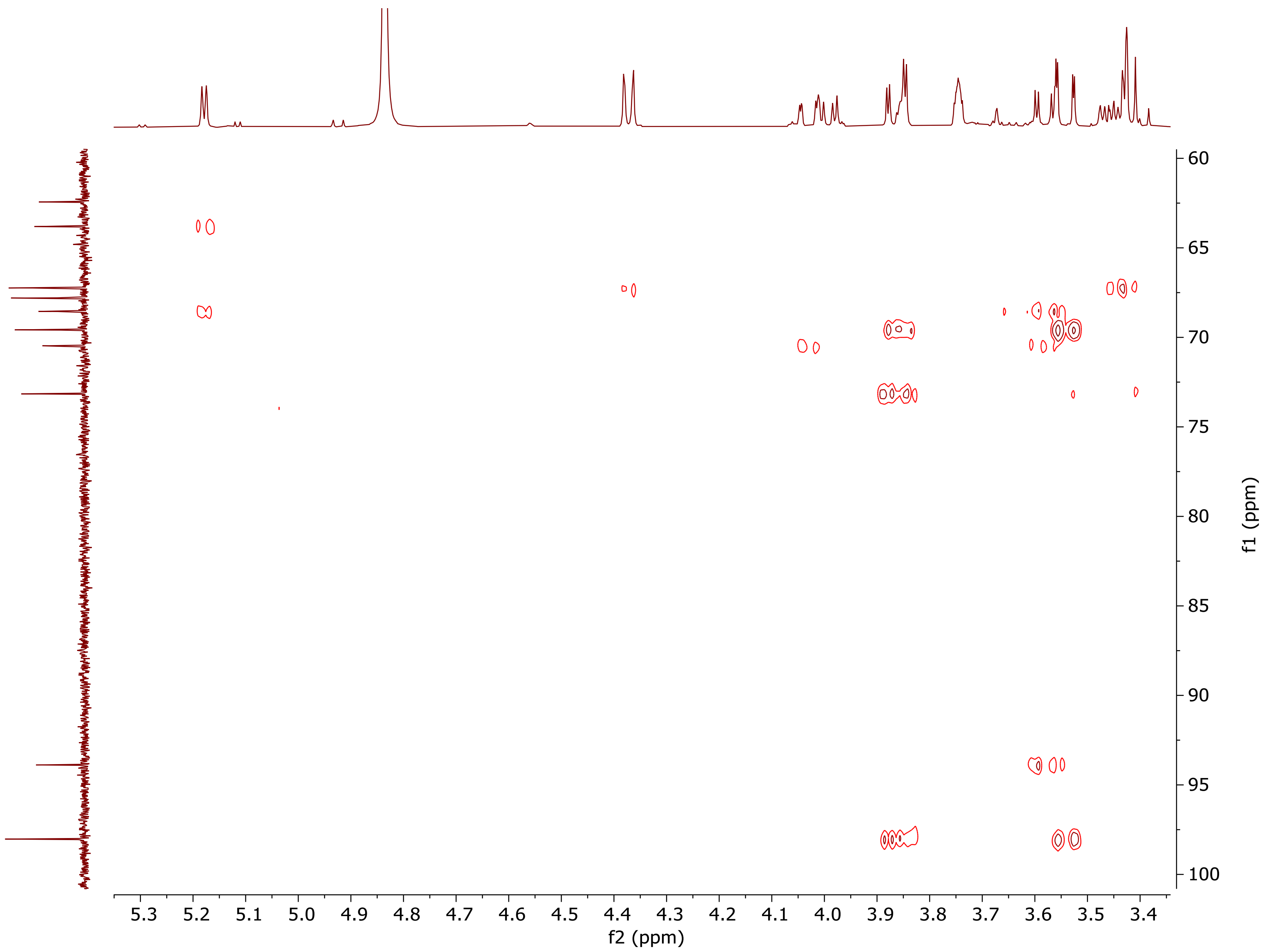

^{1}H NMR Spectrum (400.34 MHz, $CDCl_3$) of Compound 6a

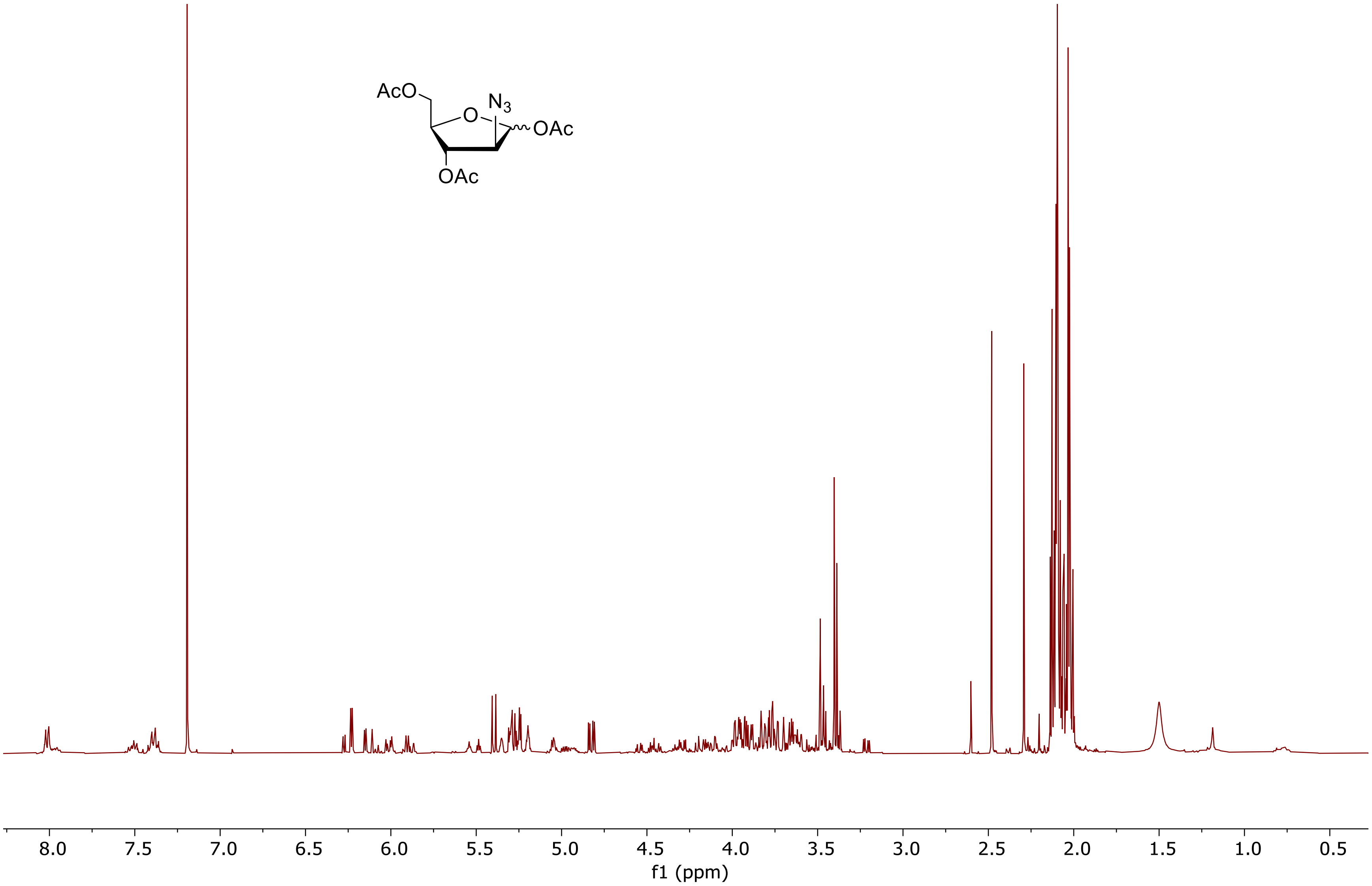

COSY NMR Spectrum (400.34 MHz, $CDCl_3$) of Compound 6a

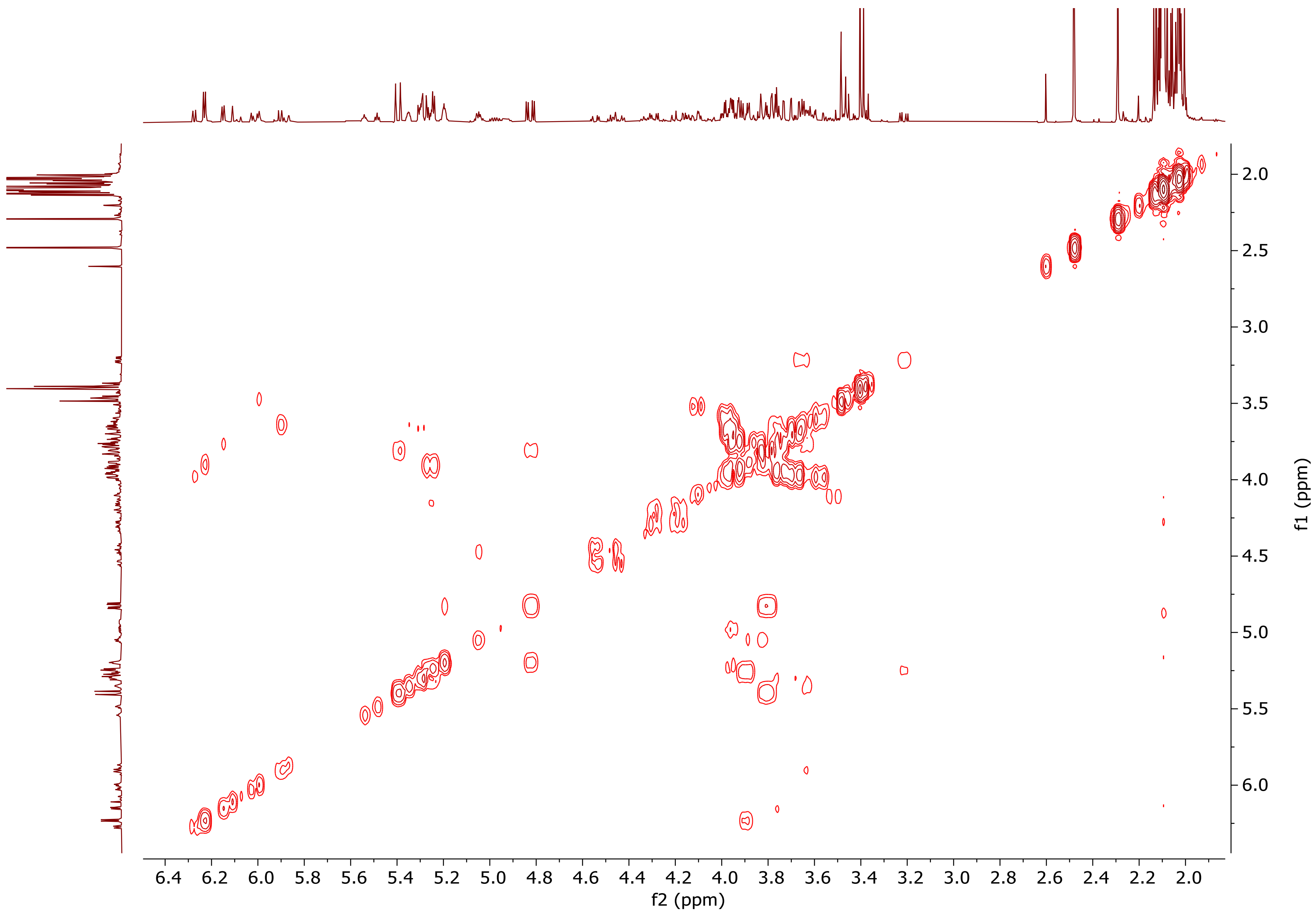

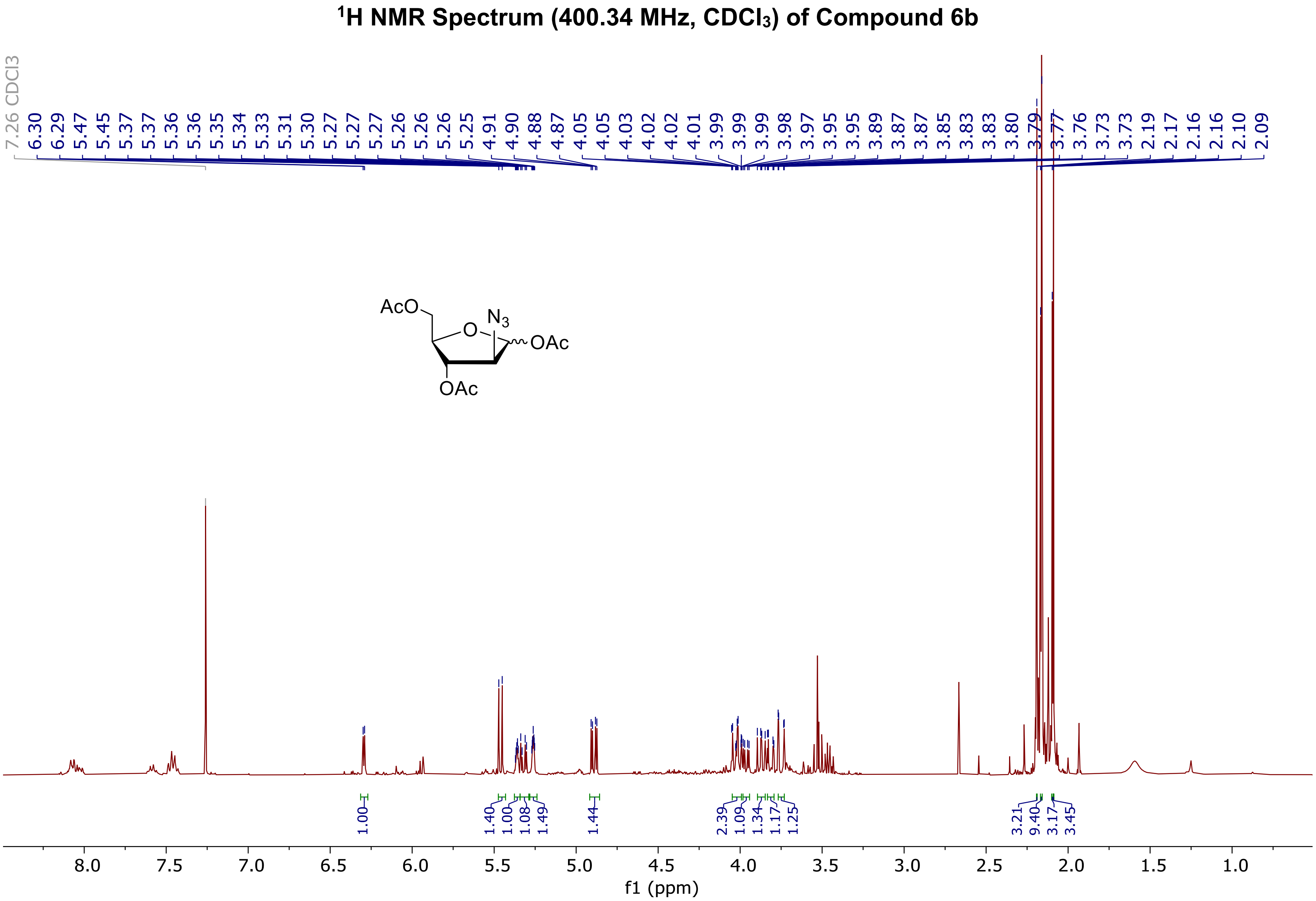

^{1}H NMR Spectrum (400.34 MHz, CDCl3) of Compound 6b
7.26 CDCl3
6.30 6.29 5.47 5.45 5.37 5.37 5.36 5.36 5.35 5.34 5.33 5.31 5.30 5.27 5.27 5.27 5.26 5.26 5.26 5.25 4.91 4.90 4.88 4.87 4.05 4.05 4.03 4.02 4.02 4.01 3.99 3.99 3.99 3.98 3.97 3.95 3.95 3.89 3.87 3.87 3.85 3.83 3.83 3.80 3.79 3.77 3.76 3.73 3.73 2.19 2.17 2.16 2.16 2.10 2.09
AcO
O
N3
OAc
OAc
1.00 1.40 1.00 1.08 1.49 1.44 2.39 1.09 1.34 1.17 1.25 3.21 9.40 3.17 3.45
8.0 7.5 7.0 6.5 6.0 5.5 5.0 4.5 4.0 3.5 3.0 2.5 2.0 1.5 1.0
f1 (ppm)

^{13}C NMR Spectrum (100.68 MHz, $CDCl_3$) of Compound 6b

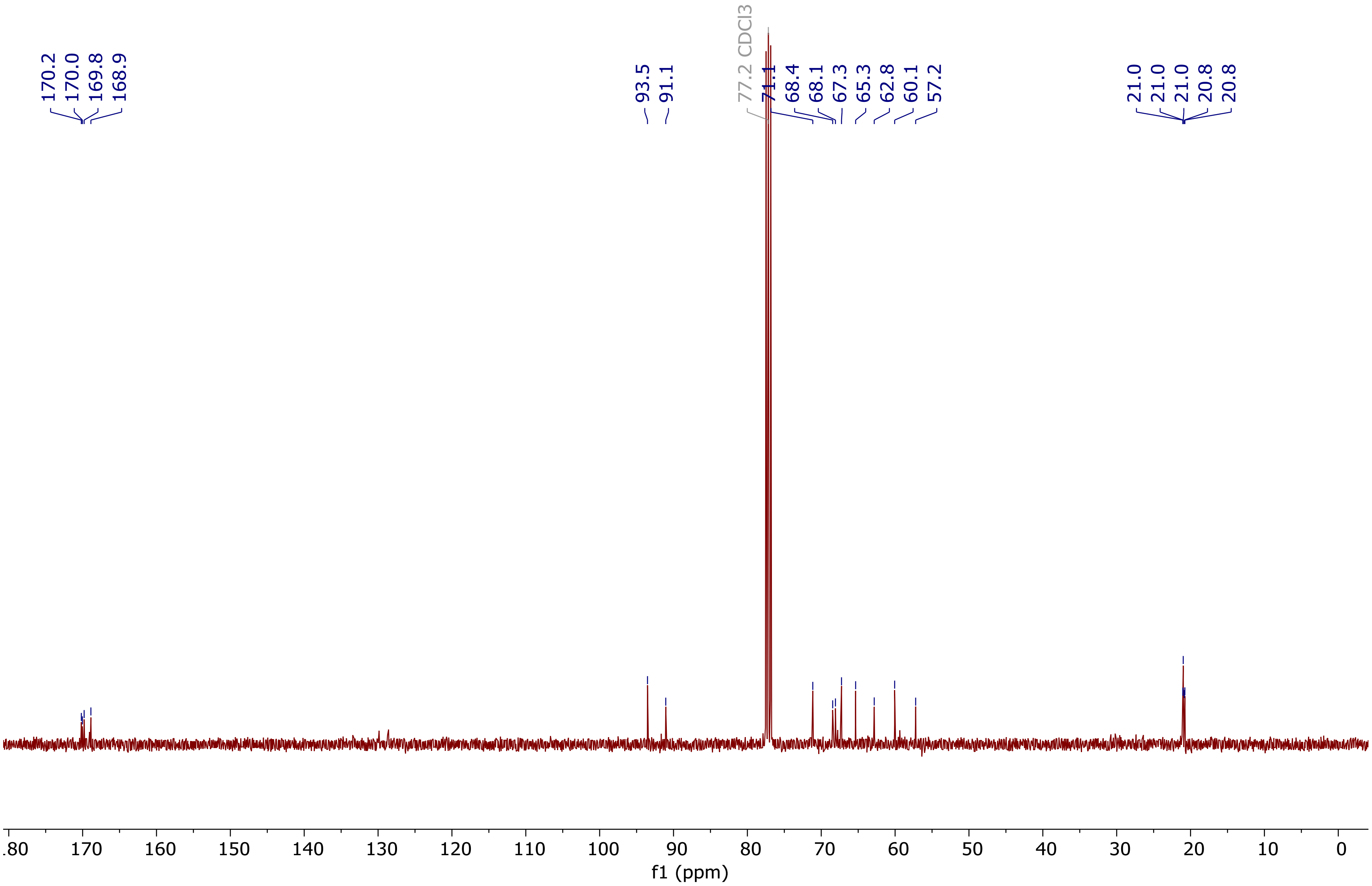

HSQC NMR Spectrum (400.34, 100.67 MHz, $CDCl_3$) of Compound 6b

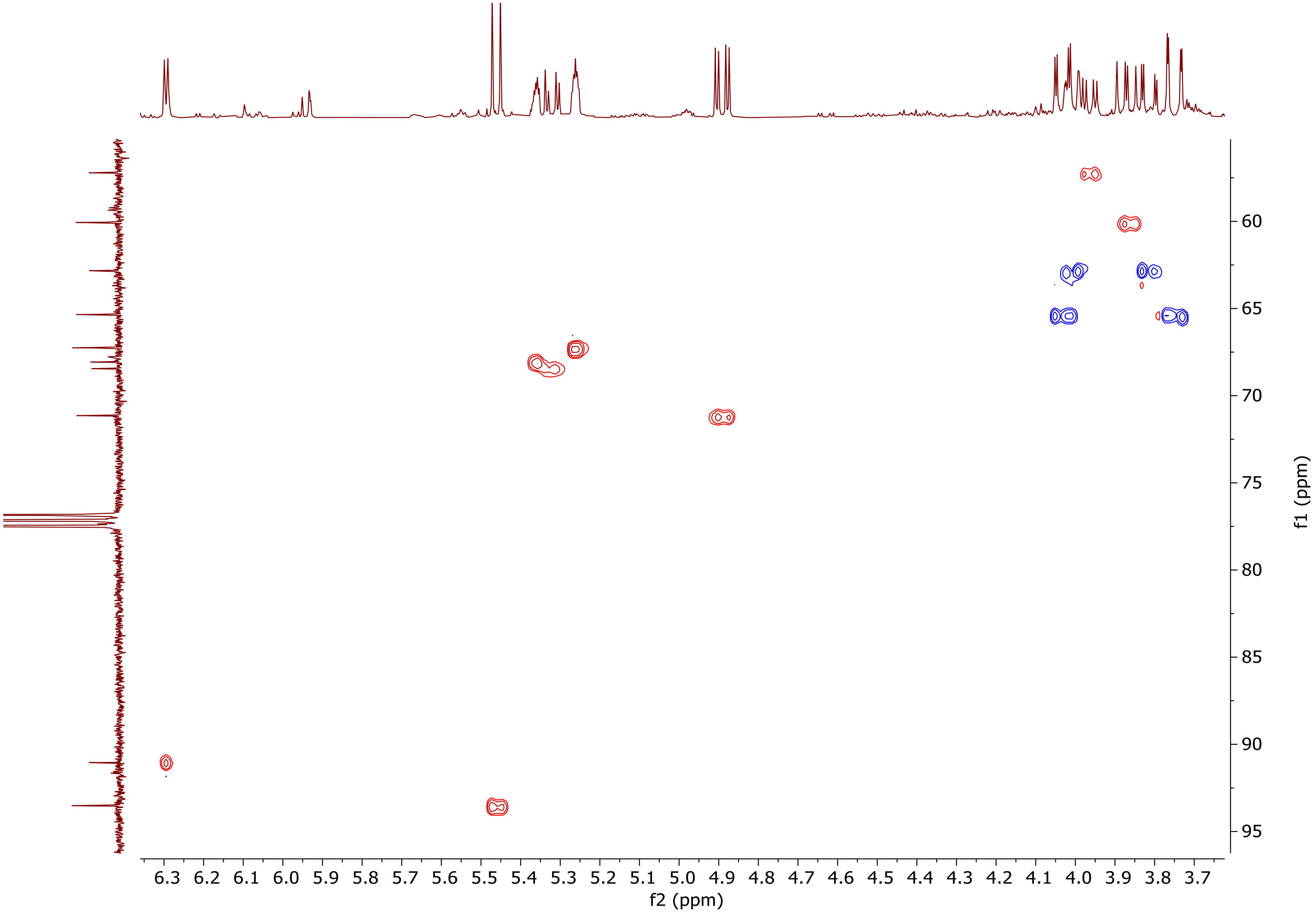

COSY NMR Spectrum (400.34 MHz, $CDCl_3$) of Compound 6b

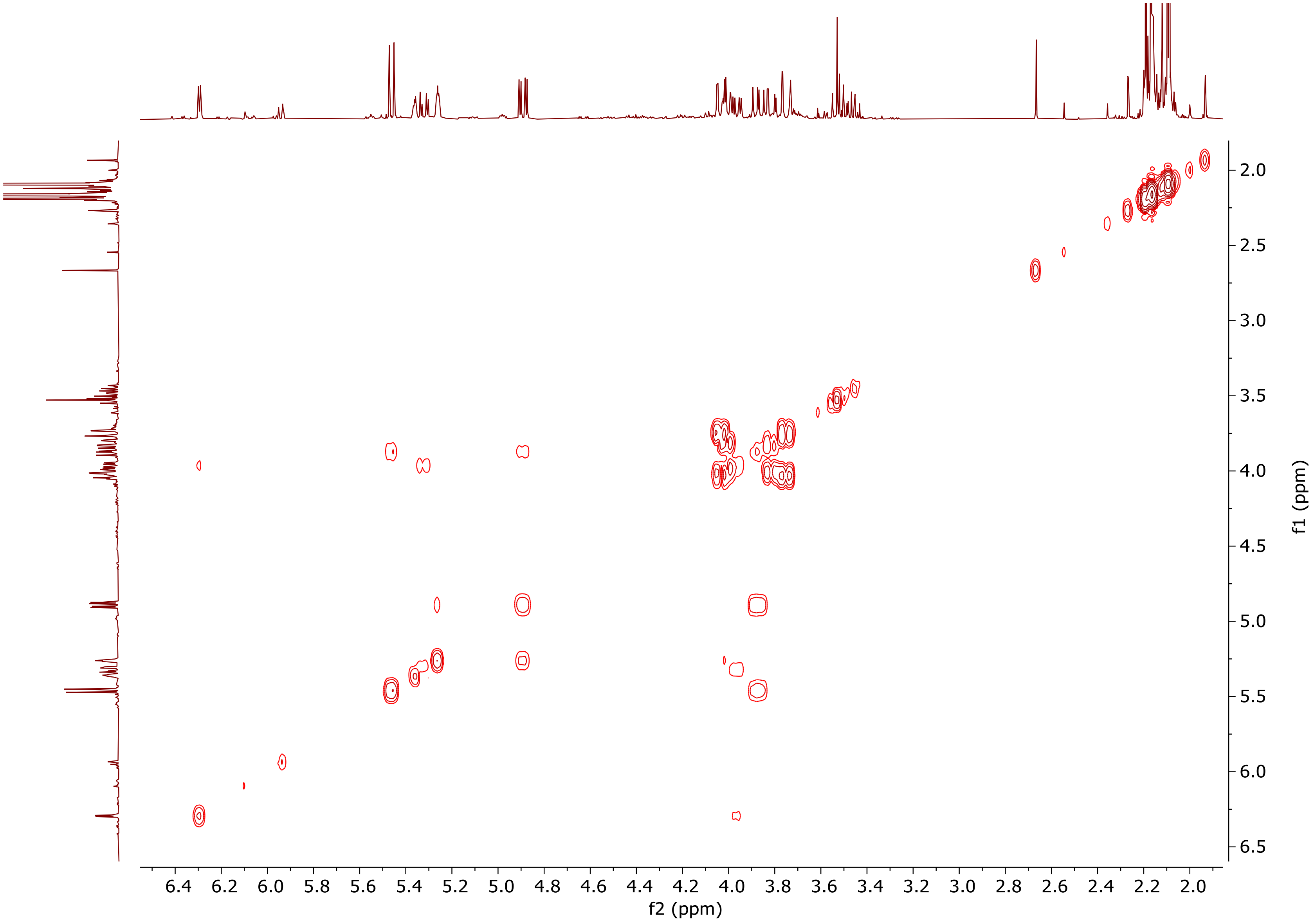

HMBC NMR Spectrum (400.34, 100.68 MHz, $CDCl_3$) of Compound 6b

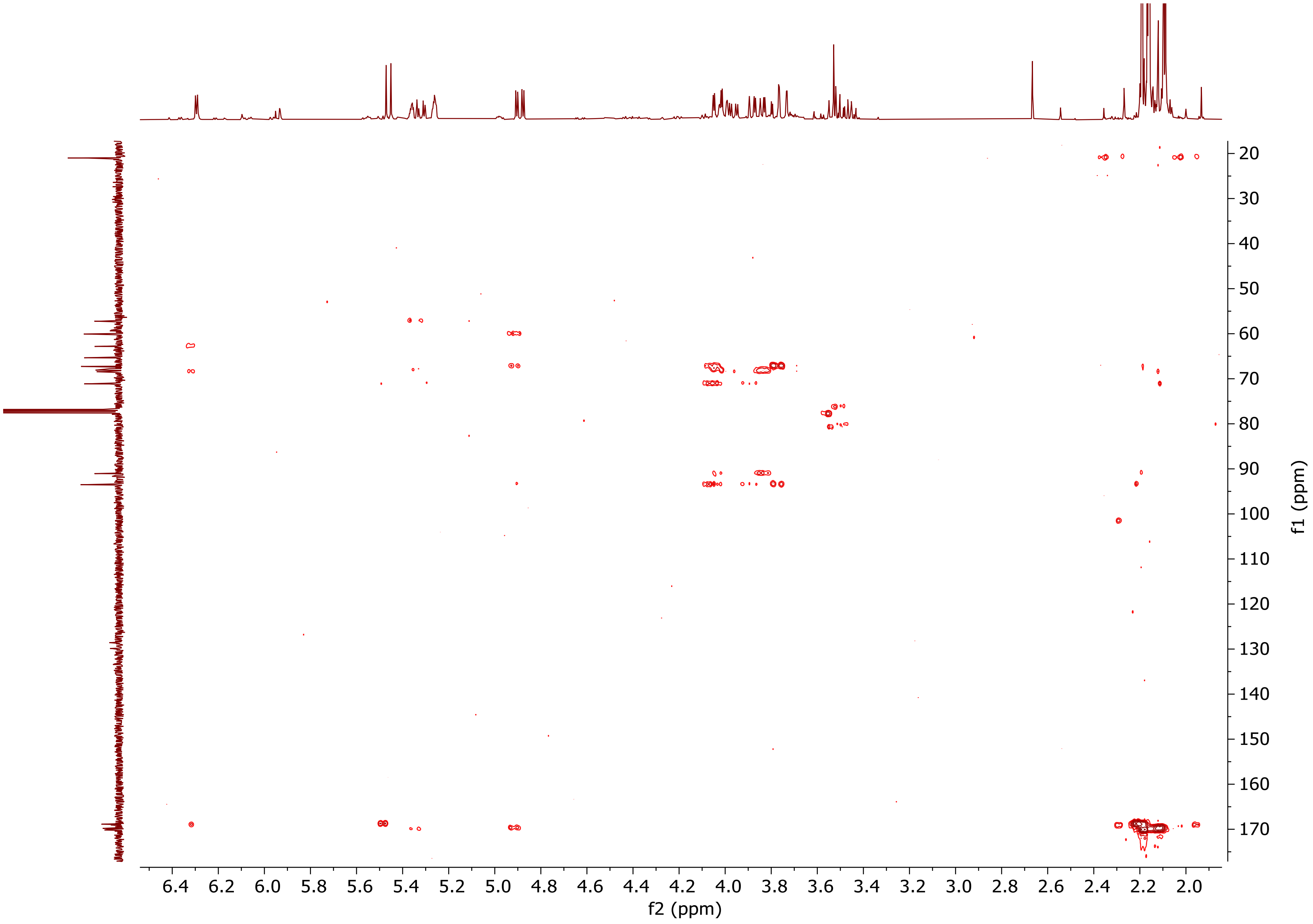

^{1}H NMR Spectrum (500.23 MHz, $CDCl_3$) of Compound 8

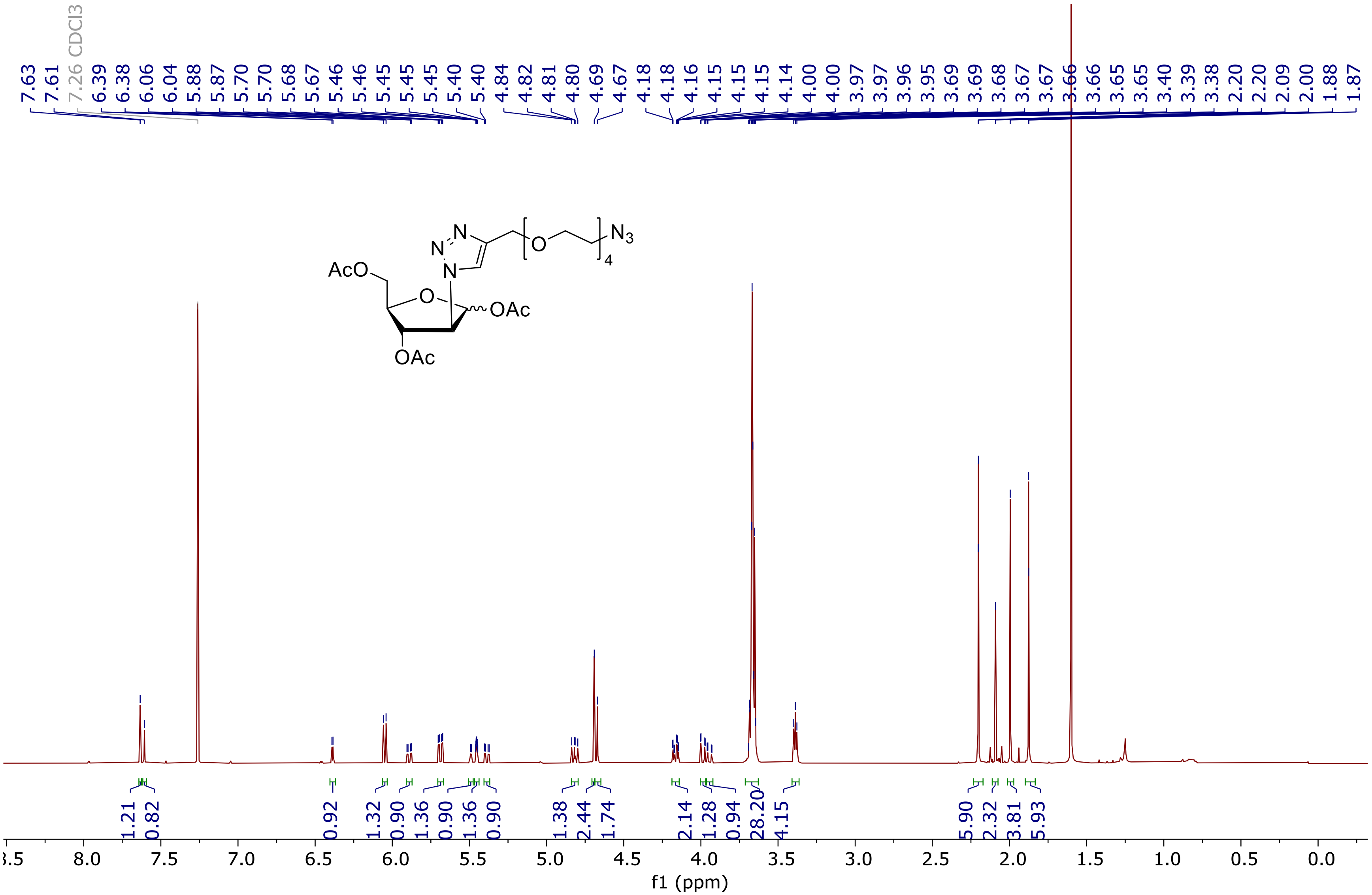

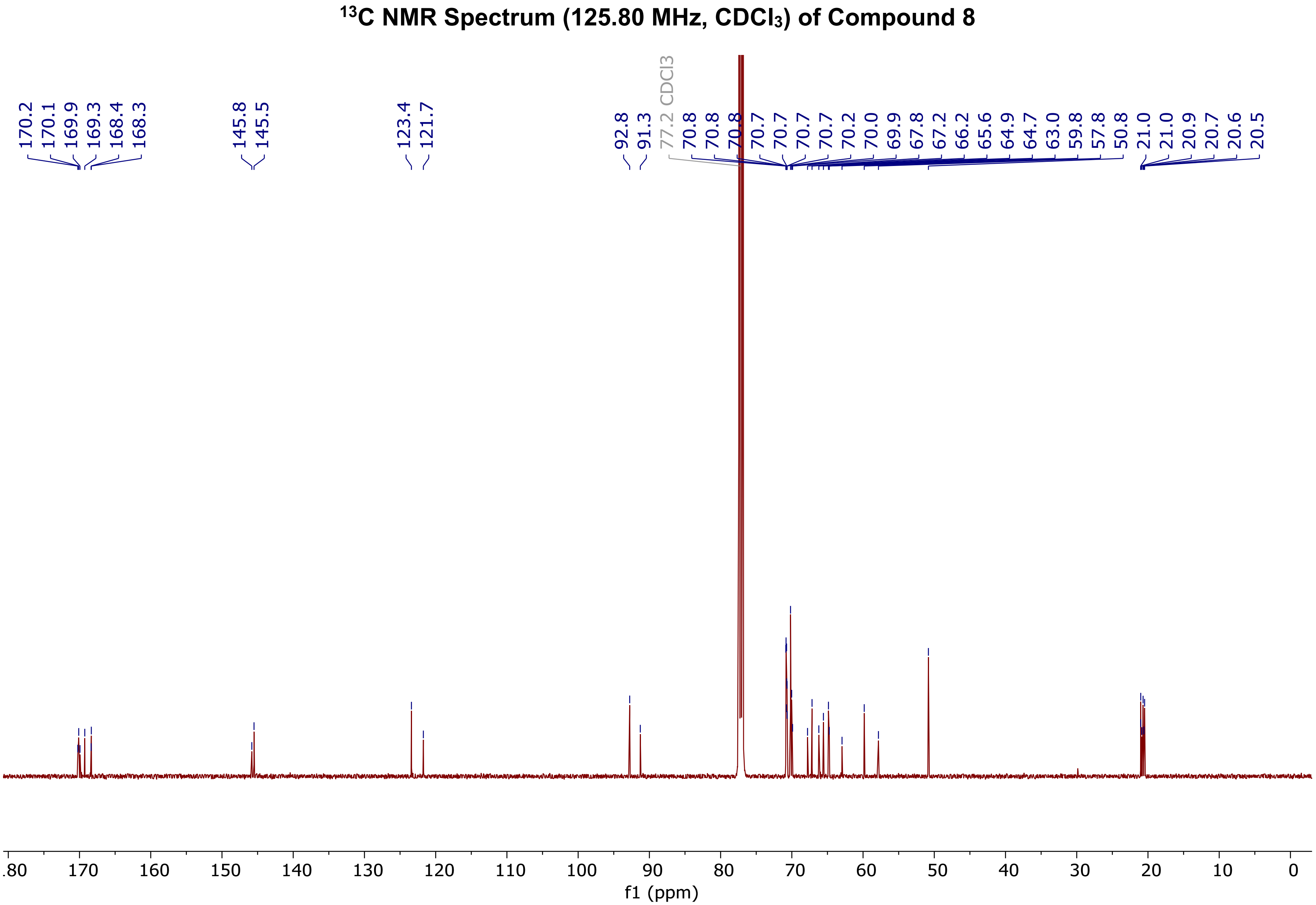

13C NMR Spectrum (125.80 MHz, CDCl3) of Compound 8
170.2
170.1
169.9
169.3
168.4
168.3
145.8
145.5
123.4
121.7
92.8
91.3
77.2 CDCl3
70.8
70.8
70.8
70.7
70.7
70.7
70.7
70.2
70.0
69.9
67.8
67.2
66.2
65.6
64.9
64.7
63.0
59.8
57.8
50.8
21.0
21.0
20.9
20.7
20.6
20.5
f1 (ppm)

HSQC NMR Spectrum (500.23, 125.80 MHz, $CDCl_3$) of Compound 8

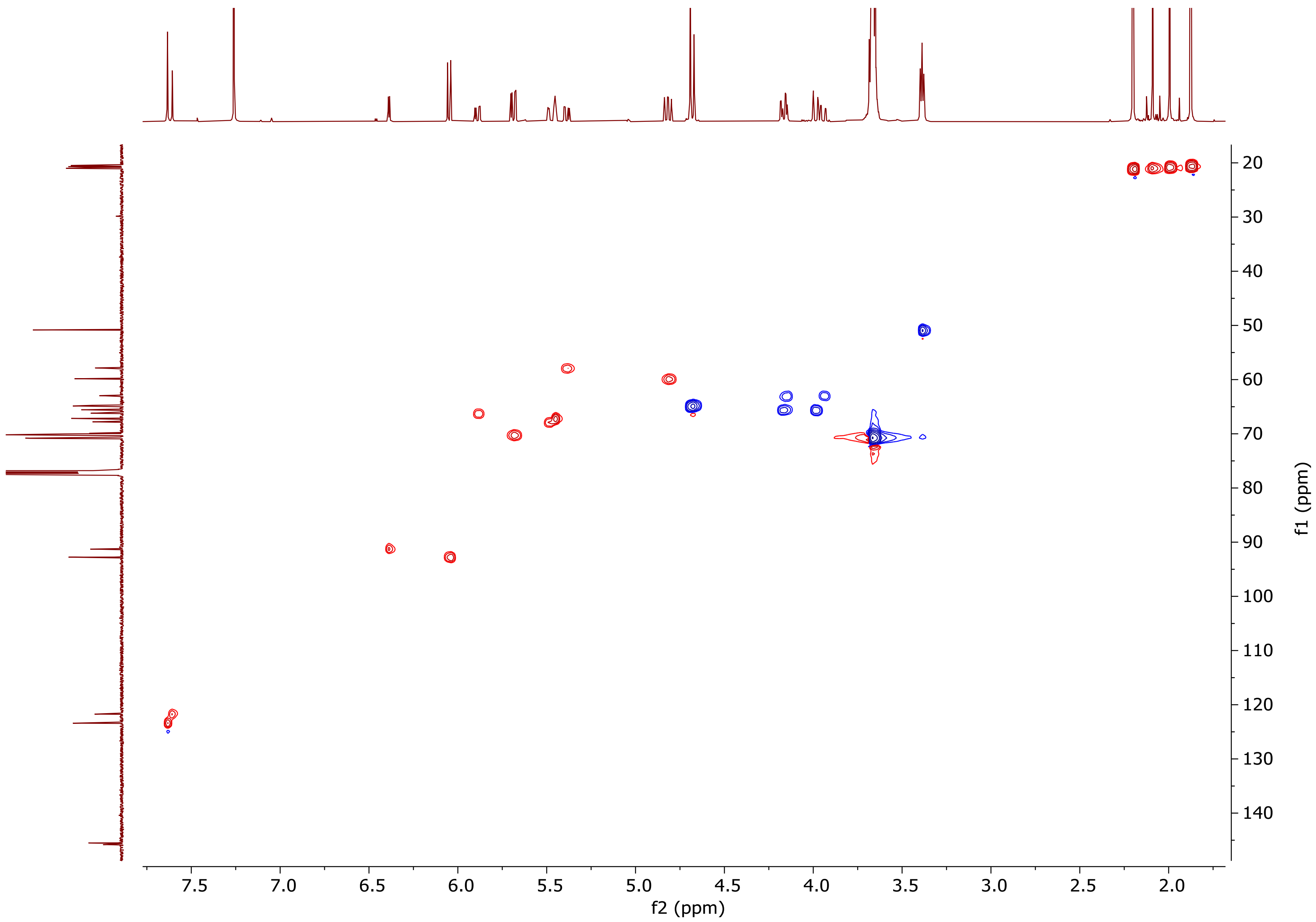

COSY NMR Spectrum (500.23 MHz, $CDCl_3$) of Compound 8

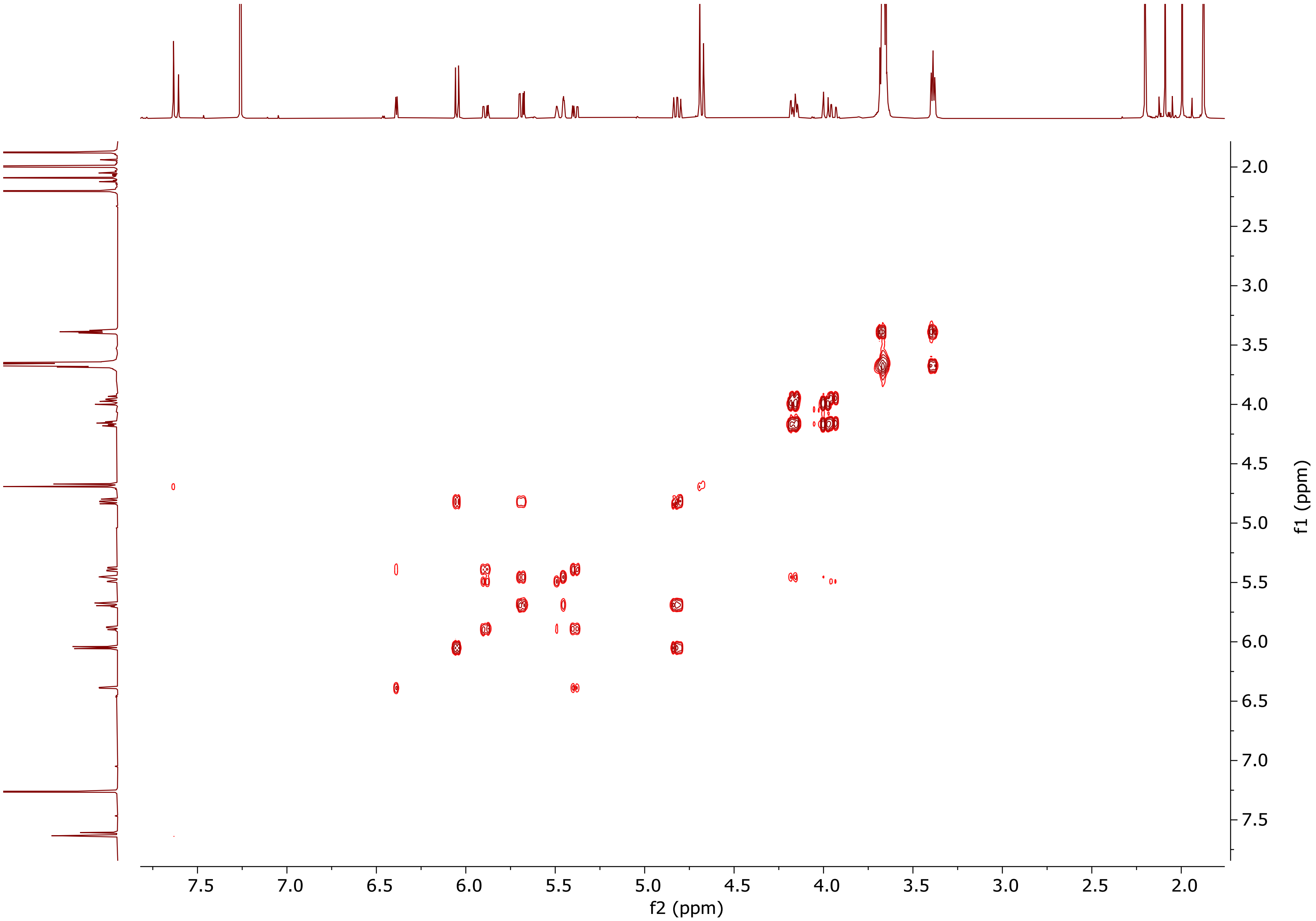

HMBC NMR Spectrum (500.23, 125.80 MHz, $CDCl_3$) of Compound 8

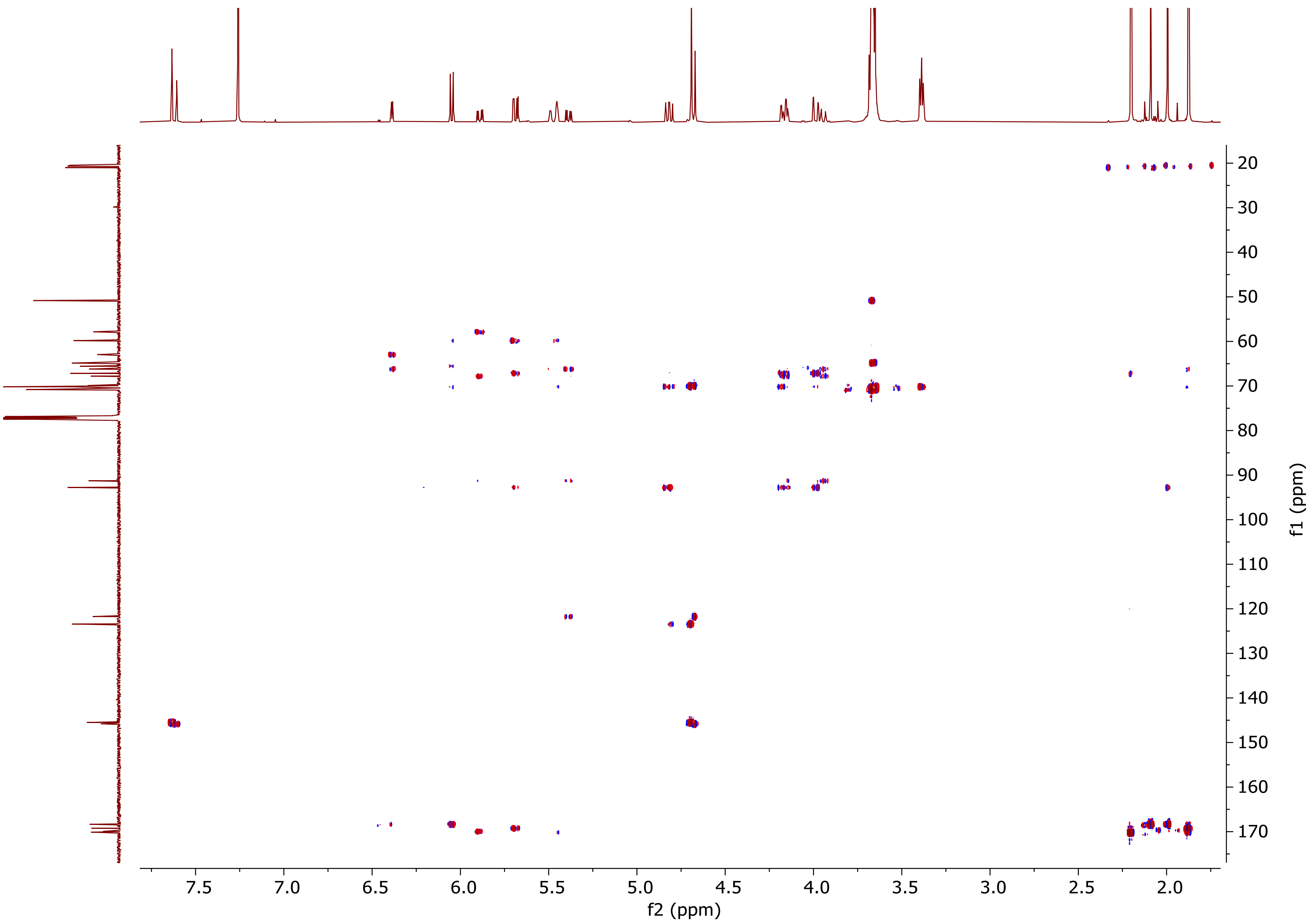

^{1}H NMR Spectrum (400.31 MHz, $CDCl_3$) of **Compound 23**

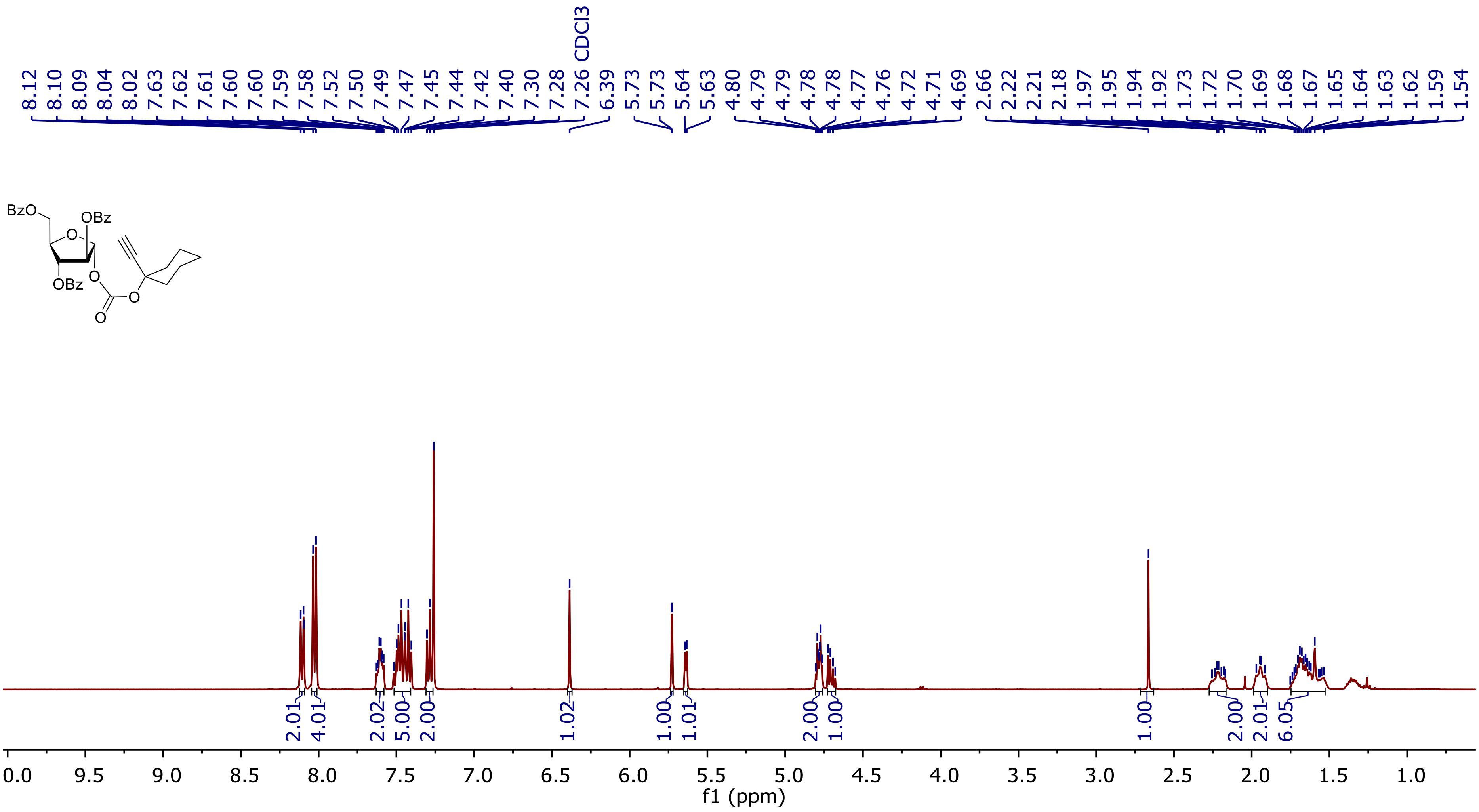

^{13}C NMR Spectrum (100.66 MHz, $CDCl_3$) of **Compound 23**

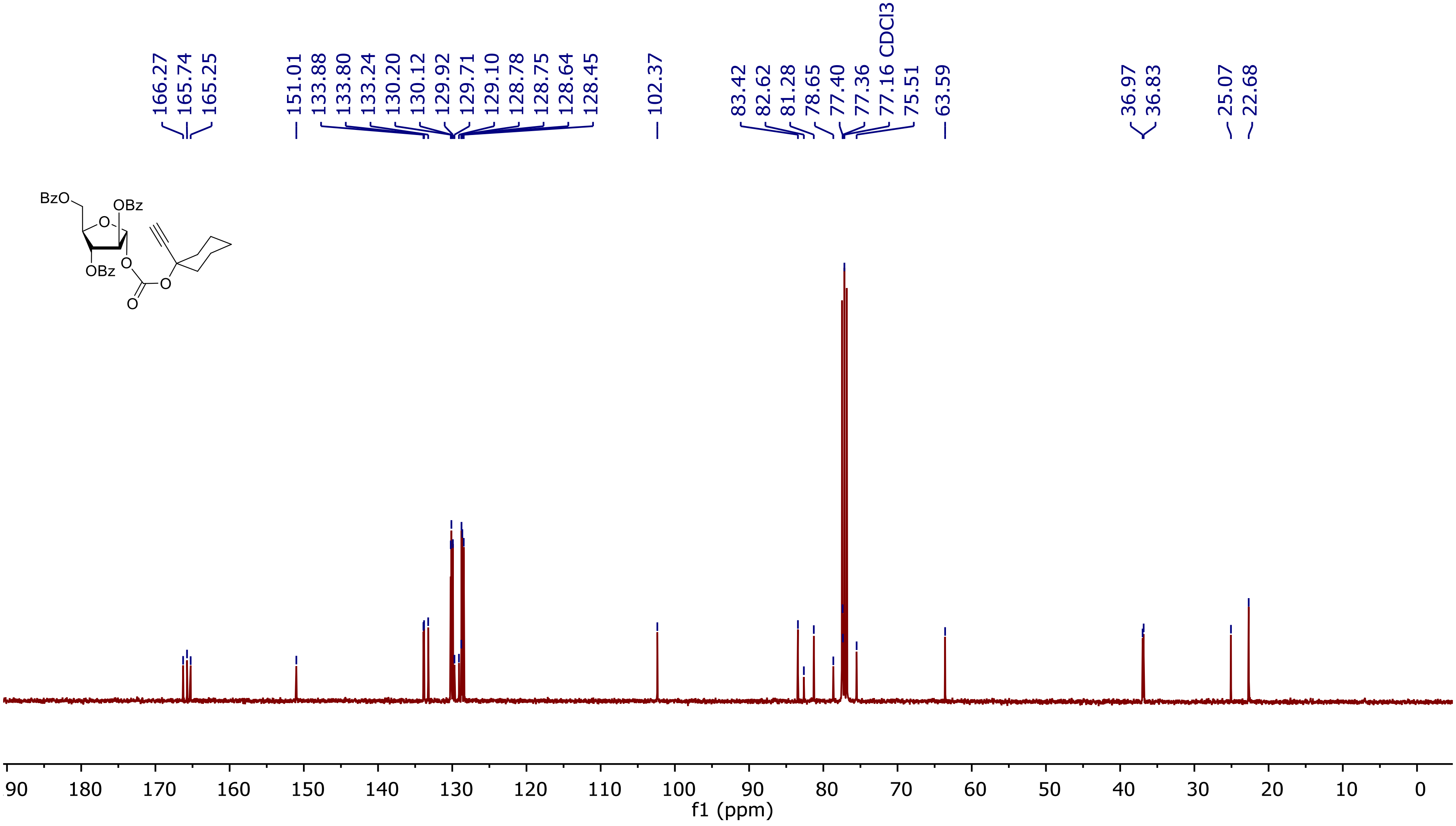

DEPT NMR Spectrum (100.66 MHz, $CDCl_3$) of **Compound 23**

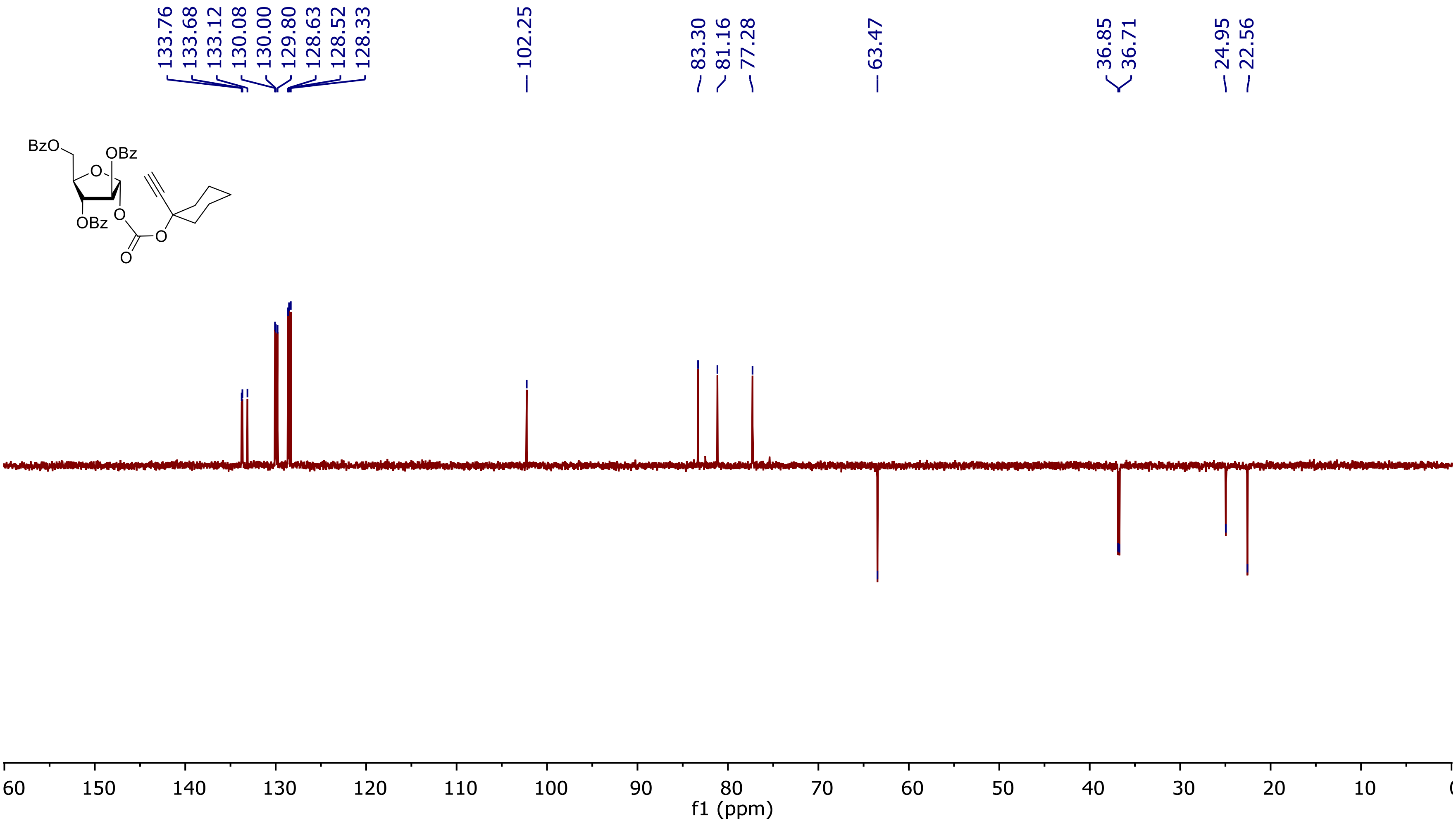

^{1}H NMR Spectrum (400.31 MHz, $CDCl_3$) of **Compound 25**

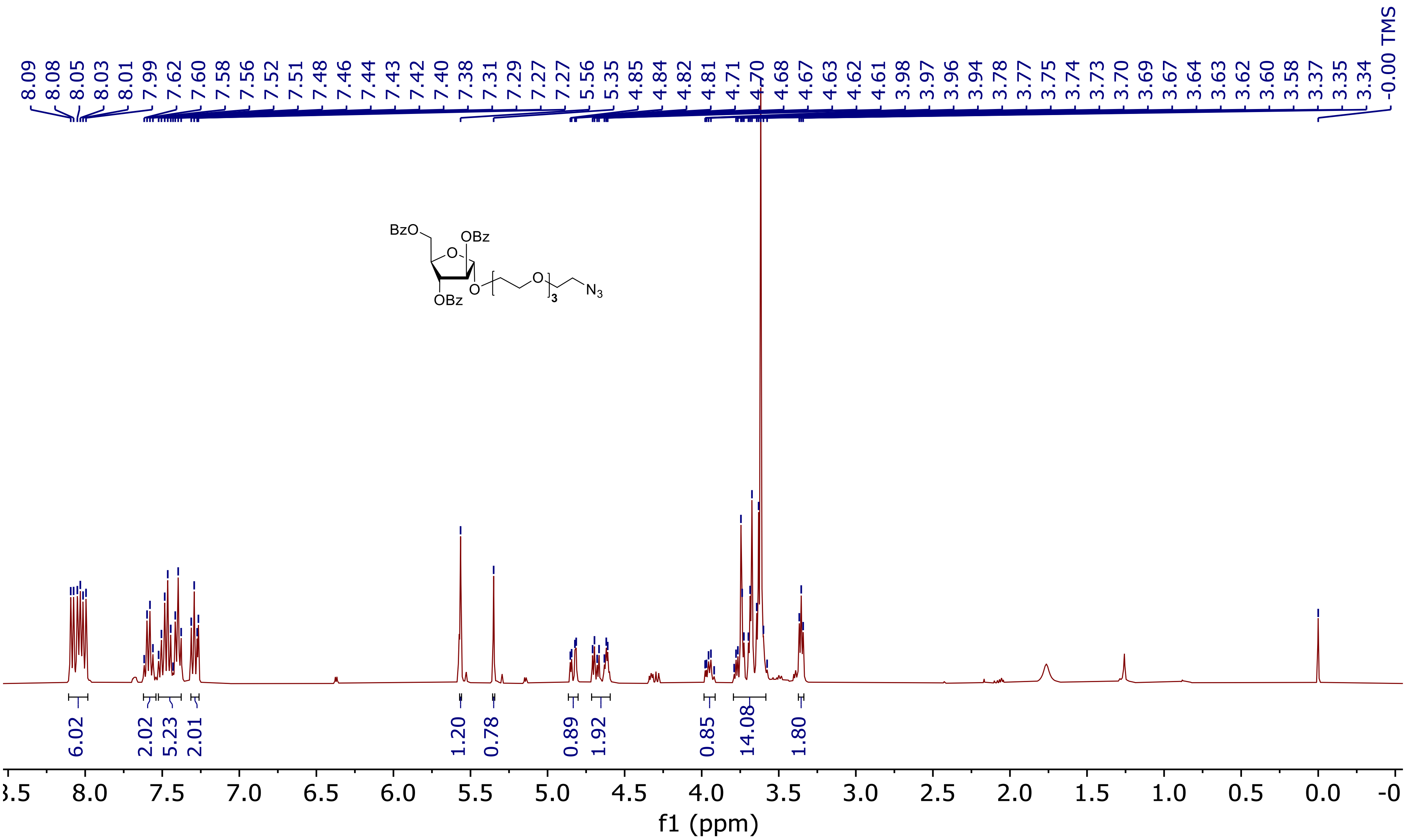

^{13}C NMR Spectrum (100.66 MHz, $CDCl_3$) of **Compound 25**

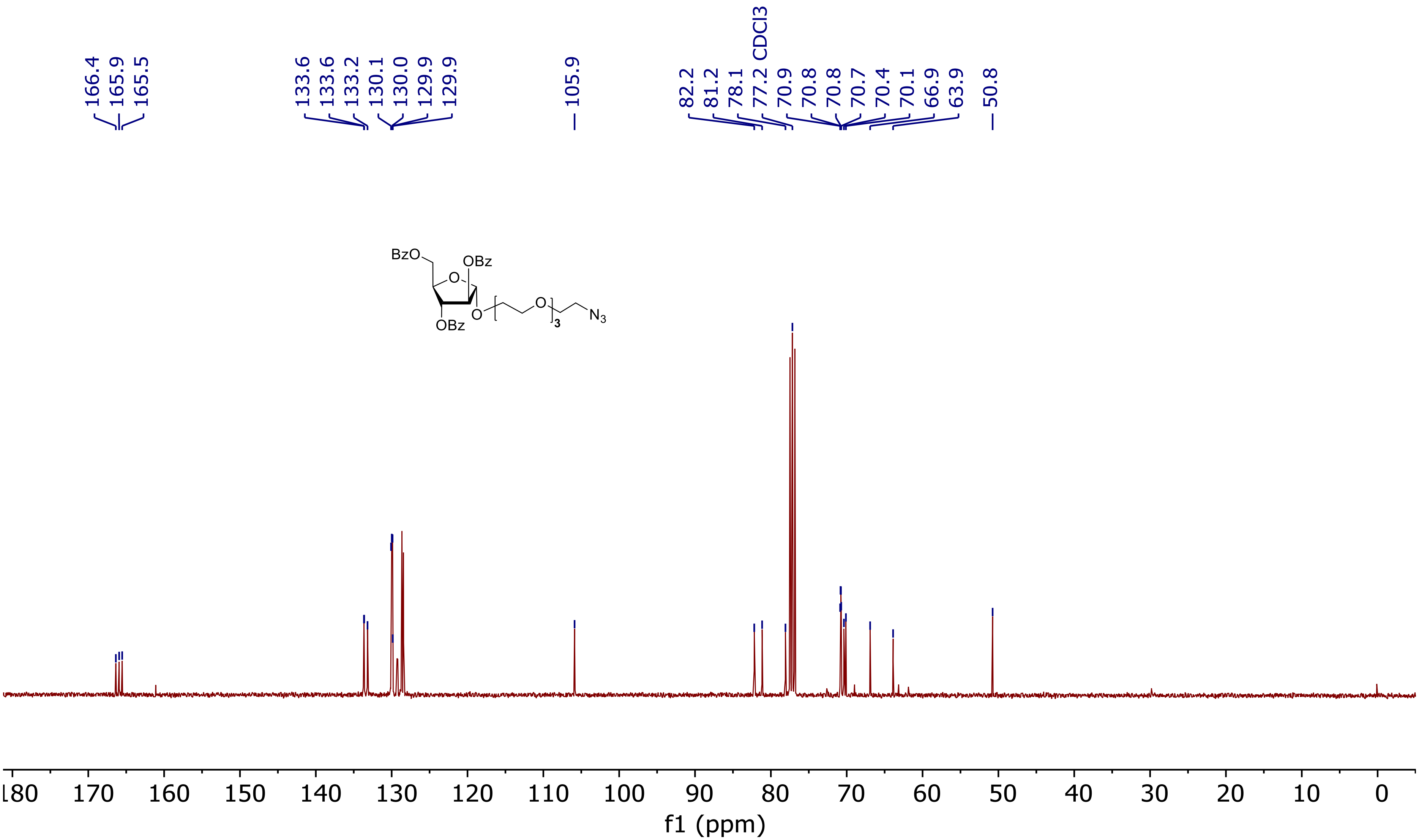

DEPT NMR Spectrum (100.66 MHz, $CDCl_3$) of **Compound 25**

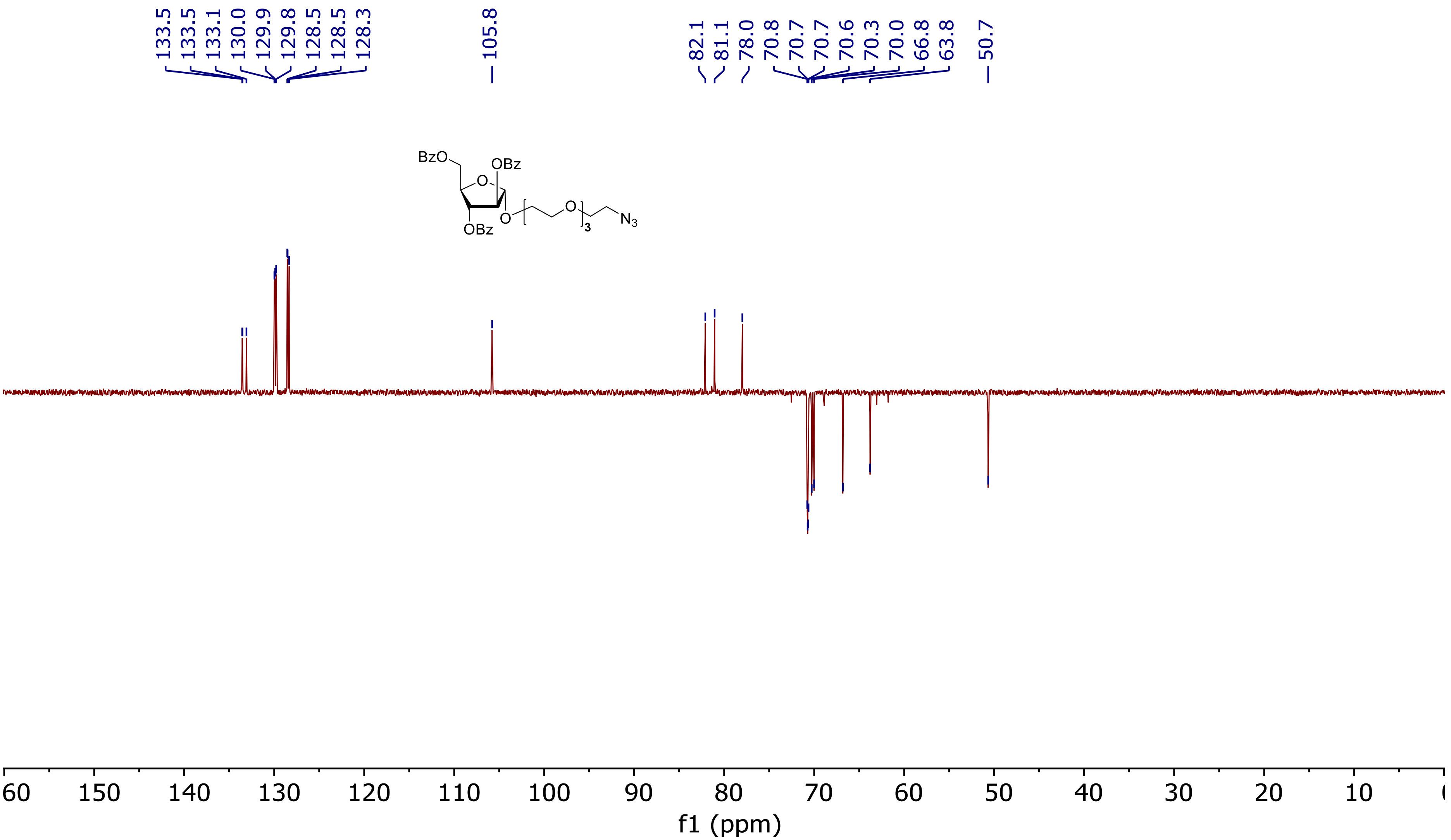

^{1}H NMR Spectrum (400.31 MHz, $CDCl_3$) of **Compound 10**

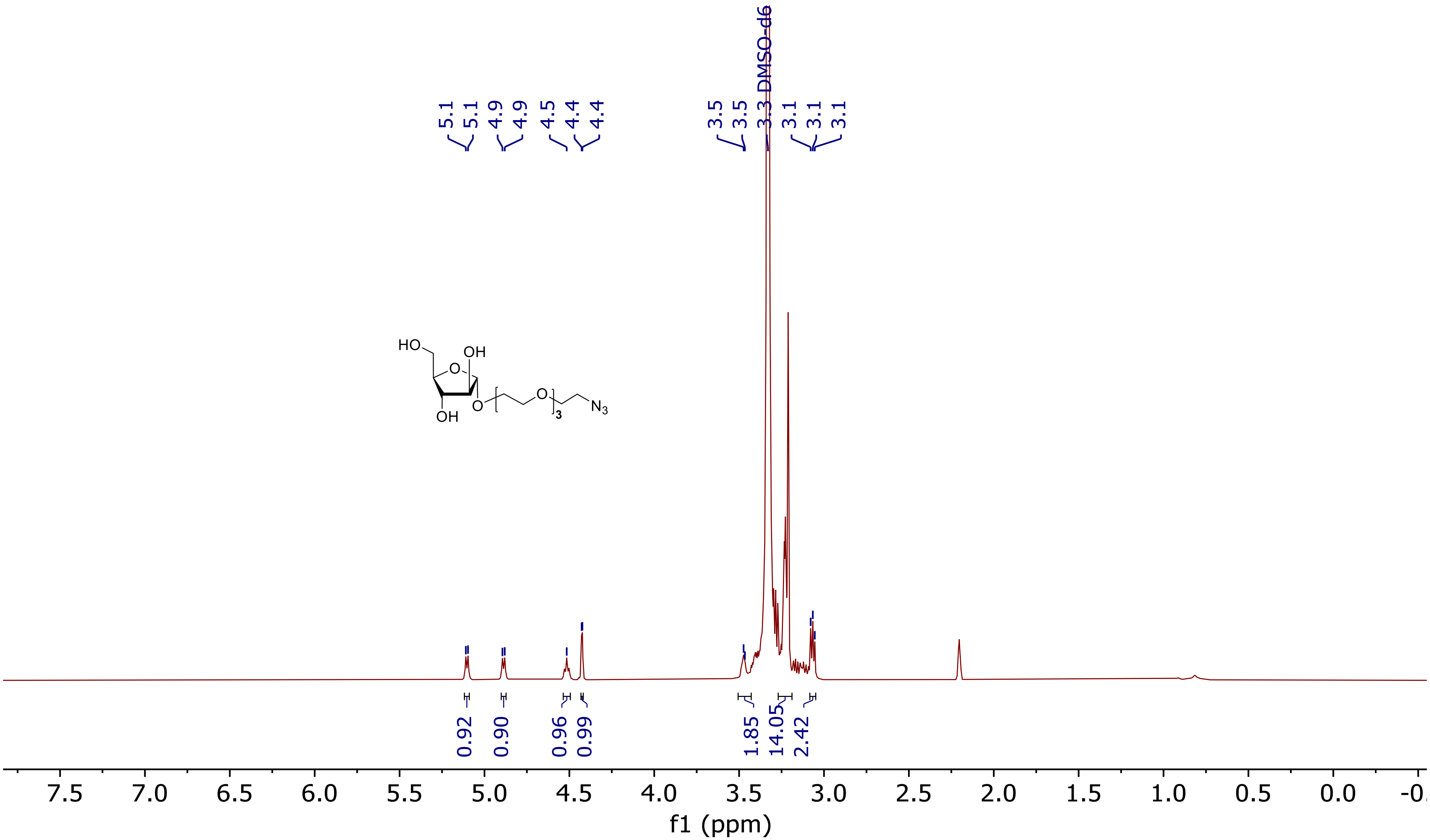

^{13}C NMR Spectrum (100.66 MHz, $CDCl_3$) of **Compound 10**

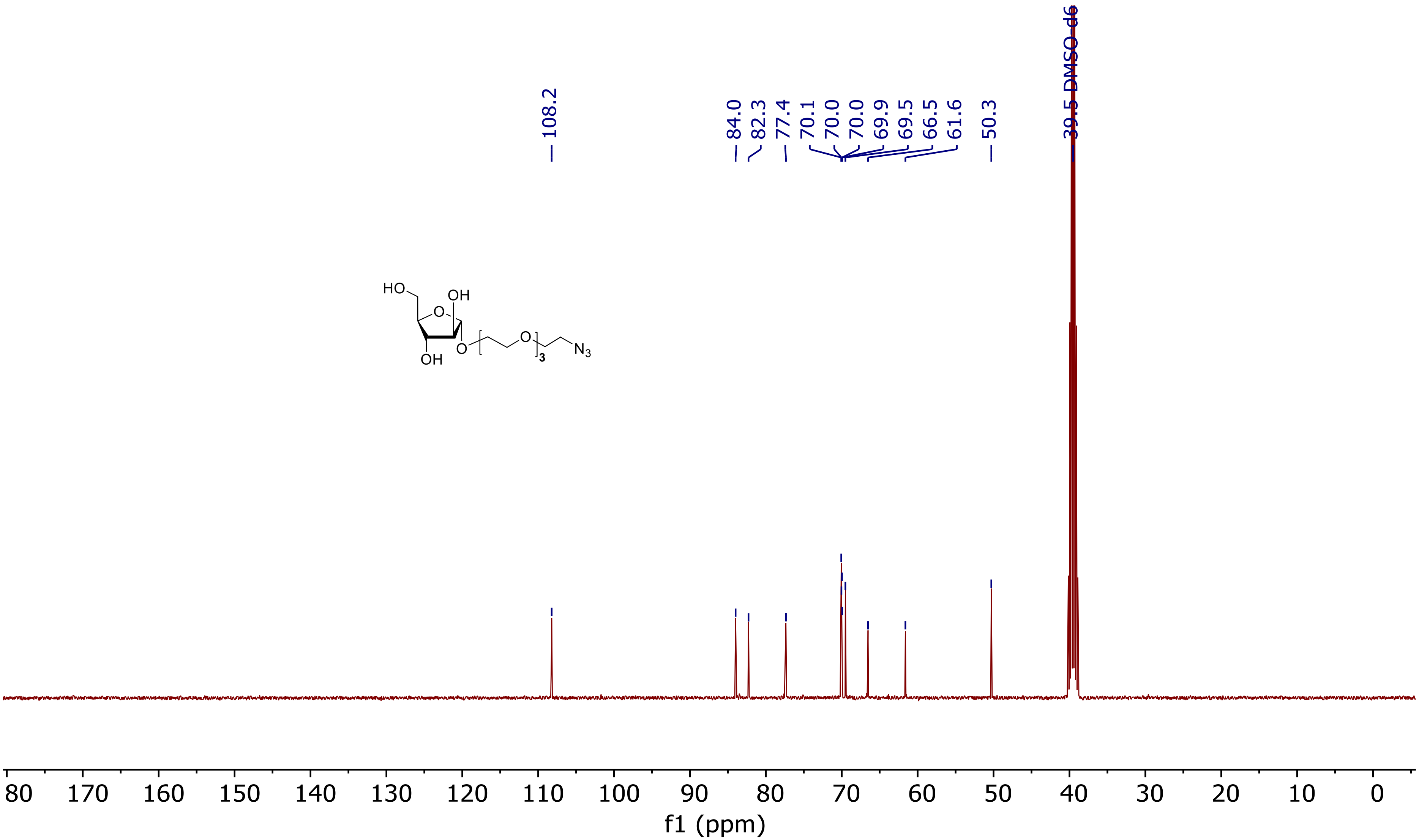

DEPT NMR Spectrum (100.66 MHz, $CDCl_3$) of **Compound 10**

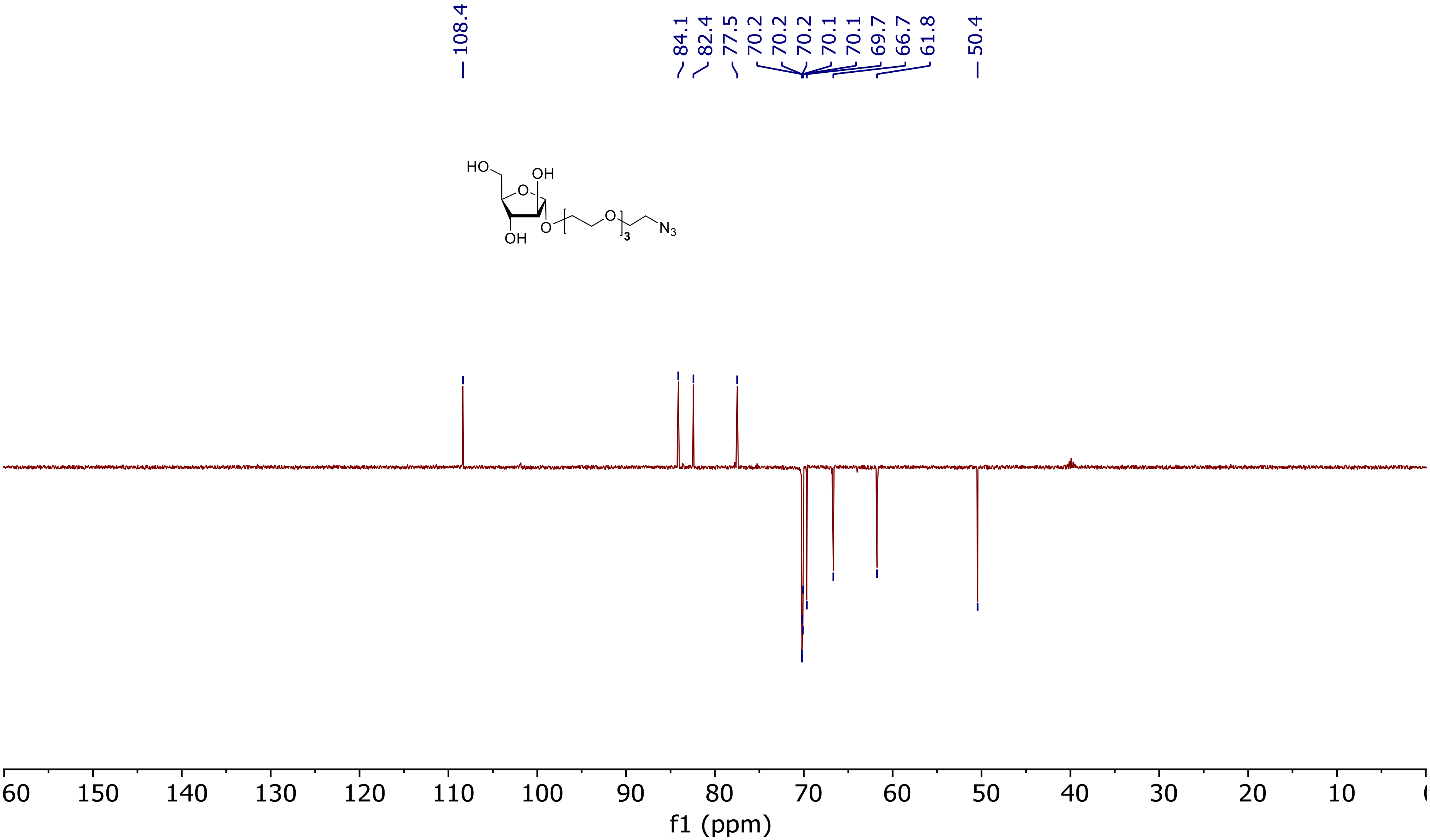

^{1}H NMR Spectrum (400.31 MHz, $CDCl_3$) of **Compound 27**

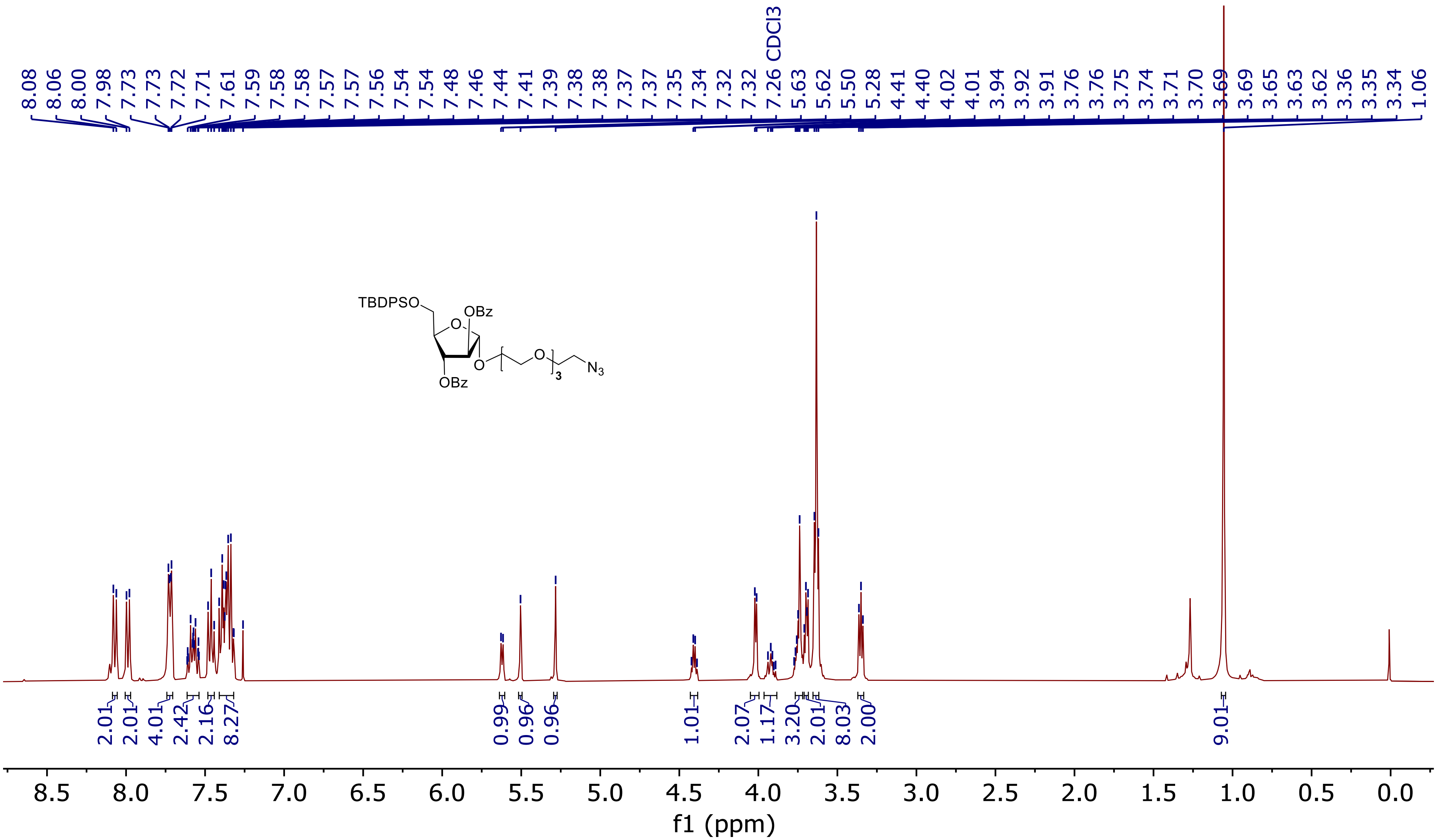

^{13}C NMR Spectrum (100.67 MHz, $CDCl_3$) of **Compound 27**

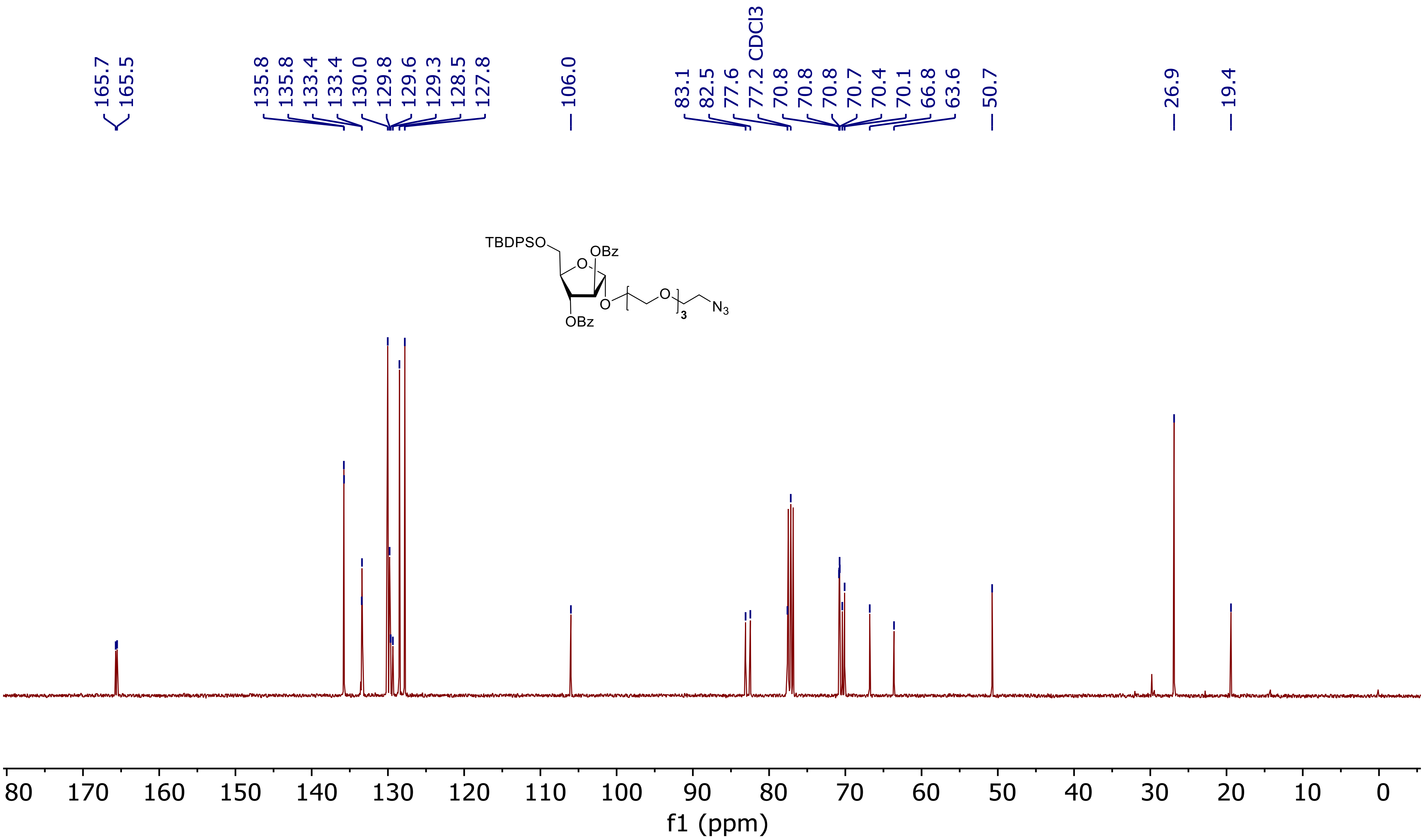

DEPT NMR Spectrum (100.67 MHz, $CDCl_3$) of **Compound 27**

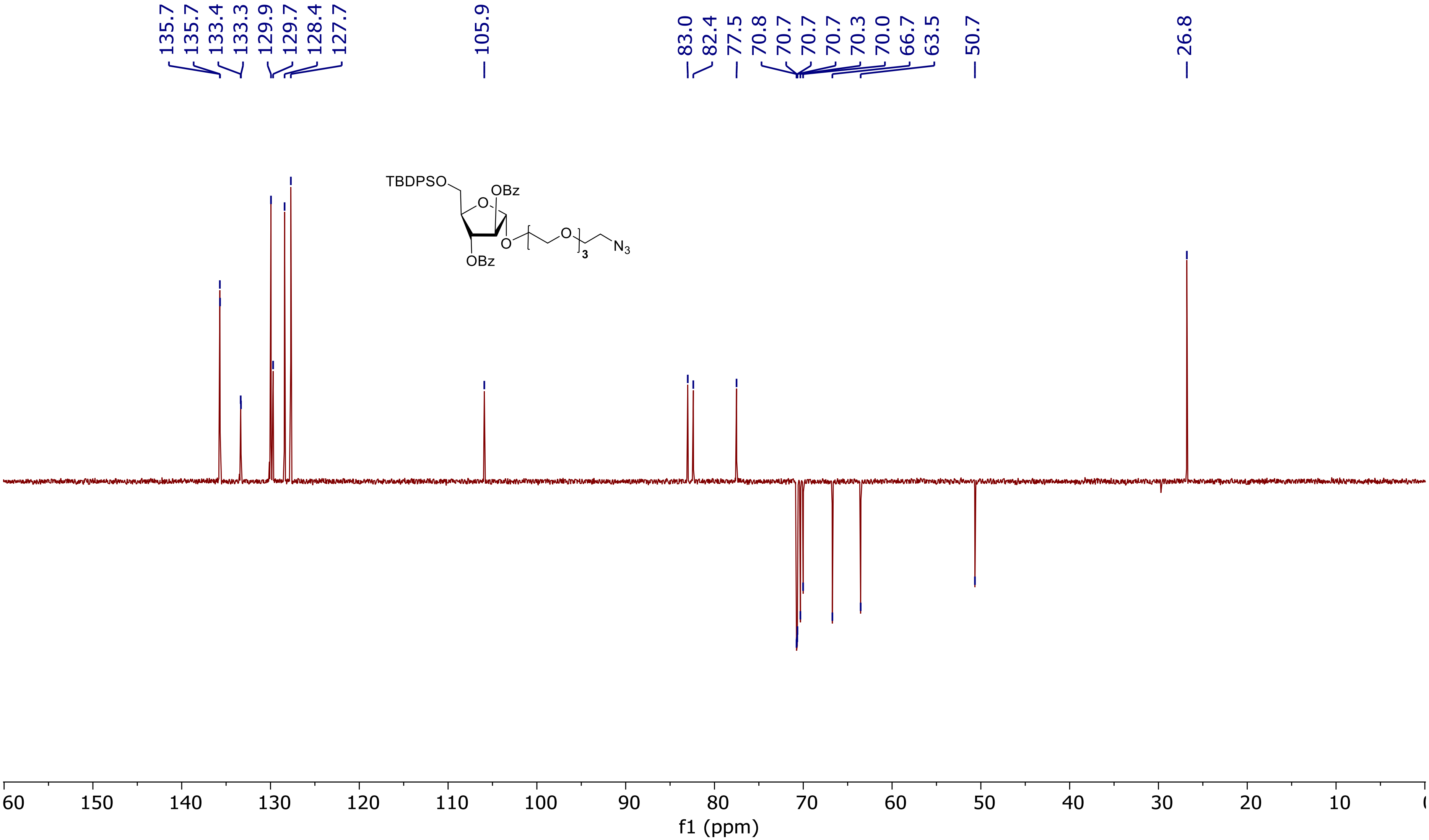

^{1}H NMR Spectrum (400.31 MHz, $CDCl_3$) of **Compound 28**

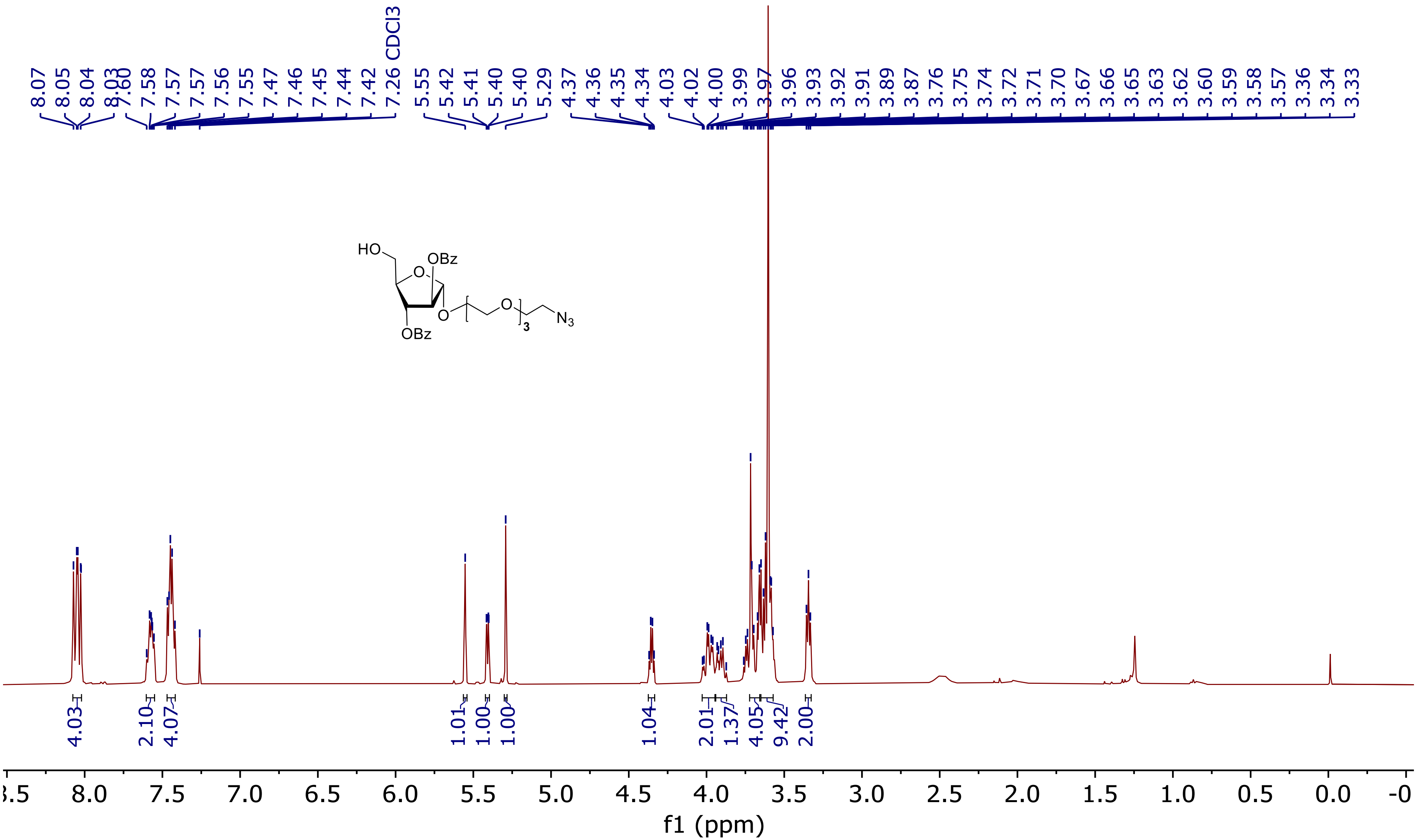

^{13}C NMR Spectrum (100.67 MHz, $CDCl_3$) of **Compound 28**

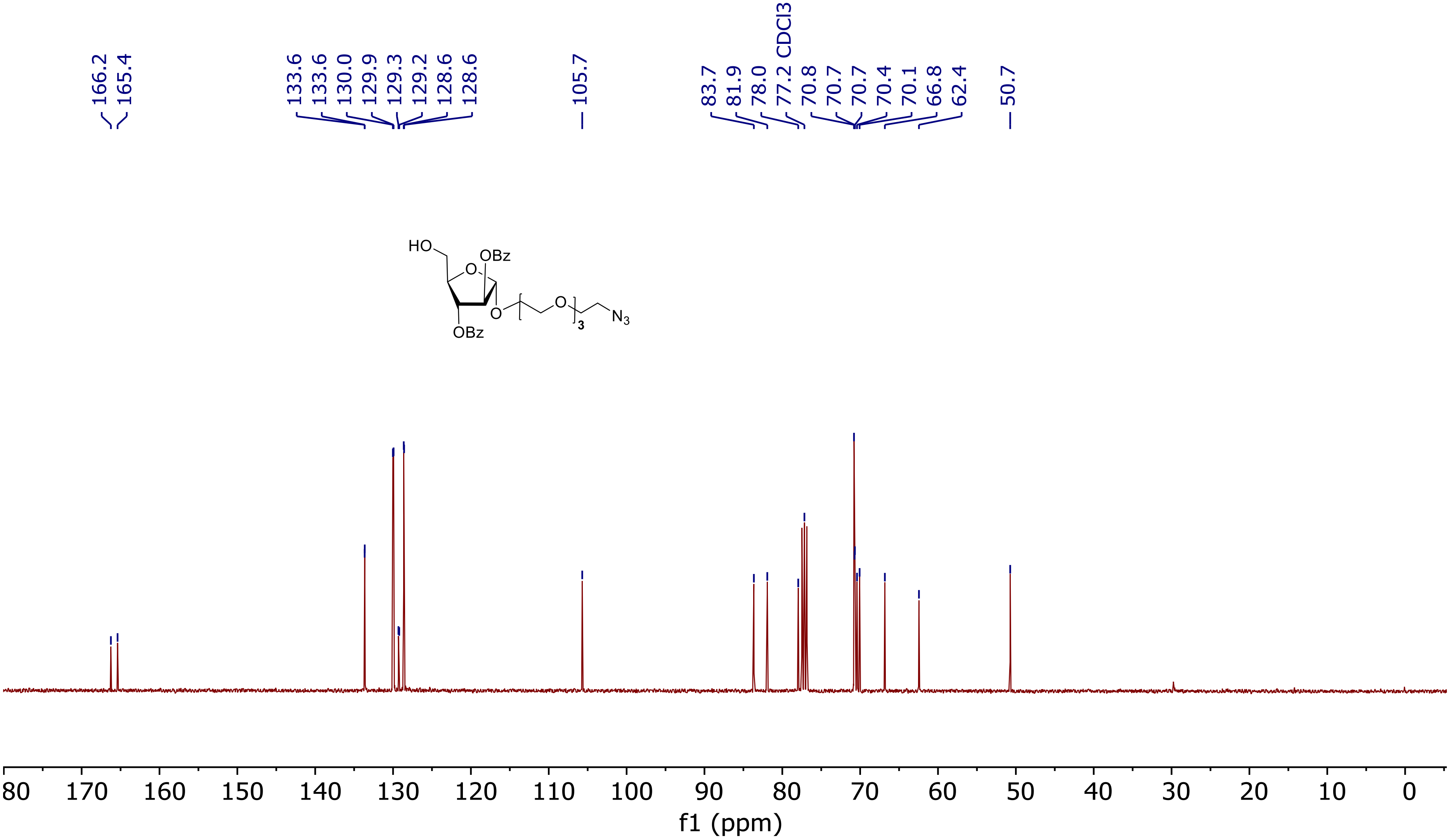

DEPT NMR Spectrum (100.67 MHz, $CDCl_3$) of **Compound 28**

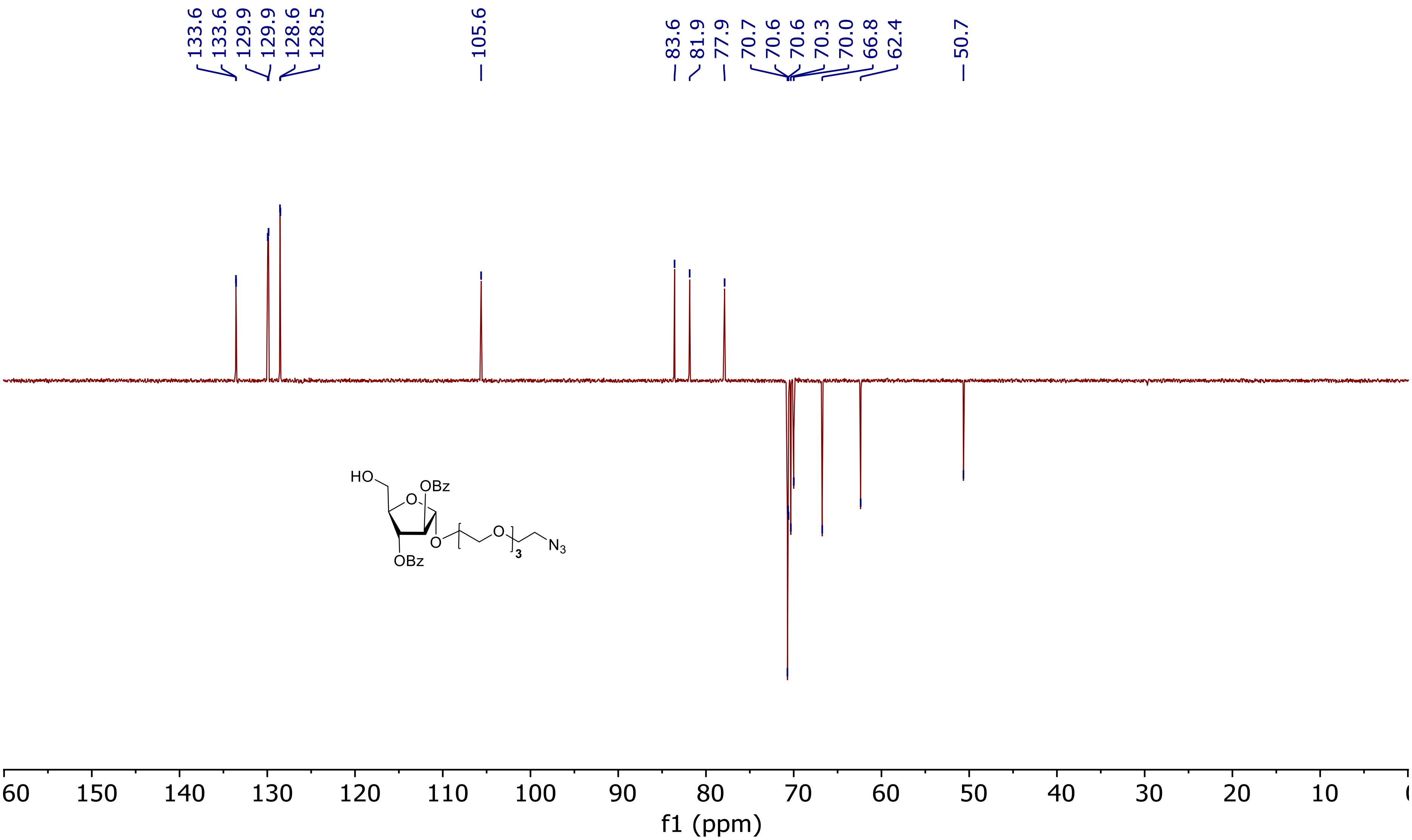

^{1}H NMR Spectrum (399.78 MHz, $CDCl_3$) of **Compound 29**

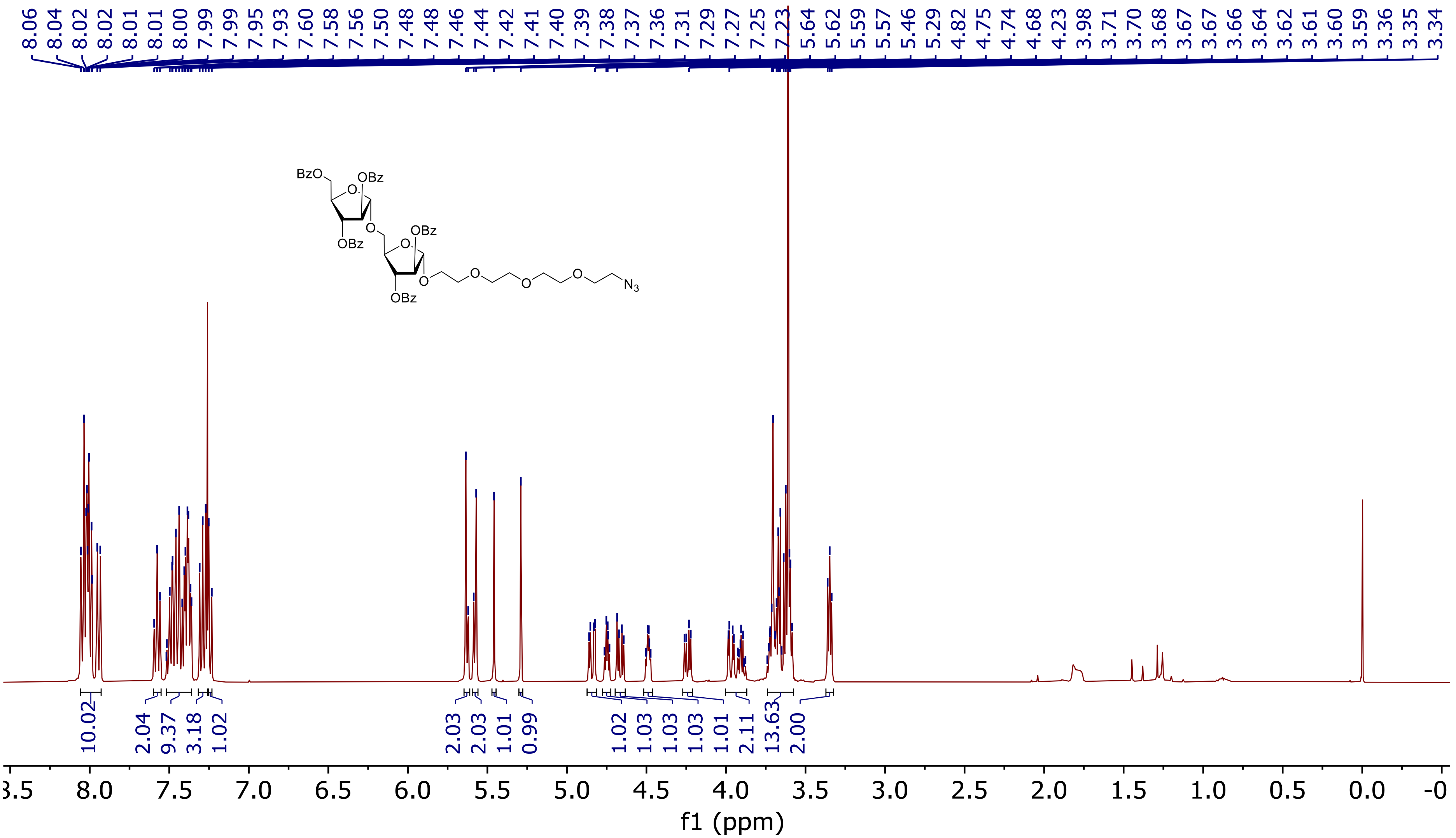

^{13}C NMR Spectrum (100.53 MHz, $CDCl_3$) of **Compound 29**

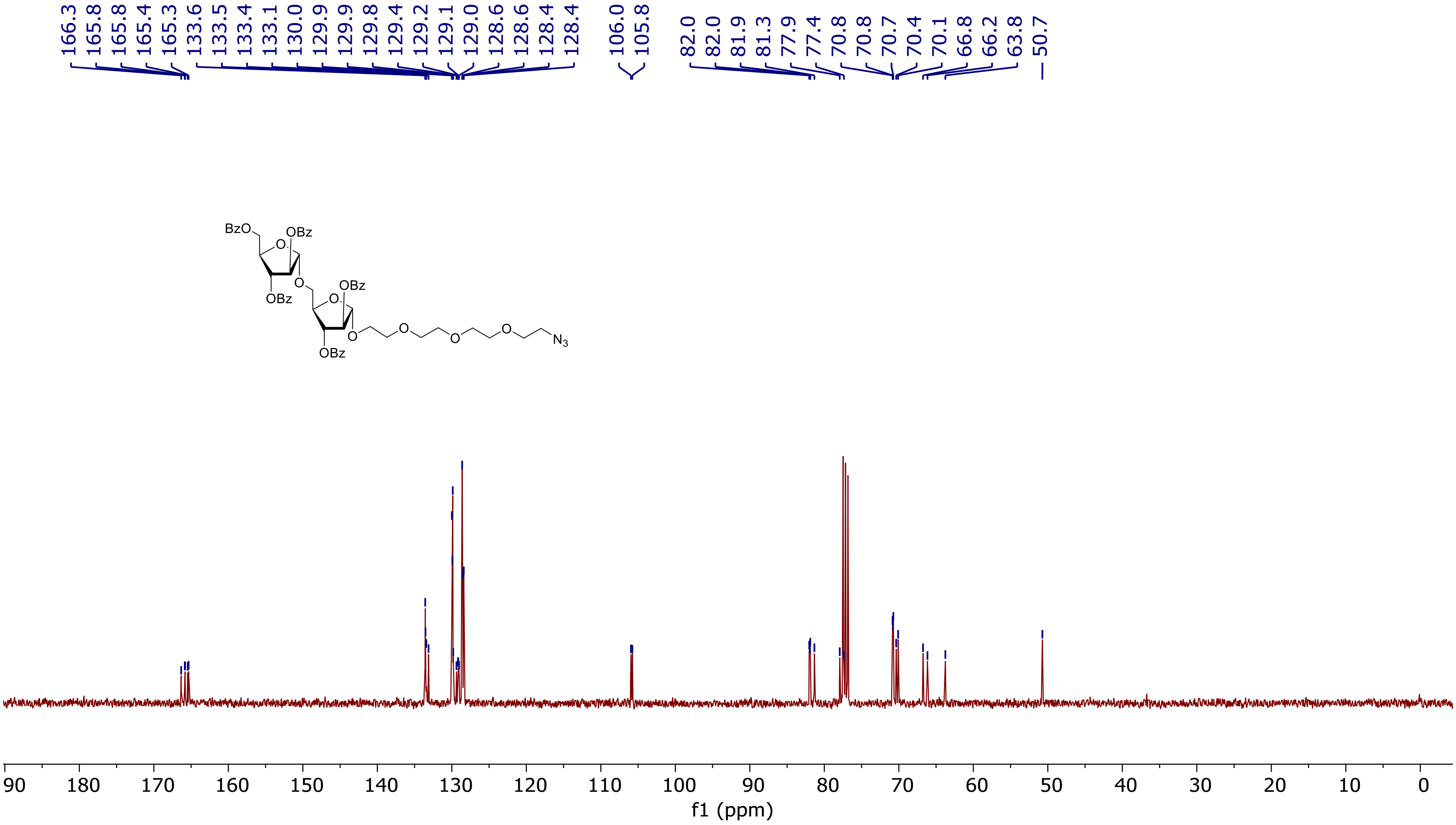

DEPT-NMR Spectrum (100.53 MHz, $CDCl_3$) of **Compound 29**

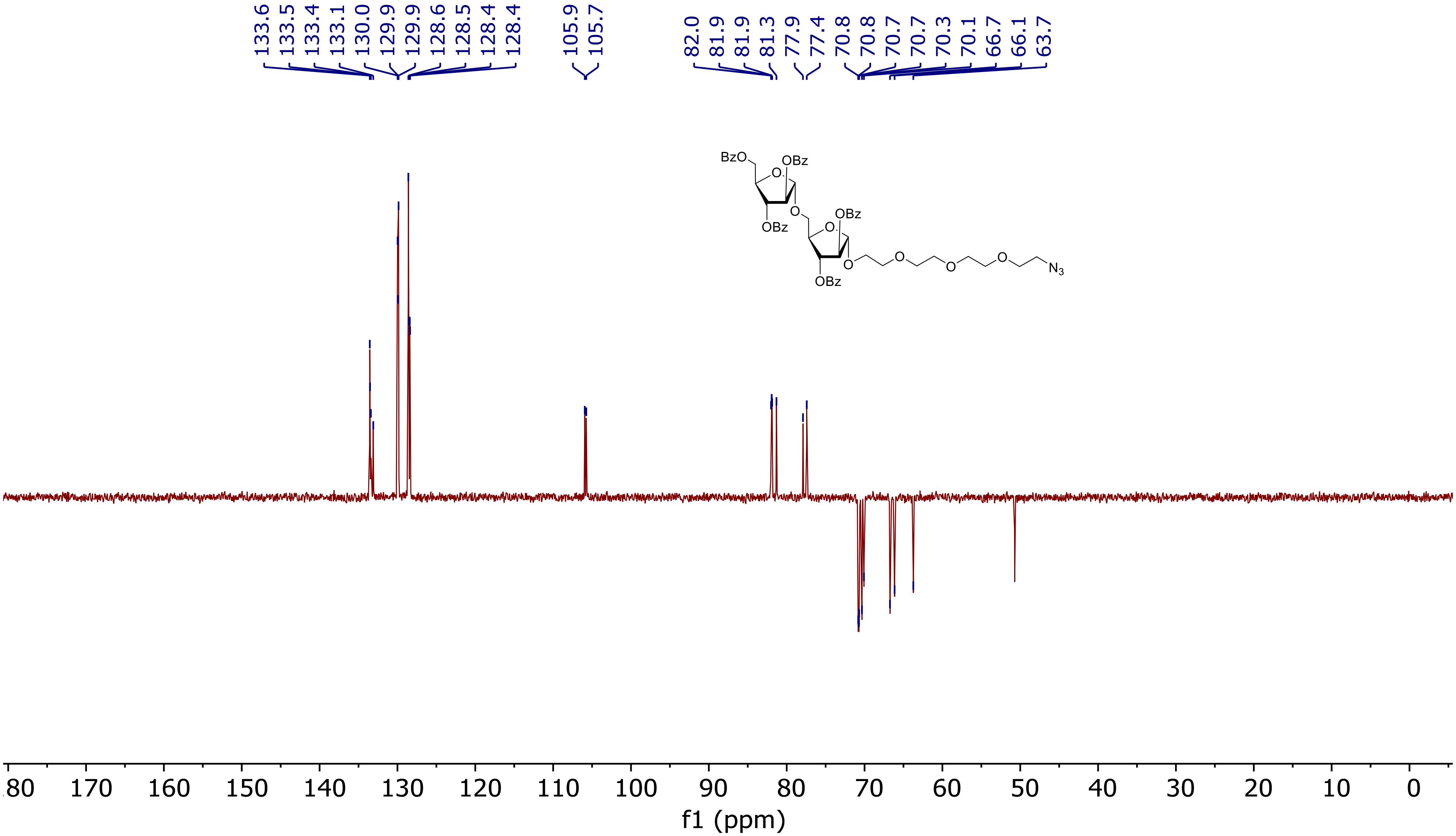

^{1}H NMR Spectrum (400.31 MHz, $CDCl_3$) of **Compound 11**

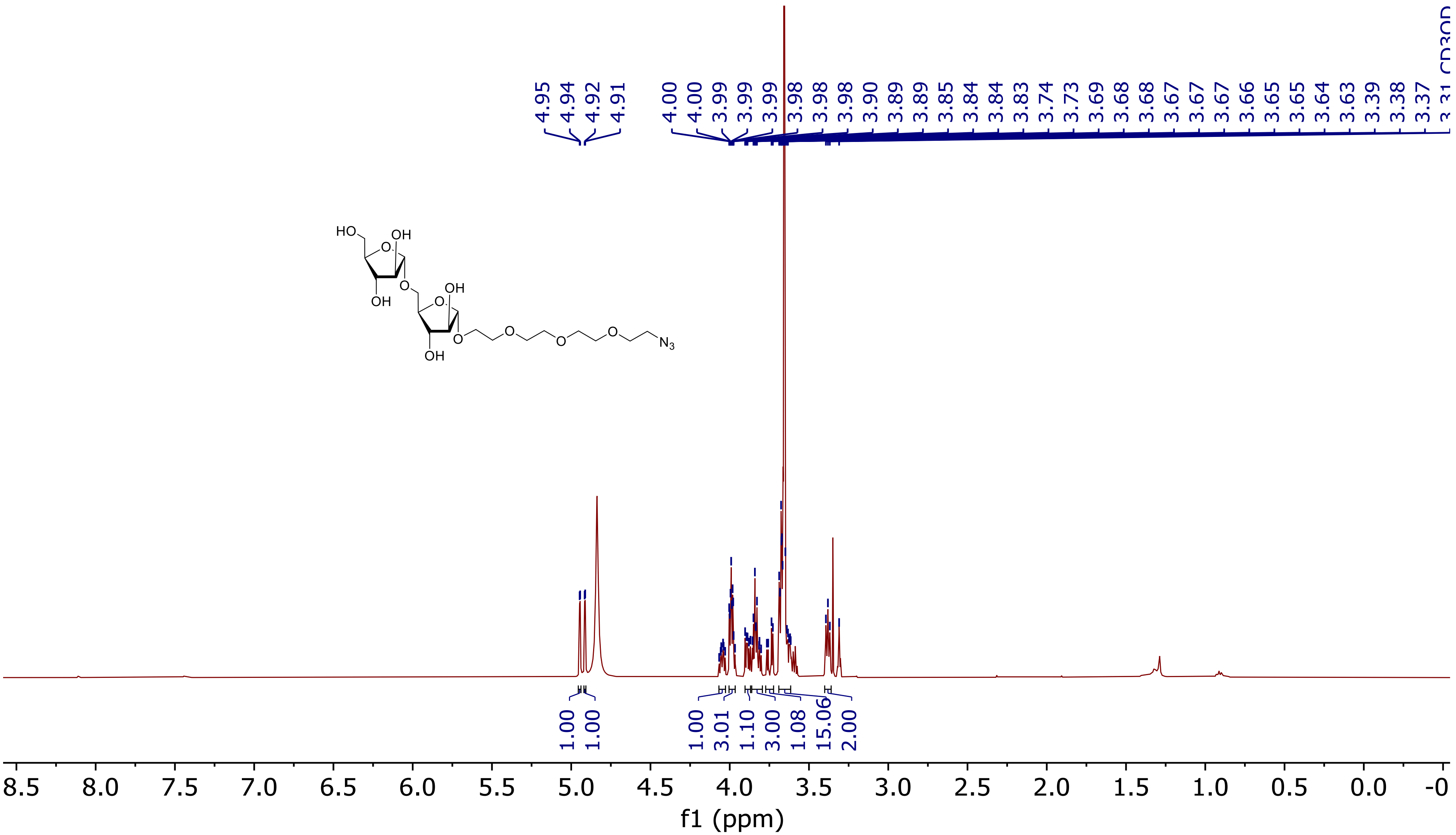

^{13}C NMR Spectrum (100.67 MHz, $CDCl_3$) of **Compound 11**

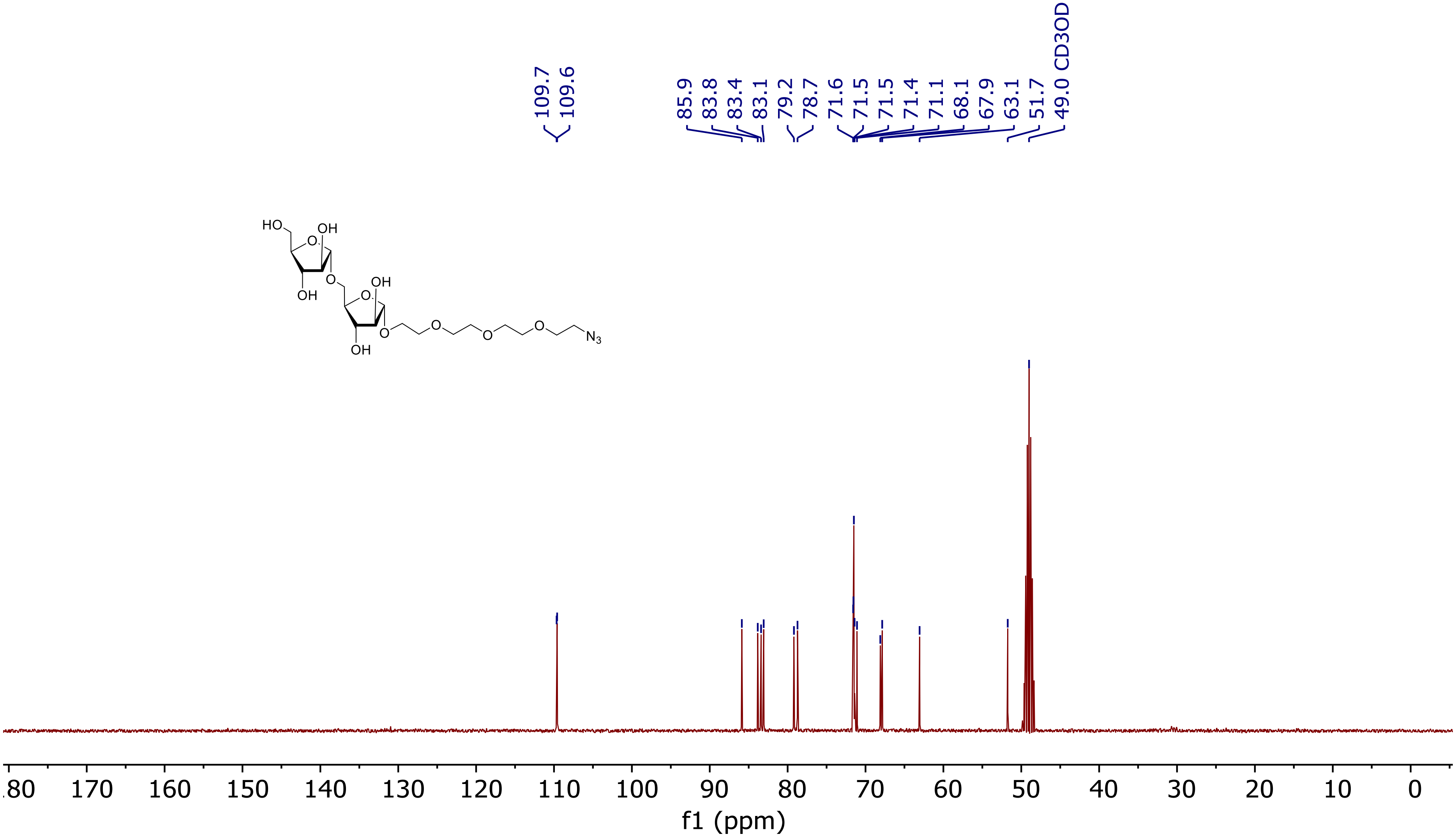

DEPT NMR Spectrum (100.67 MHz, $CDCl_3$) of **Compound 11**

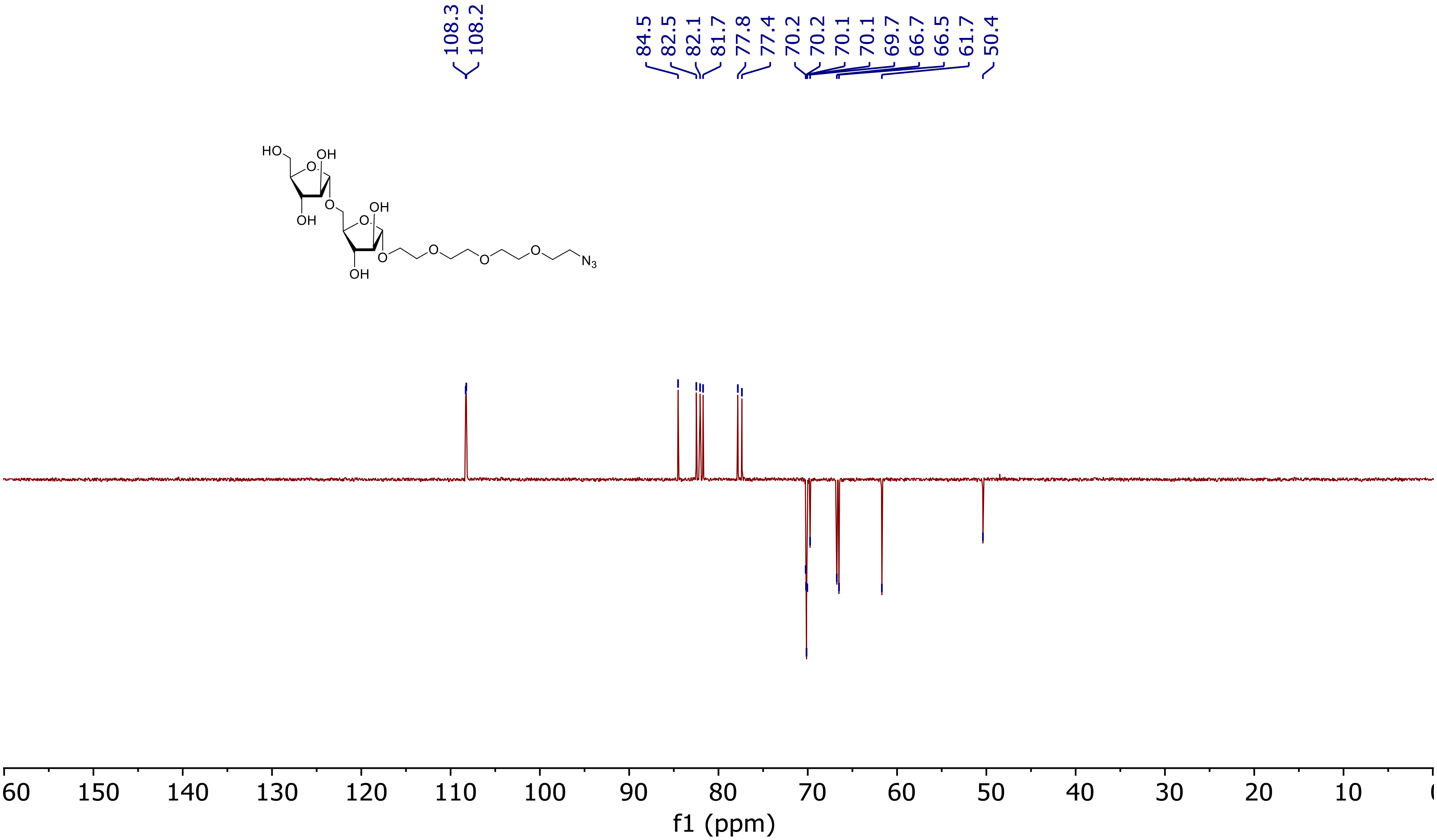

^{1}H NMR Spectrum (400.31 MHz, $CDCl_3$) of **Compound S4**

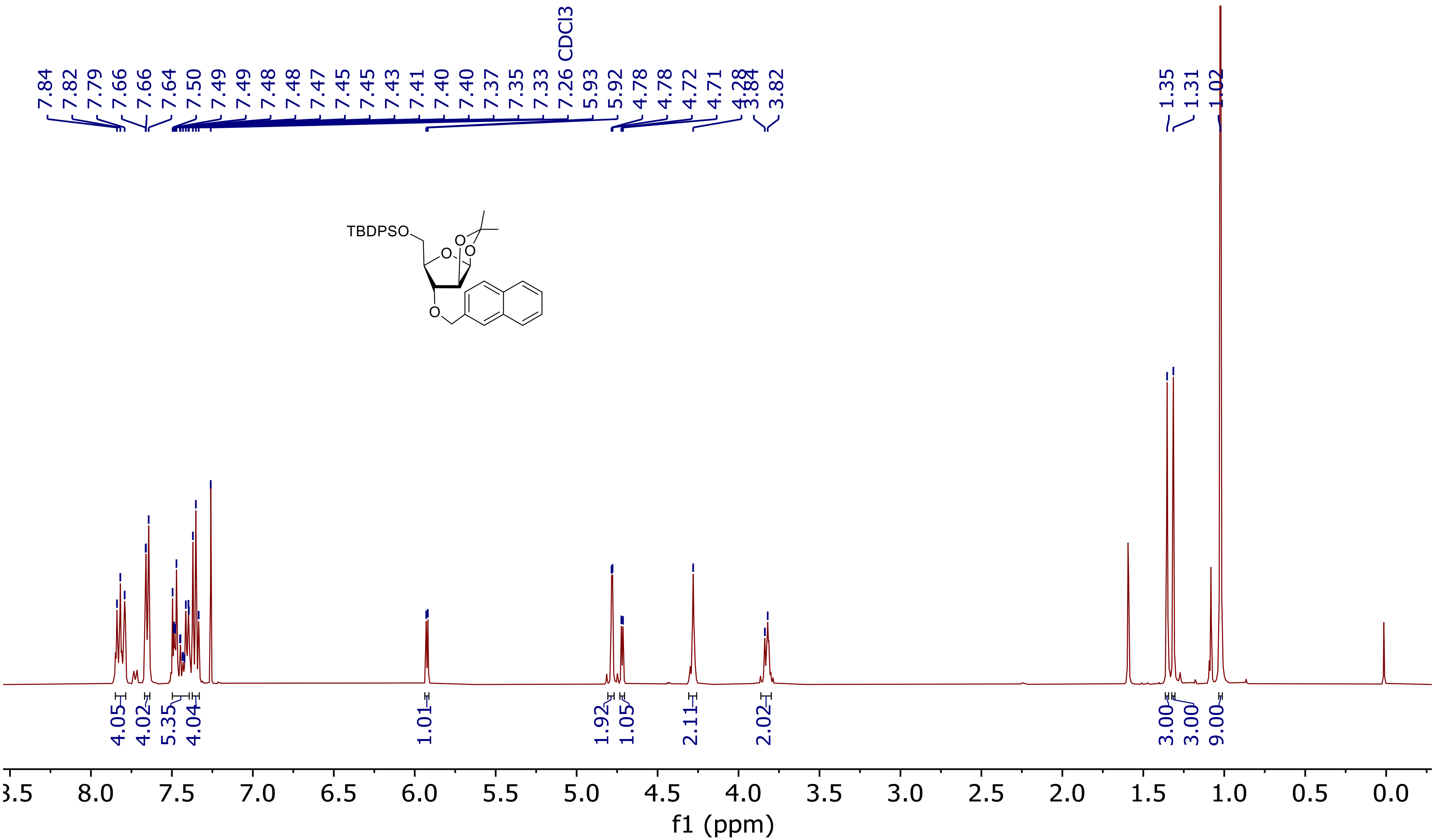

^{13}C NMR Spectrum (100.67 MHz, $CDCl_3$) of **Compound S4**

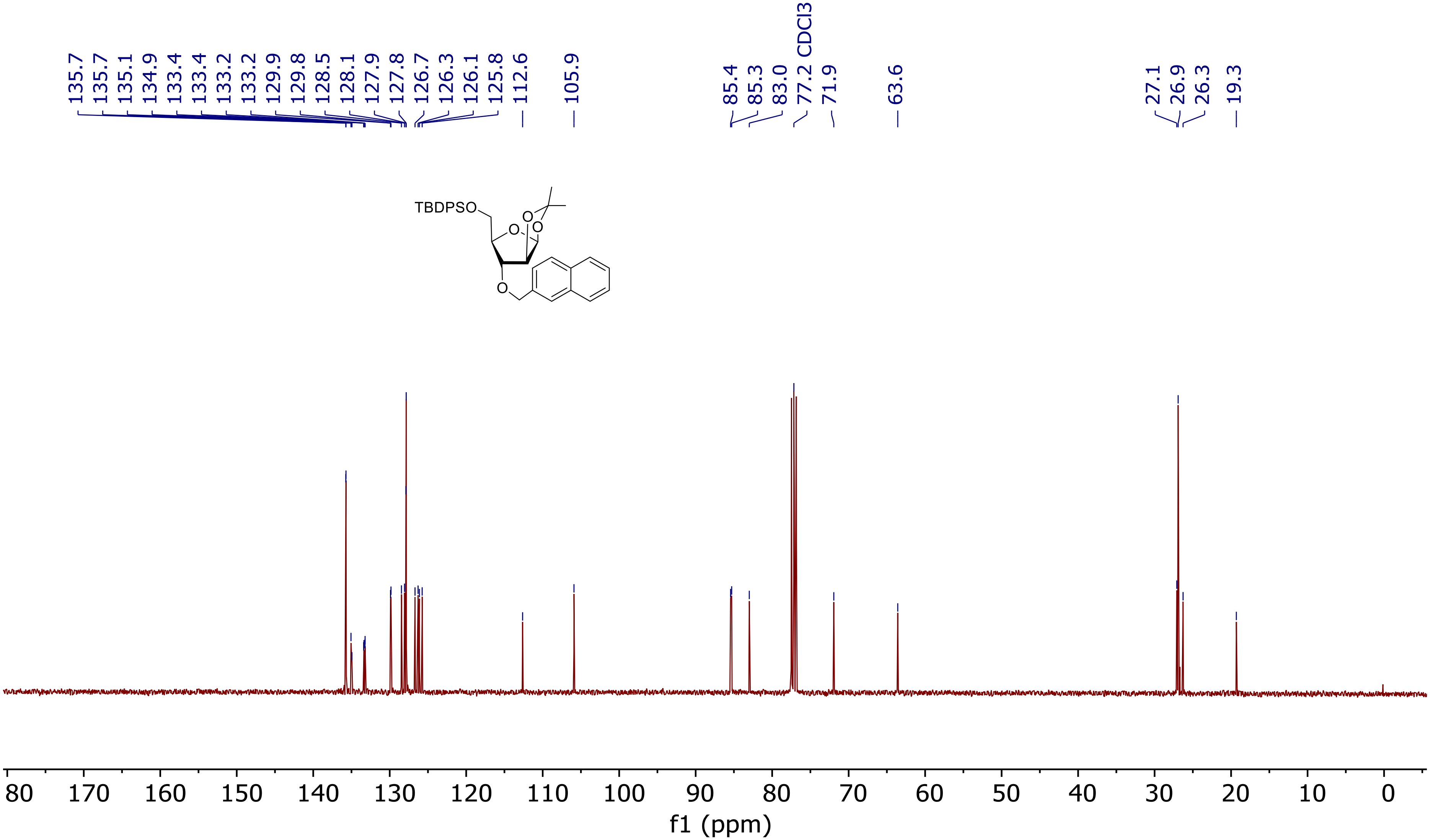

DEPT NMR Spectrum (100.67 MHz, $CDCl_3$) of **Compound S4**

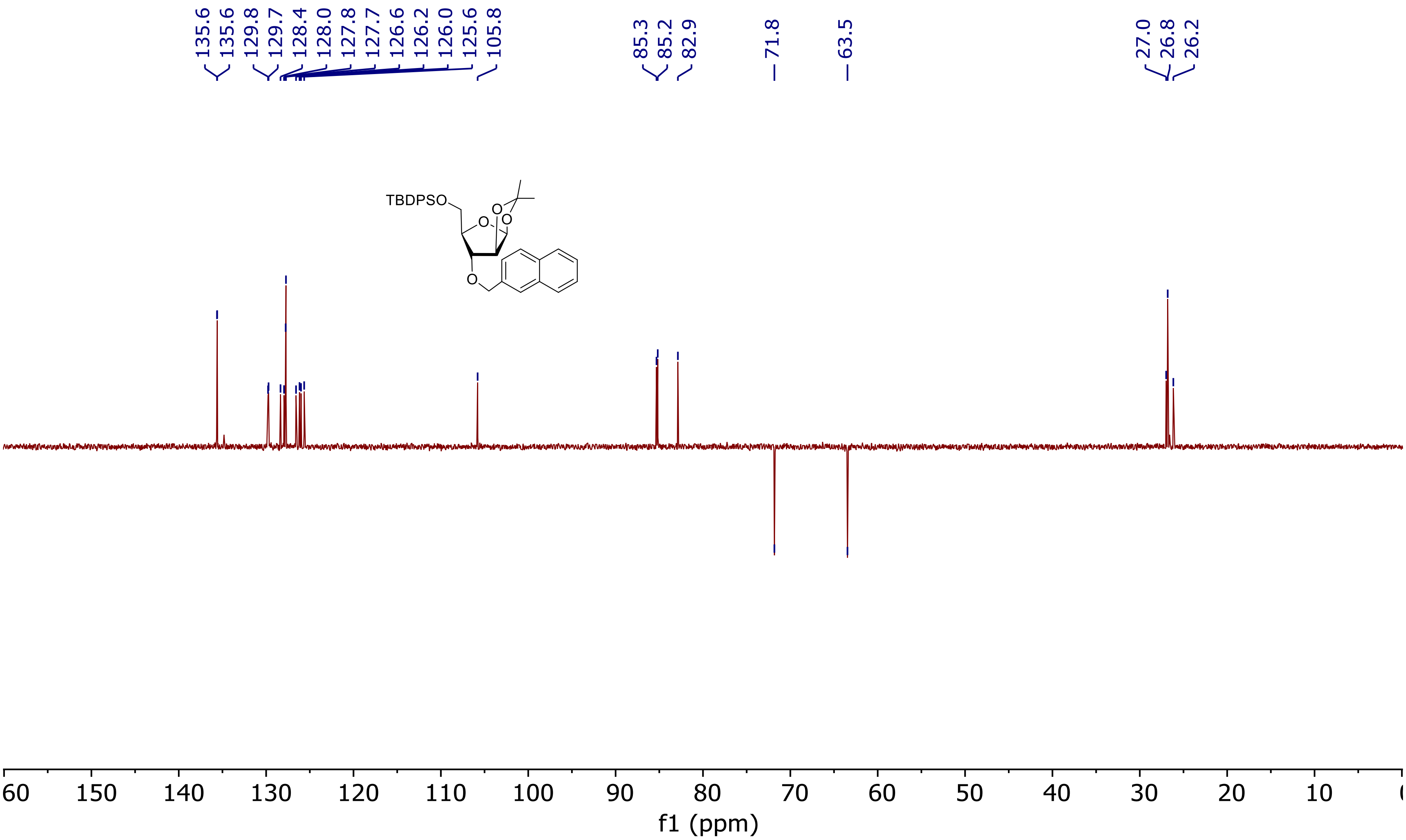

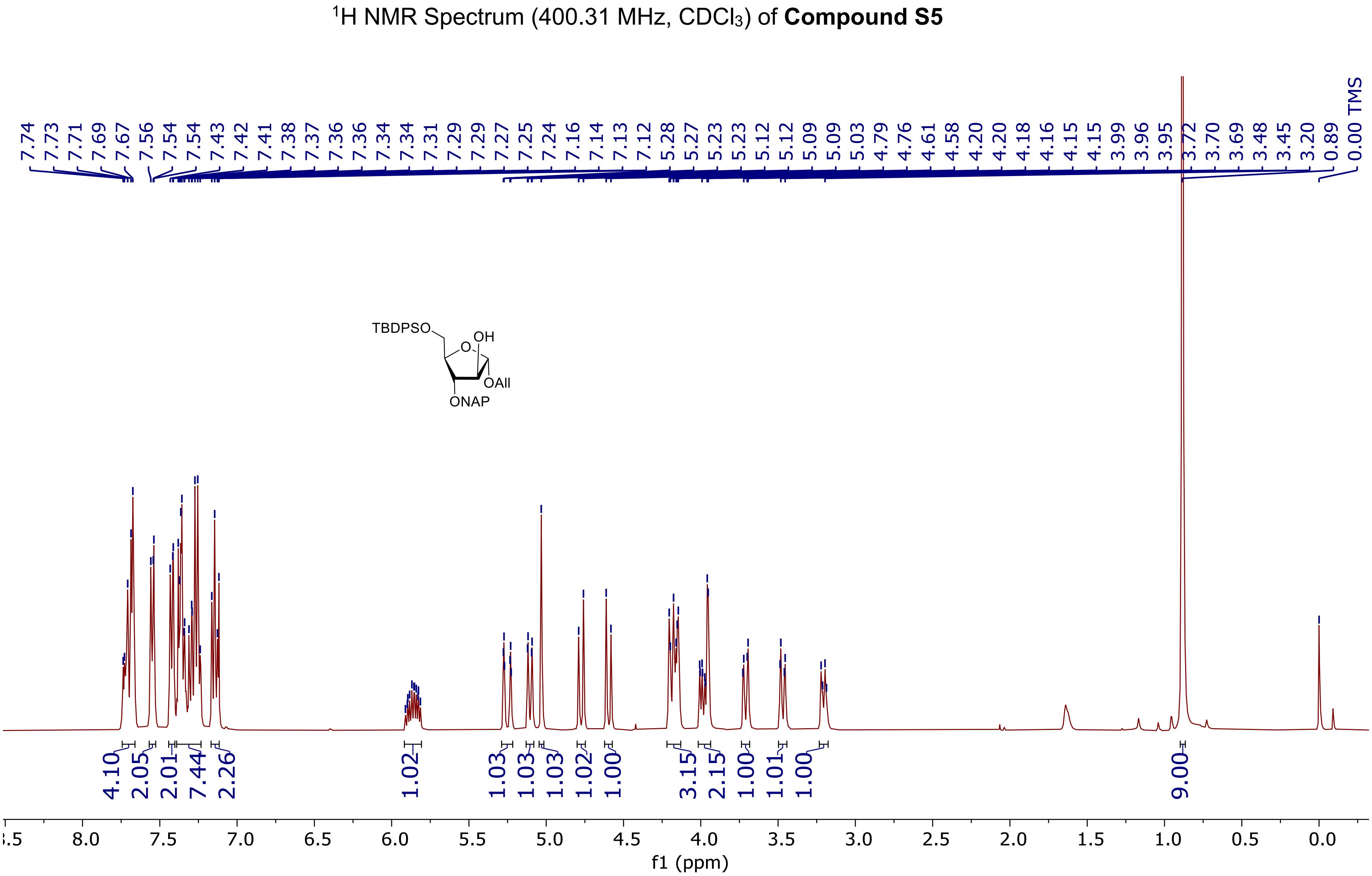

^{1}H NMR Spectrum (400.31 MHz, CDCl3) of **Compound S5**
TBDPSO
OH
O
OAll
ONAP
7.74 7.73 7.71 7.69 7.67 7.56 7.54 7.54 7.43 7.42 7.41 7.38 7.37 7.36 7.36 7.34 7.34 7.31 7.29 7.29 7.27 7.25 7.24 7.16 7.14 7.13 7.12 5.28 5.27 5.23 5.23 5.12 5.12 5.09 5.09 5.03 4.79 4.76 4.61 4.58 4.20 4.20 4.18 4.16 4.15 4.15 3.99 3.96 3.95 3.72 3.70 3.69 3.48 3.45 3.20 0.89 0.00 TMS
4.10 2.05 2.01 7.44 2.26 1.02 1.03 1.03 1.03 1.02 1.00 3.15 2.15 1.00 1.01 1.00 9.00
8.0 7.5 7.0 6.5 6.0 5.5 5.0 4.5 4.0 3.5 3.0 2.5 2.0 1.5 1.0 0.5 0.0
f1 (ppm)

^{13}C NMR Spectrum (100.67 MHz, $CDCl_3$) of **Compound S5**

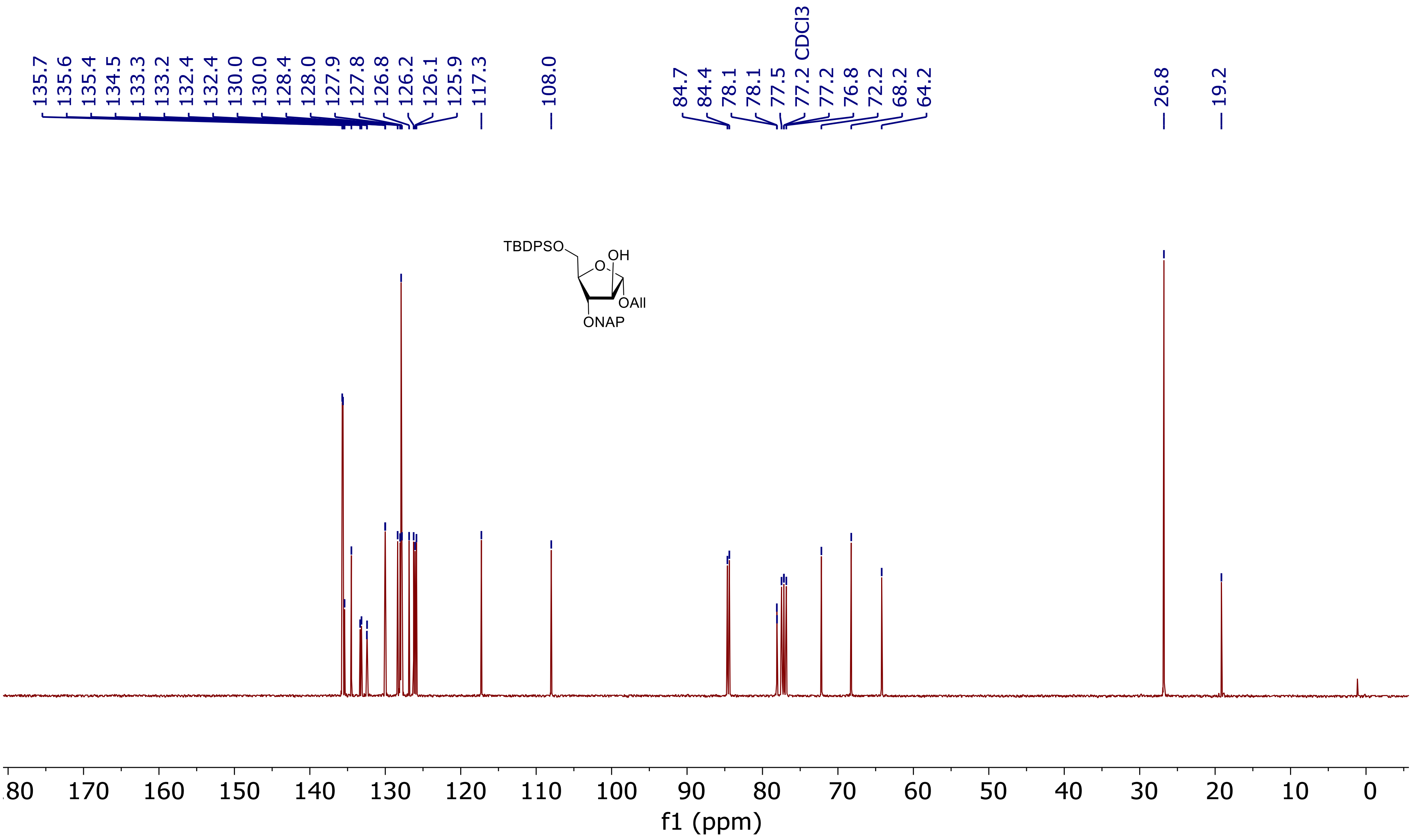

DEPT NMR Spectrum (100.67 MHz, $CDCl_3$) of **Compound S5**

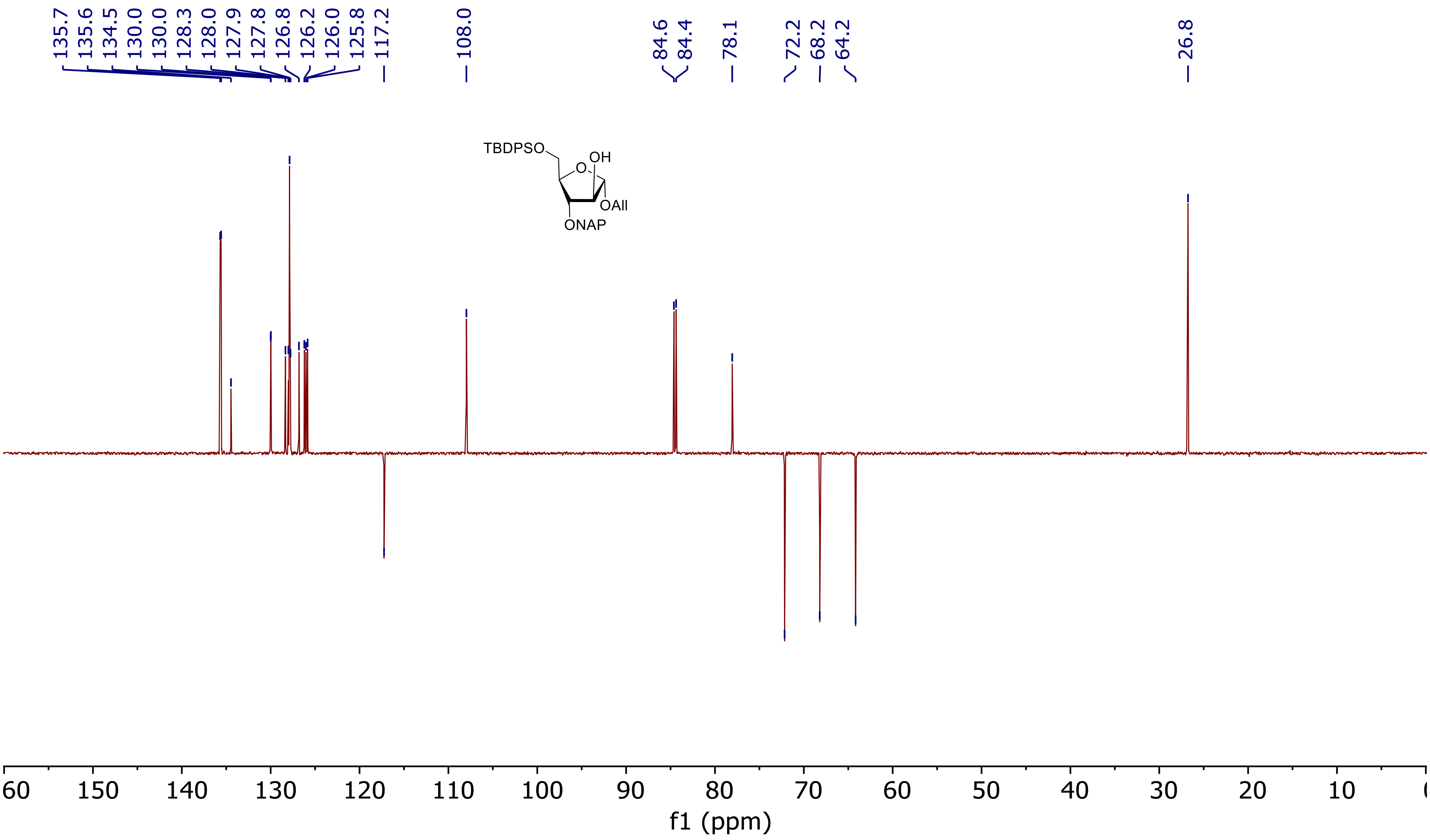

^{1}H NMR Spectrum (400.31 MHz, $CDCl_3$) of **Compound S6**

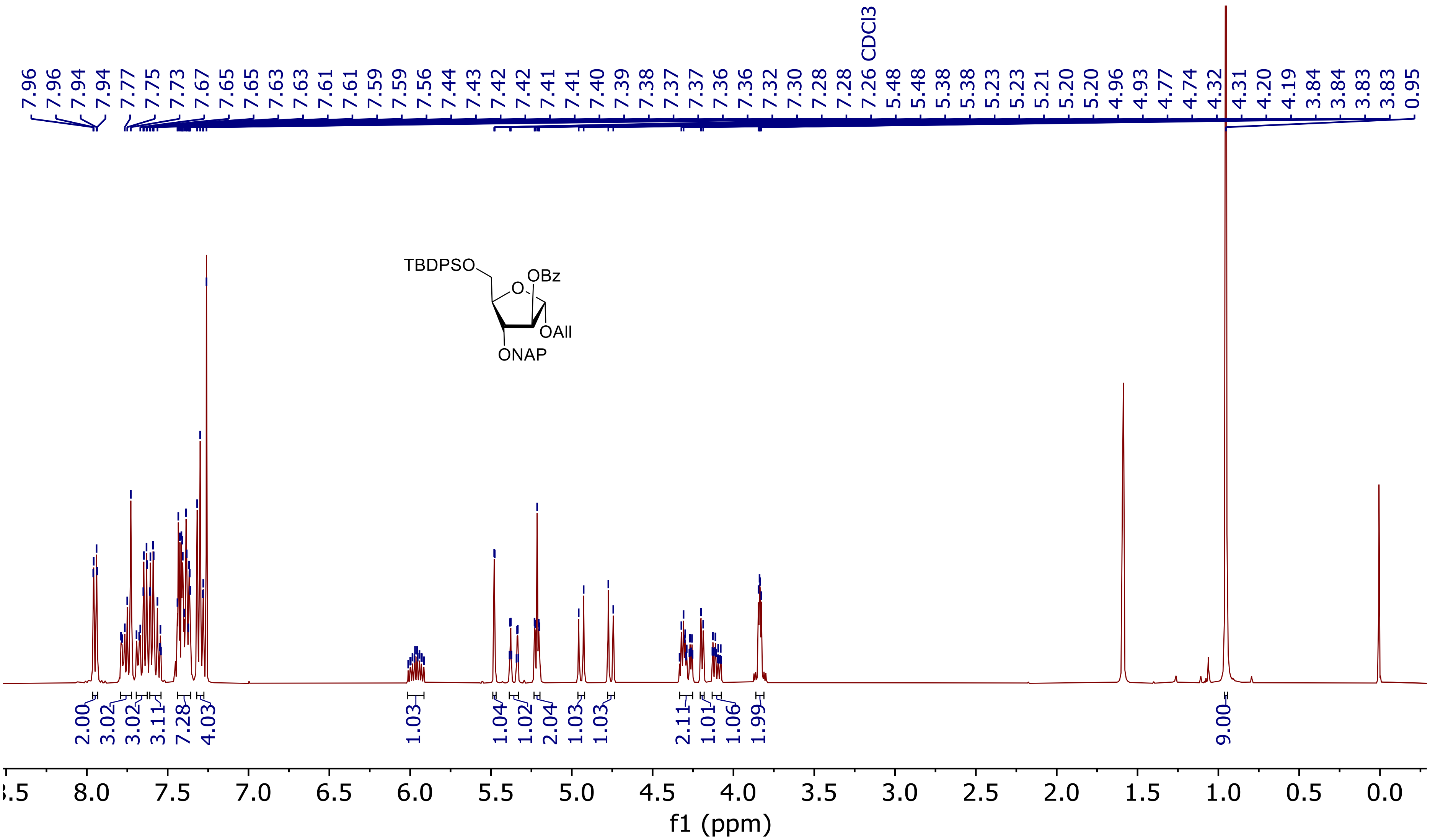

^{13}C NMR Spectrum (100.67 MHz, $CDCl_3$) of **Compound S6**

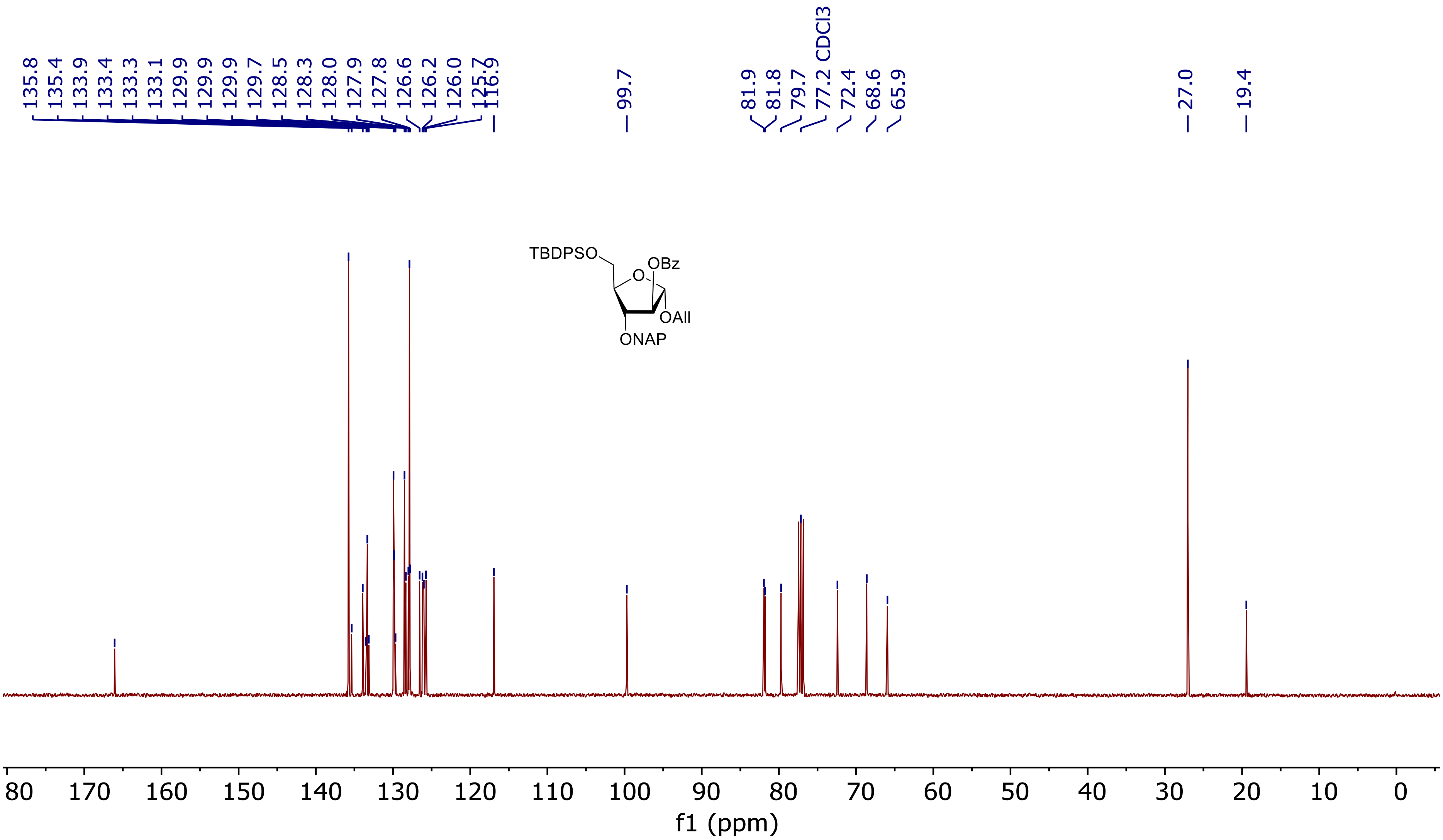

DEPT NMR Spectrum (100.67 MHz, $CDCl_3$) of **Compound S6**

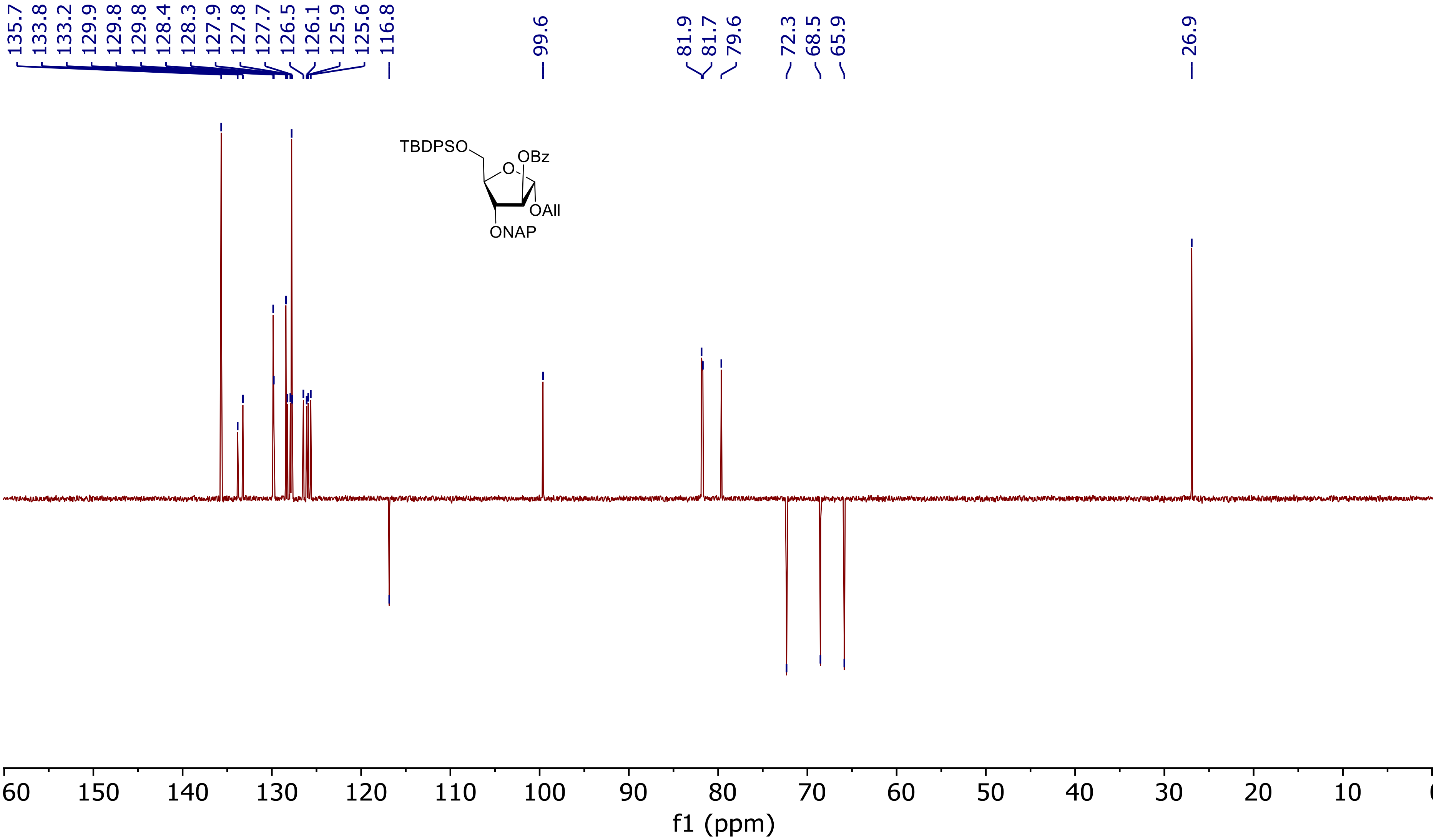

^{1}H NMR Spectrum (400.31 MHz, $CDCl_3$) of **Compound 30**

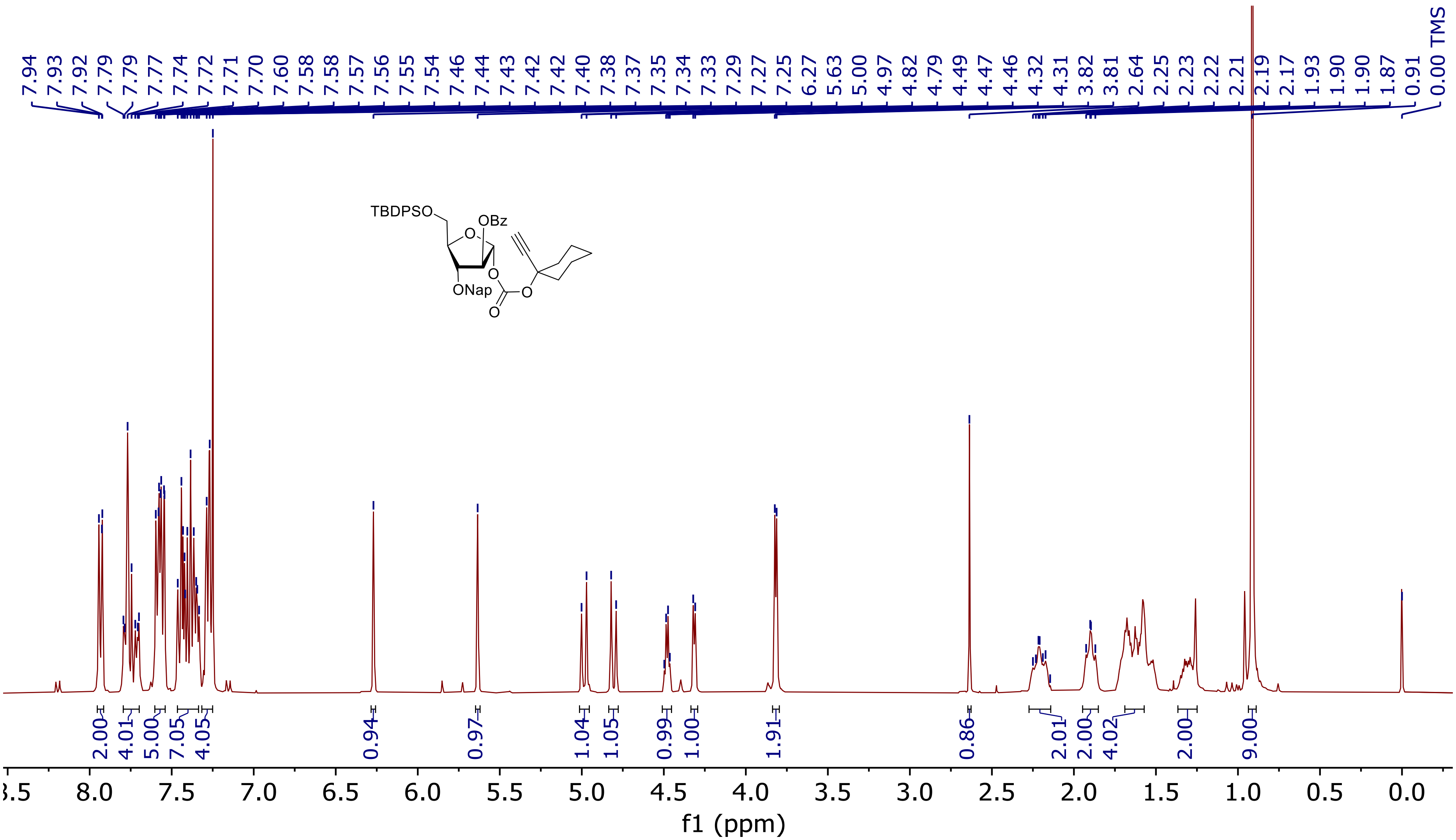

^{13}C NMR Spectrum (100.67 MHz, $CDCl_3$) of **Compound 30**

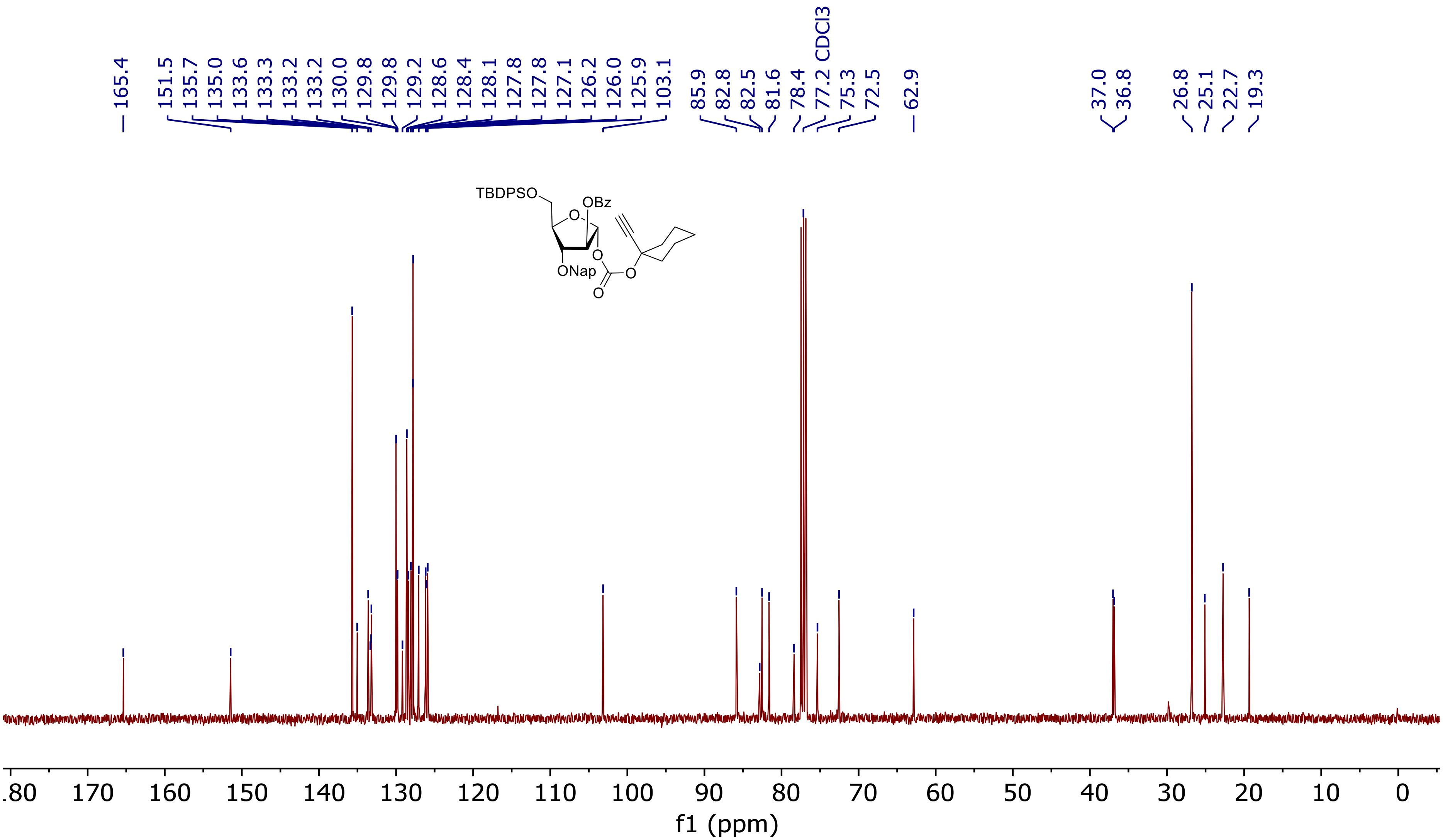

DEPT NMR Spectrum (100.67 MHz, $CDCl_3$) of **Compound 30**

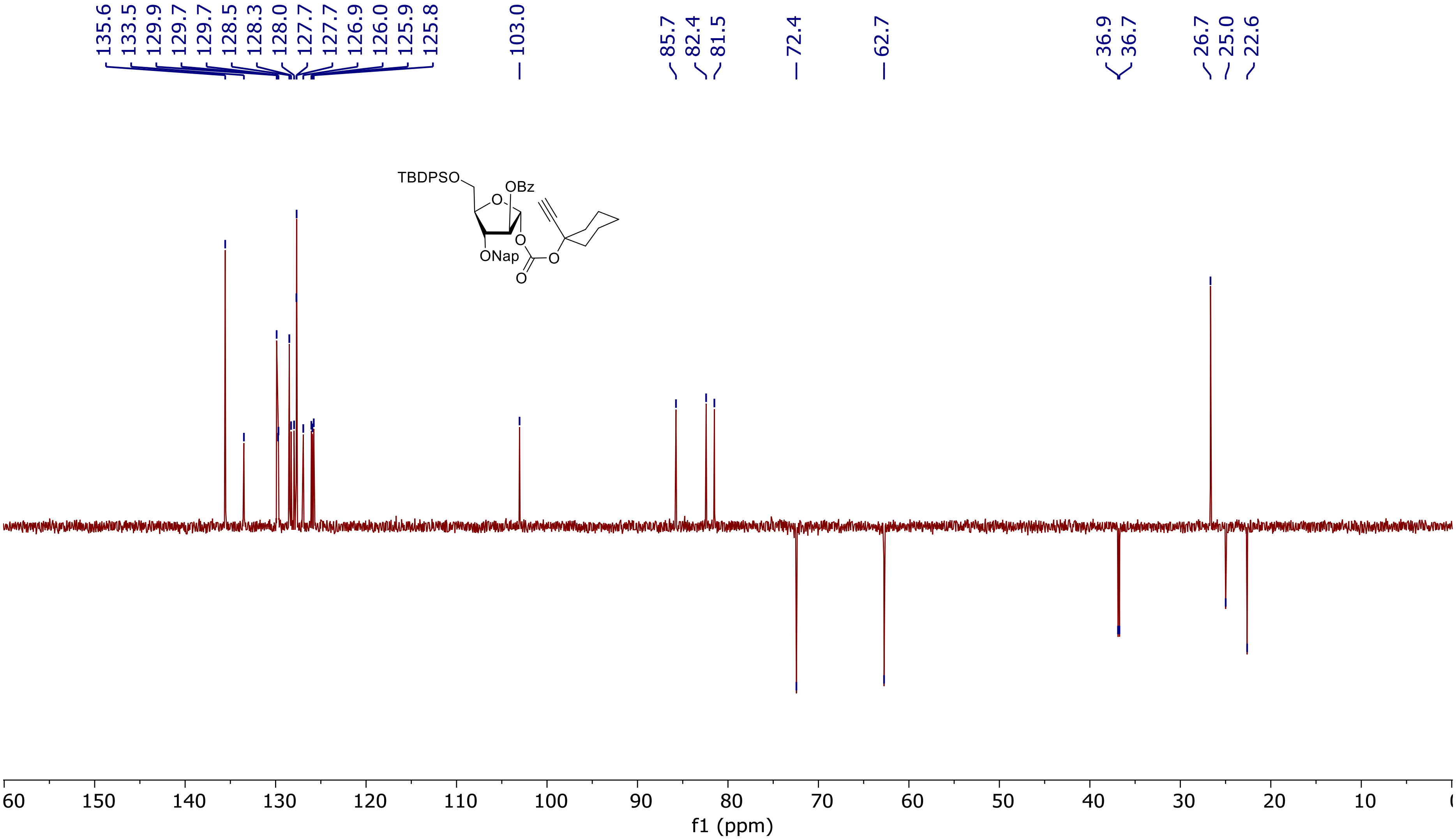

^{1}H NMR Spectrum (400.31 MHz, $CDCl_3$) of **Compound 31**

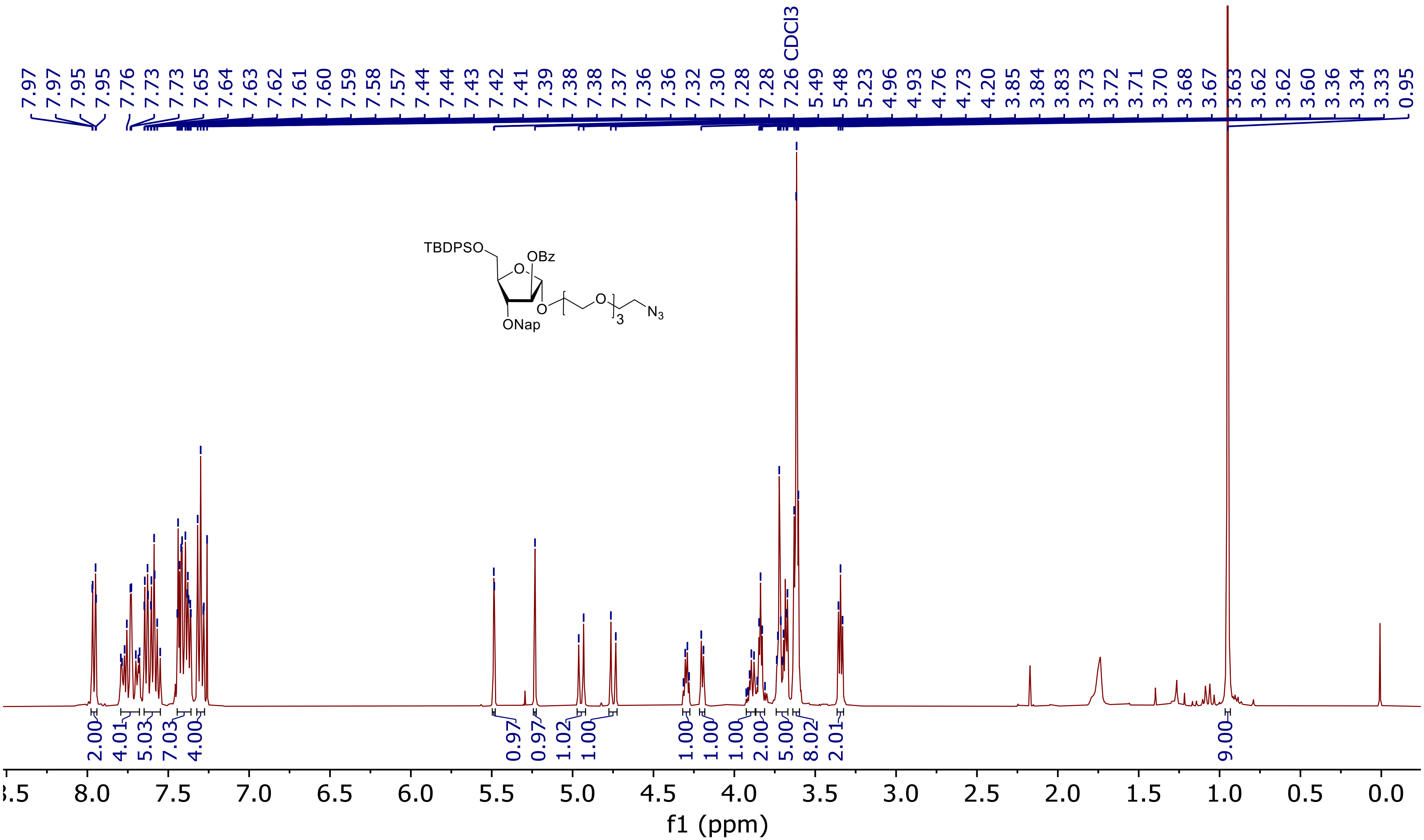

^{13}C NMR Spectrum (100.67 MHz, $CDCl_3$) of **Compound 31**

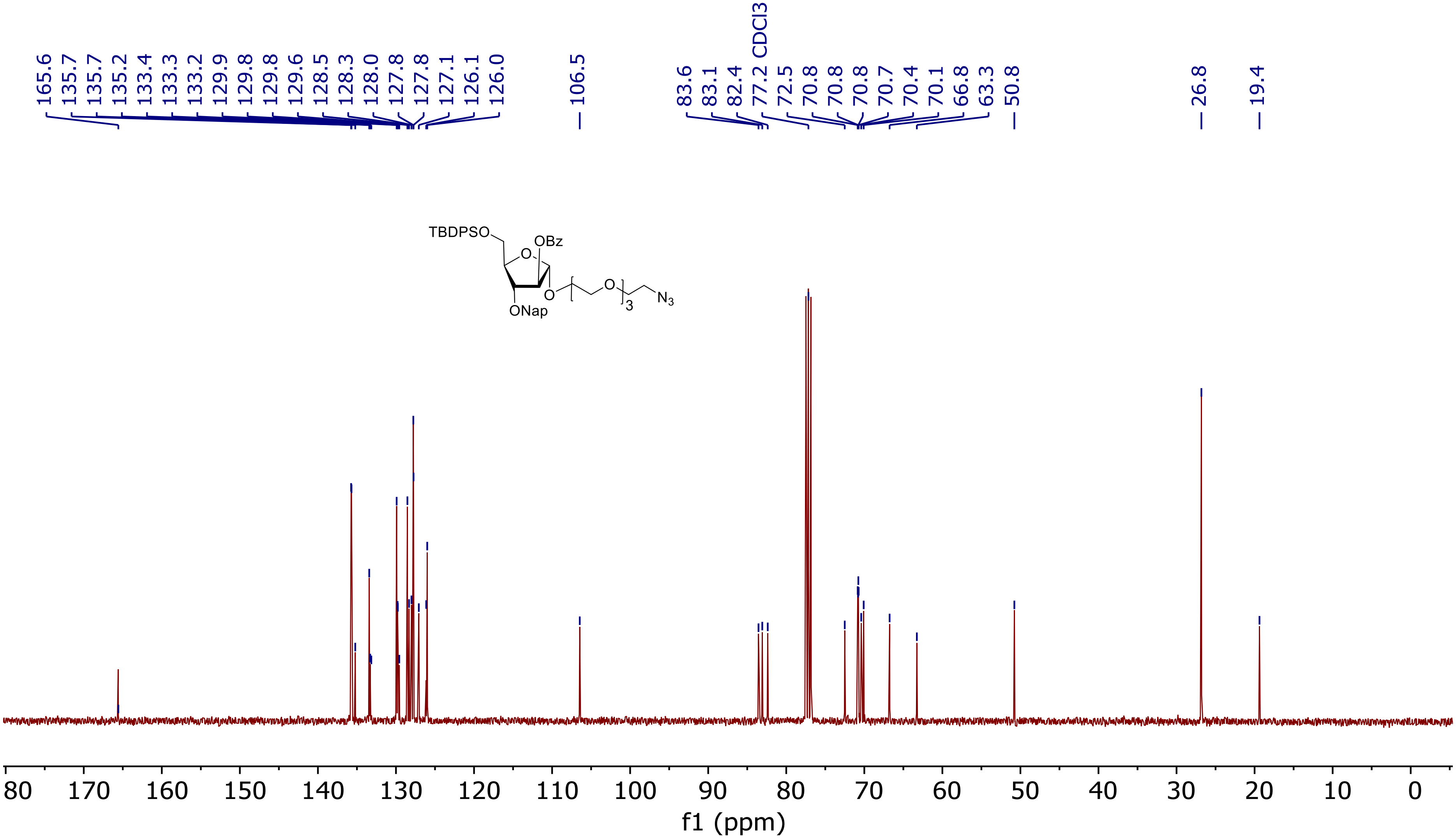

DEPT NMR Spectrum (100.67 MHz, $CDCl_3$) of **Compound 31**

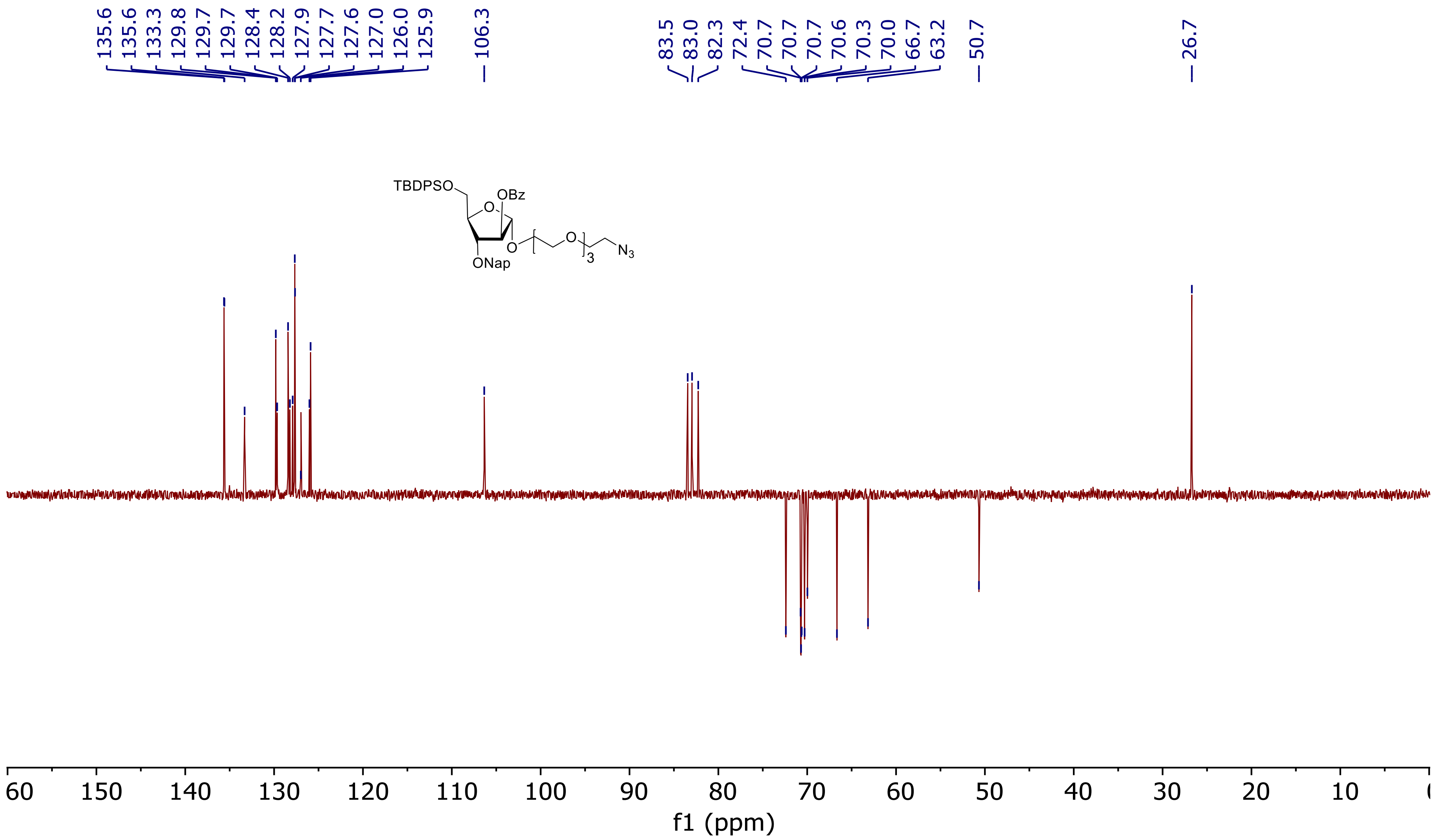

^{1}H NMR Spectrum (400.31 MHz, $CDCl_3$) of **Compound 32**

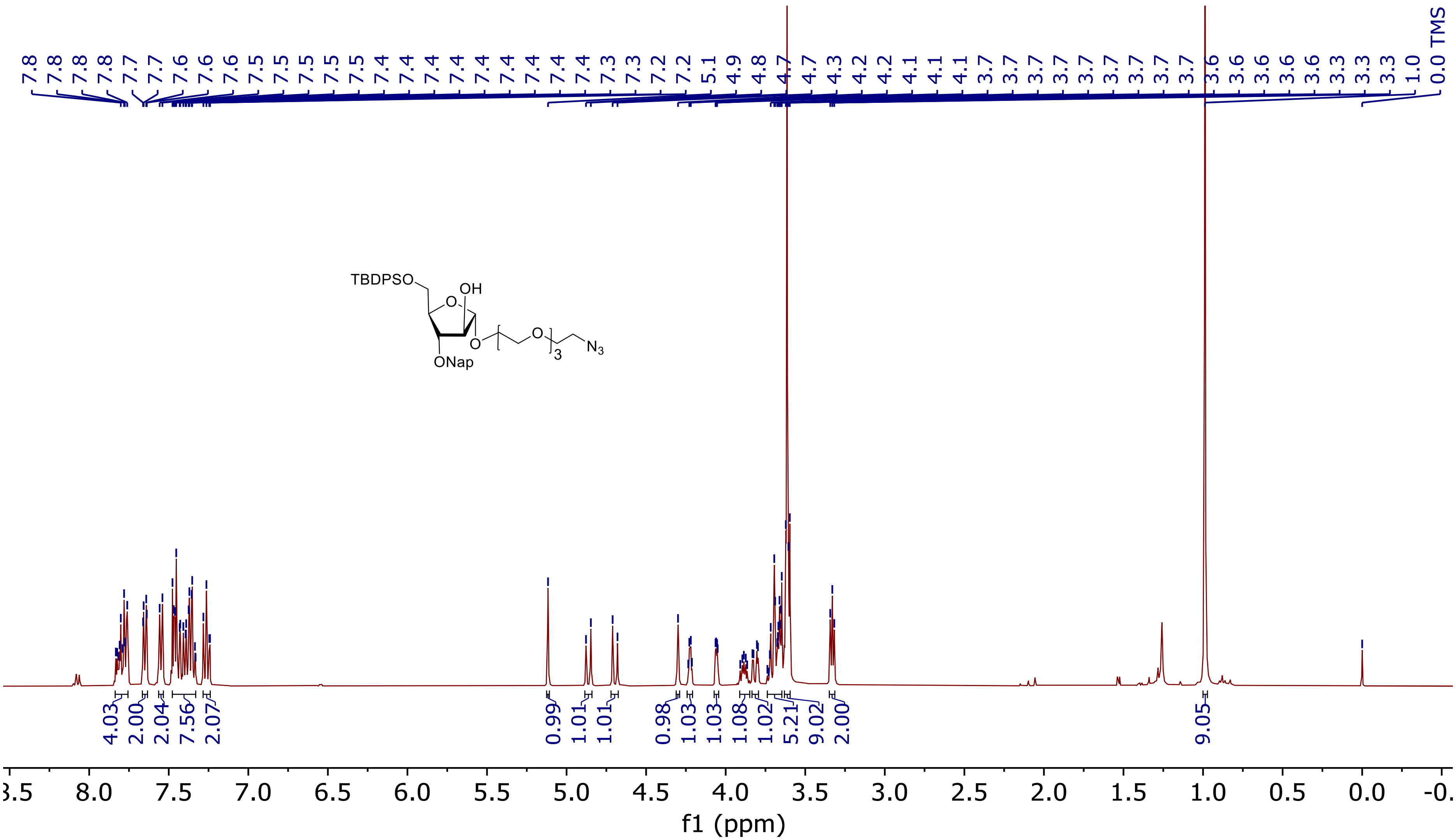

^{13}C NMR Spectrum (100.67 MHz, $CDCl_3$) of **Compound 32**

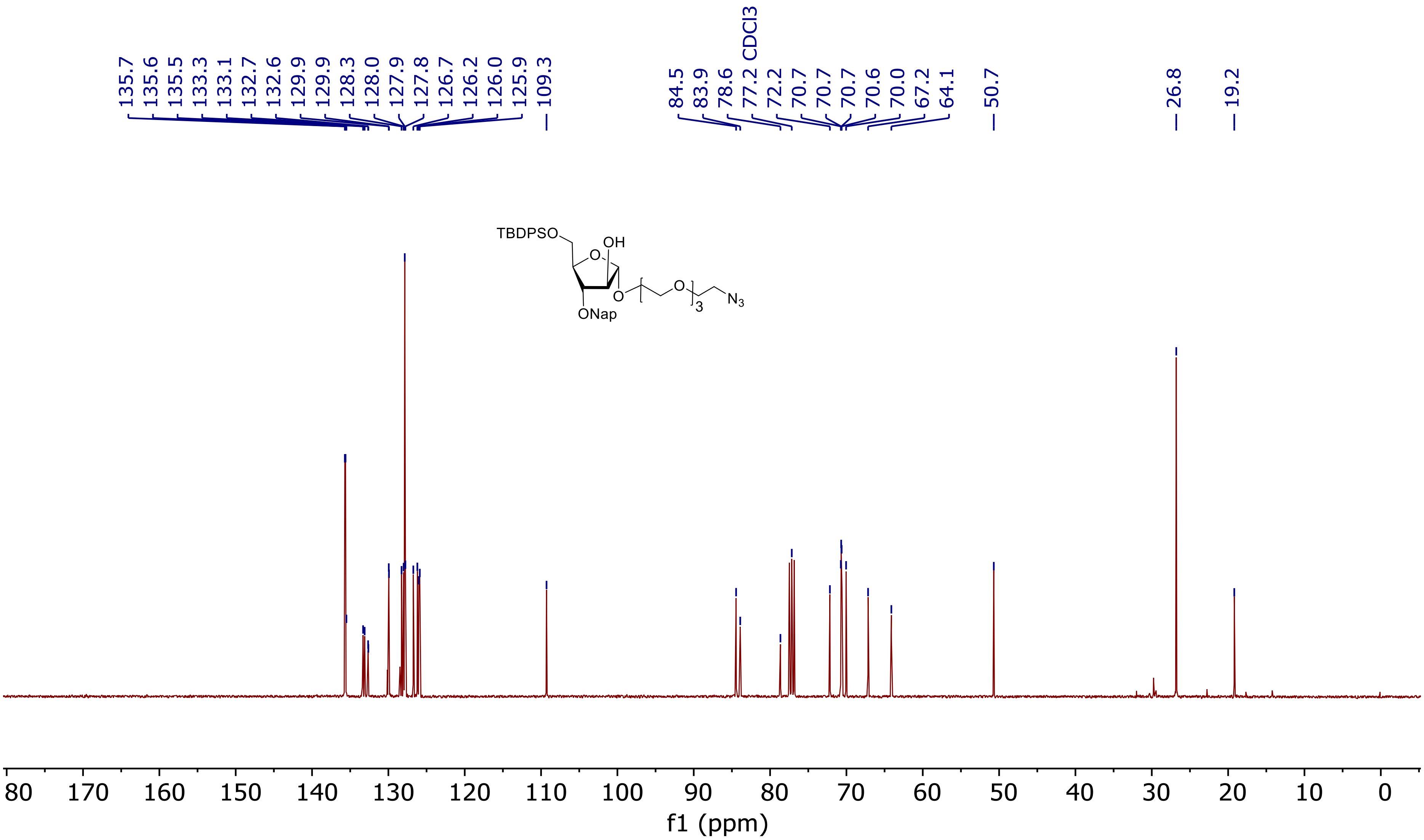

DEPT NMR Spectrum (100.67 MHz, $CDCl_3$) of **Compound 32**

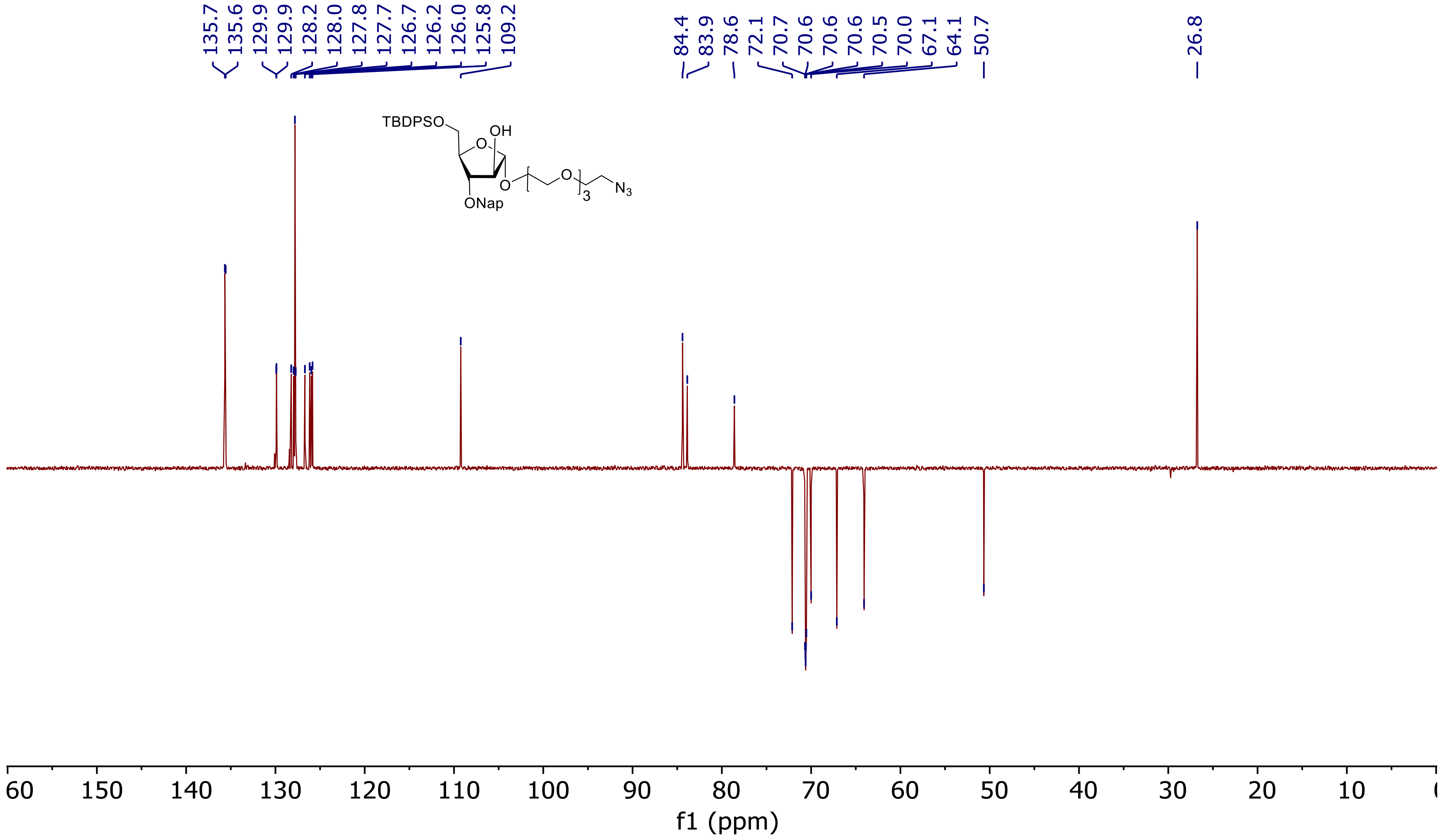

^{1}H NMR Spectrum (400.31 MHz, $CDCl_3$) of **Compound 33**

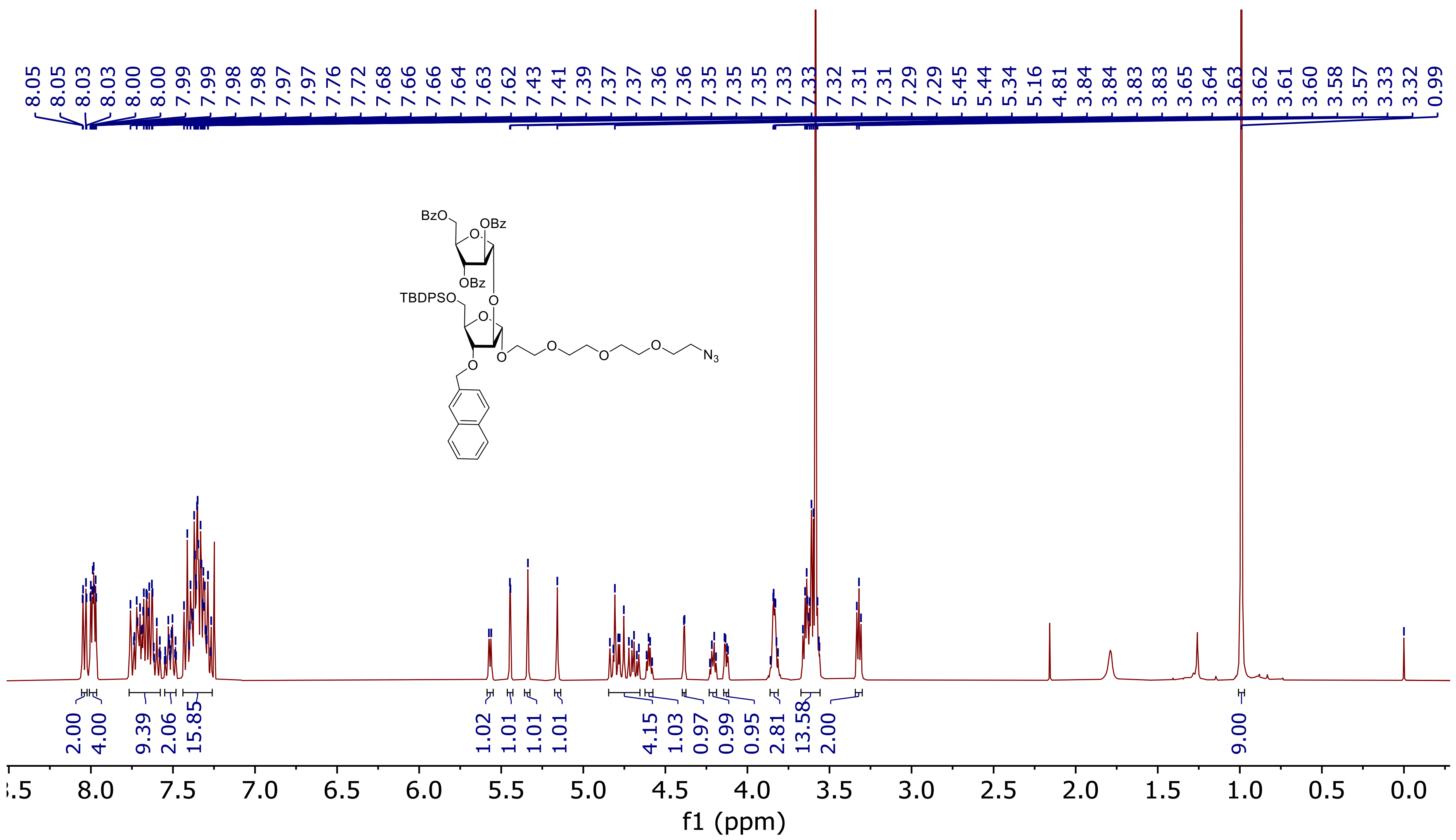

^{13}C NMR Spectrum (100.67 MHz, $CDCl_3$) of **Compound 33**

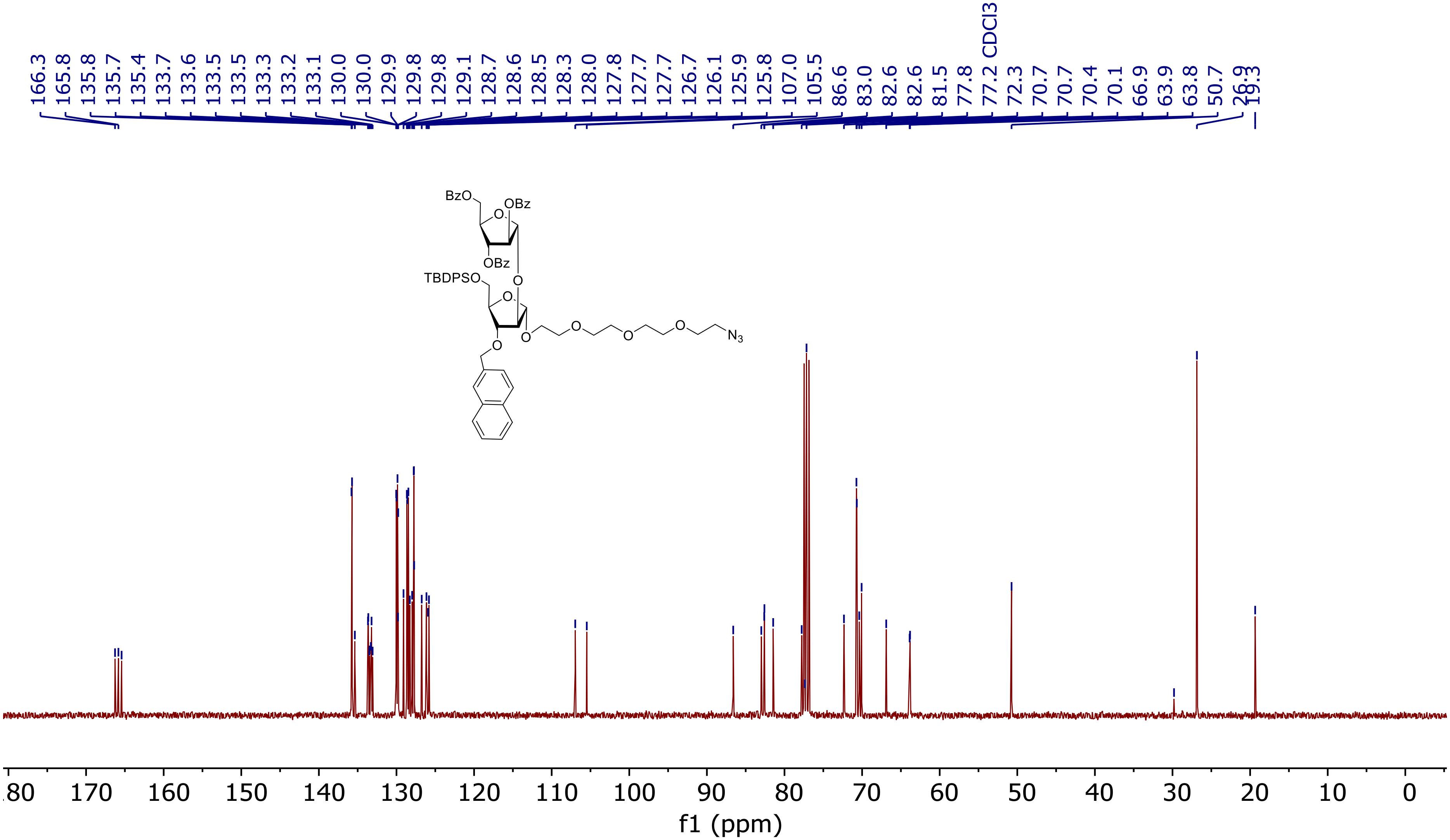

DEPT NMR Spectrum (100.67 MHz, $CDCl_3$) of **Compound 33**

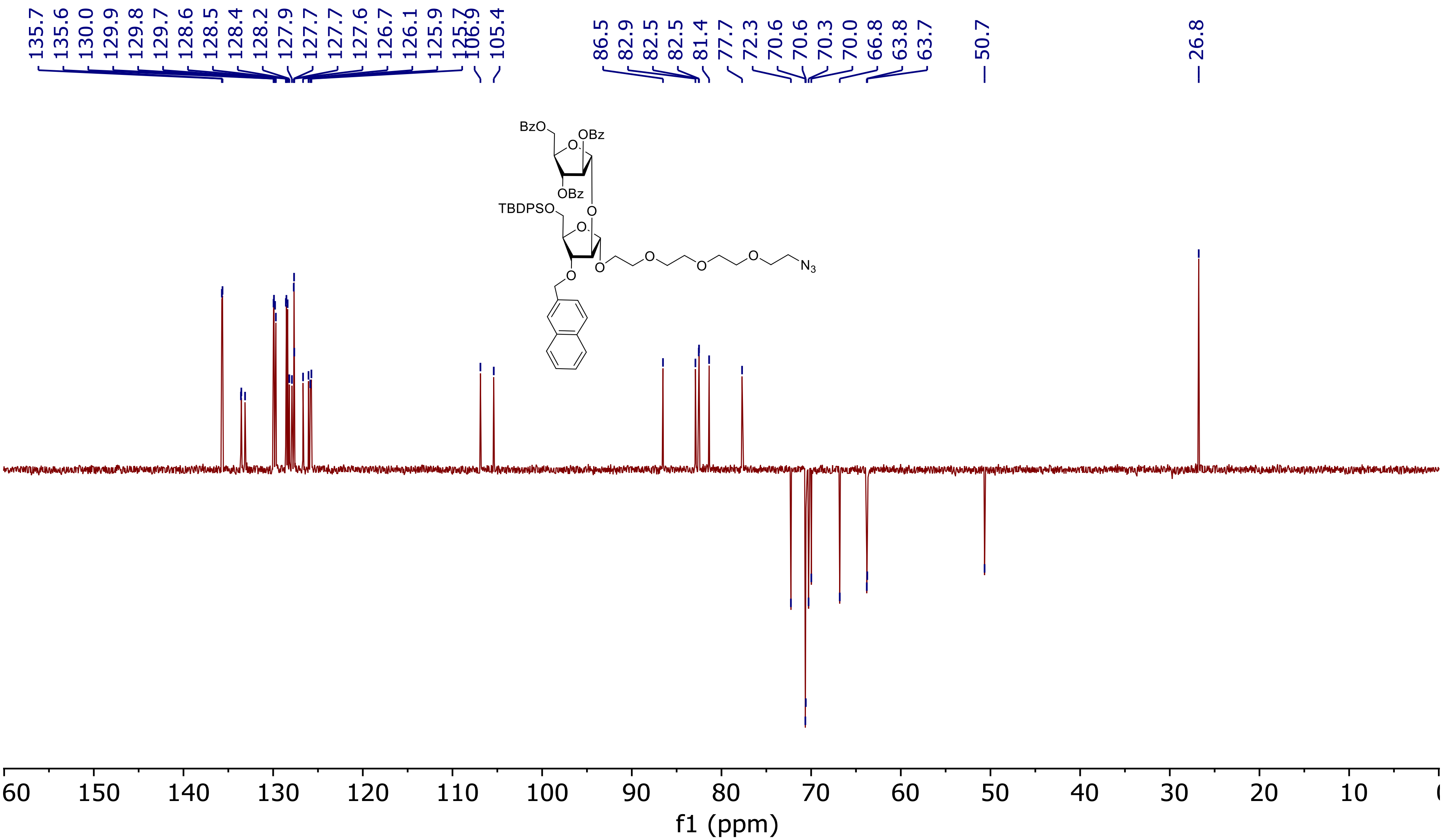

^{1}H NMR Spectrum (400.31 MHz, $CDCl_3$) of **Compound 34**

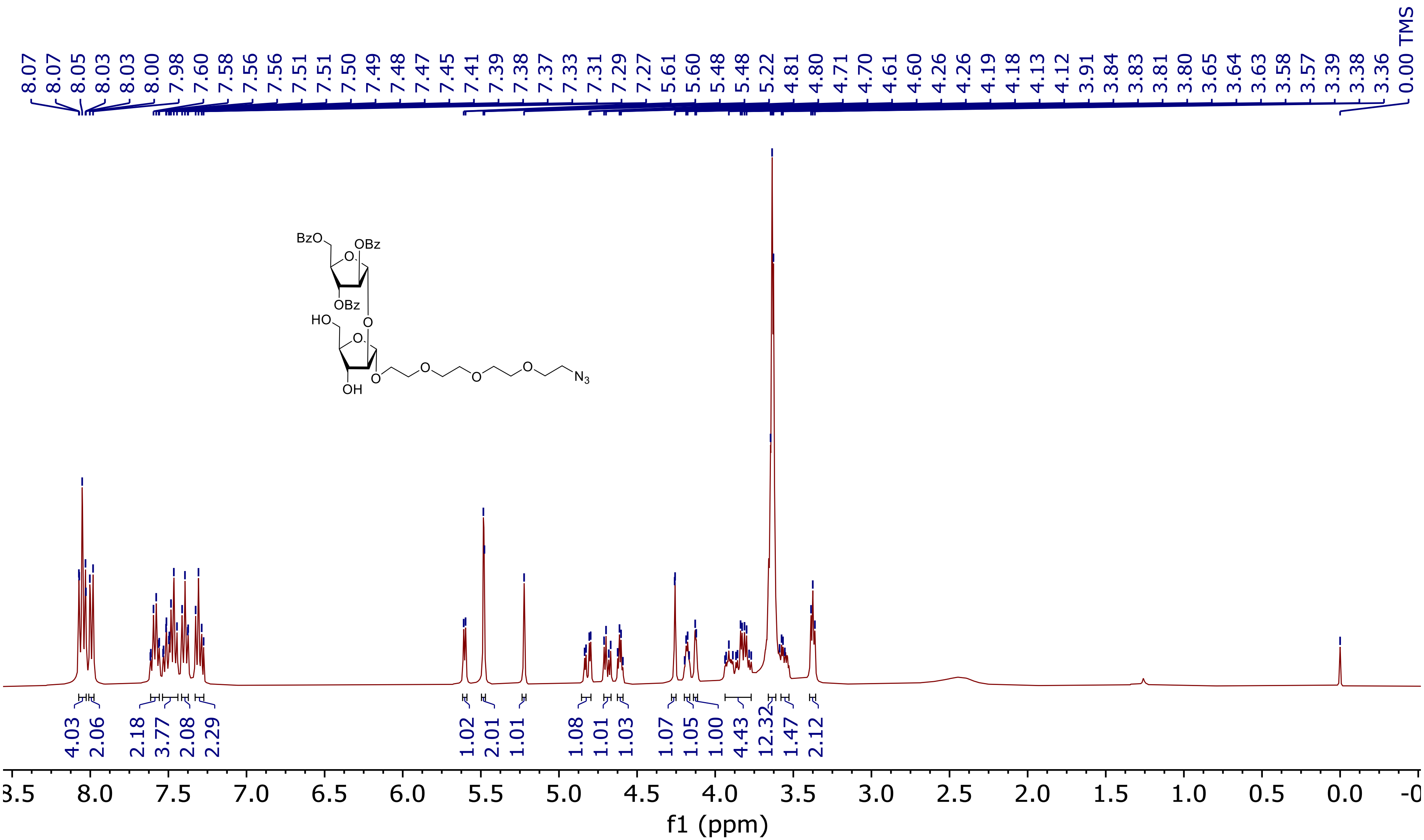

^{13}C NMR Spectrum (100.67 MHz, CDCl$_3$) of **Compound 34**

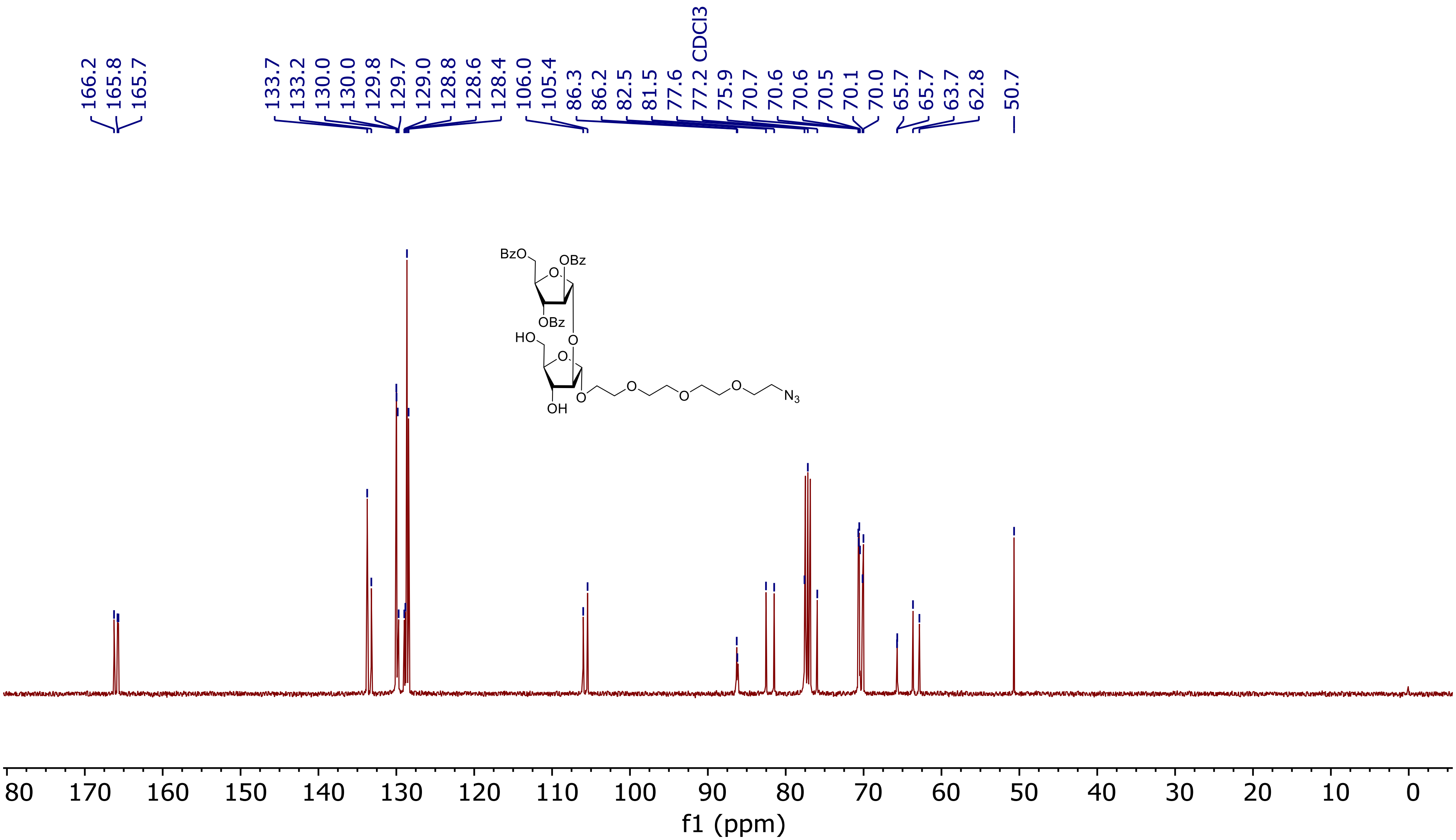

DEPT NMR Spectrum (100.67 MHz, $CDCl_3$) of **Compound 34**

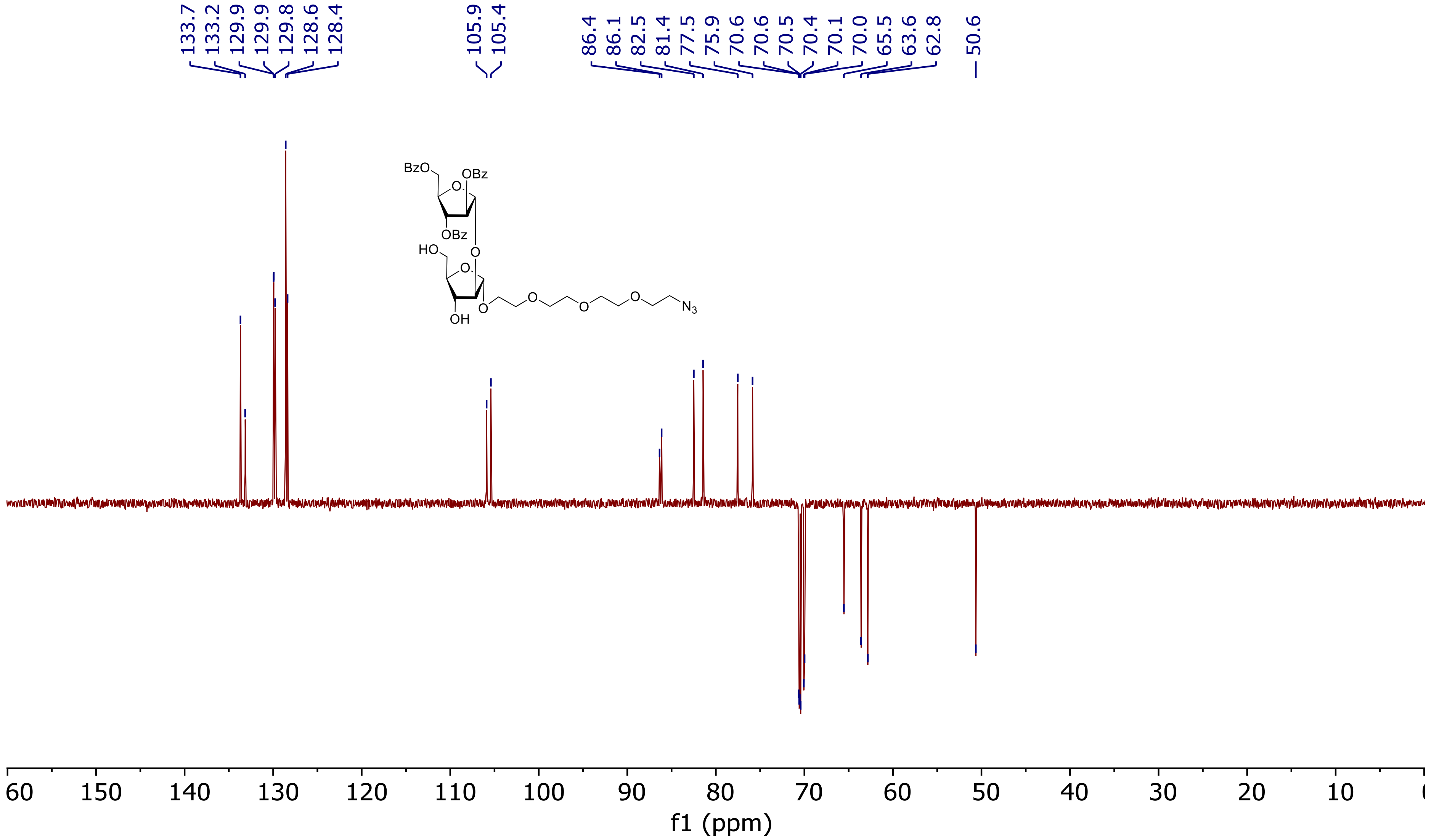

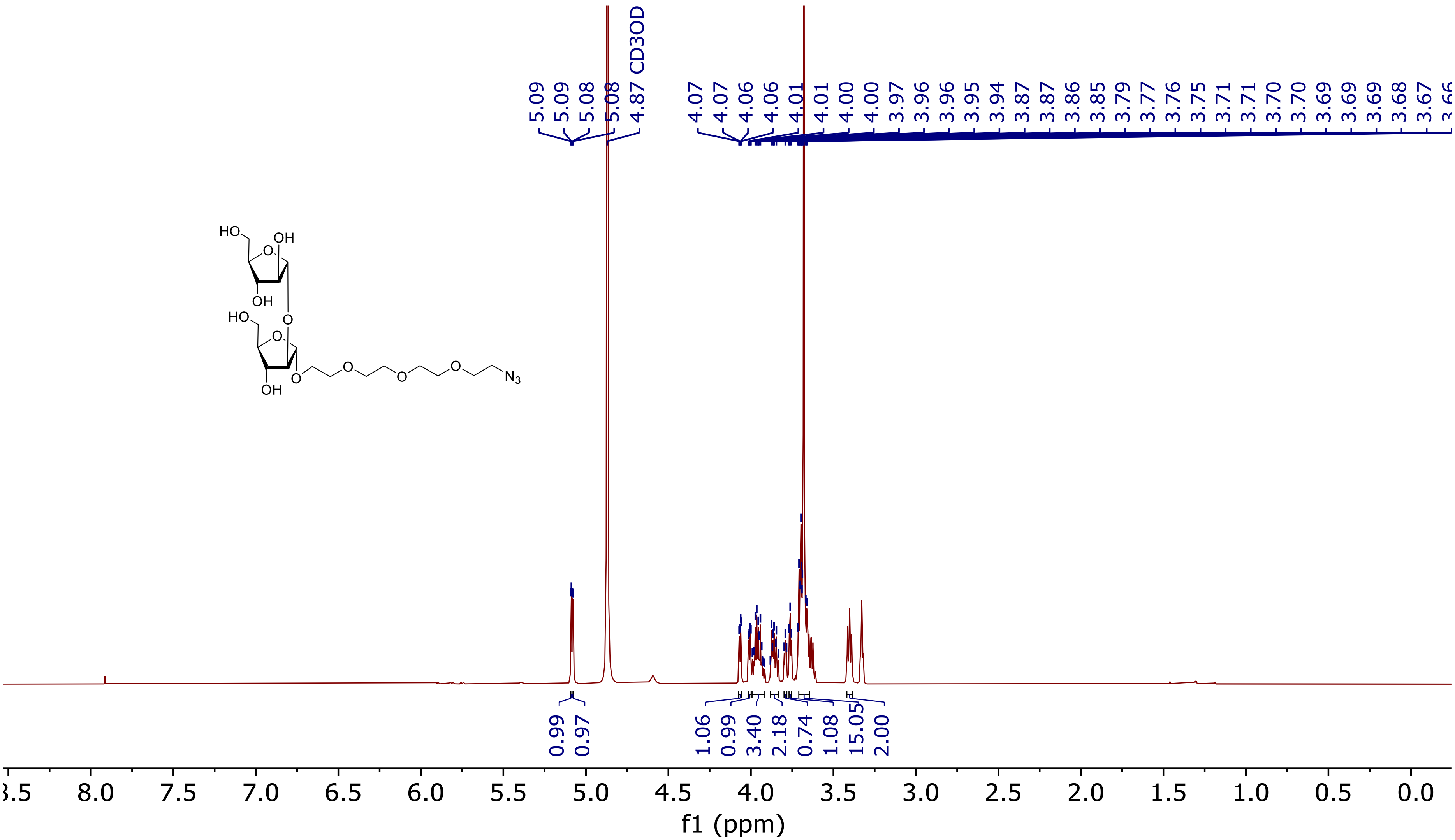

5.09
5.09
5.08
5.08
4.87 CD3OD
4.07
4.07
4.06
4.06
4.01
4.01
4.00
4.00
3.97
3.96
3.96
3.95
3.94
3.87
3.87
3.86
3.85
3.79
3.77
3.76
3.75
3.71
3.71
3.70
3.70
3.69
3.69
3.69
3.68
3.67
HO
OH
O
OH
HO
O
O
OH
O
O
O
O
N3
0.99
0.97
1.06
0.99
3.40
2.18
0.74
1.08
15.05
2.00
8.0
7.5
7.0
6.5
6.0
5.5
5.0
4.5
4.0
3.5
3.0
2.5
2.0
1.5
1.0
0.5
0.0
f1 (ppm)

^{13}C NMR Spectrum (100.67 MHz, $CDCl_3$) of **Compound 12**

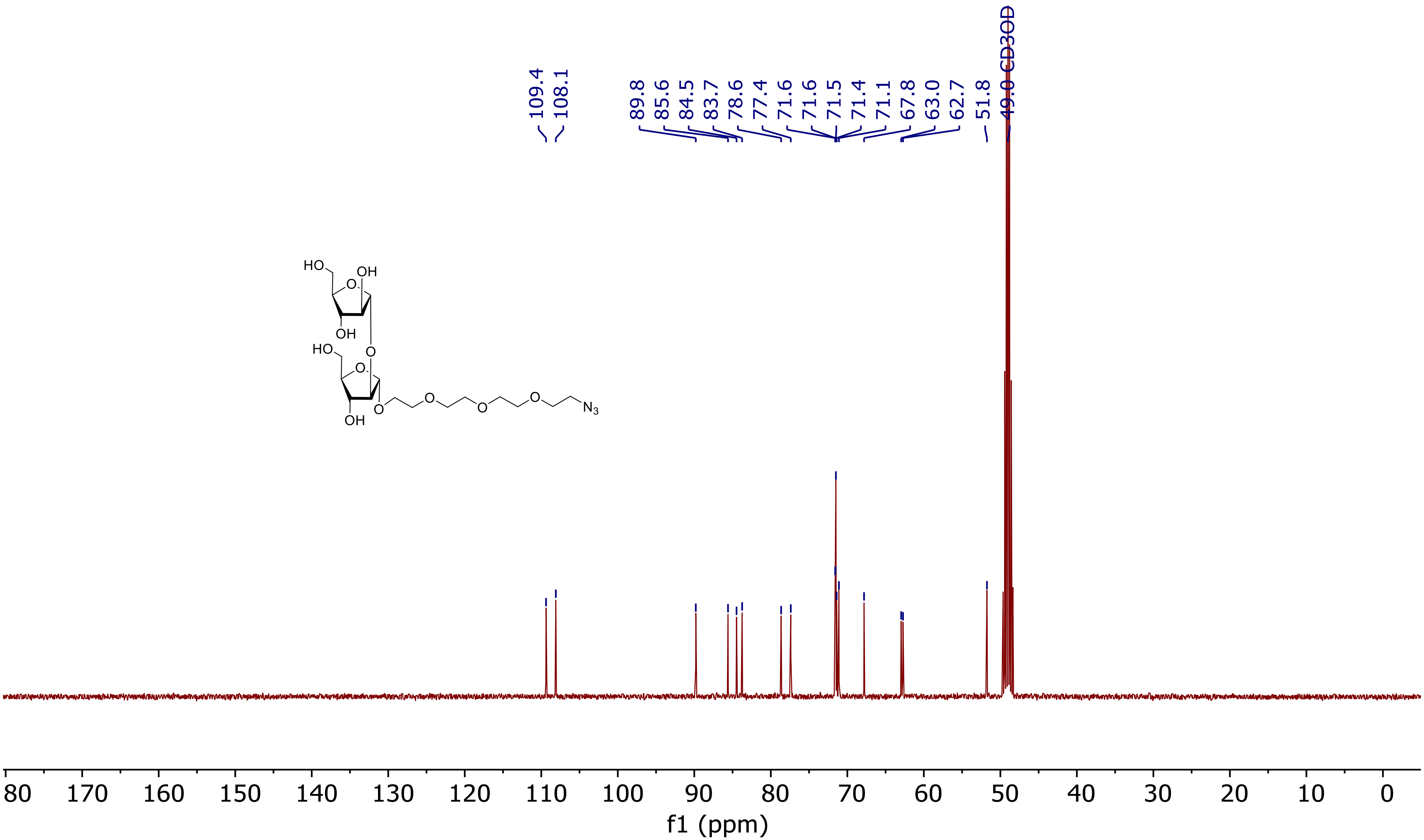

DEPT NMR Spectrum (100.67 MHz, $CDCl_3$) of **Compound 12**

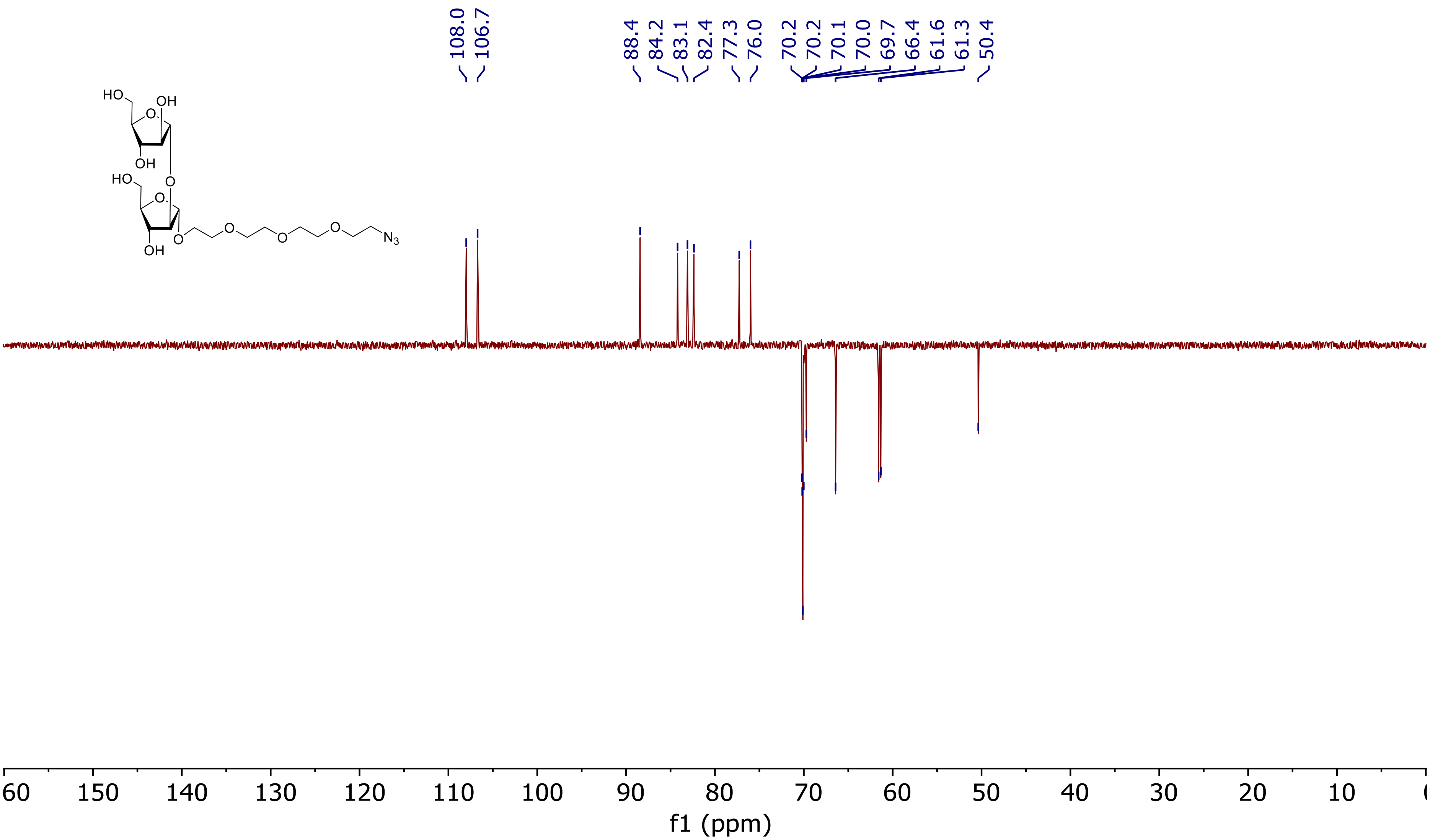

^{1}H NMR Spectrum (399.79 MHz, $CDCl_3$) of **Compound 18**

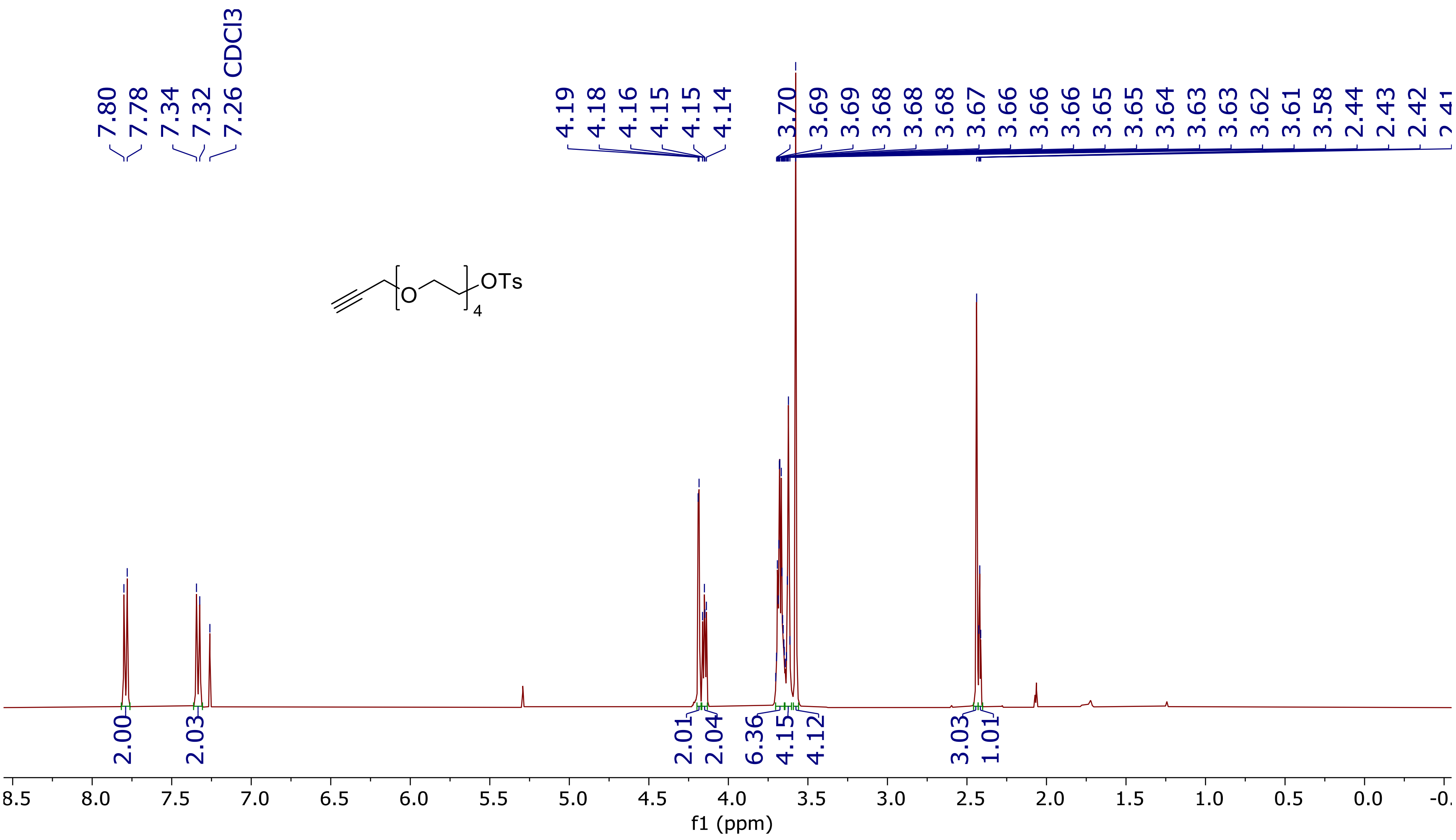

^{13}C NMR Spectrum (100.53 MHz, $CDCl_3$) of **Compound 18**

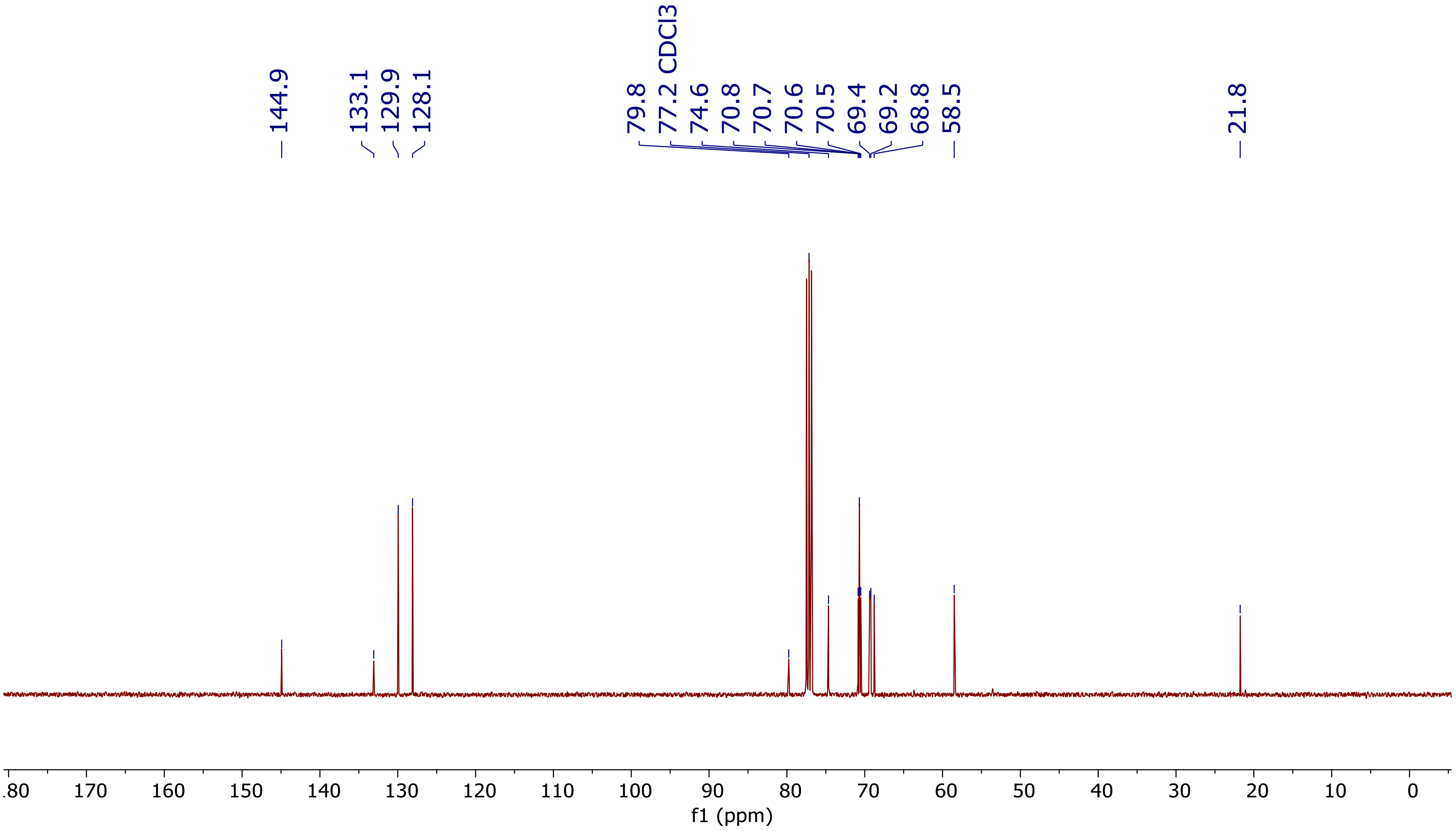

DEPT NMR Spectrum (100.53 MHz, $CDCl_3$) of **Compound 18**

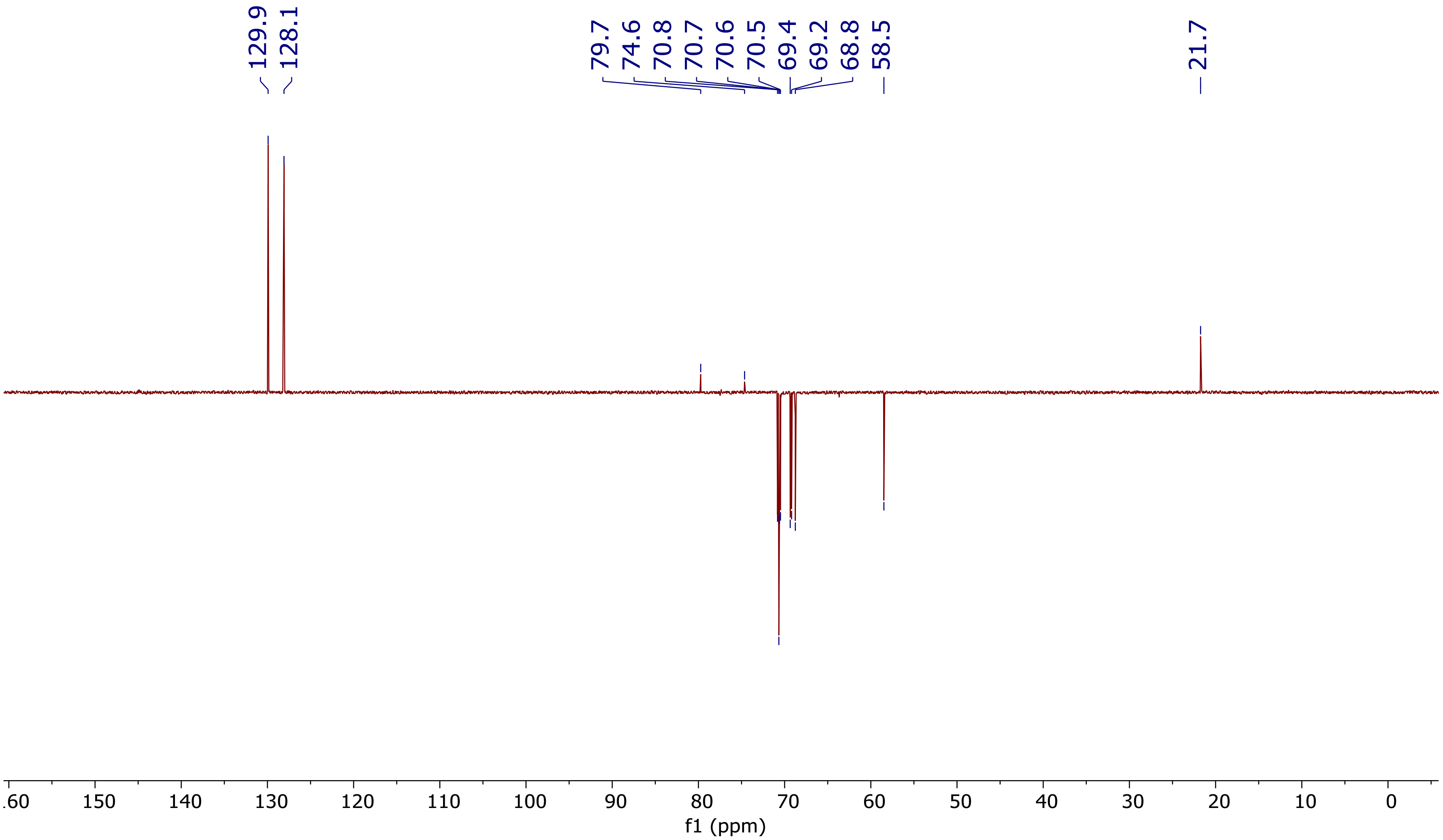

HSQC NMR Spectrum (399.79, 100.53 MHz, $CDCl_3$) of **Compound 18**

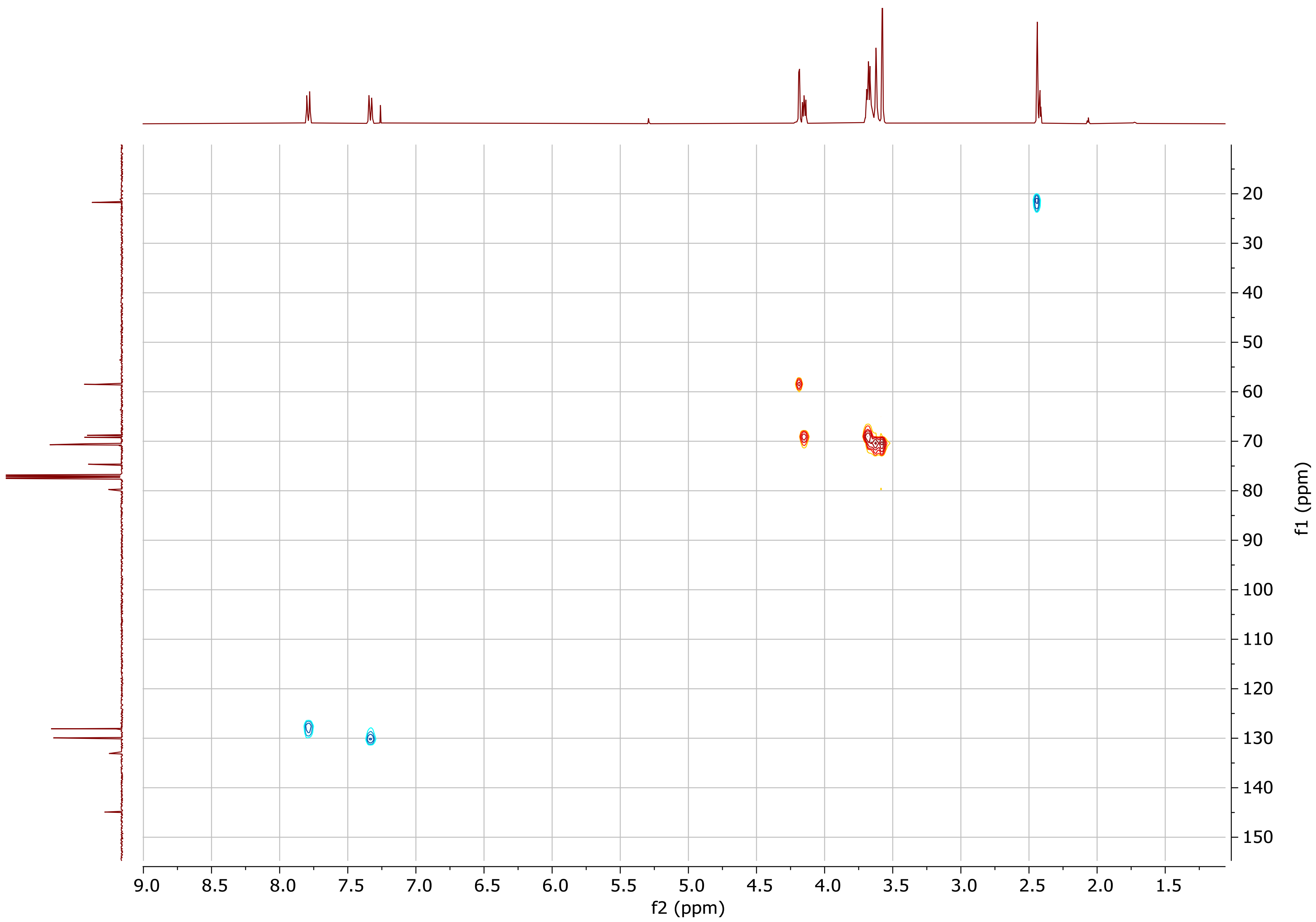

COSY NMR Spectrum (399.79 MHz, $CDCl_3$) of **Compound 18**

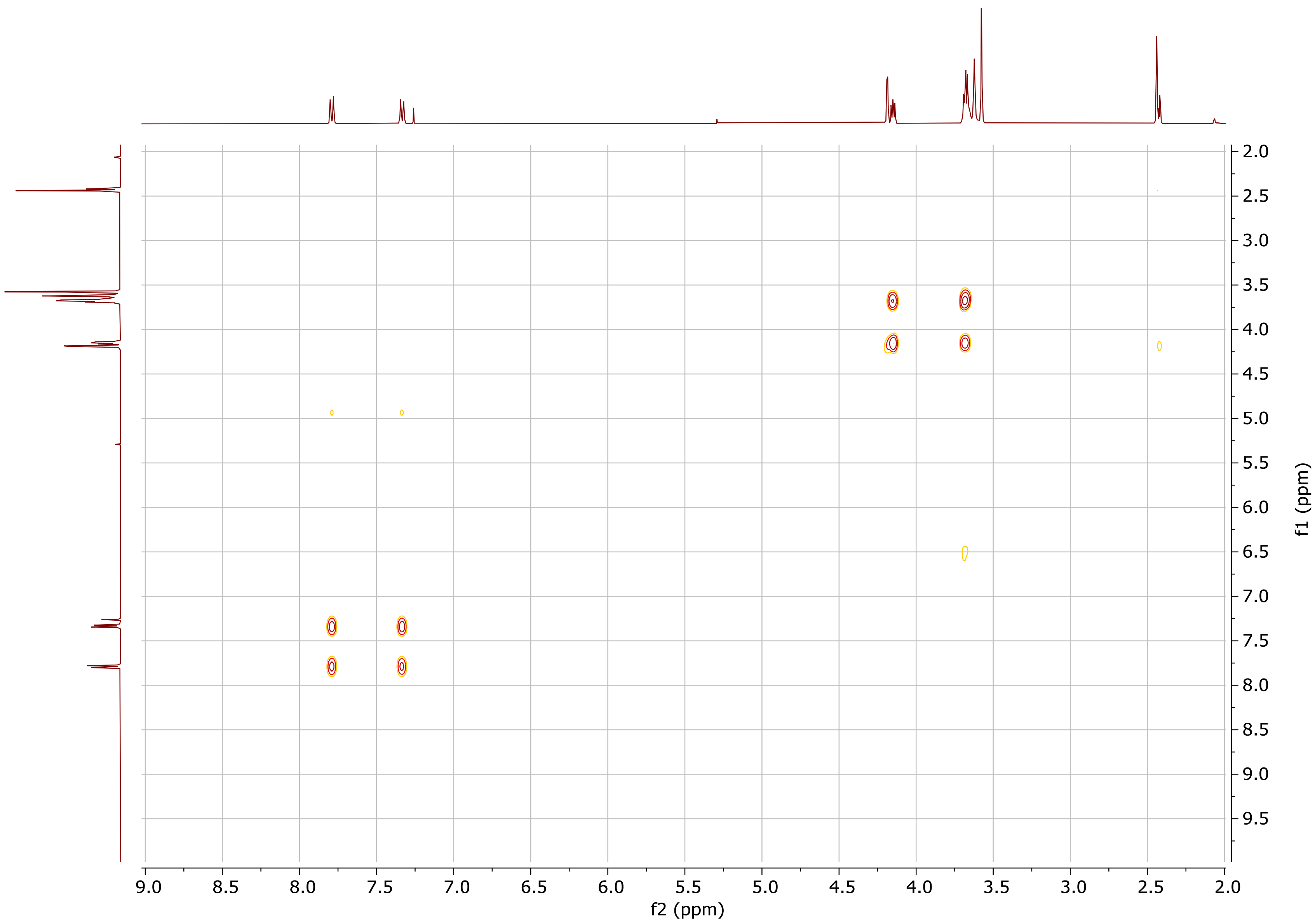

HMBC NMR Spectrum (399.79, 100.53 MHz, $CDCl_3$) of **Compound 18**

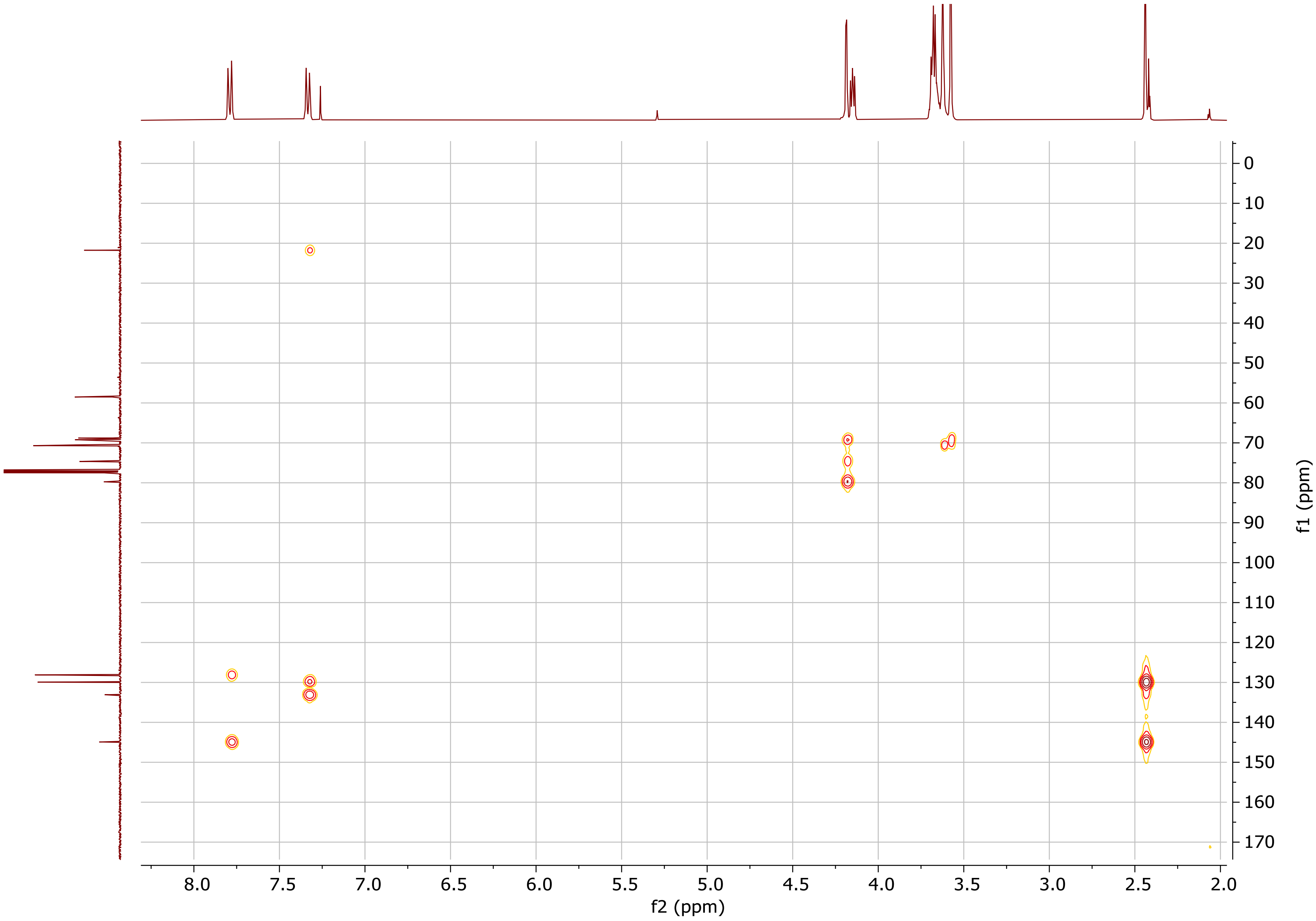

^{1}H NMR Spectrum (400.31 MHz, $CDCl_3$) of **Compound 24**

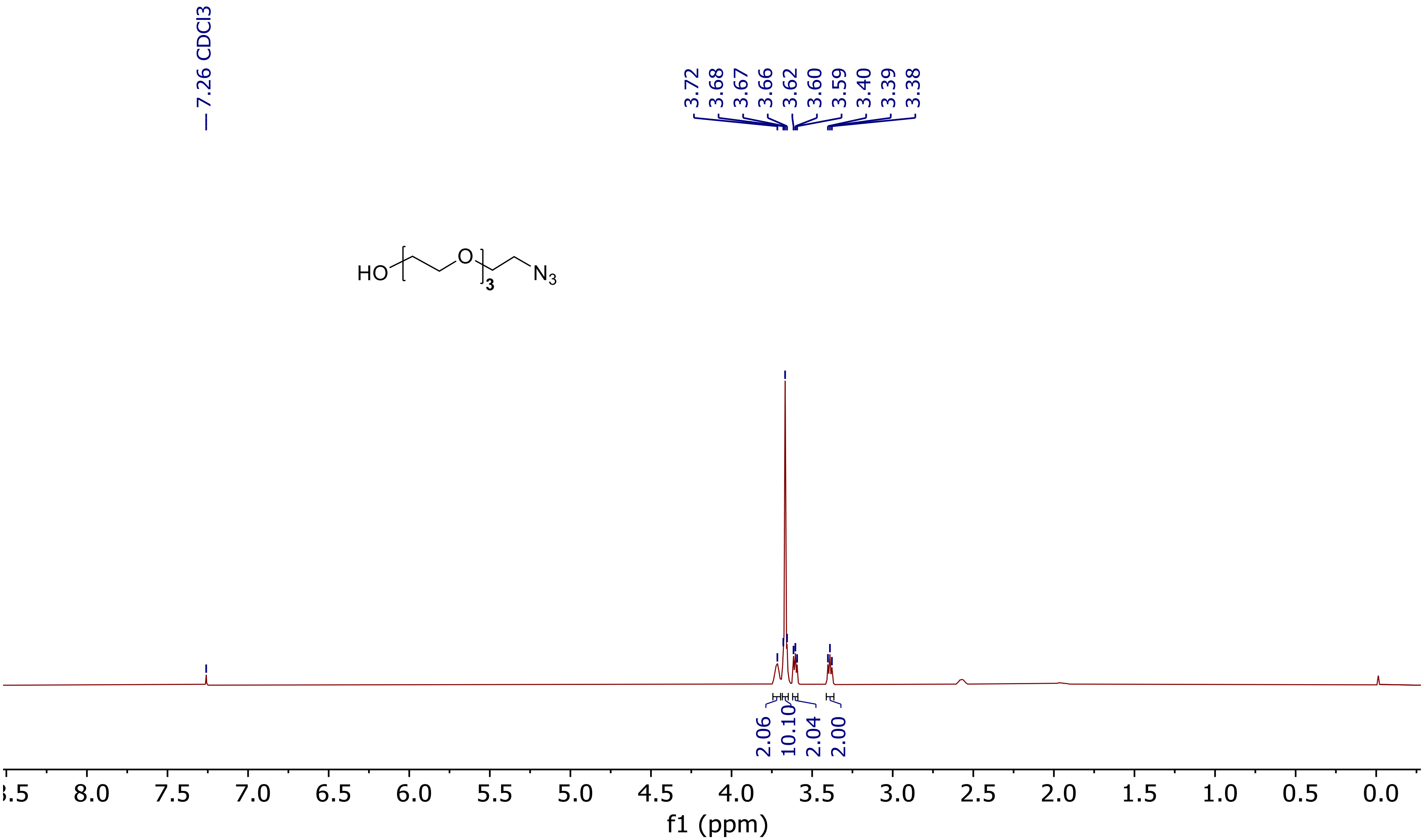

^{13}C NMR Spectrum (100.66 MHz, $CDCl_3$) of **Compound 24**

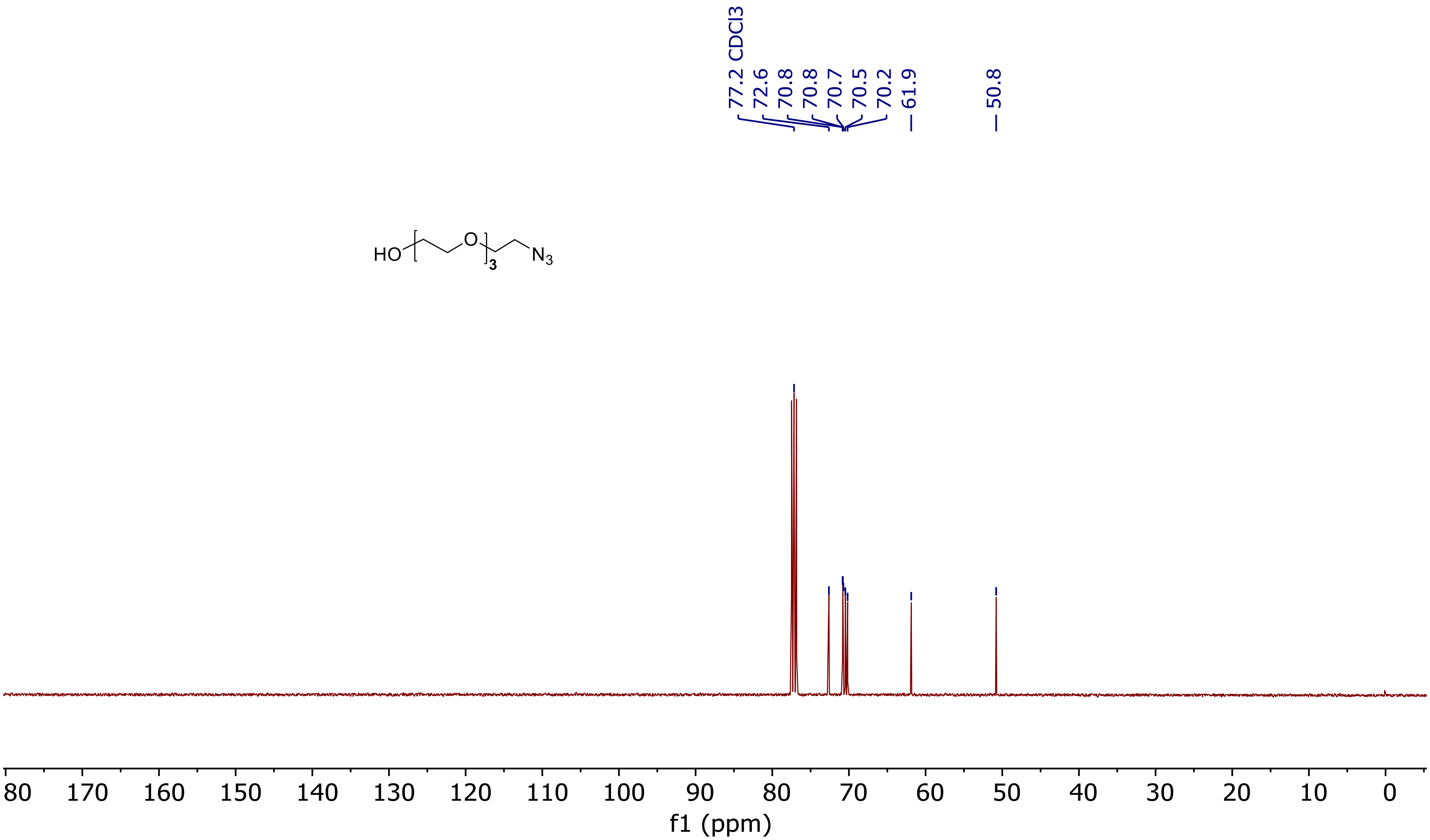

DEPT NMR Spectrum (400.31 MHz, $CDCl_3$) of **Compound 24**

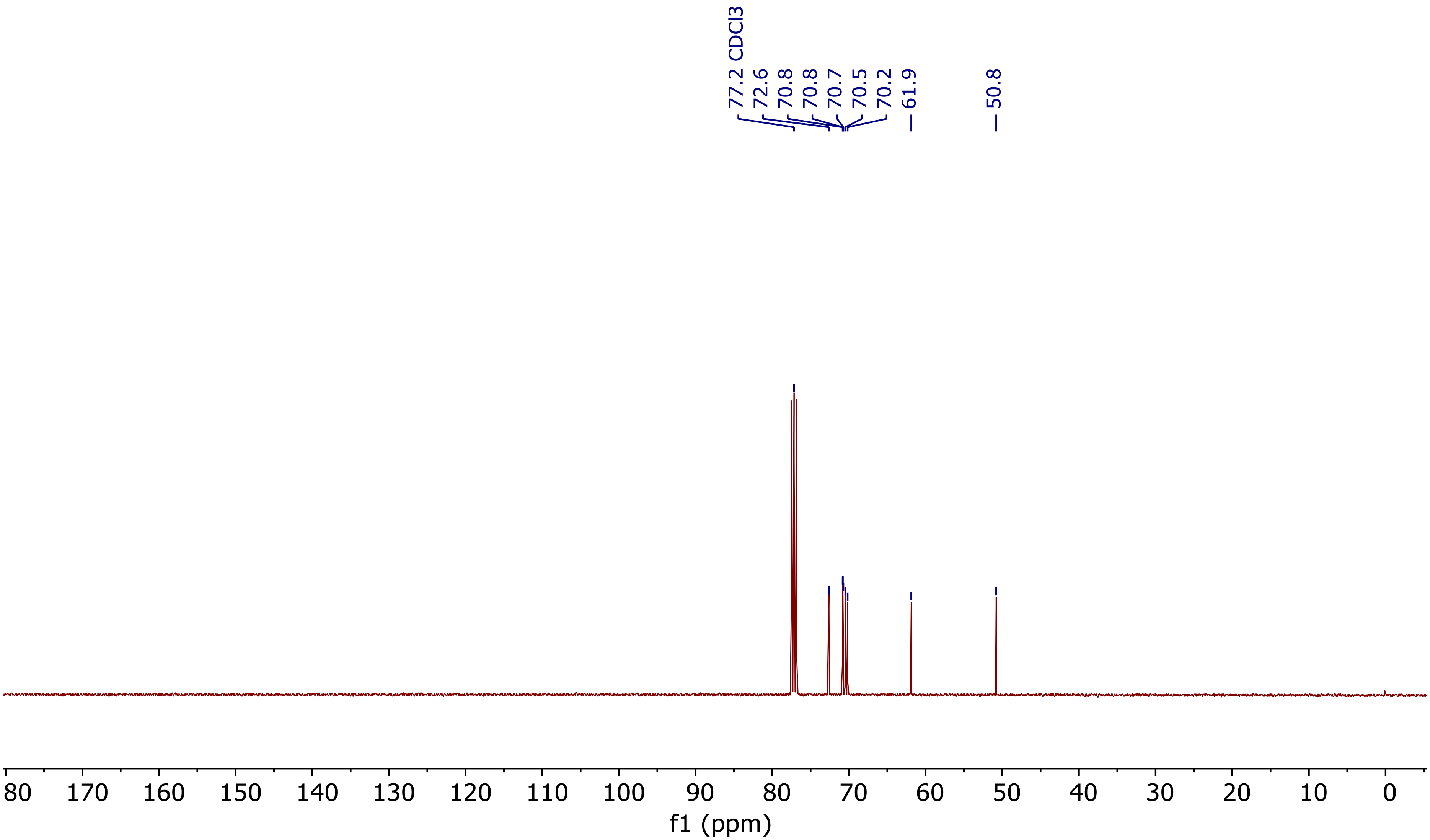

Supplement: Supplementary file 1 — au4c00931_si_001.pdf [file au4c00931_si_001.pdf]
